# Soil fungal communities show more specificity than bacteria for plant species composition in a temperate forest in China

**DOI:** 10.1186/s12866-022-02591-1

**Published:** 2022-08-30

**Authors:** Yun Chen, Jingjing Xi, Man Xiao, Senlin Wang, Wenju Chen, Fengqin Liu, Yizhen Shao, Zhiliang Yuan

**Affiliations:** 1 College of Life Sciences, Henan Agricultural University, No.63 Agricultural Road, Zhengzhou, 450002 China; 2 Institute of Botany, Chinese Academy of Sciences, Beijing, 100093 China; 3 Field Scientific Observation and Research Station of Forest Ecosystem in the North-South Transition Zone of Funiu Mountain, Zhengzhou, 450000 China; 4 College of Resources and Environment Sciences, Henan Agricultural University, No.63 Agricultural Road, Zhengzhou, 450002 China

**Keywords:** Distribution patterns, Soil microbes, Forest ecosystem, Plant population, Specialization, Niche differentiation

## Abstract

**Background:**

Soil microbiome is an important part of the forest ecosystem and participates in forest ecological restoration and reconstruction. Niche differentiation with respect to resources is a prominent hypothesis to account for the maintenance of species diversity in forest ecosystems. Resource-based niche differentiation has driven ecological specialization. Plants influence soil microbial diversity and distribution by affecting the soil environment. However, with the change in plant population type, whether the distribution of soil microbes is random or follows an ecologically specialized manner remains to be further studied. We characterized the soil microbiome (bacteria and fungi) in different plant populations to assess the effects of phytophysiognomy on the distribution patterns of soil microbial communities in a temperate forest in China.

**Results:**

Our results showed that the distribution of most soil microbes in different types of plant populations is not random but specialized in these temperate forests. The distribution patterns of bacteria and fungi were related to the composition of plant communities. Fungal species (32%) showed higher specialization than bacterial species (15%) for different types of plant populations. Light was the main driving factor of the fungal community, and soil physicochemical factors were the main driving factor of the bacterial community.

**Conclusion:**

These findings suggest that ecological specialization is important in maintaining local diversity in soil microbial communities in this forest. Fungi are more specialized than bacteria in the face of changes in plant population types. Changes in plant community composition could have important effects on soil microbial communities by potentially influencing the stability and stress resistance of forest ecosystems.

**Supplementary Information:**

The online version contains supplementary material available at 10.1186/s12866-022-02591-1.

## Introduction

Soil microbiome not only plays a critical role in regulating ecological processes relevant to nutrient cycling and carbon [1], but also produces strong positive feedback to promote plant regeneration and succession [2]. Soil microbial communities’ kind mutualistic dependent associations with soil and plants to enhance nutrient absorption [3]. The synergistic effect between the aboveground plant community and the underground microbial community contributes significantly to the restoration and stability of the ecosystem [4–7]. Mangan et al*.* (2010) reported that plant–soil feedback is an important mechanism that can maintain species diversity and explain patterns of tree-species relative abundance in forests [8]. Although soil microbes play a critical role in regulating ecological processes relevant to nutrient cycling and carbon in forest ecosystems, the distribution patterns of soil microbes with changes in plant populations remain to be further studied.

Niche differentiation is a prominent mechanism that facilitates the maintenance of species diversity in forest biomes [9–11]. One manifestation of resource-based niche differentiation consists of ecological specialization, such that different species are best suited to different habitats [9, 12–16]. If niche-relevant environmental conditions are spatially structured, then species distribution ought to be mirrored by the association between species and different habitats [16–18]. Harms et al*.* (2001) quantified the association of woody plants with topographic habitats, and their inference provides a good idea for assessing the contribution of ecological specialization to species coexistence [9]. Many researchers have since suggested that coexisting woody plants in forest ecosystems have distinct habitat preferences and emphasized the importance of ecological specialization for woody plant assemblage characteristics [9, 13, 19, 20]. Most of these studies have focused on woody plant populations [9, 18–20]. Nevertheless, the relative contribution of ecological specialization to the maintenance of diversity in soil microbial communities remains unknown.

Community structure, understory vegetation, and soil structure differ among different plant populations, which provide diverse habitats for the growth of soil microbes [21, 22]. Differences also exist between the life history and physiological traits of plants [23]. The differences among vegetation species create conditions for the specificity of soil environment and biological community [24]. Different forest types will lead to different components of litter, and the decomposition rate of plant litters will vary [25]. Moreover, soil nutrients, physicochemical properties, and light vary among different vegetation types, which affect the distribution of soil microbes. Many studies have shown that different plant species affect soil microbial diversity through root exudates and root properties [3, 26]. However, the role of plant population partitioning in soil microbe diversity maintenance remains poorly known.

Many studies have confirmed that the lifestyles of fungi and bacteria and their responses to environmental changes are different [27, 28]. Soil bacteria have stronger adaptability than fungi in the face of environmental changes [28–32]. For example, climate extremes-induced changes in plant communities had long-lasting associations with bacterial communities and strongly governed their recovery, but much less so for fungal communities [28]. However, whether bacteria and fungi have different distribution patterns in the face of plant population type changes is not well understood.

In this study, we hypothesized that ecological specialization of plant population types is important for structuring soil microbial communities in temperate mountain forest ecosystems and hence would be important for the maintenance of their local species diversity. To test these hypotheses, we characterized soil bacterial and fungal communities to determine their spatial distribution in 18 woody plant populations belonging to six community types in a temperate forest in China. We examined the distribution preferences of soil microbes (soil bacteria and fungi) at the community level (correlation network) and species level (torus-translation test). We also evaluated the influence of the environment on microbial community among different plant populations by variance partitioning. Results can improve understanding of the distribution patterns of fungi and bacteria in response to changes in plant population types in forest ecosystems.

## Materials and methods

### Site description

This study was conducted in Baiyun mountain in Luoyang City of Henan province, east China (33°38'–33°34' N, 111°48'–111°52' E, 1500 m above sea level). The total area is 168 km^2^, and the mean annual temperature is 18℃, with an average temperature of 0.2℃ in the coldest month (January) and 27.3℃ in the hottest month (July) [33]. The average annual precipitation is 1200 mm [34–36].

The forest canopy closure is 98.5% in Baiyun mountain. The dominant species in the forest are *Quercus aliena* var. *acutiserrata*, *Forsythia suspensa*, *Pinus armandii*, *Quercus serrata* var. *brevipetiolata*, *Sorbus hupehensis*, and *Larix gmelinii* [37].

### Sampling design

Differences in soil microbial distribution under different communities were explored. Based on a comprehensive investigation, 18 communities belonging to six community types were selected in the Baiyun Mountain National Nature Reserve. The six community types are *Quercus aliena* var. *acutiserrata* community (QAV), *Quercus serrata* var. *brevipetiolata* community (QSV), *Larix gmelinii* community (LAG), *Pinus armandii* community (PIA), *Forsythia suspensa* community (FOS), and *Sorbus hupehensis* community (SOH) (Fig. S1). These communities are the main community types in this area. QAV and QSV belong to broad-leaved forests, LAG and PIA belong to coniferous forests, and FOS and SOH belong to shrub forests. Table S1 shows detailed information about the six communities. Three 20 m × 20 m quadrats were set in each type of community. All woody plants ≥ 1 cm diameter at breast height (DBH) in each 20 m × 20 m quadrat was identified, measured, and recorded.

In each 20 m × 20 m quadrat, topsoil (0–20 cm) was selected and three replicates were randomly sampled with a soil corer. After the visible stones, roots, and litter were removed, three samples were mixed to obtain one composite sample. Finally, 18 mixed soil samples were obtained. Each soil sample was divided into two parts: one part was immediately stored at − 80 °C until DNA extraction, and the other part at 4 °C until analysis of soil physicochemical properties.

### DNA extraction, amplification, and high-throughput sequencing

Soil total DNA was extracted from 0.5 g of the freeze-dried soil samples by using FastDNA® Spin Kit for Soil based on the manufacturer’s instructions. The reaction mixture had a total volume of 20 μL, including 10 ng of template DNA, 2 μL of 2.5 mM dNTPs, 4 μL of 5 × TransStart FastPfu Buffer, 0.4 μL of TransStart FastPfu DNA Polymerase, 0.8 μL of 5 μM forward primer, 0.8 μL of 5 μM reverse primer, and finally ddH_2_O up to 20 μL. PCR conditions were 3 min at 95 °C, followed by 27 cycles of 30 s at 95 °C, 30 s at 55 °C, and 30 s at 72 °C; and a final extension at 72 °C for 10 min. PCR reactions were performed in triplicate. The hypervariable region V4 of the bacterial 16S rRNA [38] gene was amplified with primer pairs 515F(5ʹ-GTGCCAGCMGCCGCGGTAA-3ʹ) and 806R(5ʹ-GGACTACHVGGGTWTCTAAT-3ʹ) by an ABI GeneAmp® 9700 PCR thermocycler (ABI, CA, USA). The hypervariable region ITS1 of the fungal ITS gene [39] was amplified with primer pairs ITS1F (5ʹ-CTTGGTCATTTAGAGGAAGTAA-3ʹ) and ITS2R (5ʹ-GCTGCGTTCTTCATCGATGC-3ʹ) by an ABI GeneAmp® 9700 PCR thermocycler (ABI, CA, USA). The PCR products were separated on 2% agarose gels and purified using AxyPrep DNA Gel Extraction Kit (Axygen Biosciences, Union City, CA, USA) [39]. All purified amplicons were sequenced (2 × 300) on Illumina Miseq platform of Majorbio Bio-Pharm Technology Co. Ltd (Shanghai, China) [39].

Sequencing of bacterial and fungal raw data yielded 14,455,502 and 12,614,520 reads, respectively. The raw gene sequencing reads were demultiplexed, quality filtered by Trimmomatic, and incorporated by Fast Length Adjustment of Short reads (FLASH, v1.2.11) with specific criteria. After the demultiplexed and quality control, the number of bacterial and fungal sequences were 7,320,500 and 6,584,900, respectively. Operational taxonomic units (OTUs) with 97% similarity cutoff [40] were clustered using UPARSE (version 7.1, http://drive5.com/uparse/) from bacteria and fungi. Chimeric sequences were identified and removed. The taxonomy of each OTU representative sequence was analyzed by RDP Classifier (http://rdp.cme.msu.edu/) against the 16S rRNA and ITS database (e.g. Silva SSU128) by using confidence threshold of 0.7 [41].

### Environmental data

The total station was used to record the elevation of the plots. Slope, convex–concave, aspect, and mean elevation were calculated based on the elevation of the plots. Topographic factors were calculated using the methods of Harms et al.(2001) and Valencia et al.(2004) [9].

SLM9-UM-1.2 canopy analyzer (Delta-T Devices Co, Ltd.) was used to collect light environment data [42]. Light environment parameters include light transmittance (LT), scattered radiation (SR), total radiation (TR), canopy cover (CC), average leaf angle (ALA), and leaf area index (LAI).

Soil physicochemical properties evaluated include pH, soil moisture content (SWC), nitrogen (N), phosphorus (P), and soil organic matter (SOM) content [43–46]. Differences in environmental factors (topographic, light, soil) among different communities are shown in Figure S2.

### Data analysis

Three microbial groups, namely, all species, core species, and dominant species, were constructed to understand the species information of different groups. We defined core species as those having relative abundances above 10% of all species [47–49], and dominant species as those having relative abundances above 0.5% of all species [50]. Core and dominant species have an important ecological role in microbiome assembly and ecosystem functions [51, 52].

Venn diagrams were plotted to show the number of OTUs that are unique to and shared among different communities and visualized using the VennDiagram package in R [53]. Rarefaction curves of the observed bacterial and fungal OTUs were calculated in each bacterial and fungal community (i.e. all 18 quadrats) by using the Specaccum command within the VEGAN package [54]. Percentage stacking diagrams were generated using R to show the relative abundance of dominant bacterial and fungal communities at genus and species levels [55]. The species composition of bacteria and fungi was analyzed by ordination using nonmetric multidimensional scaling (NMDS) with Bray Curtis dissimilarity, and the different types of communities were fitted as centroids onto the NMDS graph using the envfit function. NMDS was conducted using the metaMDS command within the VEGAN package [16, 54].

Network analysis was used to analyze the specificity of microbes to different plant populations. Network analysis data were visualized using Gephi software [56]. The network was compartmentalized into six modules of closely associated bacteria and fungi. The structure of the community–microbe network was evaluated using modularity index [54]. This parameter was calculated using the network-level function of the bipartite package [57].

Torus-translation test is currently the most used method for determining the association between species and habitat [19, 58, 59]. This test calculates the probability of the true distribution of a species in each habitat under the condition of random distribution and determines whether a species is significantly correlated with a certain type of habitat through probability analysis. In addition, the test considers the spatial autocorrelation of species distribution (Harms et al., 2001; for details) [9]. In the present study, torus-translation test was used to examine the associations between microbes (11,610 OTUs of bacteria and 4340 OTUs of fungi) and community in Baiyun mountain. Prior to data analysis, OTUs with relative abundances less than 0.01% were removed [48]. The associations of all species, core species, dominant species with community type (positive correlations, *P* ≤ 0.05) were analyzed by torus-translation test.

Variance partitioning was used to distinguish the results of various environmental factors on microbial communities (all species, core species, dominant species) [60]. Variations in species compositions in soil bacteria and fungi were partitioned into topographical factors (aspect, slop, elevation, and convex and concave), soil physicochemical properties (pH, P, N, SWC, and SOM), and light (LT, SR, TR, CC, LAI, and ALA) by using the Varpart function in the VEGAN package [54].

All statistical analyses were conducted in R 4.0.3.

## Results

### Change to microbiome composition among different phytophysiognomies

**Fig. 1:**
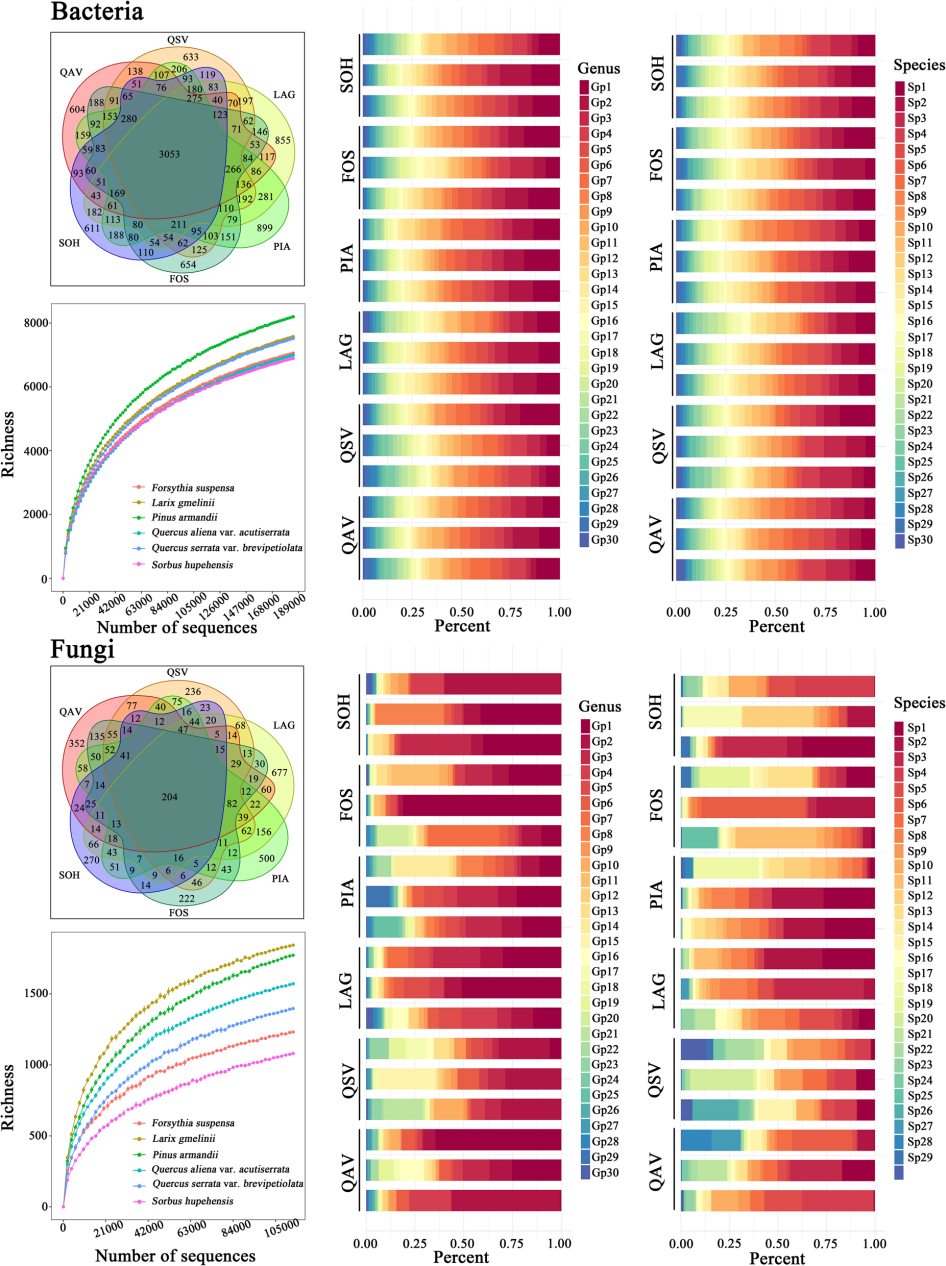
Venn diagrams showing the number of OTUs that were unique to and shared among different communities. Rarefaction curves of the observed OTU numbers of all the samples from the six types of communities after rarefied. Percentage stacking diagrams show the community composition of bacteria and fungi at genus and species levels. The top 30 genera and species were selected for abundance analysis. Three quadrats were set for each population, with 18 quadrats in 6 populations. The abscissa is the proportion of species in the sample, and the ordinate is the plot. Different colored columns represent different species, and the length of the columns represents the proportion of the size of the species. The abbreviations of species are shown in Supplementary Table S4

The Venn diagram showed that the OTU numbers of bacteria and fungi varied among different communities (Fig. 1). The highest number (8200) of OTUs of bacteria was found in the PIA community. The maximum (1844) number of OTUs of fungi was detected in LAG community.

**Fig. 2:**
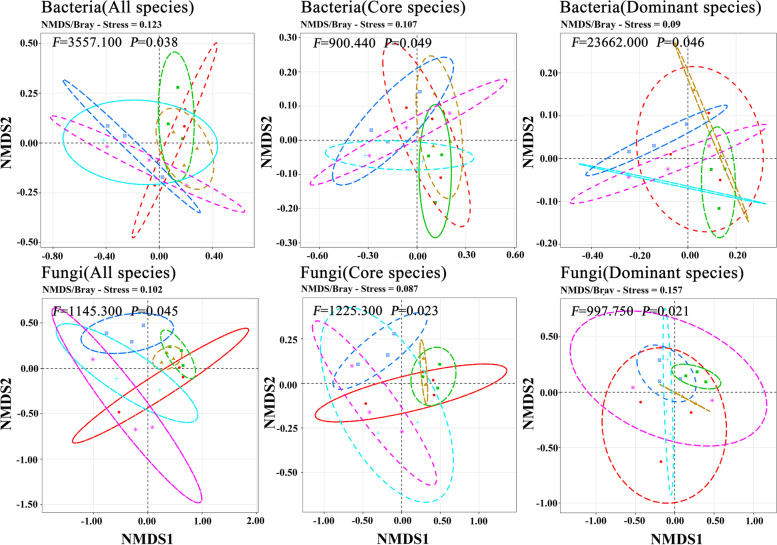
NMDS analysis of species composition among the six types of communities. Different colored dots indicate different types of communities. The ellipse has a 95% confidence interval. PERMANOVA was used to evaluate intercommunity significance

The rarefaction curve tended to flatten as the number of measured sequences increased (Fig. 1). At both genus and species levels, the diversity of fungal richness of the six plant populations was greater than that of bacteria (Fig. S3). The percentage stacking diagrams showed that the dominant species of bacteria at the genus and species levels were similar, but their relative abundance was different. The three most abundant genera were *Udaeobacter* (13.04%), *Subgroup_2* (9.41%), and *Bradyrhizobium* (6.17%). The three most abundant species were *Udaeobacter* sp. (19.48%), *Rokubacteriales* sp. (13.96%), and *Acidobacteria* sp. (10.12%). The dominant species and relative abundance of fungi differed at the genus and species levels. The three most abundant genara were *Russula* (31.29%), *Mortierella* (19.94%), and *Sebacina* (11.17%). The three most abundant species were *Sebacina* sp. (13.66%), *Russula vesca* (9.43%), and *Mortierella elongata* (9.02%) (Fig. 1). The results of NMDS showed significant differences in the species composition of bacteria and fungi among different communities (all species of bacteria: *P* = 0.038; all species of fungi: *P* = 0.045) (Fig. 2).

### Species specialization characteristics at community level

**Fig. 3:**
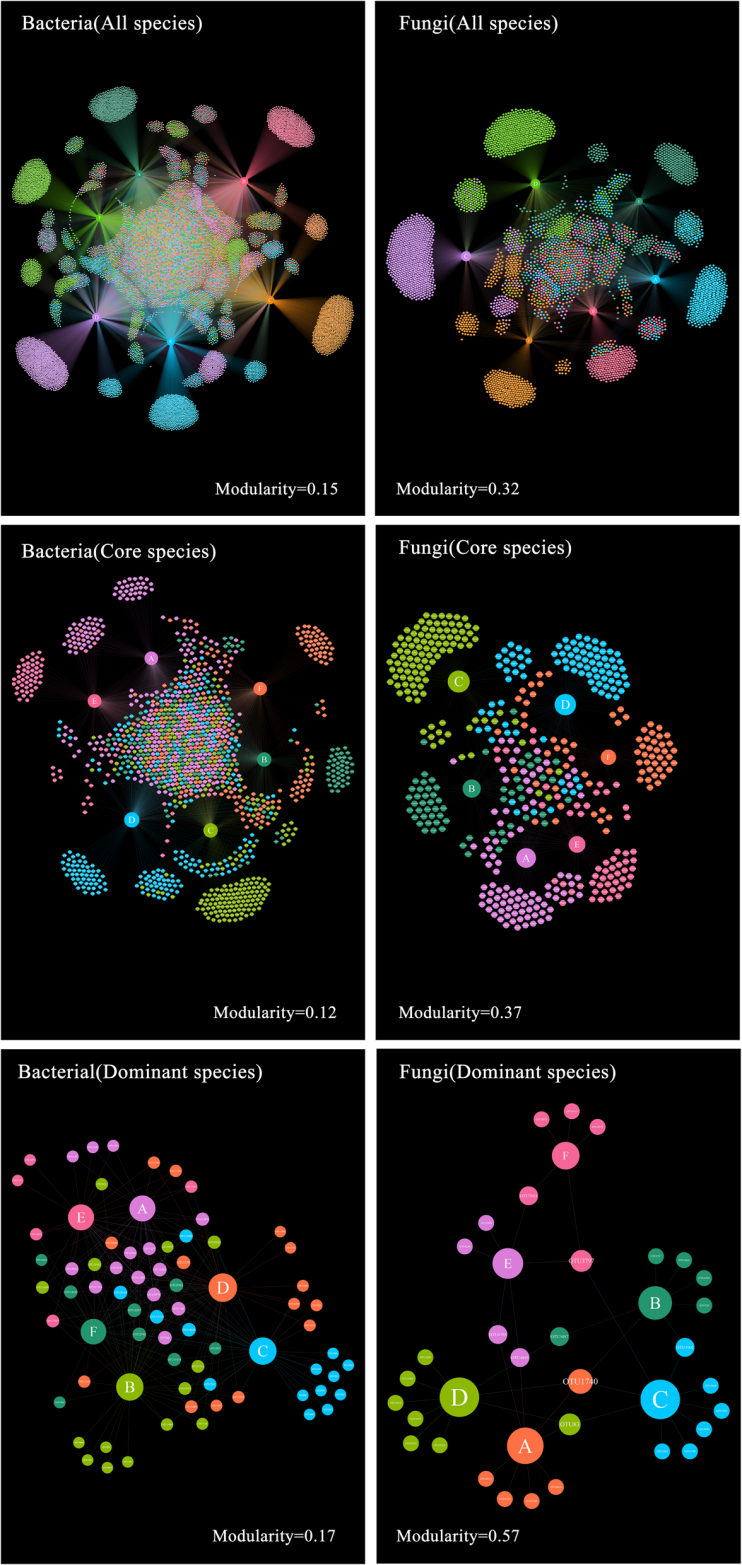
Network analysis of OTU of bacteria and fungi in the six types of communities. The size of the node indicates the richness of the species. The color of the node indicates the distribution of species in different communities. **A**, **B**, **C**, **D**, **E**, and **F** were the *Quercus aliena* var. *acutiserrata* community, *Quercus serrata* var. *brevipetiolata* community, *Larix gmelinii* community, *Pinus armandii* community, *Forsythia suspensa* community, and *Sorbus hupehensis* community, respectively

We detected six interconnected modules with different species compositions of fungi and bacteria among the communities (Fig. 3). The modularity index values were 0.15 for all bacterial species (Core: 0.12; Dominant: 0.17) and 0.32 for all fungal species (Core: 0.37; Dominant: 0.57) (Fig. 3).

### Species specialization characteristics at species level

**Fig. 4:**
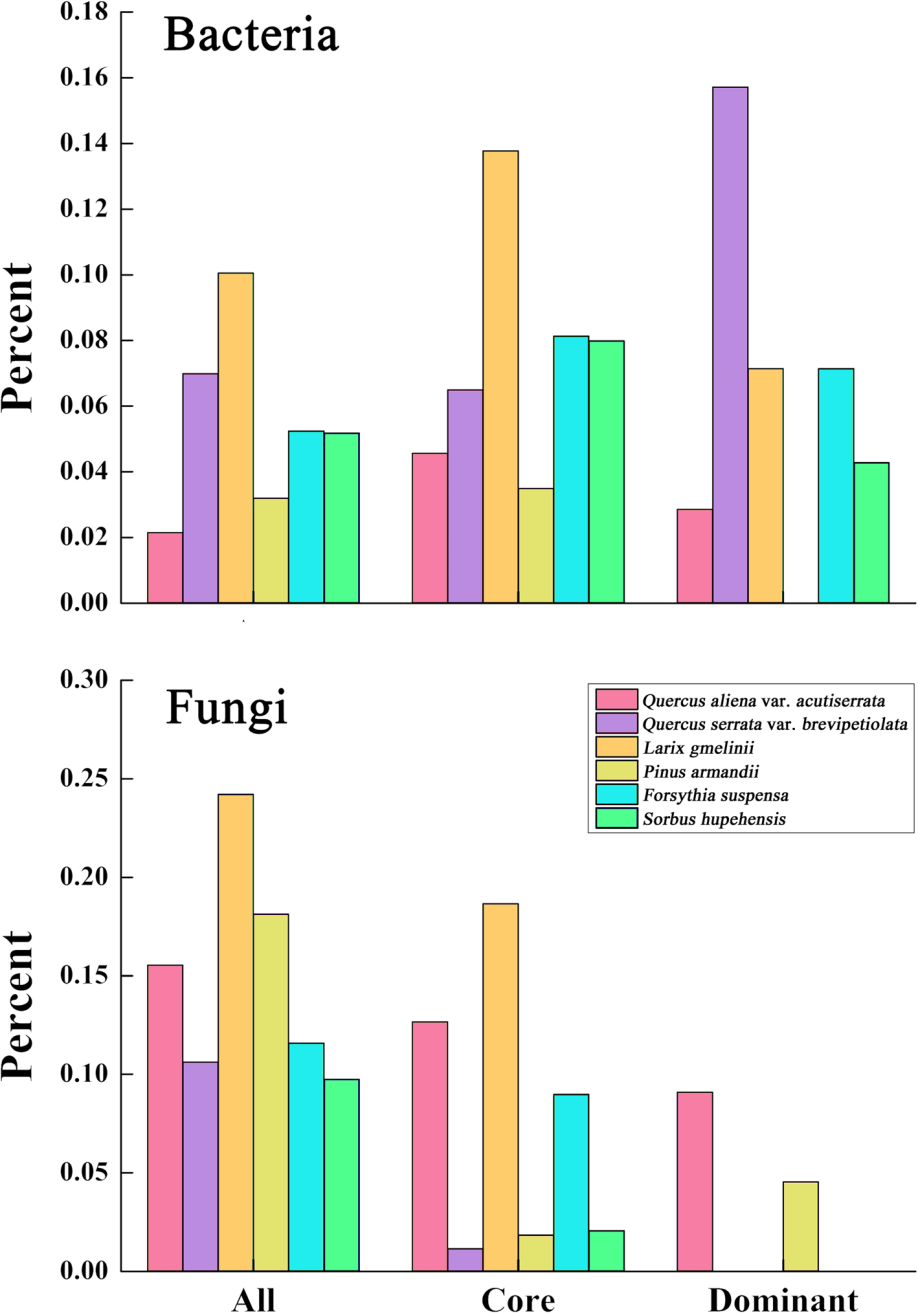
Bar diagrams of bacterial and fungal distribution at the OTU level. The bar diagrams show the percent of bacteria and fungi associated with the six types of communities. Association between microbe and community was tested by torus-translation random test (Torus-translation test, *P* ≤ 0.05 significance level)

Based on torus-translation tests, positive associations with the six habitats were observed among 3811 out of the 11,610 (32.83%) and 3717 out of the 4340 (85.65%) examined bacterial and fungal species, respectively (*P* < 0.05). Most species were positively correlated with LAG community, with 1168 (1168/3811) bacterial species and 1051 (1051/3717) fungal species, accounting for one-third of the total positive correlation number (Fig. 4).

The torus translation showed that 21.17% (3377/15950) of the species tended to be distributed in coniferous forests, while 13.77% (2197/15950) and 13.40% (2137/15950) of the species were distributed in broad-leaved forests and shrub forests, respectively. A total of 2.15%, 6.99%, 10.06%, 3.20%, 5.25%, and 5.19% bacteria OTUs showed positive associations (*P* < 0.05) with QAV, QSV, LAG, PIA, FOS, and SOH communities, respectively. A total of 15.55%, 10.62%, 24.22%, 18.13%, 11.59%, and 9.75% fungi OTUs showed positive associations (*P* < 0.05) with QAV, QSV, LAG, PIA, FOS, and SOH community, respectively. No species was negatively correlated with the communities (Fig. 4). In addition, some species (bacteria: 67.17%; fungi: 14.35%) were found to be neutral to all communities. Tables S2 and S3 show the detailed associations between species and communities.

### Effect of environmental factors on soil microbial communities

**Fig. 5:**
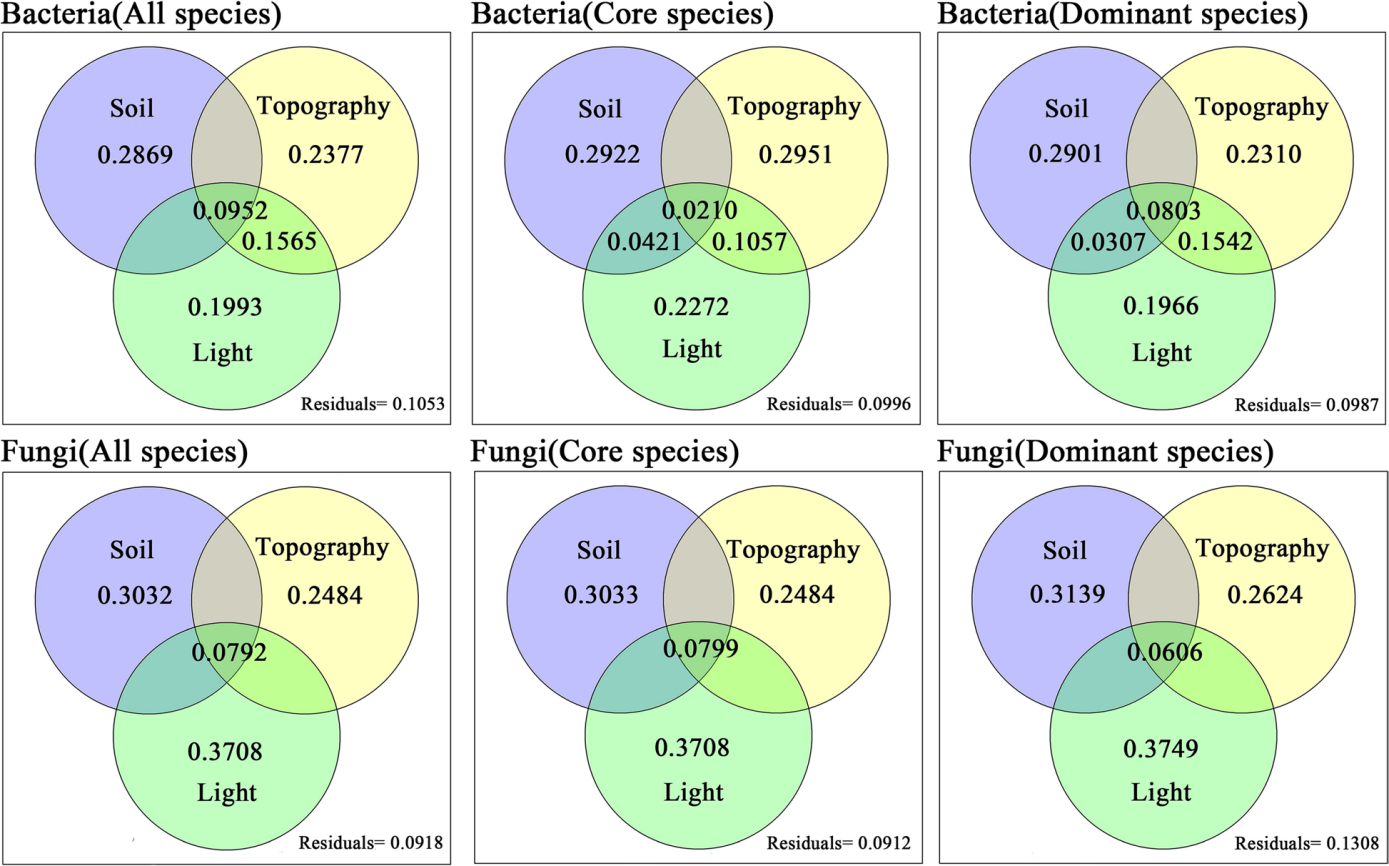
Variance partitioning of the effects of soil, topography, and light on the species abundance of soil bacteria (first row) and soil fungi (second row). Numbers indicate the proportions of explained variation (adjusted R^2^ values). Values less than zero are not shown. Soil pH, soil water content, N, P, and soil organic matter. Topographical factors: elevation, slope, aspect, and convex–concave. Light: light transmittance, scattered radiation, total radiation, canopy cover, leaf area index, and average leaf angle

Variance partitioning analysis showed that all environmental factors (topography, understory light availability, and soil physicochemical properties) explained 89.47% and 90.82% of the overall variation of bacteria and fungi, respectively (Fig. 5). Soil properties (28.69%) explained more variation in bacterial community than light (19.93%) and topography variables (23.77%). Light factors (37.08%) explained more variation in fungal community than topography (24.84%) and soil variables (30.32%) (Fig. 5).

## Discussion

In this study, the distribution pattern of most soil microbes in different types of plant populations is not random but specialized. Different microbial species show different plant population preferences. Fungal species showed higher specialization than bacterial species in different types of plant populations. The main environmental factors driving bacterial and fungal distribution vary among different types of plant populations in this temperate mountain forest. These findings suggest that ecological specialization is important in maintaining local diversity in soil microbial communities at local scales.

### Not random but specialization

The characteristics of bacteria and fungi assemblages differed among different plant population types. The specificity of spatial distribution of soil microbes may be determined by abiotic environmental factors (e.g., topography, understory light availability, and soil physicochemical properties) and biological environmental factors (e.g. plant composition, and community structure) [61, 62]. Each plant population is characterized by particular vegetation communities, soil properties, and soil microbial community structure [63, 64]. In general, changes in the soil microbial community distribution reflect changing interactions between microbes and their environment [65, 66]. Plant communities influence microbial distribution through direct host-microbial interactions and rhizosphere effects [67] and indirect regulation of soil physicochemical properties [68]. Differences in canopy structure and litterfall and plant species composition among different communities affect understory light availability and soil physicochemical properties [25, 69, 70]. Different types of plant populations may also be the reason for the moderate modularity observed in the microbial–community networks (Fig. 3). Therefore, the distribution pattern of most soil microbes in different types of plant populations is not random but specialized in temperate forests.

### Different plant population preferences

NMDS analysis showed that differences in plant populations could affect the community structure of soil microbes. Plant residues are the main source of nutrients for bacteria and fungi, and plant composition varies greatly among different plant communities, leading to significant microbial differences [71–73]. In the torus translation test, 85.65% (3717/4340) of the fungal species and 32.83% (3811/11610) of the bacterial species were associated with specific community types. The torus-translation test also showed that some of the species (bacteria: 67.17%; fungi: 14.35%) examined were not significantly related to any population. The lack of obvious ecological preferences in these species may be due to their wide niche width and high resistance to environmental change. Plant diversity [36], identity [74], and composition [75] also influence microbial distribution in forest ecosystems. Therefore, different microbial species show different plant population preferences in temperate forests.

In recent years, increasing reports have been reported on similar dominant soil bacterial groups in various ecosystems [76–78]. Fierer and Jackson (2006) [79] showed that the species composition of *Acidobacteria*, *Actinobacteria*, *Proteobacteria*, and *Bacteroidetes* dominated the bacterial community, and no significant change in different biologic communities was found. For example, *Acidobacteria*, *Actinobacteria*, and *Proteobacteria* account for more than 75% of the bacterial sequence in the soil of Changbai Mountain in northern China [78]. *Proteobacteria*, *Acidobacteria*, *Actinobacteria* and *Verrucomicrobia* are the dominant bacterial groups in Shennongjia, Southern China [80]. *Proteobacteria*, *Acidobacteria* and *Verrucomicrobia* contributed more than 60% of the soil bacterial sequence in the BCI 50 ha large plot [81]. In this study, *Proteobacteria* (27.89%), *Acidobacteria* (21.22%) and *Verrucomicrobia* (14.39%) accounted for more than 63% of the soil bacteria sequences that could be classified. This implies that *Proteobacteria*, *Acidobacteria* and *Verrucomicrobia* are likely the phylum principally reacting to plant population richness. As for soil fungi, Tedersoo et al. (2014) [82] studied the diversity and geographical distribution pattern of global soil fungi and showed that *Basidiomycota* and *Ascomycota* were the two most abundant groups in all ecosystems, but their relative proportions varied differently in all biotic communities. The soil fungal community of the BCI 50 ha plot was mainly composed of *Basidiomycota* and *Ascomycota*, with a relative abundance of 66% and 27%, respectively [81]. This study also showed that *Ascomycota* and *Basidiomycota* were the dominant groups of soil fungal communities, contributing 60.96% and 27.93%, respectively, accounting for 88% of all taxonomic fungal sequences. Hence, *Ascomycota* and *Basidiomycota* are likely the phylum principally reacting to plant population richness. Therefore, the dominant species of soil bacterial community and soil fungal community in different plant populations are similar, but their relative abundance is significantly different.

Our results showed that soil microbes preferred to be distributed in coniferous forests. Different components of litter among different forest types possibly affect the decomposition rate of litter, leading to differences in soil nutrients and properties, and thus affecting the distribution of microbes [25]. Soil nutrients have an important influence on plant-(above-ground) microbial interactions in forests, which may reinforce microbe-environment associations [13, 14]. Soil properties, especially organic matter and pH, are critical factors that govern the microbial assembly processes in forest ecosystems [3, 83–85]. In the present work, the contents of P, N, pH, and SWC in coniferous forest were higher than those in broad-leaved and shrub forests (Fig. S2). Therefore, coniferous forests may be the preferred habitat for many soil microbes in temperate regions. Our study demonstrates the importance of different types of plant populations in maintaining local diversity in soil microbial communities in temperate forests.

### Fungi are more specialized than bacteria

Consistent with our prediction, fungal species showed higher specialization than bacterial species in different types of plant populations. The modularity index of fungi (32.00%) was higher than that of bacteria (15.00%). In addition, more fungi (85.65%) had specific preferences than bacteria (32.83%) with respect to different types of plant populations. In general, bacteria are more resilient than fungi in the face of environmental changes due to their relatively high intrinsic growth rates and unicellular nature [27]. Fungi are more closely related to plants roots than bacteria [3]. Plants can affect the survival of soil microbes by changing the input of root exudates [86, 87]. Fungi coevolve with host plants, forming mutualistic symbiosis during evolution [3]. Fungal communities are coupled with plant communities in forest ecosystems [3]. The plant–fungi coupling in a forest ecosystem may be due to the direct effects of the plant community through host–fungi specificity [67, 88], indirect effects through input of litter resources [86, 87], or plant-driven changes in soil physicochemical characteristics [68]. For example, abundant ectomycorrhizal trees (e.g., *Larix gmelinii* and *Pinus armandii*) could develop strong biotic interactions with ectomycorrhizal fungi [26]. Soil properties also can determine host–microbe and soil fungal communities interactions [89–91]. AM fungal communities, for example, may be driven more strongly by soil characteristics than host-symbiotic interactions [91]. Some autotrophic bacteria can obtain nutrients through chemosynthesis. Therefore, fungal community show more specialized than bacterial communities in the face of changes in plant population types.

### Main drivers of fungal and bacterial communities are different in different types of plant populations

Ecological specialization is the process by which a species adapts to and persists in its living environment [92, 93]. Ecological specialization depends on species-specific relationships and local and contingent environmental constraints. This study found that main differences in influencing factors between bacterial and fungal communities in different types of plant populations. Light was the main driving factor of the fungal community, and soil physicochemical factors were the main driving factor of bacterial community. Light factors explained more variation in the fungal community than in the bacterial community. It has been reported that soil properties and nutrient content have a great influence on soil fungi [91, 94]. Soil properties and nutrients are important, but light is also the main factor affecting the distribution of soil fungi. Fungi are more sensitive to changes in light than bacteria [95]. Forest canopy is a crucial factor that affects the distribution of soil microbes [96, 97]. Different forest canopy structures will lead to different levels of understory light availability, which will lead to stronger specialization of fungi than bacteria [42]. Therefore, understory light availability may be one of the factors that contribute to the higher specificity of fungal community than bacterial community. The soil physicochemical factors explained more variations in the bacterial community than in the fungal community. As an important part of the soil ecosystem, soil bacteria play an active role in regulating soil nutrient cycling and soil carbon and nitrogen cycling [28]. Soil organic matter affects the survival of soil microbes by affecting soil physicochemical properties and soil matrix composition [61]. Forest litter provides high-quality substrate, which promotes the growth and reproduction of bacteria adapted to high-nutrient environment [61, 98]. The distribution of fungi is more influenced by plant community structure and composition than that of bacteria [99]. In this study, the influence of canopy structure on fungi was greater than that of bacteria. Hence, the main environmental factors driving bacterial and fungal distribution vary among different types of plant populations.

## Conclusion and implications

Our findings are of great significance for understanding how complex soil microbial communities respond to changes in plant populations. Fungal species showed higher specialization than bacterial species in different types of plant populations. Changes in plant population types could have important effects on soil microbial communities by potentially influencing the stability and stress resistance of forest ecosystems. These findings underscore that in sustainable forest management, diverse plant populations should be maintained, since plant populations variability will promote soil microbial diversity. Our study also has its limitations. We did not identify mycorrhizal fungi species among the species detected. However, many fungi show host specificity and are thought to be closely related to plant communities. Therefore, studying the relationship between mycorrhizal fungi and host is the focus of future research.

## Supplementary Information

**Additional file 1: Figure S1.** Sampling sites and plot division. **Figure S2.** Environmental factors of different communities. **Figure S3.** Rarefaction curves of bacteria and fungi. **Table S1.** Dominant species of different communities. **Table S2.** Significant associations of bacteria with different communities based on Torus test. **Table S3.** Significant associations of fungi with different communities based on Torus test. **Table S4.** Abbreviations for bacteria and fungi.

## Data Availability

The raw reads of sequencing data is available at NCBI BioProject SRA database under the accession number PRJNA785719.
