# Supplementary material for: Soil fungal communities show more specificity than bacteria for plant species composition in a temperate forest in China
Source: BMC Microbiol. 2022 Aug 30;22:208. doi: 10.1186/s12866-022-02591-1 (PMC9426227; doi:10.1186/s12866-022-02591-1)
Supplement: Supplementary file 1 — Additional file 1: Figure S1. Sampling sites and plot division. Figure S2. Environmental factors of different communities. Figure S3. Rarefaction curves of bacteria and fungi. Table S1. Dominant species of different communities. Table S2. Significant associations of bacteria with different communities based on Torus test. Table S3. Significant associations of fungi with different communities based on Torus test. Table S4. Abbreviations for bacteria and fungi. [file 12866_2022_2591_MOESM1_ESM.docx]

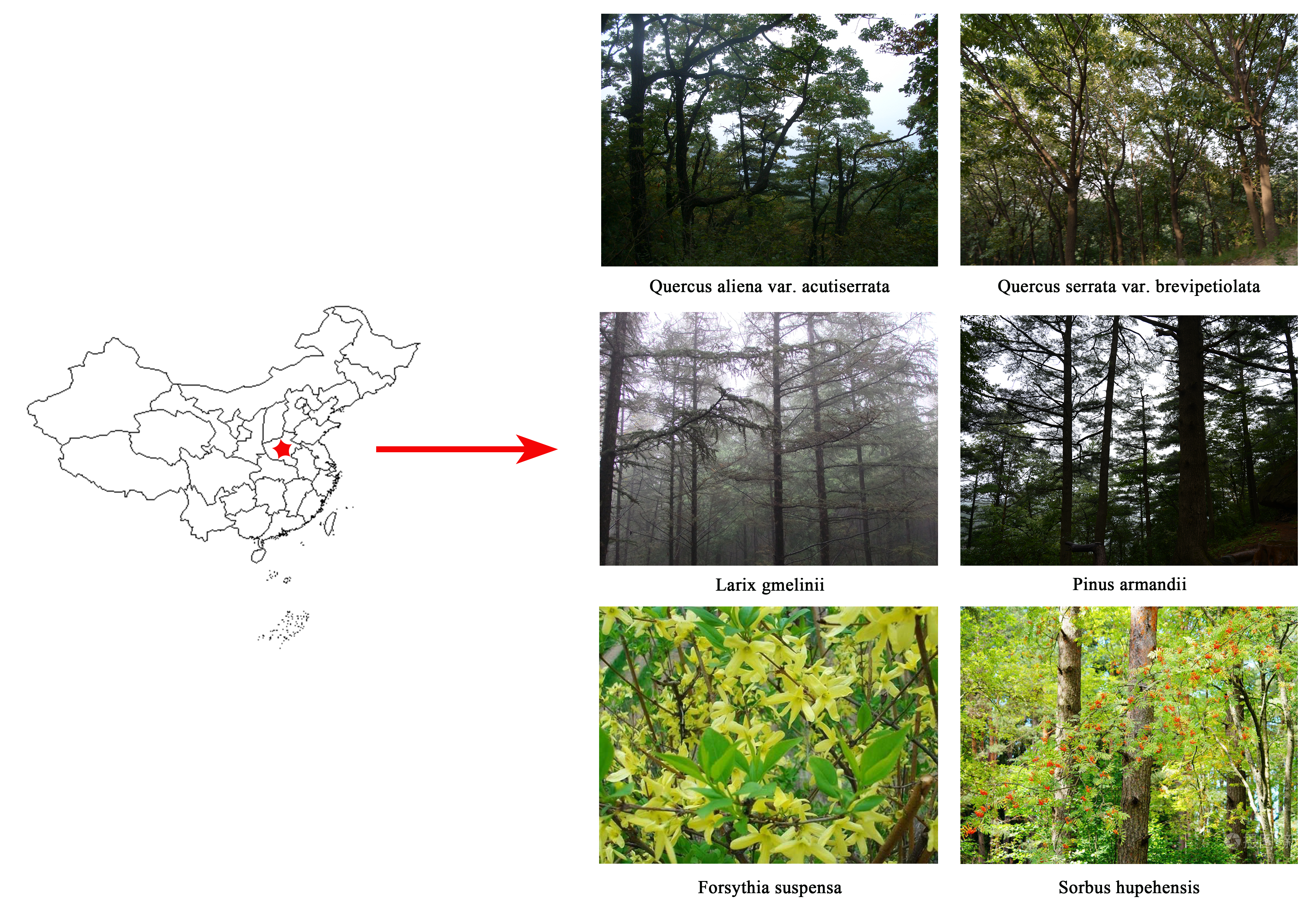


**Figure S1.** Sampling sites and plant community types in this study.


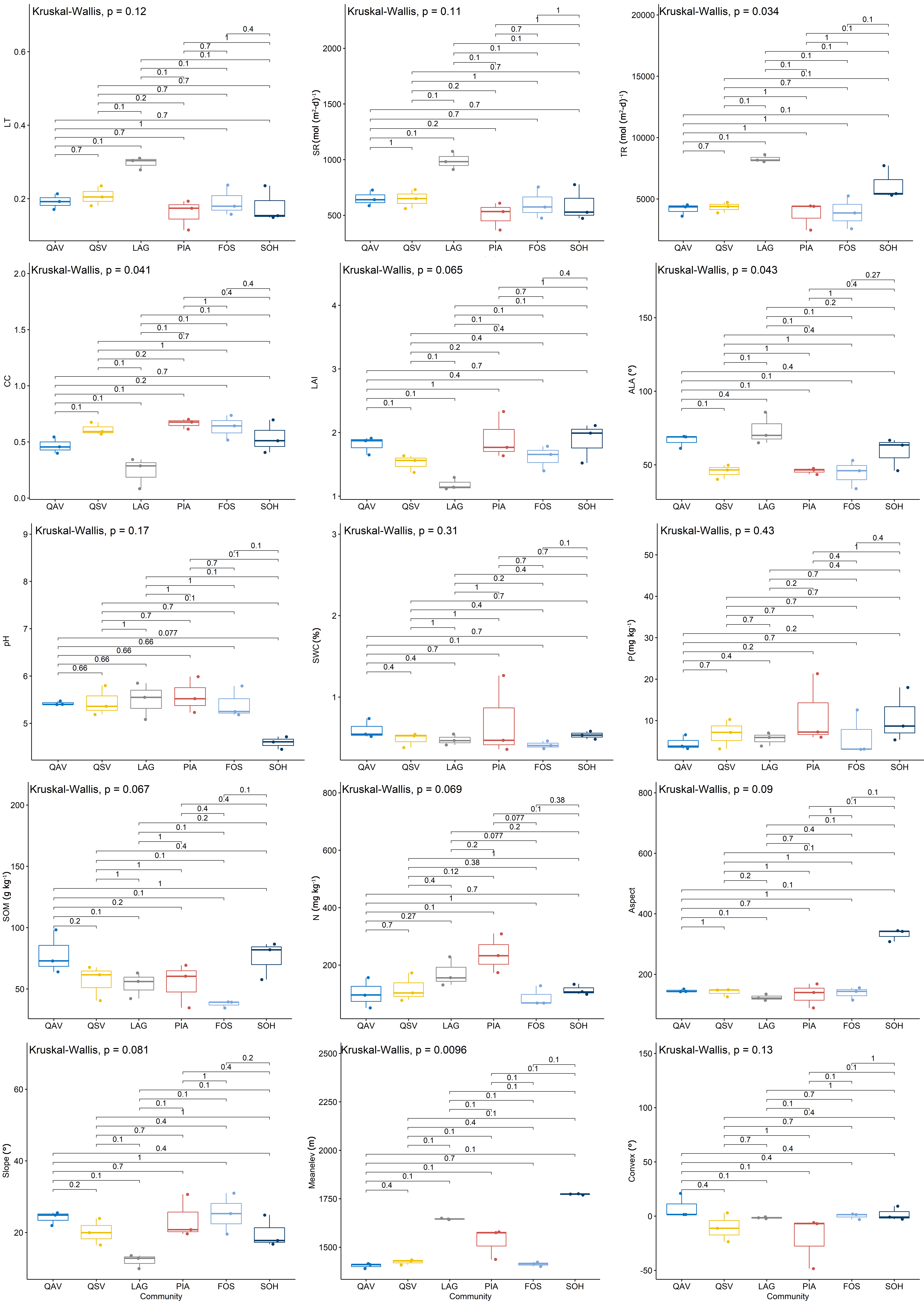


**Figure S2.** Differences in environmental factors among the six types of communities. Black lines indicate significant differences, as obtained using Kruskal–Wallis method (p≤0.05 level of significance). Soil: pH, SWC (soil moisture content), N, P, and SOM (soil organic matter). Topographical factors: meanelevation, slope, aspect, and convex–concave. Light: LT (light transmittance), SR (scattered radiation), TR (total radiation), CC (canopy cover), LAI (leaf area index), and ALA (average leaf angle).


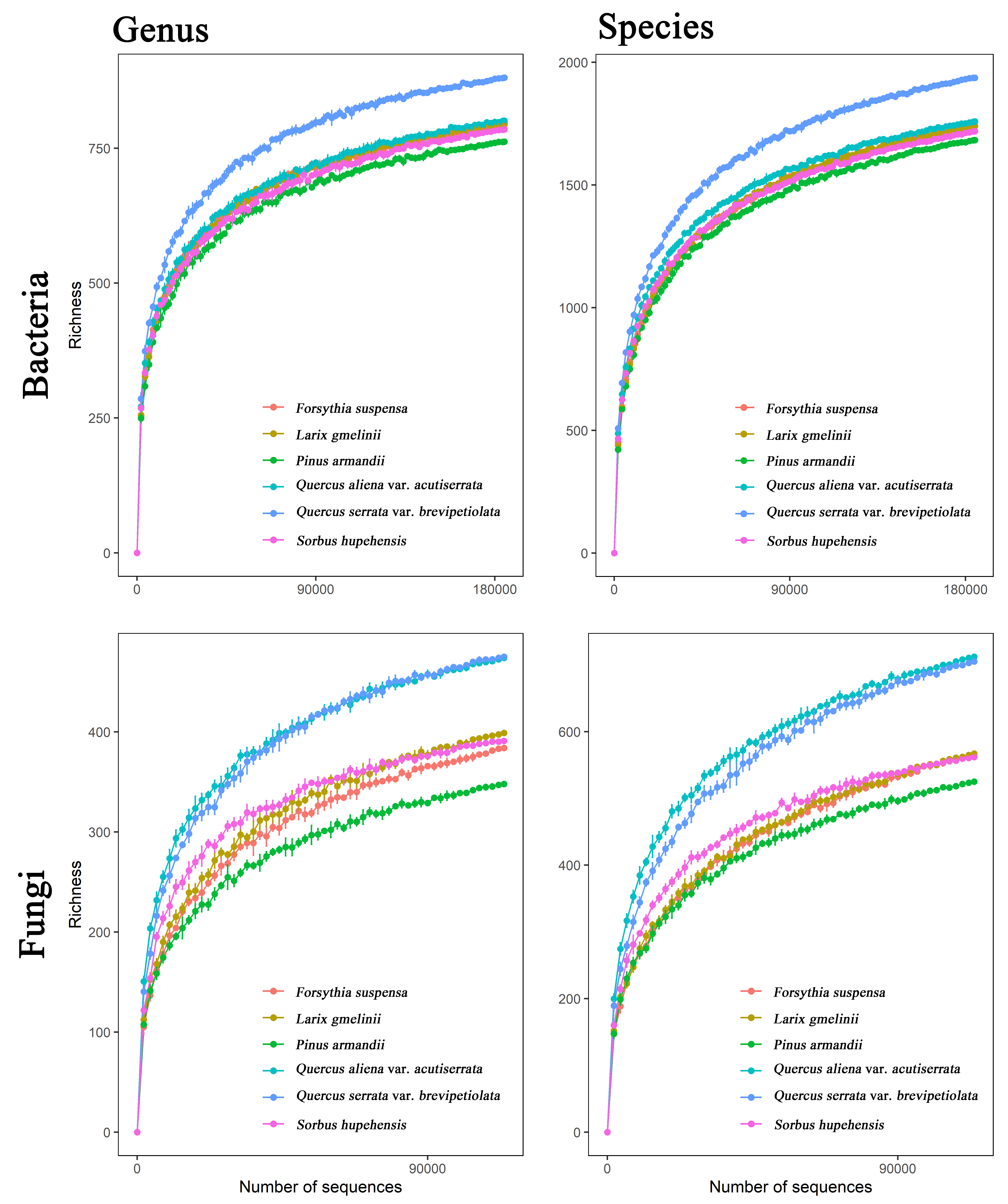


**Figure S3.** Rarefaction curves of the observed genus and species numbers of all the samples from the six types of communities after rarefied.

**Table S1.** Dominant species in the six types of communities. Important value was calculated as follows: important value = (relative abundance (%) + relative frequency (%) + relative breast height sectional area (%))/3.

| Species | Abundance | Mean DBH | Bascal area at breast height (cm^2^) | Importance Value |
| --- | --- | --- | --- | --- |
| ***Quercus aliena* var*. acutiserrata*** |  |  |  |  |
| *Quercus aliena* var*. acutiserrata* | 272.00 | 8.77 | 3.08 | 44.76 |
| *Corylus heterophylla* | 50.00 | 3.58 | 0.11 | 6.22 |
| *Forsythia suspensa* | 53.00 | 2.14 | 0.02 | 5.64 |
| *Cornus kousa* subsp*. chinensis* | 42.00 | 4.62 | 0.08 | 5.50 |
| *Toxicodendron vernicifluum* | 22.00 | 9.17 | 0.19 | 5.27 |
| *Sorbus alnifolia* | 24.00 | 4.11 | 0.05 | 3.37 |
| ***Quercus serrata* var*. brevipetiolata*** |  |  |  |  |
| *Quercus serrata* var*. brevipetiolata* | 256.00 | 8.44 | 2.53 | 33.21 |
| *Quercus aliena* var*. acutiserrata* | 64.00 | 12.59 | 1.02 | 12.75 |
| *Pinus armandii* | 65.00 | 9.46 | 0.52 | 9.41 |
| *Pinus tabuliformis* | 38.00 | 13.38 | 0.66 | 8.91 |
| *Forsythia suspensa* | 106.00 | 1.78 | 0.04 | 8.33 |
| *Sorbus alnifolia* | 29.00 | 3.66 | 0.06 | 4.36 |
| ***Larix gmelinii*** |  |  |  |  |
| *Larix gmelinii* | 51.00 | 25.96 | 2.91 | 70.52 |
| *Toxicodendron vernicifluum* | 5.00 | 17.48 | 0.18 | 8.71 |
| *Betula chinensis* | 3.00 | 6.60 | 0.01 | 5.87 |
| *Litsea tsinlingensis* | 2.00 | 5.95 | 0.01 | 5.29 |
| *Betula platyphylla* | 1.00 | 13.00 | 0.01 | 4.84 |
| *Lindera obtusiloba Blume* | 1.00 | 9.70 | 0.01 | 4.77 |
| ***Pinus armandii*** |  |  |  |  |
| *Pinus armandii* | 96.00 | 12.15 | 1.39 | 24.88 |
| *Quercus aliena* var*. acutiserrata* | 60.00 | 13.23 | 1.30 | 20.40 |
| *Symplocos paniculata* | 16.00 | 2.04 | 0.01 | 4.70 |
| *Sorbus alnifolia* | 26.00 | 3.72 | 0.05 | 4.57 |
| *Toxicodendron vernicifluum* | 7.00 | 14.50 | 0.13 | 4.20 |
| *Pinus tabuliformis* | 11.00 | 13.48 | 0.17 | 4.16 |
| ***Forsythia suspensa*** |  |  |  |  |
| *Forsythia suspensa* | 333.00 | 2.44 | 0.32 | 19.55 |
| *Quercus aliena* var*. acutiserrata* | 105.00 | 12.66 | 1.63 | 19.23 |
| *Pinus tabuliformis* | 56.00 | 13.26 | 0.95 | 11.82 |
| *Pinus armandii* | 51.00 | 11.70 | 0.63 | 9.15 |
| *Lindera obtusiloba Blume* | 32.00 | 4.43 | 0.07 | 3.93 |
| *Quercus serrata* var*. brevipetiolata* | 22.00 | 8.72 | 0.18 | 3.72 |
| ***Sorbus hupehensis*** |  |  |  |  |
| *Sorbus hupehensis* | 338.00 | 2.73 | 0.34 | 31.43 |
| *Quercus aliena* var*. acutiserrata* | 32.00 | 18.48 | 1.08 | 20.76 |
| *Salix chaenomeloides Kimura* | 27.00 | 11.49 | 0.36 | 9.67 |
| *Litsea tsinlingensis* | 36.00 | 4.29 | 0.07 | 7.15 |
| *Pinus armandii* | 5.00 | 12.94 | 0.13 | 4.79 |
| *Toxicodendron vernicifluum* | 6.00 | 17.30 | 0.18 | 4.25 |

**Table S2.** Significant associations of soil bacteria with the six types of communities (P ≤ 0.05 level of significance for torus-translation test). QAV, QSV, LAG, PIA, FOS, and SOH represent *Quercus aliena* var. *acutiserrata*, *Quercus serrata* var. *brevipetiolata*, *Larix gmelinii*, *Pinus armandii*, *Forsythia suspensa*, and *Sorbus hupehensis*, respectively. NA represents no significant correlation. (+) indicates positive correlation.

| Species | Community | | | | | |
| --- | --- | --- | --- | --- | --- | --- |
|  | QAV | QSV | LAG | PIA | FOS | SOH |
| OTU270 | NA | NA | + | NA | NA | NA |
| OTU6325 | NA | NA | NA | + | NA | NA |
| OTU4608 | NA | + | + | NA | NA | NA |
| OTU1342 | NA | NA | NA | NA | NA | + |
| OTU4605 | NA | + | NA | + | NA | NA |
| OTU2468 | + | NA | NA | + | NA | NA |
| OTU4279 | NA | NA | NA | NA | NA | NA |
| OTU122 | NA | NA | NA | NA | + | NA |
| OTU6917 | NA | NA | NA | NA | NA | NA |
| OTU6740 | NA | NA | NA | NA | NA | + |
| OTU236 | NA | NA | NA | + | NA | NA |
| OTU7142 | NA | NA | + | NA | NA | NA |
| OTU2909 | NA | NA | + | NA | NA | NA |
| OTU3108 | NA | NA | NA | + | NA | NA |
| OTU1567 | NA | NA | NA | NA | + | NA |
| OTU4365 | NA | NA | NA | + | NA | NA |
| OTU4590 | NA | NA | NA | + | NA | NA |
| OTU5173 | NA | NA | NA | NA | + | NA |
| OTU4340 | NA | NA | + | NA | NA | NA |
| OTU7671 | NA | NA | NA | NA | NA | NA |
| OTU1177 | + | NA | NA | NA | NA | NA |
| OTU4507 | + | NA | NA | NA | NA | NA |
| OTU5485 | + | NA | NA | NA | NA | NA |
| OTU5114 | NA | NA | NA | + | NA | NA |
| OTU6896 | NA | NA | NA | + | NA | NA |
| OTU6593 | NA | NA | NA | + | NA | NA |
| OTU3193 | NA | NA | NA | NA | + | NA |
| OTU6911 | NA | NA | NA | + | NA | NA |
| OTU2533 | + | NA | NA | NA | NA | NA |
| OTU6433 | NA | NA | NA | + | NA | NA |
| OTU5728 | NA | + | NA | NA | NA | NA |
| OTU1966 | NA | NA | NA | NA | NA | + |
| OTU2561 | NA | NA | NA | NA | NA | NA |
| OTU3927 | NA | NA | NA | NA | + | NA |
| OTU7652 | NA | NA | NA | NA | NA | NA |
| OTU3400 | NA | NA | NA | NA | NA | NA |
| OTU3403 | NA | NA | NA | + | NA | NA |
| OTU2428 | NA | + | NA | + | NA | NA |
| OTU3154 | NA | NA | NA | NA | NA | NA |
| OTU2373 | NA | NA | + | NA | NA | NA |
| OTU871 | NA | NA | + | NA | NA | NA |
| OTU5213 | NA | NA | NA | NA | + | NA |
| OTU5584 | + | NA | NA | NA | NA | NA |
| OTU5564 | NA | NA | NA | + | NA | NA |
| OTU911 | NA | NA | + | NA | NA | NA |
| OTU7340 | NA | + | NA | NA | NA | NA |
| OTU4254 | NA | NA | NA | NA | NA | NA |
| OTU1176 | NA | + | NA | NA | NA | NA |
| OTU1832 | NA | NA | NA | NA | NA | NA |
| OTU2059 | NA | NA | NA | + | NA | NA |
| OTU1831 | + | NA | NA | NA | NA | NA |
| OTU377 | NA | + | NA | NA | NA | NA |
| OTU2157 | NA | NA | NA | NA | + | NA |
| OTU380 | NA | NA | NA | NA | NA | NA |
| OTU8260 | NA | NA | + | NA | NA | NA |
| OTU5251 | NA | NA | NA | NA | + | NA |
| OTU824 | NA | NA | NA | NA | NA | NA |
| OTU674 | NA | NA | + | NA | NA | NA |
| OTU1963 | NA | NA | + | NA | NA | NA |
| OTU3970 | NA | NA | NA | + | NA | NA |
| OTU742 | NA | NA | NA | + | NA | NA |
| OTU269 | NA | NA | NA | + | NA | + |
| OTU2589 | + | NA | NA | NA | NA | NA |
| OTU4451 | + | NA | NA | NA | NA | NA |
| OTU4458 | + | NA | NA | NA | + | NA |
| OTU7587 | NA | NA | NA | NA | NA | + |
| OTU7566 | NA | NA | NA | NA | NA | NA |
| OTU2269 | NA | NA | + | NA | NA | NA |
| OTU3585 | NA | NA | NA | NA | NA | NA |
| OTU3042 | NA | NA | NA | NA | NA | NA |
| OTU3355 | NA | NA | NA | + | NA | NA |
| OTU633 | NA | NA | + | NA | NA | NA |
| OTU3076 | NA | NA | NA | + | NA | NA |
| OTU8221 | NA | NA | + | NA | NA | NA |
| OTU1442 | NA | NA | + | NA | NA | NA |
| OTU3389 | NA | NA | NA | + | NA | NA |
| OTU7720 | NA | NA | NA | NA | NA | + |
| OTU7231 | NA | NA | + | NA | NA | NA |
| OTU7236 | NA | + | NA | NA | + | NA |
| OTU4743 | NA | NA | NA | NA | + | NA |
| OTU700 | NA | NA | NA | + | NA | NA |
| OTU7084 | NA | NA | + | NA | NA | NA |
| OTU3864 | NA | NA | NA | NA | + | NA |
| OTU549 | NA | NA | + | NA | NA | NA |
| OTU5150 | + | NA | NA | NA | NA | NA |
| OTU4729 | NA | NA | NA | NA | + | NA |
| OTU5906 | NA | NA | NA | NA | + | NA |
| OTU6598 | NA | NA | NA | NA | NA | NA |
| OTU727 | NA | NA | + | NA | NA | NA |
| OTU4387 | + | NA | NA | + | NA | NA |
| OTU5685 | NA | NA | NA | + | NA | + |
| OTU288 | NA | NA | + | NA | NA | NA |
| OTU2144 | NA | NA | NA | NA | + | NA |
| OTU4838 | + | NA | NA | NA | NA | NA |
| OTU506 | NA | NA | + | NA | NA | NA |
| OTU4978 | NA | NA | NA | NA | + | NA |
| OTU1098 | + | NA | + | NA | NA | NA |
| OTU5162 | + | NA | NA | NA | NA | NA |
| OTU1805 | + | NA | NA | NA | NA | NA |
| OTU2952 | NA | NA | + | NA | NA | NA |
| OTU3509 | NA | NA | + | NA | NA | NA |
| OTU6921 | NA | NA | NA | + | NA | NA |
| OTU5043 | + | NA | NA | NA | NA | NA |
| OTU616 | NA | + | NA | NA | NA | NA |
| OTU6212 | NA | NA | + | NA | NA | NA |
| OTU2609 | NA | + | + | NA | NA | NA |
| OTU6068 | NA | + | NA | NA | NA | NA |
| OTU1528 | NA | NA | NA | NA | NA | NA |
| OTU1355 | NA | NA | NA | NA | NA | NA |
| OTU5875 | NA | NA | NA | NA | NA | + |
| OTU1353 | NA | NA | + | NA | NA | NA |
| OTU6772 | NA | NA | NA | NA | NA | + |
| OTU7658 | NA | + | NA | NA | NA | NA |
| OTU4187 | NA | + | NA | NA | NA | NA |
| OTU3777 | + | NA | NA | NA | NA | NA |
| OTU6113 | NA | NA | + | NA | NA | NA |
| OTU4101 | NA | NA | NA | NA | NA | + |
| OTU3676 | NA | NA | + | NA | NA | NA |
| OTU4369 | NA | NA | NA | NA | NA | + |
| OTU3070 | NA | NA | NA | NA | NA | + |
| OTU3222 | NA | NA | + | NA | NA | NA |
| OTU3221 | NA | NA | NA | + | NA | NA |
| OTU8006 | NA | NA | NA | NA | NA | NA |
| OTU2567 | NA | NA | NA | NA | + | NA |
| OTU5863 | NA | NA | NA | + | NA | + |
| OTU1639 | NA | NA | NA | NA | NA | + |
| OTU1149 | NA | + | NA | NA | NA | NA |
| OTU3022 | NA | + | NA | NA | NA | NA |
| OTU1632 | NA | + | NA | NA | NA | NA |
| OTU378 | NA | NA | + | NA | NA | NA |
| OTU1939 | NA | NA | + | NA | NA | NA |
| OTU5053 | + | NA | NA | NA | NA | NA |
| OTU4741 | NA | NA | NA | NA | + | NA |
| OTU1616 | + | NA | NA | NA | NA | NA |
| OTU5605 | NA | NA | NA | NA | NA | NA |
| OTU5490 | + | NA | NA | NA | NA | NA |
| OTU223 | NA | NA | + | NA | NA | NA |
| OTU6918 | NA | NA | NA | NA | NA | + |
| OTU4523 | + | NA | NA | NA | NA | NA |
| OTU6998 | NA | NA | NA | NA | NA | NA |
| OTU7991 | + | NA | NA | NA | NA | NA |
| OTU6651 | NA | NA | NA | NA | NA | NA |
| OTU3425 | NA | NA | NA | + | NA | NA |
| OTU1897 | NA | + | NA | NA | NA | NA |
| OTU2595 | NA | + | NA | NA | NA | NA |
| OTU2599 | NA | + | NA | NA | NA | NA |
| OTU1200 | NA | NA | NA | NA | NA | NA |
| OTU5110 | + | NA | NA | NA | NA | NA |
| OTU1169 | NA | NA | + | NA | NA | NA |
| OTU2315 | NA | NA | NA | NA | + | NA |
| OTU7066 | NA | NA | + | NA | NA | NA |
| OTU6627 | NA | NA | + | NA | NA | NA |
| OTU4848 | + | NA | NA | NA | NA | NA |
| OTU7036 | NA | NA | + | NA | NA | NA |
| OTU643 | NA | NA | + | NA | NA | NA |
| OTU257 | NA | NA | NA | + | NA | NA |
| OTU3364 | NA | NA | NA | + | NA | NA |
| OTU3368 | NA | NA | NA | + | NA | NA |
| OTU142 | NA | NA | + | NA | NA | NA |
| OTU550 | NA | NA | + | NA | NA | NA |
| OTU5709 | + | NA | NA | NA | NA | NA |
| OTU1463 | NA | NA | NA | + | NA | NA |
| OTU2201 | NA | NA | NA | + | NA | NA |
| OTU3460 | NA | NA | NA | + | NA | NA |
| OTU5532 | NA | NA | NA | NA | + | NA |
| OTU350 | NA | NA | NA | NA | NA | NA |
| OTU4733 | NA | + | NA | NA | NA | NA |
| OTU5466 | NA | NA | NA | NA | NA | NA |
| OTU5467 | + | NA | NA | NA | NA | NA |
| OTU3636 | NA | NA | NA | NA | + | NA |
| OTU5495 | + | NA | NA | NA | NA | NA |
| OTU3754 | NA | + | NA | NA | NA | NA |
| OTU276 | NA | NA | + | NA | NA | NA |
| OTU3408 | NA | NA | NA | + | NA | NA |
| OTU3034 | NA | NA | + | NA | NA | NA |
| OTU5470 | + | NA | NA | NA | NA | NA |
| OTU8219 | NA | NA | + | NA | NA | NA |
| OTU2548 | + | NA | NA | NA | NA | NA |
| OTU5393 | NA | NA | + | NA | NA | NA |
| OTU5826 | NA | NA | NA | NA | NA | NA |
| OTU4376 | NA | NA | NA | + | NA | NA |
| OTU4669 | NA | NA | + | NA | NA | NA |
| OTU5166 | NA | NA | NA | NA | + | NA |
| OTU6755 | NA | NA | + | NA | NA | NA |
| OTU230 | NA | + | NA | NA | NA | NA |
| OTU7598 | NA | NA | NA | NA | NA | NA |
| OTU5228 | NA | NA | NA | NA | + | NA |
| OTU1465 | NA | NA | + | NA | NA | NA |
| OTU546 | NA | NA | + | NA | NA | NA |
| OTU7391 | NA | NA | NA | + | NA | NA |
| OTU4282 | NA | + | NA | NA | NA | NA |
| OTU6126 | NA | NA | + | NA | NA | NA |
| OTU4521 | + | NA | NA | NA | NA | NA |
| OTU2894 | NA | NA | + | NA | NA | NA |
| OTU5070 | NA | + | NA | NA | NA | NA |
| OTU3287 | NA | NA | + | NA | NA | NA |
| OTU7707 | NA | NA | NA | NA | NA | + |
| OTU5305 | NA | NA | NA | NA | NA | NA |
| OTU4470 | NA | NA | NA | NA | NA | NA |
| OTU1262 | NA | NA | NA | NA | NA | NA |
| OTU2431 | NA | NA | NA | + | NA | NA |
| OTU4476 | NA | NA | NA | NA | NA | + |
| OTU1705 | + | NA | NA | NA | NA | NA |
| OTU7636 | NA | NA | + | NA | NA | NA |
| OTU2624 | NA | NA | NA | + | NA | NA |
| OTU6311 | + | NA | NA | NA | + | NA |
| OTU4433 | NA | + | NA | NA | NA | NA |
| OTU7010 | NA | NA | NA | + | NA | NA |
| OTU1600 | NA | NA | NA | NA | NA | NA |
| OTU7015 | NA | NA | + | NA | NA | NA |
| OTU6022 | NA | NA | NA | + | NA | NA |
| OTU4290 | NA | NA | NA | + | NA | NA |
| OTU3058 | NA | NA | NA | + | NA | NA |
| OTU717 | NA | NA | NA | NA | NA | NA |
| OTU3381 | NA | NA | NA | + | NA | NA |
| OTU4739 | + | NA | NA | NA | NA | NA |
| OTU5348 | NA | NA | + | NA | NA | NA |
| OTU3176 | NA | NA | NA | + | NA | NA |
| OTU7611 | NA | NA | NA | + | NA | NA |
| OTU4790 | + | NA | NA | NA | NA | NA |
| OTU827 | + | NA | NA | NA | NA | NA |
| OTU7089 | NA | NA | + | NA | NA | NA |
| OTU6054 | NA | NA | NA | + | NA | NA |
| OTU5824 | NA | + | NA | + | NA | + |
| OTU4326 | NA | NA | NA | NA | NA | NA |
| OTU4398 | + | NA | NA | NA | NA | NA |
| OTU893 | NA | NA | NA | NA | NA | NA |
| OTU7122 | NA | NA | NA | NA | NA | + |
| OTU4474 | NA | NA | NA | NA | NA | NA |
| OTU4039 | + | NA | NA | NA | NA | NA |
| OTU5970 | NA | NA | NA | NA | NA | NA |
| OTU7223 | NA | NA | + | NA | NA | NA |
| OTU5245 | NA | NA | NA | NA | + | NA |
| OTU7103 | NA | NA | + | NA | NA | NA |
| OTU1115 | NA | NA | NA | + | NA | NA |
| OTU7008 | NA | NA | NA | NA | NA | + |
| OTU5725 | NA | + | NA | NA | NA | NA |
| OTU4471 | + | NA | NA | NA | NA | NA |
| OTU672 | NA | NA | NA | NA | NA | NA |
| OTU1428 | NA | + | NA | NA | NA | NA |
| OTU4203 | NA | + | NA | NA | NA | NA |
| OTU2659 | NA | NA | NA | + | NA | NA |
| OTU7876 | NA | NA | + | NA | NA | NA |
| OTU8192 | NA | NA | NA | NA | NA | + |
| OTU4172 | NA | + | NA | NA | NA | NA |
| OTU4418 | NA | NA | NA | NA | NA | NA |
| OTU722 | NA | NA | NA | NA | NA | NA |
| OTU4255 | NA | NA | NA | + | NA | NA |
| OTU3407 | NA | NA | NA | + | NA | NA |
| OTU6912 | NA | NA | NA | + | NA | NA |
| OTU5483 | + | NA | NA | NA | NA | NA |
| OTU4472 | + | NA | NA | NA | NA | NA |
| OTU249 | NA | NA | NA | NA | NA | NA |
| OTU1462 | NA | NA | NA | + | NA | NA |
| OTU5707 | + | NA | NA | NA | NA | NA |
| OTU2482 | NA | NA | NA | + | NA | NA |
| OTU154 | NA | + | NA | NA | NA | NA |
| OTU5522 | NA | NA | NA | NA | + | NA |
| OTU6391 | NA | NA | NA | NA | NA | + |
| OTU6396 | NA | NA | NA | NA | NA | + |
| OTU6395 | NA | NA | NA | NA | NA | NA |
| OTU4508 | NA | NA | NA | NA | NA | NA |
| OTU5152 | NA | + | NA | NA | NA | NA |
| OTU4501 | + | NA | NA | NA | NA | NA |
| OTU7095 | NA | NA | + | NA | NA | NA |
| OTU5458 | NA | NA | NA | + | NA | NA |
| OTU3467 | NA | NA | NA | + | NA | NA |
| OTU1686 | NA | NA | + | NA | NA | NA |
| OTU5167 | + | NA | NA | NA | NA | NA |
| OTU6647 | NA | + | NA | NA | NA | NA |
| OTU4862 | + | NA | NA | NA | NA | NA |
| OTU4860 | + | NA | NA | NA | NA | NA |
| OTU4865 | + | NA | NA | NA | NA | NA |
| OTU4868 | + | NA | NA | NA | NA | NA |
| OTU77 | NA | NA | NA | NA | NA | NA |
| OTU3928 | NA | NA | NA | NA | NA | NA |
| OTU3612 | + | NA | NA | NA | NA | NA |
| OTU3617 | NA | + | NA | NA | NA | NA |
| OTU6312 | NA | NA | + | NA | NA | NA |
| OTU4280 | NA | + | NA | NA | NA | NA |
| OTU5272 | NA | NA | NA | NA | + | NA |
| OTU5903 | NA | NA | NA | NA | NA | + |
| OTU4637 | + | NA | NA | NA | NA | NA |
| OTU2939 | NA | NA | NA | NA | NA | NA |
| OTU2938 | NA | NA | NA | NA | + | NA |
| OTU7590 | NA | NA | NA | NA | + | NA |
| OTU5295 | NA | NA | NA | NA | NA | NA |
| OTU5194 | + | NA | NA | NA | NA | NA |
| OTU7208 | NA | NA | NA | NA | NA | NA |
| OTU6108 | NA | NA | NA | NA | NA | NA |
| OTU4454 | + | NA | NA | NA | NA | NA |
| OTU626 | NA | NA | + | NA | NA | NA |
| OTU3406 | NA | NA | NA | + | NA | NA |
| OTU1656 | NA | NA | NA | NA | NA | NA |
| OTU4646 | NA | NA | NA | NA | + | NA |
| OTU4135 | NA | NA | + | NA | NA | NA |
| OTU7654 | NA | NA | + | NA | NA | NA |
| OTU2427 | NA | NA | + | NA | NA | NA |
| OTU275 | NA | NA | + | NA | NA | NA |
| OTU3820 | NA | NA | + | NA | NA | NA |
| OTU250 | NA | + | NA | NA | NA | NA |
| OTU4024 | NA | NA | NA | + | NA | NA |
| OTU5831 | NA | + | NA | NA | NA | NA |
| OTU3554 | NA | NA | NA | + | + | NA |
| OTU5492 | + | NA | NA | NA | NA | NA |
| OTU49 | NA | NA | NA | NA | + | NA |
| OTU7080 | NA | NA | + | NA | NA | NA |
| OTU3081 | NA | NA | NA | NA | NA | NA |
| OTU346 | NA | NA | + | NA | NA | NA |
| OTU7258 | NA | NA | + | NA | NA | NA |
| OTU7111 | NA | NA | + | NA | NA | NA |
| OTU1679 | NA | NA | + | NA | NA | NA |
| OTU1601 | NA | NA | NA | NA | + | NA |
| OTU1315 | NA | NA | NA | + | NA | NA |
| OTU3535 | NA | NA | NA | NA | NA | + |
| OTU4396 | NA | NA | NA | + | NA | NA |
| OTU1481 | NA | NA | NA | + | NA | NA |
| OTU173 | NA | NA | NA | NA | NA | + |
| OTU959 | NA | NA | + | NA | NA | NA |
| OTU135 | NA | NA | NA | + | NA | NA |
| OTU6550 | NA | NA | NA | NA | NA | NA |
| OTU6097 | NA | NA | + | NA | NA | NA |
| OTU4020 | NA | NA | NA | NA | + | NA |
| OTU8229 | NA | NA | + | NA | NA | NA |
| OTU8228 | NA | NA | NA | NA | NA | NA |
| OTU1737 | NA | NA | NA | NA | NA | NA |
| OTU7928 | + | NA | NA | NA | NA | NA |
| OTU1052 | NA | NA | + | NA | NA | NA |
| OTU8226 | NA | NA | NA | + | NA | NA |
| OTU4759 | NA | NA | NA | NA | + | NA |
| OTU5434 | NA | NA | NA | NA | + | NA |
| OTU7870 | + | NA | NA | NA | NA | NA |
| OTU3114 | NA | NA | + | NA | NA | NA |
| OTU3118 | NA | NA | NA | + | NA | NA |
| OTU3463 | NA | NA | NA | + | NA | NA |
| OTU2016 | + | NA | NA | NA | NA | NA |
| OTU3553 | NA | NA | NA | NA | NA | + |
| OTU6898 | NA | NA | NA | NA | NA | NA |
| OTU800 | NA | NA | NA | NA | NA | + |
| OTU7829 | NA | NA | NA | NA | NA | NA |
| OTU6250 | NA | NA | NA | NA | + | NA |
| OTU2394 | NA | NA | NA | NA | + | NA |
| OTU2395 | NA | NA | NA | NA | + | NA |
| OTU3379 | NA | NA | NA | NA | NA | NA |
| OTU5514 | NA | NA | + | NA | NA | NA |
| OTU1870 | NA | NA | + | NA | NA | NA |
| OTU1871 | NA | NA | + | NA | NA | NA |
| OTU5326 | NA | NA | NA | NA | NA | + |
| OTU5413 | NA | NA | NA | NA | + | NA |
| OTU2762 | NA | NA | NA | NA | + | NA |
| OTU163 | NA | NA | + | NA | NA | NA |
| OTU734 | NA | NA | + | NA | NA | NA |
| OTU5973 | NA | NA | NA | NA | NA | + |
| OTU2015 | + | NA | NA | NA | NA | NA |
| OTU317 | NA | NA | + | NA | NA | NA |
| OTU3007 | NA | NA | + | NA | NA | NA |
| OTU129 | + | NA | NA | NA | NA | NA |
| OTU4180 | NA | + | NA | NA | NA | NA |
| OTU679 | NA | NA | + | NA | NA | NA |
| OTU4577 | NA | NA | NA | NA | + | NA |
| OTU7477 | NA | NA | NA | NA | NA | + |
| OTU3801 | NA | NA | + | NA | NA | NA |
| OTU7856 | + | NA | NA | NA | NA | NA |
| OTU4981 | NA | NA | NA | NA | + | NA |
| OTU4625 | NA | + | NA | NA | NA | NA |
| OTU609 | NA | NA | + | NA | NA | NA |
| OTU4866 | NA | NA | + | NA | NA | NA |
| OTU4475 | NA | NA | NA | NA | NA | NA |
| OTU3972 | NA | NA | NA | NA | NA | + |
| OTU3478 | NA | NA | NA | + | NA | NA |
| OTU1407 | NA | NA | NA | NA | + | NA |
| OTU4159 | NA | + | NA | NA | NA | NA |
| OTU6055 | NA | NA | + | NA | NA | NA |
| OTU6910 | NA | NA | NA | + | NA | NA |
| OTU296 | NA | NA | + | NA | NA | NA |
| OTU3166 | NA | + | NA | NA | NA | NA |
| OTU958 | NA | NA | + | NA | NA | NA |
| OTU858 | NA | + | NA | NA | NA | NA |
| OTU3028 | NA | NA | + | NA | NA | NA |
| OTU2410 | NA | NA | + | NA | NA | NA |
| OTU1437 | NA | NA | + | NA | NA | NA |
| OTU3991 | NA | NA | NA | NA | NA | NA |
| OTU6332 | + | NA | NA | NA | NA | NA |
| OTU5882 | NA | NA | NA | NA | NA | + |
| OTU2042 | NA | NA | NA | NA | NA | + |
| OTU1113 | NA | NA | NA | NA | NA | NA |
| OTU5848 | + | NA | NA | NA | NA | NA |
| OTU337 | NA | NA | + | NA | NA | NA |
| OTU5233 | NA | NA | NA | NA | + | NA |
| OTU2704 | NA | NA | NA | NA | + | NA |
| OTU3186 | NA | NA | NA | NA | NA | NA |
| OTU7039 | NA | NA | + | NA | NA | NA |
| OTU4559 | NA | NA | NA | NA | NA | NA |
| OTU5161 | NA | NA | NA | NA | + | NA |
| OTU5160 | + | NA | NA | NA | NA | NA |
| OTU6004 | NA | NA | + | NA | NA | NA |
| OTU4065 | NA | NA | + | NA | NA | NA |
| OTU7556 | + | NA | NA | NA | NA | NA |
| OTU1623 | NA | NA | + | NA | NA | NA |
| OTU1152 | NA | NA | + | NA | NA | NA |
| OTU2727 | NA | NA | NA | NA | NA | NA |
| OTU984 | NA | NA | + | NA | NA | NA |
| OTU6802 | NA | NA | NA | NA | NA | NA |
| OTU4505 | + | NA | NA | NA | NA | NA |
| OTU7481 | NA | NA | NA | NA | NA | NA |
| OTU302 | NA | NA | + | NA | NA | NA |
| OTU6244 | NA | NA | NA | + | NA | NA |
| OTU1313 | NA | NA | + | NA | NA | NA |
| OTU4633 | NA | + | NA | NA | NA | NA |
| OTU5729 | NA | + | NA | NA | NA | NA |
| OTU592 | NA | NA | + | NA | NA | NA |
| OTU3832 | NA | NA | NA | NA | NA | NA |
| OTU1816 | NA | NA | NA | NA | NA | NA |
| OTU2312 | NA | NA | NA | NA | NA | NA |
| OTU7232 | NA | + | NA | NA | NA | NA |
| OTU3607 | + | NA | NA | NA | NA | NA |
| OTU2901 | NA | NA | NA | + | NA | NA |
| OTU2987 | NA | NA | + | NA | NA | NA |
| OTU2985 | NA | NA | + | NA | NA | NA |
| OTU2989 | NA | NA | + | NA | NA | NA |
| OTU3847 | + | NA | NA | NA | NA | NA |
| OTU6098 | NA | NA | + | NA | NA | NA |
| OTU7618 | NA | NA | NA | NA | NA | + |
| OTU1938 | + | NA | + | NA | NA | NA |
| OTU3396 | NA | NA | NA | + | NA | NA |
| OTU3895 | NA | NA | NA | NA | NA | NA |
| OTU3363 | NA | NA | NA | + | NA | NA |
| OTU7137 | NA | NA | + | NA | NA | NA |
| OTU7136 | NA | NA | + | NA | NA | NA |
| OTU2973 | NA | NA | + | NA | NA | NA |
| OTU3017 | NA | NA | + | NA | NA | NA |
| OTU5533 | + | NA | NA | NA | NA | NA |
| OTU3386 | NA | NA | NA | + | NA | NA |
| OTU1678 | NA | NA | + | NA | NA | NA |
| OTU3079 | NA | NA | NA | NA | NA | NA |
| OTU3746 | NA | NA | NA | NA | NA | NA |
| OTU209 | + | NA | + | NA | NA | NA |
| OTU3790 | NA | NA | NA | NA | NA | + |
| OTU2532 | NA | NA | NA | NA | NA | NA |
| OTU7712 | NA | NA | + | NA | NA | NA |
| OTU4921 | NA | NA | NA | NA | NA | NA |
| OTU3623 | + | NA | NA | NA | NA | NA |
| OTU4569 | NA | NA | NA | NA | + | NA |
| OTU2723 | NA | NA | NA | NA | NA | NA |
| OTU2399 | NA | + | NA | NA | NA | NA |
| OTU3691 | NA | NA | NA | NA | NA | NA |
| OTU4682 | NA | NA | NA | NA | NA | NA |
| OTU5124 | + | NA | NA | NA | NA | NA |
| OTU6666 | NA | NA | NA | NA | NA | + |
| OTU2967 | NA | NA | + | NA | NA | NA |
| OTU5861 | NA | NA | NA | + | NA | + |
| OTU4804 | + | NA | NA | NA | + | NA |
| OTU875 | NA | + | NA | NA | NA | NA |
| OTU4808 | NA | NA | NA | NA | + | NA |
| OTU4809 | NA | NA | NA | NA | + | NA |
| OTU3328 | NA | NA | NA | + | NA | NA |
| OTU3329 | NA | NA | NA | + | NA | NA |
| OTU2611 | + | NA | NA | NA | NA | NA |
| OTU4982 | + | NA | NA | NA | NA | NA |
| OTU6397 | NA | NA | NA | NA | NA | + |
| OTU7169 | NA | NA | + | NA | NA | NA |
| OTU7167 | NA | NA | + | NA | NA | NA |
| OTU560 | NA | NA | + | NA | NA | NA |
| OTU4423 | + | NA | NA | NA | NA | NA |
| OTU2237 | NA | NA | NA | + | NA | NA |
| OTU5575 | NA | + | NA | NA | NA | NA |
| OTU4544 | NA | NA | NA | NA | + | NA |
| OTU5329 | NA | NA | + | NA | NA | NA |
| OTU4296 | NA | NA | NA | + | NA | NA |
| OTU1505 | + | NA | NA | NA | NA | NA |
| OTU7381 | NA | NA | NA | NA | NA | + |
| OTU1227 | NA | NA | NA | + | NA | NA |
| OTU7123 | NA | NA | NA | NA | NA | NA |
| OTU521 | NA | NA | + | NA | NA | NA |
| OTU4598 | NA | NA | NA | NA | NA | NA |
| OTU4677 | + | NA | NA | NA | NA | NA |
| OTU2101 | + | NA | NA | NA | NA | NA |
| OTU6240 | NA | + | NA | NA | NA | NA |
| OTU143 | NA | NA | NA | NA | NA | + |
| OTU4597 | NA | NA | + | NA | NA | NA |
| OTU544 | NA | + | NA | NA | NA | NA |
| OTU2889 | + | NA | NA | NA | NA | NA |
| OTU3596 | NA | NA | + | NA | NA | NA |
| OTU4481 | + | NA | NA | NA | + | NA |
| OTU445 | NA | NA | NA | NA | NA | NA |
| OTU2204 | NA | NA | + | NA | NA | + |
| OTU3456 | NA | NA | NA | + | NA | NA |
| OTU7261 | NA | NA | + | NA | NA | NA |
| OTU7868 | NA | NA | NA | + | NA | NA |
| OTU2408 | NA | NA | + | NA | NA | NA |
| OTU6383 | NA | NA | NA | + | NA | NA |
| OTU7861 | NA | NA | NA | NA | NA | + |
| OTU588 | NA | NA | + | NA | NA | NA |
| OTU277 | NA | NA | + | NA | NA | NA |
| OTU278 | NA | + | NA | NA | NA | NA |
| OTU5481 | + | NA | NA | NA | NA | NA |
| OTU7244 | NA | NA | NA | NA | NA | NA |
| OTU7241 | NA | + | NA | NA | NA | NA |
| OTU2432 | NA | NA | NA | NA | NA | + |
| OTU4922 | + | NA | NA | NA | NA | NA |
| OTU3090 | NA | NA | NA | + | NA | NA |
| OTU3395 | NA | NA | NA | + | NA | NA |
| OTU3251 | NA | NA | + | NA | NA | NA |
| OTU6632 | NA | NA | NA | NA | NA | + |
| OTU3387 | NA | NA | NA | + | NA | NA |
| OTU1688 | NA | NA | NA | + | NA | NA |
| OTU4361 | NA | NA | NA | NA | NA | NA |
| OTU669 | NA | NA | + | NA | NA | NA |
| OTU3204 | NA | NA | + | NA | NA | NA |
| OTU300 | NA | NA | + | NA | NA | NA |
| OTU4414 | + | NA | NA | NA | NA | NA |
| OTU3791 | NA | NA | NA | + | NA | NA |
| OTU2322 | NA | NA | NA | + | NA | NA |
| OTU4511 | + | NA | NA | NA | NA | NA |
| OTU3572 | NA | NA | NA | + | + | NA |
| OTU5959 | NA | NA | NA | NA | + | NA |
| OTU1674 | NA | NA | NA | NA | NA | NA |
| OTU3271 | NA | NA | NA | NA | NA | + |
| OTU1687 | NA | NA | + | NA | + | NA |
| OTU4773 | NA | + | NA | NA | NA | NA |
| OTU4771 | + | NA | NA | NA | + | NA |
| OTU4959 | + | NA | NA | NA | NA | NA |
| OTU4774 | NA | NA | NA | NA | + | NA |
| OTU1374 | NA | + | NA | + | NA | NA |
| OTU6565 | NA | NA | NA | + | NA | NA |
| OTU3190 | NA | NA | + | NA | NA | NA |
| OTU5264 | NA | NA | NA | NA | NA | NA |
| OTU4500 | + | NA | NA | NA | NA | NA |
| OTU4355 | + | NA | NA | NA | NA | NA |
| OTU5454 | + | NA | NA | NA | NA | NA |
| OTU1645 | NA | NA | NA | + | NA | NA |
| OTU5463 | NA | NA | + | NA | NA | NA |
| OTU4561 | NA | NA | NA | NA | + | NA |
| OTU4567 | NA | NA | NA | NA | + | NA |
| OTU8015 | NA | + | NA | NA | NA | NA |
| OTU5327 | NA | NA | NA | + | NA | NA |
| OTU1731 | + | + | NA | NA | NA | NA |
| OTU4568 | NA | NA | NA | NA | + | NA |
| OTU7250 | NA | NA | NA | NA | NA | + |
| OTU5654 | + | NA | NA | NA | NA | NA |
| OTU8204 | NA | NA | NA | NA | NA | NA |
| OTU5652 | NA | NA | NA | NA | NA | NA |
| OTU2591 | + | NA | NA | NA | NA | + |
| OTU5268 | NA | NA | NA | NA | + | NA |
| OTU4460 | + | NA | NA | NA | NA | NA |
| OTU6888 | NA | NA | NA | NA | NA | + |
| OTU5580 | NA | + | NA | NA | NA | NA |
| OTU7718 | NA | NA | NA | NA | + | NA |
| OTU2218 | NA | + | NA | NA | NA | NA |
| OTU3409 | NA | NA | NA | + | NA | NA |
| OTU8223 | NA | NA | + | NA | NA | NA |
| OTU7812 | NA | NA | NA | + | NA | NA |
| OTU3907 | NA | NA | NA | + | NA | NA |
| OTU1598 | + | NA | NA | NA | NA | NA |
| OTU4337 | NA | NA | NA | + | NA | NA |
| OTU7152 | NA | NA | + | NA | NA | NA |
| OTU7838 | NA | + | NA | NA | NA | NA |
| OTU6699 | NA | NA | NA | NA | NA | NA |
| OTU2236 | NA | NA | NA | + | NA | NA |
| OTU3718 | NA | NA | NA | NA | NA | + |
| OTU2435 | + | NA | NA | NA | NA | NA |
| OTU4615 | NA | NA | NA | + | NA | NA |
| OTU1247 | NA | NA | + | NA | NA | NA |
| OTU1794 | NA | NA | + | NA | NA | NA |
| OTU2263 | NA | + | NA | NA | NA | NA |
| OTU481 | NA | NA | NA | NA | NA | + |
| OTU4136 | + | NA | NA | NA | NA | NA |
| OTU251 | NA | + | NA | NA | NA | NA |
| OTU4622 | NA | + | NA | NA | NA | NA |
| OTU2765 | NA | NA | NA | + | NA | NA |
| OTU5422 | NA | NA | NA | + | NA | NA |
| OTU4557 | + | NA | NA | NA | NA | NA |
| OTU7993 | NA | NA | NA | NA | + | NA |
| OTU5021 | NA | NA | NA | NA | + | NA |
| OTU110 | NA | NA | NA | + | NA | NA |
| OTU3481 | NA | NA | NA | NA | NA | NA |
| OTU2907 | + | NA | NA | NA | NA | NA |
| OTU6469 | NA | NA | + | NA | NA | NA |
| OTU5645 | + | NA | NA | NA | NA | NA |
| OTU6752 | NA | + | NA | NA | NA | NA |
| OTU192 | NA | + | NA | NA | NA | NA |
| OTU4230 | NA | NA | + | NA | NA | NA |
| OTU6689 | NA | NA | NA | NA | NA | + |
| OTU580 | NA | NA | + | NA | NA | NA |
| OTU6205 | NA | NA | NA | NA | NA | + |
| OTU1599 | NA | + | NA | NA | NA | NA |
| OTU4822 | NA | NA | NA | NA | NA | NA |
| OTU4823 | NA | NA | NA | NA | + | NA |
| OTU4239 | NA | + | NA | NA | NA | NA |
| OTU4446 | + | NA | NA | NA | NA | NA |
| OTU1611 | + | NA | NA | NA | NA | NA |
| OTU8043 | NA | NA | NA | NA | NA | + |
| OTU4308 | NA | NA | NA | + | NA | NA |
| OTU4824 | NA | NA | NA | + | + | NA |
| OTU602 | NA | NA | + | NA | NA | NA |
| OTU4820 | NA | NA | NA | + | NA | NA |
| OTU1932 | NA | NA | NA | NA | + | NA |
| OTU1936 | NA | NA | NA | + | NA | NA |
| OTU222 | NA | NA | + | NA | NA | NA |
| OTU1934 | + | NA | NA | NA | NA | NA |
| OTU1663 | NA | NA | NA | + | NA | NA |
| OTU3784 | NA | NA | NA | + | NA | NA |
| OTU3477 | NA | NA | NA | + | NA | NA |
| OTU1910 | NA | NA | + | NA | NA | NA |
| OTU35 | NA | NA | + | NA | NA | NA |
| OTU4594 | NA | NA | NA | NA | + | NA |
| OTU7723 | NA | NA | NA | + | NA | NA |
| OTU100 | + | NA | NA | NA | NA | NA |
| OTU3850 | NA | NA | + | NA | NA | NA |
| OTU6142 | NA | NA | NA | NA | NA | NA |
| OTU8081 | + | NA | NA | NA | NA | NA |
| OTU8084 | NA | NA | NA | NA | NA | + |
| OTU2911 | NA | NA | NA | + | NA | NA |
| OTU589 | NA | NA | + | NA | NA | NA |
| OTU5884 | NA | NA | NA | NA | NA | + |
| OTU952 | NA | NA | + | NA | NA | NA |
| OTU5880 | NA | NA | NA | NA | NA | + |
| OTU7843 | NA | NA | + | NA | NA | NA |
| OTU3903 | NA | NA | NA | NA | NA | NA |
| OTU4173 | NA | NA | NA | + | NA | NA |
| OTU1582 | NA | NA | NA | NA | NA | NA |
| OTU4524 | NA | NA | NA | NA | NA | NA |
| OTU4029 | + | NA | NA | NA | + | NA |
| OTU4455 | + | NA | NA | NA | + | NA |
| OTU6820 | + | NA | NA | NA | NA | NA |
| OTU5261 | NA | NA | NA | NA | + | NA |
| OTU3487 | NA | NA | NA | + | NA | NA |
| OTU1292 | NA | NA | + | NA | NA | NA |
| OTU8026 | NA | NA | NA | NA | NA | + |
| OTU1911 | NA | NA | + | NA | NA | NA |
| OTU297 | NA | NA | + | NA | NA | + |
| OTU5455 | NA | NA | NA | + | NA | NA |
| OTU3303 | NA | NA | NA | + | NA | NA |
| OTU5593 | NA | NA | NA | NA | + | NA |
| OTU7333 | NA | NA | + | NA | NA | NA |
| OTU2206 | NA | NA | NA | NA | NA | NA |
| OTU3675 | NA | NA | NA | + | NA | NA |
| OTU4571 | NA | NA | NA | NA | NA | NA |
| OTU394 | NA | NA | + | NA | NA | NA |
| OTU1273 | NA | NA | NA | NA | NA | + |
| OTU3744 | NA | NA | + | NA | NA | NA |
| OTU7158 | NA | NA | + | NA | NA | NA |
| OTU1755 | NA | NA | + | NA | NA | NA |
| OTU7155 | + | NA | NA | NA | NA | NA |
| OTU7154 | NA | NA | + | NA | NA | NA |
| OTU2465 | NA | NA | NA | NA | + | NA |
| OTU6248 | NA | + | NA | NA | NA | NA |
| OTU3819 | NA | NA | NA | NA | NA | NA |
| OTU3990 | NA | NA | NA | + | NA | NA |
| OTU1651 | NA | NA | + | NA | NA | NA |
| OTU4137 | NA | NA | NA | NA | + | NA |
| OTU6983 | NA | NA | NA | NA | NA | + |
| OTU2545 | NA | NA | NA | + | NA | NA |
| OTU4963 | + | NA | NA | NA | NA | NA |
| OTU7924 | + | NA | NA | NA | NA | NA |
| OTU3664 | + | NA | NA | NA | NA | NA |
| OTU6228 | NA | NA | NA | NA | + | NA |
| OTU5104 | NA | NA | NA | NA | NA | NA |
| OTU8093 | NA | NA | NA | NA | NA | + |
| OTU973 | NA | NA | + | NA | NA | NA |
| OTU3798 | NA | NA | NA | NA | NA | NA |
| OTU2753 | NA | + | NA | + | NA | NA |
| OTU4936 | NA | NA | NA | NA | + | NA |
| OTU5866 | NA | NA | NA | NA | NA | + |
| OTU7987 | NA | NA | NA | NA | NA | + |
| OTU7984 | NA | NA | NA | NA | NA | NA |
| OTU4447 | + | NA | NA | NA | NA | NA |
| OTU3285 | NA | NA | NA | + | NA | NA |
| OTU1105 | + | NA | + | NA | NA | NA |
| OTU6591 | + | NA | NA | NA | NA | NA |
| OTU2669 | NA | NA | NA | + | NA | NA |
| OTU6858 | NA | NA | NA | NA | NA | + |
| OTU5947 | NA | NA | + | NA | NA | NA |
| OTU6851 | NA | NA | NA | NA | NA | + |
| OTU2492 | NA | + | NA | NA | NA | NA |
| OTU2858 | NA | NA | + | NA | NA | NA |
| OTU5239 | + | NA | NA | NA | NA | NA |
| OTU5227 | NA | NA | NA | NA | + | NA |
| OTU5225 | + | NA | NA | NA | NA | NA |
| OTU5559 | NA | NA | NA | NA | NA | + |
| OTU5229 | NA | + | NA | NA | + | NA |
| OTU4405 | + | NA | NA | NA | NA | NA |
| OTU6859 | + | NA | NA | NA | NA | NA |
| OTU2962 | NA | NA | + | NA | NA | NA |
| OTU1602 | NA | NA | NA | NA | NA | + |
| OTU5431 | NA | NA | NA | NA | + | NA |
| OTU2594 | + | NA | NA | NA | NA | NA |
| OTU3244 | NA | NA | NA | NA | NA | NA |
| OTU5262 | NA | NA | NA | NA | NA | NA |
| OTU7159 | NA | NA | + | NA | NA | NA |
| OTU2002 | NA | NA | NA | + | NA | NA |
| OTU3182 | NA | NA | + | NA | NA | NA |
| OTU7308 | NA | NA | NA | + | NA | NA |
| OTU5606 | NA | NA | NA | NA | + | NA |
| OTU3849 | NA | NA | NA | + | NA | NA |
| OTU4258 | NA | NA | NA | + | NA | NA |
| OTU1220 | NA | NA | NA | + | NA | NA |
| OTU307 | NA | NA | NA | + | NA | NA |
| OTU3009 | NA | NA | + | NA | NA | NA |
| OTU6696 | NA | NA | NA | + | NA | + |
| OTU8063 | NA | NA | NA | NA | NA | NA |
| OTU4213 | NA | + | NA | NA | NA | NA |
| OTU8044 | + | NA | NA | NA | NA | NA |
| OTU6144 | NA | NA | NA | NA | NA | NA |
| OTU2168 | NA | NA | NA | NA | + | NA |
| OTU5941 | + | NA | NA | NA | NA | NA |
| OTU3258 | NA | NA | NA | NA | NA | NA |
| OTU3191 | NA | NA | NA | + | NA | NA |
| OTU2685 | + | NA | NA | + | NA | NA |
| OTU6963 | NA | NA | NA | + | NA | NA |
| OTU6573 | NA | NA | NA | NA | NA | + |
| OTU2052 | NA | + | NA | NA | NA | NA |
| OTU7764 | NA | NA | NA | + | NA | NA |
| OTU3785 | NA | NA | NA | + | NA | NA |
| OTU6562 | NA | NA | NA | NA | NA | + |
| OTU1330 | NA | NA | + | NA | NA | NA |
| OTU2050 | NA | NA | NA | NA | NA | NA |
| OTU6502 | + | NA | NA | NA | NA | NA |
| OTU1970 | + | NA | NA | NA | NA | NA |
| OTU4318 | + | NA | NA | NA | NA | NA |
| OTU1626 | NA | NA | + | NA | NA | NA |
| OTU4643 | NA | NA | NA | NA | NA | NA |
| OTU5672 | + | NA | NA | NA | NA | NA |
| OTU1557 | + | NA | NA | NA | NA | NA |
| OTU1655 | NA | + | NA | NA | NA | NA |
| OTU3393 | NA | NA | NA | + | NA | NA |
| OTU124 | + | NA | + | NA | NA | NA |
| OTU2783 | NA | + | NA | NA | NA | NA |
| OTU3589 | NA | + | NA | NA | NA | NA |
| OTU7573 | NA | NA | NA | + | NA | NA |
| OTU760 | NA | NA | NA | NA | NA | NA |
| OTU768 | NA | NA | + | NA | NA | NA |
| OTU503 | + | + | NA | NA | NA | NA |
| OTU5760 | + | NA | NA | NA | NA | NA |
| OTU6195 | NA | + | NA | NA | NA | NA |
| OTU42 | NA | NA | NA | + | NA | NA |
| OTU3208 | + | NA | NA | NA | NA | NA |
| OTU4540 | NA | NA | NA | NA | + | NA |
| OTU334 | NA | NA | + | NA | NA | NA |
| OTU738 | + | NA | NA | NA | NA | NA |
| OTU656 | NA | NA | + | NA | NA | NA |
| OTU4256 | NA | NA | NA | + | NA | NA |
| OTU3856 | NA | NA | + | NA | NA | NA |
| OTU3347 | NA | NA | NA | NA | + | NA |
| OTU3401 | + | NA | NA | NA | NA | NA |
| OTU7188 | NA | NA | NA | NA | + | NA |
| OTU2238 | NA | + | NA | NA | NA | NA |
| OTU2233 | + | NA | NA | NA | NA | NA |
| OTU4229 | NA | + | NA | NA | NA | NA |
| OTU2460 | NA | NA | NA | NA | NA | NA |
| OTU3672 | NA | NA | NA | + | NA | NA |
| OTU4367 | NA | NA | NA | NA | + | NA |
| OTU2205 | NA | + | NA | NA | NA | NA |
| OTU447 | NA | NA | + | NA | NA | NA |
| OTU3281 | NA | NA | + | NA | NA | NA |
| OTU8273 | NA | NA | NA | NA | NA | NA |
| OTU3514 | NA | NA | NA | NA | NA | NA |
| OTU7034 | NA | NA | + | NA | NA | NA |
| OTU1872 | NA | NA | NA | NA | NA | NA |
| OTU7173 | NA | NA | + | NA | NA | NA |
| OTU7175 | NA | NA | NA | NA | NA | NA |
| OTU7177 | NA | NA | + | NA | NA | NA |
| OTU7178 | NA | NA | NA | NA | NA | NA |
| OTU2759 | NA | NA | NA | NA | NA | NA |
| OTU7190 | NA | + | NA | NA | NA | NA |
| OTU5517 | + | NA | NA | NA | NA | NA |
| OTU5920 | NA | NA | NA | NA | NA | NA |
| OTU1168 | NA | NA | + | NA | NA | + |
| OTU3349 | NA | NA | NA | + | NA | NA |
| OTU5646 | NA | + | NA | NA | NA | NA |
| OTU5857 | NA | NA | NA | NA | NA | + |
| OTU6334 | NA | NA | NA | + | NA | NA |
| OTU179 | NA | NA | + | NA | NA | NA |
| OTU3402 | NA | NA | NA | + | NA | NA |
| OTU4186 | NA | + | NA | NA | NA | NA |
| OTU7756 | NA | NA | NA | NA | NA | + |
| OTU4445 | + | NA | NA | NA | NA | NA |
| OTU3995 | + | NA | NA | NA | NA | NA |
| OTU4796 | NA | NA | NA | NA | + | NA |
| OTU4599 | + | NA | NA | NA | NA | NA |
| OTU3602 | NA | NA | NA | NA | NA | NA |
| OTU892 | NA | NA | NA | NA | NA | NA |
| OTU5202 | + | NA | NA | NA | NA | NA |
| OTU4851 | + | NA | NA | NA | NA | NA |
| OTU1875 | NA | NA | NA | NA | NA | NA |
| OTU4373 | + | NA | NA | NA | + | NA |
| OTU4309 | NA | NA | NA | + | NA | NA |
| OTU4377 | NA | NA | NA | NA | + | NA |
| OTU7182 | NA | NA | + | NA | NA | NA |
| OTU511 | NA | NA | NA | NA | NA | NA |
| OTU4734 | + | NA | NA | NA | NA | NA |
| OTU7783 | NA | + | NA | NA | NA | NA |
| OTU725 | NA | + | NA | NA | NA | NA |
| OTU7794 | NA | NA | NA | NA | NA | + |
| OTU7791 | NA | NA | NA | + | NA | NA |
| OTU4425 | + | NA | NA | NA | NA | NA |
| OTU4157 | NA | + | NA | NA | NA | NA |
| OTU1064 | NA | NA | + | NA | NA | NA |
| OTU4151 | NA | + | + | NA | NA | NA |
| OTU4100 | NA | NA | NA | NA | + | NA |
| OTU1343 | NA | + | NA | + | NA | NA |
| OTU3350 | + | NA | NA | NA | NA | NA |
| OTU5912 | NA | NA | NA | NA | NA | NA |
| OTU3064 | NA | NA | NA | + | NA | NA |
| OTU2603 | NA | NA | NA | NA | + | NA |
| OTU7608 | NA | NA | NA | NA | NA | NA |
| OTU3772 | + | NA | NA | NA | NA | NA |
| OTU92 | NA | NA | + | NA | NA | NA |
| OTU5502 | NA | NA | NA | NA | + | NA |
| OTU4983 | NA | + | NA | NA | NA | NA |
| OTU7450 | + | NA | NA | NA | NA | NA |
| OTU4891 | + | NA | NA | NA | NA | NA |
| OTU7434 | NA | NA | NA | NA | NA | NA |
| OTU5147 | NA | + | NA | NA | NA | NA |
| OTU6175 | NA | NA | NA | NA | NA | + |
| OTU3035 | NA | NA | + | NA | NA | NA |
| OTU606 | NA | NA | + | NA | NA | NA |
| OTU4785 | NA | NA | NA | NA | + | NA |
| OTU3738 | NA | NA | NA | + | NA | NA |
| OTU4917 | + | NA | NA | NA | NA | NA |
| OTU1806 | NA | NA | + | NA | NA | NA |
| OTU1466 | NA | NA | NA | NA | NA | NA |
| OTU7926 | + | NA | NA | NA | NA | NA |
| OTU8066 | NA | NA | NA | NA | NA | NA |
| OTU6524 | NA | NA | NA | NA | NA | NA |
| OTU3277 | NA | NA | + | NA | NA | NA |
| OTU4301 | NA | NA | NA | + | NA | NA |
| OTU676 | NA | NA | + | NA | NA | NA |
| OTU1203 | NA | NA | NA | NA | NA | + |
| OTU4611 | NA | NA | NA | NA | NA | NA |
| OTU6164 | NA | NA | + | NA | NA | NA |
| OTU553 | NA | NA | NA | NA | NA | NA |
| OTU5106 | NA | NA | NA | NA | + | NA |
| OTU557 | NA | NA | + | NA | NA | NA |
| OTU559 | + | NA | + | NA | NA | NA |
| OTU4098 | NA | NA | NA | NA | + | NA |
| OTU2996 | NA | NA | + | NA | NA | NA |
| OTU687 | NA | NA | + | NA | NA | NA |
| OTU5411 | NA | NA | NA | NA | + | NA |
| OTU758 | NA | NA | NA | NA | NA | NA |
| OTU4532 | + | NA | NA | NA | NA | NA |
| OTU246 | NA | + | NA | NA | NA | NA |
| OTU1190 | + | NA | + | NA | NA | NA |
| OTU358 | NA | NA | NA | + | NA | NA |
| OTU3433 | NA | NA | NA | + | NA | NA |
| OTU8180 | NA | NA | + | NA | NA | NA |
| OTU8186 | NA | NA | + | NA | NA | + |
| OTU2149 | NA | NA | NA | NA | NA | + |
| OTU3536 | NA | NA | NA | + | NA | NA |
| OTU3538 | NA | NA | NA | + | NA | NA |
| OTU5694 | + | NA | NA | NA | NA | NA |
| OTU1753 | NA | NA | + | NA | NA | NA |
| OTU5488 | NA | NA | NA | NA | + | NA |
| OTU1750 | NA | NA | NA | + | NA | NA |
| OTU3453 | NA | NA | NA | + | NA | NA |
| OTU5491 | + | NA | NA | NA | NA | NA |
| OTU1673 | NA | NA | + | NA | NA | NA |
| OTU1499 | + | NA | NA | NA | NA | NA |
| OTU3131 | + | NA | NA | NA | NA | NA |
| OTU7965 | NA | NA | NA | + | NA | NA |
| OTU6317 | NA | + | NA | NA | NA | NA |
| OTU1979 | NA | NA | NA | NA | NA | NA |
| OTU8024 | NA | NA | NA | NA | NA | + |
| OTU7105 | NA | NA | + | NA | NA | NA |
| OTU6507 | NA | NA | NA | NA | NA | + |
| OTU2295 | NA | NA | NA | NA | NA | NA |
| OTU130 | NA | NA | NA | + | NA | NA |
| OTU2968 | NA | NA | + | NA | NA | NA |
| OTU5962 | NA | + | NA | NA | NA | NA |
| OTU3547 | NA | NA | NA | + | + | NA |
| OTU645 | NA | NA | + | NA | NA | NA |
| OTU2051 | NA | NA | NA | NA | NA | NA |
| OTU2252 | + | NA | NA | NA | NA | NA |
| OTU6337 | NA | NA | NA | NA | + | NA |
| OTU7437 | NA | NA | NA | NA | NA | + |
| OTU2957 | NA | NA | + | NA | NA | NA |
| OTU7113 | NA | NA | + | NA | NA | NA |
| OTU7110 | NA | NA | + | NA | NA | NA |
| OTU1609 | + | NA | NA | NA | NA | NA |
| OTU2978 | NA | NA | + | NA | NA | NA |
| OTU2946 | + | NA | NA | NA | NA | NA |
| OTU4316 | NA | NA | NA | + | NA | NA |
| OTU4313 | NA | NA | NA | + | NA | NA |
| OTU4530 | + | NA | NA | NA | NA | NA |
| OTU7030 | NA | NA | + | NA | NA | NA |
| OTU1940 | NA | NA | + | NA | NA | NA |
| OTU4314 | NA | NA | NA | + | NA | NA |
| OTU6984 | NA | NA | NA | NA | NA | + |
| OTU4311 | NA | NA | NA | + | NA | NA |
| OTU4312 | NA | NA | NA | + | NA | NA |
| OTU7474 | + | NA | NA | NA | NA | NA |
| OTU7475 | + | NA | NA | NA | NA | NA |
| OTU5192 | NA | NA | NA | NA | + | NA |
| OTU2856 | + | NA | NA | NA | NA | NA |
| OTU3146 | NA | NA | NA | NA | + | NA |
| OTU685 | NA | NA | + | NA | NA | NA |
| OTU517 | NA | NA | + | NA | NA | NA |
| OTU5557 | + | NA | NA | NA | NA | NA |
| OTU5885 | NA | NA | NA | NA | NA | + |
| OTU1565 | NA | NA | NA | NA | NA | NA |
| OTU5047 | NA | NA | + | NA | NA | NA |
| OTU3727 | NA | + | NA | NA | NA | + |
| OTU3054 | NA | NA | NA | + | NA | NA |
| OTU6612 | + | NA | NA | NA | NA | NA |
| OTU1892 | NA | NA | + | NA | NA | NA |
| OTU7944 | NA | NA | NA | + | NA | NA |
| OTU4857 | + | NA | NA | NA | NA | NA |
| OTU5386 | NA | NA | NA | + | NA | NA |
| OTU5730 | NA | + | NA | NA | NA | NA |
| OTU3783 | NA | NA | NA | + | NA | NA |
| OTU751 | + | NA | NA | NA | NA | NA |
| OTU3156 | + | NA | NA | NA | NA | NA |
| OTU7191 | NA | NA | NA | NA | NA | + |
| OTU7197 | NA | NA | NA | NA | NA | NA |
| OTU4736 | NA | NA | NA | NA | NA | NA |
| OTU7506 | NA | + | + | NA | NA | NA |
| OTU5130 | NA | + | NA | NA | NA | NA |
| OTU4586 | NA | NA | NA | NA | + | NA |
| OTU4495 | + | NA | NA | NA | NA | NA |
| OTU4588 | + | NA | NA | NA | NA | NA |
| OTU8036 | + | NA | NA | NA | NA | NA |
| OTU3736 | NA | NA | NA | NA | NA | NA |
| OTU5518 | NA | NA | NA | + | NA | NA |
| OTU5873 | NA | NA | NA | NA | NA | + |
| OTU4794 | NA | NA | + | NA | NA | NA |
| OTU4421 | + | NA | NA | NA | NA | NA |
| OTU2065 | NA | NA | NA | NA | NA | NA |
| OTU7746 | NA | NA | NA | NA | NA | + |
| OTU7632 | + | + | NA | NA | NA | NA |
| OTU6376 | NA | + | NA | NA | NA | NA |
| OTU5511 | NA | NA | NA | NA | NA | NA |
| OTU4806 | NA | NA | NA | + | + | NA |
| OTU5700 | + | NA | NA | NA | NA | NA |
| OTU4059 | NA | NA | NA | NA | NA | NA |
| OTU8261 | NA | NA | NA | + | NA | NA |
| OTU905 | NA | NA | NA | NA | NA | NA |
| OTU3429 | NA | NA | NA | + | NA | NA |
| OTU5459 | + | NA | NA | NA | NA | NA |
| OTU5530 | NA | NA | NA | NA | NA | NA |
| OTU4440 | + | NA | NA | NA | NA | NA |
| OTU5269 | NA | NA | NA | NA | + | NA |
| OTU5265 | NA | NA | NA | NA | + | NA |
| OTU5590 | NA | NA | NA | + | NA | NA |
| OTU4168 | NA | + | NA | NA | NA | NA |
| OTU6435 | NA | NA | NA | NA | NA | NA |
| OTU5844 | NA | NA | NA | NA | NA | + |
| OTU6002 | NA | NA | + | NA | NA | NA |
| OTU7119 | NA | NA | + | NA | NA | NA |
| OTU7112 | NA | NA | + | NA | NA | NA |
| OTU1138 | NA | NA | NA | + | NA | NA |
| OTU6319 | NA | NA | NA | + | NA | NA |
| OTU1135 | NA | NA | + | NA | NA | NA |
| OTU7343 | NA | + | NA | NA | NA | NA |
| OTU5373 | NA | NA | NA | NA | NA | NA |
| OTU7157 | NA | NA | NA | NA | NA | NA |
| OTU4479 | + | NA | NA | NA | NA | NA |
| OTU5387 | NA | NA | NA | + | NA | NA |
| OTU3164 | NA | + | NA | NA | + | NA |
| OTU3555 | NA | + | NA | NA | NA | NA |
| OTU3880 | NA | NA | NA | NA | NA | NA |
| OTU5955 | NA | NA | NA | + | NA | NA |
| OTU882 | NA | NA | NA | NA | + | NA |
| OTU4644 | NA | NA | NA | NA | NA | + |
| OTU3896 | NA | NA | + | NA | NA | NA |
| OTU5438 | + | NA | NA | NA | NA | NA |
| OTU2366 | NA | NA | + | NA | NA | NA |
| OTU7153 | NA | NA | + | NA | NA | NA |
| OTU6100 | NA | NA | NA | + | NA | NA |
| OTU4484 | + | NA | NA | NA | NA | NA |
| OTU3107 | NA | NA | NA | NA | NA | NA |
| OTU53 | NA | NA | + | NA | NA | NA |
| OTU6024 | NA | NA | + | NA | NA | NA |
| OTU3086 | NA | NA | NA | + | NA | NA |
| OTU7443 | + | NA | NA | NA | NA | NA |
| OTU1921 | NA | NA | NA | + | NA | NA |
| OTU3088 | NA | NA | NA | + | NA | NA |
| OTU2902 | NA | NA | + | NA | NA | NA |
| OTU6649 | NA | NA | + | NA | NA | NA |
| OTU2272 | NA | NA | NA | NA | + | NA |
| OTU5383 | NA | NA | NA | NA | + | NA |
| OTU2971 | NA | NA | + | NA | NA | NA |
| OTU5221 | NA | NA | NA | + | NA | NA |
| OTU2334 | NA | NA | NA | NA | NA | + |
| OTU4236 | NA | + | NA | NA | NA | NA |
| OTU2773 | NA | NA | NA | NA | NA | NA |
| OTU2303 | NA | NA | NA | NA | NA | + |
| OTU4871 | + | NA | NA | NA | NA | NA |
| OTU3113 | + | NA | NA | NA | NA | NA |
| OTU4295 | NA | NA | NA | + | NA | NA |
| OTU2107 | NA | NA | + | NA | NA | NA |
| OTU5901 | NA | NA | NA | NA | NA | + |
| OTU2109 | NA | NA | NA | NA | + | NA |
| OTU5908 | NA | NA | NA | NA | NA | + |
| OTU570 | NA | NA | + | NA | NA | NA |
| OTU5342 | NA | + | NA | NA | NA | NA |
| OTU6349 | NA | NA | + | NA | NA | NA |
| OTU5918 | NA | NA | NA | NA | NA | + |
| OTU4502 | + | NA | NA | NA | NA | NA |
| OTU4509 | + | NA | NA | NA | NA | NA |
| OTU750 | NA | NA | NA | NA | NA | NA |
| OTU8152 | NA | NA | + | NA | NA | NA |
| OTU4370 | NA | NA | NA | NA | NA | NA |
| OTU579 | NA | NA | + | NA | NA | NA |
| OTU576 | NA | NA | + | NA | NA | NA |
| OTU6889 | NA | NA | + | NA | + | NA |
| OTU3459 | NA | NA | NA | + | NA | NA |
| OTU2158 | NA | NA | NA | NA | NA | + |
| OTU7257 | NA | NA | + | NA | NA | NA |
| OTU7350 | NA | NA | NA | + | NA | NA |
| OTU2369 | NA | NA | + | NA | NA | NA |
| OTU5935 | NA | NA | NA | NA | + | NA |
| OTU6029 | + | NA | NA | NA | NA | NA |
| OTU4513 | NA | NA | NA | NA | NA | NA |
| OTU6597 | NA | NA | NA | NA | NA | + |
| OTU5802 | NA | NA | NA | NA | NA | + |
| OTU5702 | NA | NA | NA | NA | + | NA |
| OTU5705 | + | NA | NA | NA | NA | NA |
| OTU3548 | + | NA | NA | NA | NA | NA |
| OTU2344 | NA | NA | + | NA | NA | NA |
| OTU4287 | NA | NA | NA | + | NA | NA |
| OTU4607 | NA | NA | NA | NA | NA | NA |
| OTU4748 | NA | NA | NA | NA | + | NA |
| OTU6371 | NA | NA | NA | NA | NA | + |
| OTU6372 | NA | NA | NA | + | NA | NA |
| OTU5003 | + | NA | NA | NA | + | NA |
| OTU4072 | NA | + | + | NA | NA | NA |
| OTU3315 | NA | NA | NA | NA | + | NA |
| OTU1143 | NA | NA | + | NA | NA | NA |
| OTU3909 | + | NA | NA | NA | + | NA |
| OTU1867 | NA | NA | + | NA | NA | NA |
| OTU456 | + | NA | NA | NA | NA | NA |
| OTU4206 | + | NA | NA | NA | NA | NA |
| OTU2013 | NA | NA | NA | + | NA | NA |
| OTU6725 | NA | NA | + | NA | NA | NA |
| OTU2089 | NA | NA | NA | + | NA | NA |
| OTU4661 | + | NA | NA | NA | NA | NA |
| OTU1713 | NA | NA | + | NA | NA | NA |
| OTU6080 | NA | NA | NA | NA | NA | NA |
| OTU1908 | NA | NA | NA | + | NA | NA |
| OTU1443 | NA | NA | NA | + | NA | + |
| OTU7162 | NA | NA | + | NA | NA | NA |
| OTU5249 | NA | NA | NA | NA | + | NA |
| OTU109 | NA | NA | + | NA | NA | NA |
| OTU6417 | NA | NA | NA | NA | NA | + |
| OTU280 | NA | NA | + | NA | NA | NA |
| OTU4533 | + | NA | NA | NA | NA | NA |
| OTU4560 | NA | NA | NA | NA | + | NA |
| OTU4338 | NA | NA | NA | + | NA | NA |
| OTU4335 | NA | NA | NA | + | NA | NA |
| OTU4332 | NA | NA | NA | + | NA | NA |
| OTU4333 | NA | NA | NA | + | NA | NA |
| OTU556 | NA | NA | + | NA | NA | NA |
| OTU7750 | NA | NA | NA | NA | NA | NA |
| OTU3941 | NA | + | NA | NA | NA | NA |
| OTU3942 | NA | + | NA | NA | NA | NA |
| OTU3943 | NA | NA | NA | + | NA | NA |
| OTU3945 | NA | NA | NA | NA | NA | + |
| OTU766 | + | NA | NA | NA | NA | NA |
| OTU2731 | NA | NA | + | NA | NA | NA |
| OTU761 | NA | NA | NA | + | NA | NA |
| OTU6215 | NA | NA | NA | + | NA | NA |
| OTU4194 | NA | + | NA | NA | NA | NA |
| OTU4199 | NA | NA | NA | NA | NA | NA |
| OTU2491 | NA | NA | NA | NA | NA | + |
| OTU6726 | + | NA | NA | NA | NA | NA |
| OTU6564 | NA | NA | NA | NA | NA | + |
| OTU5068 | NA | + | NA | NA | NA | NA |
| OTU1010 | NA | NA | NA | + | NA | NA |
| OTU642 | NA | NA | + | NA | NA | NA |
| OTU5063 | NA | NA | + | NA | NA | NA |
| OTU6748 | NA | NA | NA | + | NA | NA |
| OTU6747 | NA | + | NA | NA | NA | NA |
| OTU2025 | NA | NA | NA | NA | NA | + |
| OTU3430 | NA | NA | NA | + | NA | NA |
| OTU4547 | NA | NA | NA | NA | + | NA |
| OTU598 | NA | NA | + | NA | NA | NA |
| OTU4402 | + | NA | NA | NA | NA | NA |
| OTU2340 | NA | NA | + | NA | NA | NA |
| OTU2733 | NA | + | NA | NA | NA | NA |
| OTU3437 | NA | NA | NA | + | NA | NA |
| OTU4624 | NA | + | NA | NA | NA | NA |
| OTU5191 | + | NA | NA | NA | NA | NA |
| OTU2167 | + | NA | NA | NA | NA | NA |
| OTU6914 | NA | NA | + | NA | NA | NA |
| OTU6874 | NA | NA | NA | NA | + | NA |
| OTU3719 | NA | + | + | NA | NA | NA |
| OTU516 | NA | NA | + | NA | NA | NA |
| OTU3711 | NA | NA | + | NA | NA | NA |
| OTU7098 | NA | NA | + | NA | NA | NA |
| OTU345 | NA | NA | + | NA | NA | NA |
| OTU5064 | + | NA | NA | NA | NA | NA |
| OTU240 | NA | NA | + | NA | NA | NA |
| OTU4792 | NA | NA | NA | NA | NA | NA |
| OTU8287 | NA | NA | + | NA | NA | NA |
| OTU2936 | + | NA | NA | NA | NA | NA |
| OTU2867 | NA | NA | + | NA | NA | NA |
| OTU3342 | NA | NA | NA | + | NA | NA |
| OTU3375 | NA | NA | NA | + | NA | NA |
| OTU1594 | NA | NA | NA | + | NA | NA |
| OTU8275 | NA | NA | + | NA | NA | NA |
| OTU4272 | NA | NA | NA | + | NA | NA |
| OTU4190 | NA | + | NA | NA | NA | NA |
| OTU441 | NA | + | NA | NA | NA | NA |
| OTU446 | NA | NA | + | NA | NA | NA |
| OTU945 | NA | NA | + | NA | NA | NA |
| OTU2564 | + | + | NA | NA | NA | NA |
| OTU2565 | NA | NA | NA | NA | NA | NA |
| OTU2214 | + | NA | NA | NA | NA | NA |
| OTU5724 | NA | + | NA | NA | NA | NA |
| OTU8127 | NA | NA | NA | + | NA | NA |
| OTU1761 | NA | NA | NA | NA | NA | NA |
| OTU552 | NA | NA | + | NA | NA | NA |
| OTU8232 | NA | NA | + | NA | NA | NA |
| OTU2790 | NA | NA | NA | NA | + | NA |
| OTU518 | NA | NA | + | NA | NA | NA |
| OTU1015 | NA | NA | NA | NA | NA | NA |
| OTU514 | NA | NA | NA | NA | NA | NA |
| OTU2470 | NA | NA | NA | + | NA | NA |
| OTU512 | NA | NA | + | NA | NA | NA |
| OTU5266 | NA | NA | NA | NA | + | NA |
| OTU5449 | + | NA | NA | NA | NA | NA |
| OTU3376 | NA | NA | NA | NA | NA | NA |
| OTU4452 | + | NA | NA | NA | NA | NA |
| OTU4910 | + | NA | NA | NA | NA | NA |
| OTU6110 | NA | NA | + | NA | NA | NA |
| OTU5015 | NA | + | NA | NA | NA | NA |
| OTU6103 | NA | NA | NA | NA | NA | NA |
| OTU4890 | NA | NA | NA | NA | NA | NA |
| OTU2023 | NA | NA | NA | NA | NA | + |
| OTU1458 | NA | NA | NA | + | NA | NA |
| OTU538 | NA | NA | NA | NA | NA | NA |
| OTU1186 | NA | NA | + | NA | NA | NA |
| OTU4664 | NA | NA | + | NA | NA | NA |
| OTU4434 | + | NA | NA | NA | NA | NA |
| OTU3574 | NA | NA | + | NA | NA | NA |
| OTU1994 | NA | NA | NA | NA | NA | + |
| OTU7326 | NA | + | + | NA | NA | NA |
| OTU4967 | + | NA | NA | NA | NA | NA |
| OTU1714 | NA | NA | + | NA | NA | NA |
| OTU1136 | NA | NA | + | NA | NA | + |
| OTU6358 | NA | NA | NA | NA | + | NA |
| OTU878 | NA | NA | NA | NA | NA | NA |
| OTU6603 | NA | NA | NA | NA | NA | NA |
| OTU4012 | + | NA | NA | NA | NA | NA |
| OTU3049 | NA | NA | NA | NA | NA | NA |
| OTU5287 | NA | NA | NA | NA | NA | NA |
| OTU5536 | + | NA | NA | NA | NA | NA |
| OTU3324 | NA | NA | NA | + | NA | NA |
| OTU2066 | NA | NA | + | NA | NA | NA |
| OTU3174 | + | NA | NA | NA | NA | NA |
| OTU2774 | NA | NA | NA | NA | NA | NA |
| OTU2148 | NA | NA | NA | NA | + | NA |
| OTU2298 | NA | NA | NA | NA | + | NA |
| OTU2650 | + | NA | NA | NA | + | NA |
| OTU3126 | NA | NA | NA | + | NA | + |
| OTU1937 | NA | + | NA | NA | NA | NA |
| OTU4264 | NA | NA | NA | + | NA | NA |
| OTU6350 | NA | NA | NA | NA | NA | + |
| OTU4015 | + | NA | NA | NA | NA | NA |
| OTU4620 | NA | + | NA | NA | NA | NA |
| OTU2904 | NA | NA | NA | + | NA | NA |
| OTU885 | NA | NA | NA | + | NA | NA |
| OTU5126 | + | NA | NA | NA | NA | NA |
| OTU2706 | NA | NA | + | NA | NA | NA |
| OTU3015 | NA | NA | + | NA | NA | NA |
| OTU318 | NA | NA | NA | NA | NA | + |
| OTU2925 | NA | NA | NA | NA | NA | + |
| OTU2922 | NA | NA | NA | NA | NA | + |
| OTU4310 | NA | NA | NA | + | NA | NA |
| OTU1430 | NA | + | NA | NA | NA | NA |
| OTU5206 | NA | NA | NA | NA | + | NA |
| OTU2879 | NA | + | NA | NA | NA | NA |
| OTU3104 | NA | NA | NA | + | NA | NA |
| OTU1744 | NA | NA | NA | NA | NA | NA |
| OTU831 | NA | NA | NA | NA | NA | NA |
| OTU571 | NA | NA | + | NA | NA | NA |
| OTU7398 | NA | NA | NA | + | NA | NA |
| OTU6401 | + | NA | NA | NA | NA | NA |
| OTU6605 | NA | NA | NA | + | NA | NA |
| OTU3966 | NA | NA | + | NA | NA | NA |
| OTU3965 | NA | NA | + | NA | NA | NA |
| OTU748 | NA | NA | NA | + | NA | NA |
| OTU749 | NA | NA | NA | + | NA | NA |
| OTU741 | + | NA | + | NA | NA | NA |
| OTU5125 | + | NA | NA | NA | NA | NA |
| OTU425 | NA | NA | + | NA | NA | NA |
| OTU5732 | NA | + | NA | NA | NA | NA |
| OTU7240 | NA | NA | + | NA | NA | NA |
| OTU4498 | + | NA | NA | NA | NA | NA |
| OTU5900 | NA | NA | NA | NA | NA | + |
| OTU1974 | NA | NA | + | NA | NA | NA |
| OTU4056 | NA | NA | NA | NA | + | NA |
| OTU6769 | NA | NA | + | NA | NA | NA |
| OTU4169 | NA | + | NA | NA | NA | NA |
| OTU6790 | NA | NA | NA | NA | NA | NA |
| OTU3388 | NA | NA | NA | + | NA | NA |
| OTU573 | NA | NA | NA | NA | NA | NA |
| OTU2402 | NA | NA | + | NA | NA | NA |
| OTU3385 | NA | NA | NA | + | NA | NA |
| OTU7400 | + | NA | NA | NA | NA | NA |
| OTU835 | NA | NA | NA | + | NA | NA |
| OTU1586 | NA | NA | NA | NA | + | NA |
| OTU8014 | NA | NA | + | NA | NA | NA |
| OTU1865 | NA | NA | NA | NA | + | NA |
| OTU1011 | NA | NA | + | NA | NA | NA |
| OTU4192 | NA | + | NA | NA | NA | NA |
| OTU4722 | NA | NA | NA | NA | NA | NA |
| OTU5299 | NA | NA | NA | + | NA | NA |
| OTU6585 | NA | NA | + | NA | NA | NA |
| OTU5498 | + | NA | NA | NA | NA | NA |
| OTU7282 | NA | NA | NA | + | NA | NA |
| OTU5887 | NA | NA | NA | NA | NA | + |
| OTU1391 | NA | NA | NA | + | NA | NA |
| OTU3397 | NA | NA | NA | + | NA | NA |
| OTU3533 | NA | NA | NA | NA | + | NA |
| OTU4344 | NA | NA | NA | NA | NA | NA |
| OTU1234 | NA | + | NA | NA | NA | NA |
| OTU1803 | NA | NA | + | NA | NA | NA |
| OTU4601 | NA | + | NA | NA | NA | NA |
| OTU1026 | NA | NA | NA | NA | NA | NA |
| OTU1572 | NA | NA | NA | NA | + | NA |
| OTU2588 | NA | NA | NA | + | NA | NA |
| OTU4836 | NA | NA | + | NA | NA | NA |
| OTU4837 | NA | NA | NA | NA | + | NA |
| OTU5804 | NA | NA | NA | NA | NA | NA |
| OTU6185 | NA | NA | + | NA | NA | NA |
| OTU6602 | NA | NA | + | NA | NA | NA |
| OTU8146 | NA | NA | + | NA | NA | NA |
| OTU3178 | + | NA | NA | NA | NA | NA |
| OTU5000 | + | + | NA | NA | + | NA |
| OTU5010 | NA | NA | NA | NA | + | NA |
| OTU1120 | NA | NA | NA | NA | NA | NA |
| OTU6063 | NA | NA | + | NA | NA | NA |
| OTU5496 | + | NA | NA | NA | NA | NA |
| OTU2803 | NA | NA | + | NA | NA | NA |
| OTU4712 | NA | + | NA | NA | NA | NA |
| OTU4713 | NA | NA | NA | NA | NA | NA |
| OTU4716 | NA | NA | NA | NA | NA | NA |
| OTU2956 | NA | NA | + | NA | NA | NA |
| OTU2882 | + | NA | NA | NA | NA | NA |
| OTU7130 | NA | NA | + | NA | NA | NA |
| OTU603 | NA | NA | + | NA | NA | NA |
| OTU3776 | NA | NA | + | NA | NA | NA |
| OTU7092 | NA | NA | + | NA | NA | NA |
| OTU4334 | NA | NA | NA | + | NA | NA |
| OTU3102 | NA | NA | NA | NA | NA | NA |
| OTU3779 | NA | NA | NA | + | NA | NA |
| OTU3373 | NA | NA | + | NA | NA | NA |
| OTU4196 | NA | + | NA | NA | NA | NA |
| OTU4146 | NA | + | NA | NA | NA | NA |
| OTU2424 | NA | NA | NA | + | NA | NA |
| OTU7575 | NA | NA | NA | NA | NA | NA |
| OTU670 | NA | NA | + | NA | NA | NA |
| OTU3534 | NA | NA | NA | NA | + | NA |
| OTU1672 | NA | NA | NA | + | NA | NA |
| OTU3597 | + | NA | NA | NA | NA | NA |
| OTU3595 | + | NA | NA | NA | NA | NA |
| OTU6165 | NA | NA | NA | NA | NA | NA |
| OTU1736 | NA | NA | + | NA | NA | NA |
| OTU8004 | NA | NA | NA | + | NA | NA |
| OTU3367 | NA | NA | NA | + | NA | NA |
| OTU4271 | NA | NA | NA | + | NA | NA |
| OTU4275 | NA | NA | NA | + | NA | NA |
| OTU4359 | NA | NA | NA | NA | NA | NA |
| OTU6723 | NA | NA | NA | NA | + | NA |
| OTU3673 | + | NA | NA | NA | NA | NA |
| OTU3296 | NA | NA | NA | NA | NA | NA |
| OTU1996 | + | NA | NA | NA | NA | NA |
| OTU7915 | NA | NA | NA | NA | NA | + |
| OTU2506 | NA | NA | NA | + | NA | NA |
| OTU2638 | NA | NA | + | NA | NA | NA |
| OTU5721 | NA | NA | NA | NA | + | NA |
| OTU2999 | NA | + | NA | NA | NA | NA |
| OTU2393 | NA | NA | + | NA | NA | NA |
| OTU432 | NA | NA | + | NA | NA | NA |
| OTU2937 | NA | NA | NA | NA | NA | NA |
| OTU2943 | NA | NA | NA | + | NA | NA |
| OTU7321 | NA | NA | NA | + | NA | NA |
| OTU3071 | NA | NA | NA | + | NA | NA |
| OTU1388 | NA | NA | + | NA | NA | NA |
| OTU5563 | NA | NA | NA | NA | + | NA |
| OTU5638 | + | NA | NA | NA | NA | NA |
| OTU5637 | NA | NA | NA | NA | NA | NA |
| OTU5632 | + | NA | NA | NA | NA | NA |
| OTU1992 | NA | NA | NA | + | NA | NA |
| OTU6069 | NA | NA | + | NA | NA | NA |
| OTU5055 | NA | NA | + | NA | NA | NA |
| OTU4942 | NA | NA | NA | NA | + | NA |
| OTU6335 | NA | NA | NA | NA | NA | + |
| OTU515 | NA | NA | + | NA | NA | NA |
| OTU2296 | + | NA | NA | NA | NA | NA |
| OTU5711 | NA | + | NA | NA | NA | NA |
| OTU7076 | NA | NA | NA | NA | NA | NA |
| OTU8267 | NA | NA | NA | + | NA | NA |
| OTU7822 | NA | + | NA | NA | NA | NA |
| OTU900 | NA | NA | + | NA | NA | NA |
| OTU689 | NA | NA | + | NA | NA | NA |
| OTU684 | NA | NA | + | NA | NA | NA |
| OTU686 | NA | NA | + | NA | NA | NA |
| OTU7947 | NA | NA | + | NA | NA | NA |
| OTU4018 | NA | NA | NA | NA | + | NA |
| OTU6931 | NA | NA | NA | + | NA | NA |
| OTU2708 | NA | NA | NA | NA | + | NA |
| OTU8215 | + | NA | NA | NA | NA | NA |
| OTU2920 | NA | NA | NA | + | NA | NA |
| OTU6047 | NA | NA | + | NA | NA | NA |
| OTU7126 | NA | NA | + | NA | NA | NA |
| OTU3097 | NA | NA | NA | + | NA | NA |
| OTU536 | NA | NA | + | NA | NA | NA |
| OTU535 | + | NA | NA | NA | NA | NA |
| OTU532 | NA | NA | + | NA | NA | NA |
| OTU530 | NA | NA | + | NA | NA | NA |
| OTU3143 | NA | NA | NA | NA | NA | NA |
| OTU3276 | NA | NA | NA | NA | NA | + |
| OTU1468 | NA | + | + | NA | NA | NA |
| OTU7023 | NA | NA | + | NA | NA | NA |
| OTU2484 | NA | NA | NA | NA | NA | NA |
| OTU4885 | + | NA | NA | NA | NA | NA |
| OTU5658 | + | NA | NA | NA | NA | NA |
| OTU6056 | NA | + | NA | NA | NA | NA |
| OTU5592 | NA | NA | + | NA | NA | NA |
| OTU5856 | NA | NA | NA | NA | NA | + |
| OTU6510 | NA | NA | NA | + | NA | NA |
| OTU4285 | NA | NA | NA | + | NA | NA |
| OTU2081 | NA | NA | NA | + | NA | NA |
| OTU1780 | NA | NA | + | NA | NA | NA |
| OTU4150 | NA | + | NA | NA | NA | NA |
| OTU3725 | + | NA | NA | NA | NA | NA |
| OTU1980 | NA | + | NA | NA | NA | NA |
| OTU3569 | NA | NA | + | NA | NA | NA |
| OTU108 | NA | NA | NA | NA | NA | NA |
| OTU4379 | NA | NA | NA | NA | + | NA |
| OTU5741 | + | NA | NA | NA | NA | NA |
| OTU4140 | NA | NA | NA | NA | NA | NA |
| OTU7019 | NA | NA | + | NA | NA | NA |
| OTU6551 | NA | NA | NA | NA | NA | + |
| OTU136 | NA | NA | + | NA | NA | NA |
| OTU4732 | NA | + | NA | NA | NA | NA |
| OTU6992 | NA | NA | NA | + | NA | NA |
| OTU1359 | NA | NA | NA | + | NA | NA |
| OTU7884 | NA | NA | + | NA | NA | NA |
| OTU501 | NA | NA | + | NA | NA | NA |
| OTU6920 | NA | NA | + | NA | NA | NA |
| OTU4034 | NA | + | NA | NA | NA | NA |
| OTU3434 | NA | NA | NA | + | NA | NA |
| OTU5022 | + | NA | NA | NA | NA | NA |
| OTU3345 | NA | NA | NA | + | NA | NA |
| OTU6436 | NA | NA | NA | NA | NA | NA |
| OTU7081 | NA | NA | + | NA | NA | NA |
| OTU3129 | NA | + | NA | NA | NA | NA |
| OTU2969 | NA | NA | + | NA | NA | NA |
| OTU5862 | NA | NA | NA | + | NA | NA |
| OTU1054 | NA | NA | NA | + | NA | NA |
| OTU1053 | NA | + | NA | + | NA | NA |
| OTU6775 | + | NA | NA | NA | NA | NA |
| OTU4487 | + | NA | NA | NA | NA | NA |
| OTU455 | + | NA | NA | NA | NA | NA |
| OTU451 | NA | NA | NA | NA | NA | NA |
| OTU5625 | NA | NA | NA | NA | NA | + |
| OTU2038 | NA | NA | NA | NA | NA | NA |
| OTU4261 | NA | NA | NA | + | NA | NA |
| OTU4263 | NA | NA | NA | NA | NA | NA |
| OTU6172 | + | NA | NA | NA | NA | NA |
| OTU5148 | NA | NA | NA | NA | + | NA |
| OTU3466 | NA | NA | NA | + | NA | NA |
| OTU3461 | NA | NA | NA | NA | NA | NA |
| OTU2559 | NA | NA | NA | NA | NA | NA |
| OTU4621 | NA | + | NA | NA | NA | NA |
| OTU590 | NA | NA | + | NA | NA | NA |
| OTU637 | NA | NA | + | NA | NA | NA |
| OTU3173 | NA | NA | NA | NA | NA | + |
| OTU7689 | NA | NA | NA | NA | NA | + |
| OTU8020 | NA | + | NA | NA | NA | NA |
| OTU3904 | NA | NA | NA | NA | + | NA |
| OTU3905 | NA | NA | + | NA | NA | NA |
| OTU3902 | NA | NA | NA | NA | NA | NA |
| OTU4949 | + | NA | NA | NA | NA | NA |
| OTU7287 | NA | NA | NA | + | NA | NA |
| OTU4152 | NA | NA | NA | + | NA | NA |
| OTU7284 | + | NA | NA | NA | NA | NA |
| OTU4887 | NA | NA | NA | NA | NA | NA |
| OTU5238 | + | NA | NA | NA | NA | NA |
| OTU3103 | NA | NA | NA | + | NA | NA |
| OTU1869 | NA | NA | NA | NA | NA | NA |
| OTU5471 | + | NA | NA | NA | NA | NA |
| OTU5964 | NA | NA | NA | NA | NA | + |
| OTU1 | NA | NA | + | NA | NA | NA |
| OTU5252 | NA | NA | NA | NA | + | NA |
| OTU3741 | NA | NA | NA | NA | NA | + |
| OTU7526 | NA | NA | + | NA | NA | NA |
| OTU4375 | NA | NA | NA | NA | + | NA |
| OTU3775 | NA | NA | NA | + | NA | NA |
| OTU6259 | NA | NA | NA | NA | NA | + |
| OTU3580 | NA | NA | NA | + | NA | NA |
| OTU967 | NA | NA | + | NA | NA | NA |
| OTU4129 | NA | NA | NA | + | + | NA |
| OTU7085 | NA | NA | + | NA | NA | NA |
| OTU3975 | NA | + | NA | NA | NA | NA |
| OTU4589 | NA | NA | NA | NA | + | NA |
| OTU4504 | + | NA | NA | NA | NA | NA |
| OTU4191 | NA | NA | NA | NA | NA | NA |
| OTU4843 | + | NA | NA | NA | NA | NA |
| OTU4198 | NA | + | NA | NA | NA | NA |
| OTU1317 | NA | NA | NA | NA | NA | + |
| OTU3563 | NA | NA | NA | NA | + | NA |
| OTU2124 | NA | NA | NA | + | NA | NA |
| OTU5896 | NA | NA | NA | NA | NA | + |
| OTU5378 | + | NA | NA | NA | NA | NA |
| OTU2195 | NA | NA | NA | NA | NA | NA |
| OTU4223 | NA | + | NA | NA | NA | NA |
| OTU462 | NA | + | NA | NA | NA | NA |
| OTU5035 | + | NA | NA | NA | + | NA |
| OTU2621 | + | NA | NA | NA | NA | NA |
| OTU6940 | NA | NA | NA | NA | + | NA |
| OTU6267 | NA | NA | NA | + | NA | NA |
| OTU3080 | NA | + | NA | NA | NA | NA |
| OTU3280 | NA | NA | + | NA | NA | NA |
| OTU1522 | NA | NA | + | NA | NA | NA |
| OTU2775 | NA | NA | NA | NA | NA | NA |
| OTU8140 | + | NA | NA | NA | NA | NA |
| OTU438 | + | NA | NA | NA | NA | NA |
| OTU1049 | NA | NA | NA | NA | NA | NA |
| OTU7893 | NA | NA | + | NA | NA | NA |
| OTU7963 | NA | NA | + | NA | NA | + |
| OTU641 | NA | NA | + | NA | NA | NA |
| OTU7968 | NA | NA | + | NA | NA | NA |
| OTU1000 | NA | NA | + | NA | NA | NA |
| OTU3019 | NA | NA | + | NA | NA | NA |
| OTU6422 | NA | NA | + | NA | NA | NA |
| OTU4850 | + | NA | NA | NA | NA | NA |
| OTU5163 | + | NA | NA | NA | NA | NA |
| OTU3348 | NA | NA | NA | + | NA | NA |
| OTU677 | NA | NA | + | NA | NA | NA |
| OTU6427 | NA | NA | NA | NA | NA | NA |
| OTU5734 | NA | + | NA | NA | NA | NA |
| OTU5410 | + | NA | NA | NA | NA | NA |
| OTU6331 | NA | NA | NA | NA | NA | + |
| OTU983 | NA | NA | NA | + | NA | NA |
| OTU4030 | NA | + | NA | NA | NA | NA |
| OTU4033 | NA | + | NA | NA | + | NA |
| OTU8272 | NA | NA | NA | + | NA | NA |
| OTU4233 | NA | + | NA | NA | NA | NA |
| OTU5785 | NA | NA | NA | + | NA | NA |
| OTU7961 | NA | NA | NA | NA | NA | + |
| OTU564 | NA | NA | + | NA | NA | NA |
| OTU3886 | NA | NA | NA | NA | NA | NA |
| OTU4416 | + | NA | NA | NA | NA | NA |
| OTU1989 | NA | NA | NA | NA | NA | NA |
| OTU7070 | NA | NA | + | NA | NA | NA |
| OTU7071 | NA | NA | + | NA | NA | NA |
| OTU7075 | NA | NA | + | NA | NA | NA |
| OTU3474 | NA | NA | NA | + | NA | NA |
| OTU683 | NA | NA | + | NA | NA | NA |
| OTU3354 | NA | NA | NA | + | NA | NA |
| OTU7097 | NA | NA | + | NA | NA | NA |
| OTU6099 | NA | NA | NA | + | NA | NA |
| OTU2448 | NA | NA | NA | + | NA | NA |
| OTU5255 | NA | NA | NA | NA | + | NA |
| OTU4058 | NA | NA | NA | + | NA | NA |
| OTU1302 | NA | NA | + | NA | NA | NA |
| OTU4132 | NA | NA | NA | NA | + | NA |
| OTU8141 | NA | NA | NA | NA | + | NA |
| OTU5656 | + | NA | NA | NA | NA | NA |
| OTU4674 | NA | + | + | NA | NA | NA |
| OTU5847 | NA | NA | NA | NA | NA | NA |
| OTU2111 | NA | NA | + | NA | NA | NA |
| OTU4259 | NA | NA | NA | + | NA | NA |
| OTU8139 | NA | NA | NA | + | NA | NA |
| OTU4250 | NA | NA | NA | + | NA | NA |
| OTU4252 | NA | NA | NA | + | NA | NA |
| OTU4299 | NA | + | NA | NA | NA | NA |
| OTU3398 | NA | NA | NA | + | NA | NA |
| OTU2640 | NA | NA | NA | NA | + | NA |
| OTU584 | NA | NA | + | NA | NA | NA |
| OTU5910 | NA | NA | NA | NA | + | NA |
| OTU3312 | NA | NA | NA | + | NA | NA |
| OTU2899 | NA | NA | NA | NA | NA | NA |
| OTU6560 | NA | NA | NA | NA | NA | + |
| OTU5708 | NA | + | NA | NA | NA | NA |
| OTU325 | NA | NA | + | NA | NA | NA |
| OTU4858 | + | NA | NA | NA | NA | NA |
| OTU5696 | NA | NA | NA | NA | + | NA |
| OTU3499 | NA | NA | + | NA | NA | NA |
| OTU177 | NA | NA | NA | NA | NA | NA |
| OTU3061 | NA | NA | NA | + | NA | NA |
| OTU4525 | + | NA | NA | NA | NA | NA |
| OTU2805 | NA | NA | NA | NA | NA | NA |
| OTU8123 | NA | NA | NA | + | NA | NA |
| OTU7553 | NA | NA | NA | NA | NA | + |
| OTU7550 | NA | NA | NA | + | NA | NA |
| OTU3723 | NA | NA | + | NA | NA | NA |
| OTU1695 | NA | NA | NA | NA | NA | NA |
| OTU6900 | NA | NA | + | NA | NA | NA |
| OTU1025 | NA | NA | + | NA | NA | NA |
| OTU2963 | NA | NA | + | NA | NA | NA |
| OTU2964 | NA | NA | + | NA | NA | NA |
| OTU4463 | + | NA | NA | NA | NA | NA |
| OTU5907 | NA | NA | NA | NA | NA | + |
| OTU4968 | NA | NA | NA | + | NA | NA |
| OTU1318 | NA | NA | + | NA | NA | NA |
| OTU7716 | NA | NA | NA | NA | NA | + |
| OTU1760 | NA | NA | + | NA | NA | NA |
| OTU5604 | NA | NA | NA | NA | NA | NA |
| OTU3044 | NA | NA | NA | NA | NA | NA |
| OTU385 | NA | NA | NA | NA | NA | NA |
| OTU7670 | NA | + | NA | NA | NA | NA |
| OTU6137 | NA | NA | + | NA | NA | NA |
| OTU1068 | NA | NA | + | NA | NA | NA |
| OTU7502 | NA | NA | NA | NA | + | NA |
| OTU4551 | NA | NA | NA | NA | + | NA |
| OTU3008 | NA | NA | NA | NA | NA | NA |
| OTU6418 | NA | NA | NA | + | NA | NA |
| OTU6648 | NA | + | NA | NA | NA | NA |
| OTU3807 | NA | + | NA | NA | NA | NA |
| OTU6680 | NA | NA | NA | NA | + | NA |
| OTU7436 | NA | NA | + | NA | NA | NA |
| OTU1900 | NA | NA | NA | NA | NA | NA |
| OTU3922 | + | NA | + | NA | NA | NA |
| OTU3256 | NA | + | NA | NA | NA | NA |
| OTU4413 | + | NA | NA | NA | NA | NA |
| OTU4410 | + | NA | NA | NA | NA | NA |
| OTU5879 | NA | NA | NA | NA | NA | + |
| OTU2251 | NA | NA | NA | + | NA | NA |
| OTU4119 | + | NA | NA | NA | NA | NA |
| OTU5561 | + | NA | NA | NA | NA | NA |
| OTU5415 | NA | NA | NA | NA | + | NA |
| OTU2847 | NA | NA | NA | NA | + | NA |
| OTU4083 | + | NA | NA | NA | NA | NA |
| OTU1967 | NA | NA | NA | + | NA | NA |
| OTU1775 | NA | NA | NA | NA | NA | NA |
| OTU7312 | NA | NA | + | NA | NA | NA |
| OTU1085 | + | NA | NA | NA | + | NA |
| OTU3507 | NA | NA | NA | + | NA | NA |
| OTU3479 | NA | NA | NA | NA | NA | + |
| OTU2678 | + | NA | NA | NA | NA | NA |
| OTU7604 | NA | NA | NA | + | NA | NA |
| OTU3917 | NA | NA | NA | + | NA | NA |
| OTU5505 | NA | NA | NA | NA | + | NA |
| OTU764 | + | NA | + | NA | NA | NA |
| OTU3046 | NA | NA | NA | + | NA | NA |
| OTU744 | NA | NA | NA | NA | NA | NA |
| OTU5756 | NA | NA | NA | + | NA | NA |
| OTU2958 | NA | NA | + | NA | NA | NA |
| OTU406 | NA | NA | + | NA | NA | NA |
| OTU634 | NA | NA | + | NA | NA | NA |
| OTU2556 | NA | NA | NA | NA | + | NA |
| OTU4616 | NA | + | NA | NA | NA | NA |
| OTU7267 | NA | NA | NA | NA | NA | + |
| OTU5226 | NA | NA | NA | NA | + | NA |
| OTU2750 | NA | NA | NA | NA | NA | NA |
| OTU2758 | NA | NA | NA | + | NA | NA |
| OTU5477 | + | NA | NA | NA | NA | NA |
| OTU2730 | NA | NA | NA | NA | + | NA |
| OTU4352 | NA | NA | NA | + | NA | NA |
| OTU5408 | + | NA | NA | NA | NA | NA |
| OTU7943 | NA | NA | + | NA | NA | NA |
| OTU8051 | NA | NA | NA | NA | NA | NA |
| OTU7995 | NA | NA | NA | NA | NA | + |
| OTU7992 | NA | + | NA | NA | NA | NA |
| OTU1524 | NA | NA | NA | + | NA | NA |
| OTU7403 | NA | NA | NA | NA | NA | + |
| OTU5771 | + | NA | NA | NA | NA | NA |
| OTU5432 | NA | NA | NA | NA | + | NA |
| OTU4854 | + | NA | NA | NA | NA | NA |
| OTU5439 | NA | NA | NA | NA | + | NA |
| OTU476 | NA | NA | NA | NA | NA | NA |
| OTU475 | NA | NA | + | NA | NA | NA |
| OTU2029 | NA | NA | + | NA | NA | NA |
| OTU1332 | NA | NA | + | NA | NA | NA |
| OTU3462 | NA | NA | NA | + | NA | NA |
| OTU3004 | NA | NA | + | NA | NA | NA |
| OTU3987 | NA | NA | NA | NA | + | NA |
| OTU3001 | NA | NA | + | NA | NA | NA |
| OTU7050 | NA | NA | + | NA | NA | NA |
| OTU6027 | NA | + | NA | NA | NA | + |
| OTU2892 | NA | + | NA | NA | NA | NA |
| OTU7118 | NA | NA | NA | NA | NA | NA |
| OTU5486 | + | NA | NA | NA | NA | NA |
| OTU4347 | NA | NA | NA | NA | NA | NA |
| OTU3413 | NA | NA | NA | + | NA | NA |
| OTU7040 | NA | NA | + | NA | NA | NA |
| OTU3468 | NA | NA | NA | NA | NA | NA |
| OTU7782 | NA | NA | NA | + | NA | NA |
| OTU1207 | NA | NA | NA | NA | NA | NA |
| OTU3188 | NA | NA | + | NA | NA | NA |
| OTU4958 | NA | NA | NA | NA | + | NA |
| OTU7639 | NA | NA | + | NA | NA | NA |
| OTU19 | NA | NA | + | NA | NA | NA |
| OTU3382 | NA | NA | NA | + | NA | NA |
| OTU13 | NA | NA | NA | NA | + | NA |
| OTU1008 | NA | NA | + | NA | NA | NA |
| OTU5726 | NA | NA | NA | NA | NA | + |
| OTU5082 | NA | NA | NA | NA | NA | NA |
| OTU8005 | NA | NA | NA | NA | NA | + |
| OTU6279 | NA | + | NA | + | NA | NA |
| OTU6273 | NA | NA | NA | NA | NA | + |
| OTU6771 | NA | NA | NA | + | NA | NA |
| OTU7676 | NA | NA | NA | NA | NA | + |
| OTU4178 | NA | + | NA | NA | NA | NA |
| OTU3841 | NA | NA | NA | + | NA | NA |
| OTU4177 | NA | + | NA | NA | NA | NA |
| OTU7564 | NA | NA | NA | NA | NA | + |
| OTU2670 | NA | + | NA | NA | NA | NA |
| OTU7172 | NA | NA | + | NA | NA | NA |
| OTU2864 | NA | NA | NA | + | NA | NA |
| OTU420 | NA | NA | NA | NA | NA | NA |
| OTU3564 | NA | NA | + | NA | NA | NA |
| OTU1111 | NA | NA | + | NA | NA | NA |
| OTU1094 | NA | NA | + | NA | NA | NA |
| OTU7579 | NA | NA | NA | NA | NA | NA |
| OTU7046 | NA | NA | NA | NA | NA | NA |
| OTU7571 | NA | NA | NA | NA | NA | NA |
| OTU2202 | NA | NA | NA | NA | NA | NA |
| OTU5960 | NA | NA | NA | + | NA | NA |
| OTU1399 | NA | NA | + | NA | NA | NA |
| OTU6044 | NA | NA | NA | NA | NA | + |
| OTU5982 | NA | NA | + | NA | NA | NA |
| OTU1426 | + | NA | NA | NA | NA | NA |
| OTU1335 | NA | NA | + | NA | NA | NA |
| OTU5362 | NA | + | NA | NA | NA | NA |
| OTU5868 | NA | NA | NA | NA | NA | + |
| OTU5521 | NA | NA | NA | NA | + | NA |
| OTU4904 | + | NA | NA | NA | NA | NA |
| OTU3075 | NA | NA | NA | + | NA | NA |
| OTU4901 | NA | NA | NA | + | NA | NA |
| OTU3436 | NA | NA | NA | + | NA | NA |
| OTU3091 | NA | NA | NA | + | NA | NA |
| OTU6406 | NA | NA | NA | NA | NA | NA |
| OTU4668 | NA | NA | NA | NA | NA | + |
| OTU7037 | NA | NA | + | NA | NA | NA |
| OTU1398 | NA | + | NA | NA | NA | NA |
| OTU4234 | NA | + | NA | NA | NA | NA |
| OTU4232 | NA | NA | NA | NA | NA | NA |
| OTU4225 | NA | NA | NA | + | NA | NA |
| OTU650 | NA | NA | NA | + | NA | NA |
| OTU653 | NA | NA | + | NA | NA | NA |
| OTU4238 | NA | + | NA | NA | NA | NA |
| OTU3169 | NA | NA | NA | NA | NA | + |
| OTU3249 | NA | NA | NA | + | NA | NA |
| OTU1300 | + | NA | NA | NA | NA | NA |
| OTU7304 | NA | NA | + | NA | NA | NA |
| OTU649 | NA | NA | + | NA | NA | NA |
| OTU648 | NA | NA | + | NA | NA | NA |
| OTU4992 | NA | NA | NA | NA | + | NA |
| OTU5989 | NA | NA | + | NA | NA | NA |
| OTU644 | NA | NA | + | NA | NA | NA |
| OTU5980 | NA | NA | NA | + | NA | NA |
| OTU646 | NA | NA | + | NA | NA | NA |
| OTU7811 | NA | + | NA | NA | NA | NA |
| OTU8250 | NA | NA | + | NA | NA | NA |
| OTU4179 | NA | + | NA | NA | NA | NA |
| OTU6124 | NA | NA | + | NA | NA | NA |
| OTU5539 | + | NA | NA | NA | + | NA |
| OTU1627 | NA | NA | + | NA | NA | NA |
| OTU4912 | + | NA | NA | NA | NA | NA |
| OTU5186 | NA | NA | + | NA | NA | NA |
| OTU7106 | NA | + | NA | NA | NA | NA |
| OTU5529 | NA | NA | NA | + | NA | NA |
| OTU4788 | NA | NA | NA | NA | + | NA |
| OTU2104 | NA | NA | NA | NA | NA | NA |
| OTU7792 | NA | + | NA | NA | NA | + |
| OTU3417 | NA | NA | NA | + | NA | NA |
| OTU7094 | NA | NA | + | NA | NA | NA |
| OTU5780 | NA | NA | NA | NA | NA | NA |
| OTU1981 | NA | NA | + | NA | NA | NA |
| OTU6515 | NA | NA | NA | NA | NA | + |
| OTU6516 | NA | NA | NA | NA | NA | + |
| OTU5544 | NA | NA | NA | + | NA | NA |
| OTU5469 | + | NA | NA | NA | NA | NA |
| OTU3496 | NA | NA | NA | + | NA | NA |
| OTU3497 | NA | NA | NA | NA | NA | NA |
| OTU3494 | NA | NA | NA | + | NA | NA |
| OTU3492 | NA | NA | NA | NA | NA | NA |
| OTU3491 | NA | NA | NA | + | NA | NA |
| OTU5317 | NA | + | NA | NA | NA | NA |
| OTU510 | NA | NA | + | NA | NA | NA |
| OTU6926 | NA | NA | NA | NA | NA | NA |
| OTU2329 | NA | NA | NA | NA | + | NA |
| OTU7132 | NA | NA | + | NA | NA | NA |
| OTU851 | NA | NA | + | NA | NA | NA |
| OTU1433 | NA | NA | + | NA | NA | NA |
| OTU3470 | NA | NA | NA | + | NA | NA |
| OTU5128 | NA | NA | NA | NA | + | NA |
| OTU2808 | NA | NA | NA | + | NA | NA |
| OTU8034 | NA | NA | NA | + | NA | NA |
| OTU5243 | NA | NA | NA | NA | + | NA |
| OTU7101 | NA | NA | + | NA | NA | NA |
| OTU4002 | NA | NA | NA | NA | + | NA |
| OTU2093 | NA | NA | NA | NA | NA | NA |
| OTU2863 | NA | NA | NA | + | NA | NA |
| OTU2890 | NA | NA | NA | + | NA | NA |
| OTU5072 | NA | NA | NA | NA | NA | NA |
| OTU4419 | + | NA | NA | NA | NA | NA |
| OTU842 | NA | + | NA | NA | NA | NA |
| OTU6586 | NA | NA | NA | NA | + | NA |
| OTU4158 | NA | + | NA | NA | NA | NA |
| OTU3899 | NA | NA | NA | NA | NA | NA |
| OTU458 | NA | + | NA | NA | NA | NA |
| OTU5867 | NA | NA | NA | NA | NA | + |
| OTU4738 | NA | NA | NA | NA | + | NA |
| OTU4438 | + | NA | NA | NA | NA | NA |
| OTU4913 | + | NA | NA | NA | NA | NA |
| OTU4916 | NA | NA | + | NA | NA | NA |
| OTU4915 | NA | NA | NA | NA | + | NA |
| OTU3024 | NA | NA | + | NA | NA | NA |
| OTU7937 | NA | NA | + | NA | NA | NA |
| OTU6455 | + | NA | NA | NA | NA | NA |
| OTU4439 | NA | NA | NA | NA | + | NA |
| OTU770 | NA | NA | + | NA | NA | NA |
| OTU1624 | NA | NA | + | NA | NA | NA |
| OTU3414 | NA | NA | NA | + | NA | NA |
| OTU5801 | NA | NA | NA | NA | NA | + |
| OTU2191 | NA | NA | NA | NA | NA | + |
| OTU404 | + | NA | NA | NA | NA | NA |
| OTU4156 | NA | + | NA | NA | NA | NA |
| OTU4155 | NA | + | + | NA | NA | NA |
| OTU148 | NA | NA | + | NA | NA | NA |
| OTU4686 | NA | NA | NA | NA | NA | NA |
| OTU7038 | NA | NA | + | NA | NA | NA |
| OTU4549 | NA | NA | NA | NA | + | NA |
| OTU5141 | NA | NA | NA | NA | + | NA |
| OTU4609 | NA | NA | NA | NA | NA | NA |
| OTU3517 | NA | NA | NA | NA | NA | + |
| OTU2515 | NA | NA | NA | NA | + | NA |
| OTU2517 | + | NA | NA | NA | NA | NA |
| OTU2510 | NA | NA | + | NA | NA | NA |
| OTU2519 | NA | + | + | NA | + | NA |
| OTU2692 | NA | NA | NA | + | NA | NA |
| OTU273 | NA | NA | NA | + | NA | NA |
| OTU4927 | NA | + | NA | + | NA | NA |
| OTU6671 | + | NA | NA | NA | NA | NA |
| OTU2196 | NA | NA | NA | + | NA | NA |
| OTU7897 | NA | NA | NA | + | NA | NA |
| OTU6012 | + | NA | NA | NA | NA | NA |
| OTU7087 | NA | NA | + | NA | NA | NA |
| OTU5722 | NA | NA | NA | NA | + | NA |
| OTU7337 | NA | NA | NA | + | NA | NA |
| OTU5427 | NA | NA | NA | NA | + | NA |
| OTU6673 | NA | NA | + | NA | NA | + |
| OTU6793 | NA | NA | NA | + | NA | NA |
| OTU4977 | NA | NA | NA | NA | + | NA |
| OTU697 | NA | NA | NA | NA | NA | NA |
| OTU5275 | NA | NA | NA | NA | + | NA |
| OTU3546 | NA | NA | NA | + | NA | NA |
| OTU4212 | NA | + | NA | + | NA | NA |
| OTU4216 | NA | NA | NA | NA | NA | NA |
| OTU4322 | NA | + | NA | NA | NA | NA |
| OTU3442 | NA | NA | NA | + | NA | NA |
| OTU663 | NA | NA | + | NA | NA | NA |
| OTU667 | NA | NA | + | NA | NA | NA |
| OTU1693 | NA | NA | NA | + | NA | NA |
| OTU664 | NA | NA | + | NA | NA | NA |
| OTU668 | NA | NA | NA | NA | NA | NA |
| OTU640 | NA | NA | + | NA | NA | NA |
| OTU866 | NA | + | NA | NA | + | NA |
| OTU5615 | NA | + | NA | NA | NA | NA |
| OTU593 | + | NA | + | NA | NA | NA |
| OTU4555 | NA | + | + | NA | + | NA |
| OTU1130 | NA | NA | + | NA | NA | NA |
| OTU3646 | NA | NA | NA | NA | + | NA |
| OTU7316 | NA | NA | NA | + | NA | NA |
| OTU7035 | NA | NA | + | NA | NA | NA |
| OTU7032 | NA | NA | NA | NA | NA | NA |
| OTU6437 | NA | NA | + | NA | NA | NA |
| OTU3069 | NA | NA | NA | + | NA | NA |
| OTU3068 | NA | NA | NA | + | NA | NA |
| OTU2053 | NA | NA | + | NA | NA | NA |
| OTU2590 | NA | + | NA | NA | NA | NA |
| OTU5926 | NA | NA | NA | NA | NA | + |
| OTU2988 | NA | NA | NA | NA | NA | + |
| OTU399 | NA | NA | + | NA | NA | NA |
| OTU7620 | NA | NA | NA | NA | NA | + |
| OTU1618 | NA | NA | NA | + | NA | NA |
| OTU1233 | + | NA | NA | NA | NA | + |
| OTU4217 | NA | + | NA | NA | NA | NA |
| OTU5736 | NA | NA | NA | NA | NA | NA |
| OTU4331 | NA | NA | NA | + | NA | NA |
| OTU37 | NA | NA | NA | + | NA | NA |
| OTU4026 | NA | NA | + | NA | NA | NA |
| OTU128 | NA | NA | NA | NA | + | NA |
| OTU3087 | NA | NA | NA | + | NA | NA |
| OTU4350 | NA | NA | NA | NA | NA | NA |
| OTU2242 | NA | NA | NA | NA | + | NA |
| OTU2300 | + | NA | NA | NA | NA | NA |
| OTU4889 | + | NA | NA | NA | NA | NA |
| OTU2655 | NA | NA | NA | NA | NA | + |
| OTU6493 | + | NA | NA | NA | NA | NA |
| OTU3101 | NA | NA | NA | + | NA | NA |
| OTU7041 | NA | NA | + | NA | NA | NA |
| OTU2787 | NA | NA | NA | NA | NA | NA |
| OTU1730 | NA | NA | NA | + | NA | NA |
| OTU3440 | NA | + | NA | NA | NA | NA |
| OTU2993 | NA | NA | + | NA | NA | NA |
| OTU2992 | NA | NA | + | NA | NA | NA |
| OTU6768 | NA | NA | NA | NA | + | NA |
| OTU4867 | + | NA | NA | NA | NA | NA |
| OTU1854 | NA | NA | NA | NA | NA | NA |
| OTU6066 | NA | NA | NA | + | NA | NA |
| OTU8047 | NA | NA | NA | + | NA | NA |
| OTU5309 | NA | NA | NA | NA | NA | + |
| OTU1952 | NA | NA | NA | + | NA | NA |
| OTU2851 | NA | NA | NA | NA | + | NA |
| OTU6842 | NA | + | NA | NA | NA | NA |
| OTU785 | NA | NA | NA | NA | NA | NA |
| OTU50 | NA | NA | NA | NA | NA | NA |
| OTU4468 | + | NA | NA | NA | NA | NA |
| OTU8246 | NA | NA | + | NA | NA | NA |
| OTU3420 | NA | NA | NA | + | NA | NA |
| OTU7297 | NA | NA | + | NA | NA | NA |
| OTU829 | NA | NA | NA | NA | NA | + |
| OTU1725 | NA | + | NA | NA | NA | NA |
| OTU4143 | NA | + | NA | NA | NA | NA |
| OTU5541 | + | NA | NA | NA | NA | NA |
| OTU6891 | + | NA | NA | NA | NA | NA |
| OTU4735 | + | NA | NA | NA | NA | NA |
| OTU4973 | + | NA | NA | NA | NA | NA |
| OTU558 | NA | NA | + | NA | NA | NA |
| OTU4050 | NA | NA | NA | NA | + | NA |
| OTU7017 | NA | NA | + | NA | NA | NA |
| OTU6844 | NA | NA | NA | NA | NA | + |
| OTU861 | NA | NA | NA | + | NA | NA |
| OTU3212 | NA | NA | NA | + | NA | NA |
| OTU7772 | NA | NA | NA | + | NA | NA |
| OTU7955 | NA | NA | NA | NA | NA | + |
| OTU1790 | NA | NA | + | NA | NA | NA |
| OTU4459 | + | NA | NA | NA | NA | NA |
| OTU4456 | + | NA | NA | NA | NA | NA |
| OTU4457 | + | NA | NA | NA | NA | NA |
| OTU4450 | + | NA | NA | NA | NA | NA |
| OTU7047 | NA | NA | + | NA | NA | NA |
| OTU3774 | NA | + | NA | NA | NA | NA |
| OTU2888 | NA | NA | NA | + | NA | NA |
| OTU5428 | NA | NA | NA | + | NA | NA |
| OTU3045 | NA | NA | NA | + | NA | NA |
| OTU3919 | NA | NA | NA | NA | NA | + |
| OTU4882 | + | NA | NA | NA | NA | NA |
| OTU1306 | NA | NA | NA | NA | NA | NA |
| OTU4426 | NA | NA | NA | NA | + | NA |
| OTU7205 | NA | NA | + | NA | NA | NA |
| OTU4211 | NA | + | NA | NA | NA | NA |
| OTU4506 | NA | NA | NA | + | NA | NA |
| OTU3040 | NA | NA | + | NA | NA | NA |
| OTU2554 | NA | NA | NA | NA | NA | NA |
| OTU6530 | NA | NA | NA | + | NA | NA |
| OTU7801 | NA | NA | + | NA | NA | NA |
| OTU3136 | NA | NA | NA | NA | NA | + |
| OTU3498 | NA | NA | NA | + | NA | NA |
| OTU7164 | NA | NA | + | NA | NA | NA |
| OTU3472 | NA | NA | + | NA | NA | NA |
| OTU2820 | NA | + | NA | NA | + | NA |
| OTU2826 | NA | NA | NA | NA | + | NA |
| OTU4130 | + | NA | NA | NA | NA | NA |
| OTU575 | NA | NA | NA | NA | NA | NA |
| OTU578 | NA | NA | + | NA | NA | NA |
| OTU6115 | NA | NA | NA | NA | NA | + |
| OTU4185 | NA | + | NA | NA | NA | NA |
| OTU5889 | NA | NA | NA | NA | NA | + |
| OTU956 | NA | NA | + | NA | NA | NA |
| OTU4591 | NA | NA | NA | NA | NA | + |
| OTU2359 | NA | NA | + | NA | NA | NA |
| OTU6683 | NA | NA | NA | NA | NA | + |
| OTU2844 | NA | NA | NA | NA | NA | + |
| OTU4182 | NA | + | NA | NA | NA | NA |
| OTU5447 | + | NA | NA | NA | NA | NA |
| OTU1419 | NA | NA | NA | + | NA | NA |
| OTU5090 | NA | NA | NA | NA | NA | NA |
| OTU2353 | NA | NA | NA | + | NA | NA |
| OTU4718 | NA | NA | NA | NA | NA | NA |
| OTU5755 | NA | NA | NA | NA | NA | NA |
| OTU4462 | + | NA | NA | NA | NA | NA |
| OTU3781 | NA | NA | NA | NA | NA | NA |
| OTU4953 | NA | NA | NA | NA | + | NA |
| OTU7480 | NA | NA | NA | NA | NA | + |
| OTU4758 | NA | NA | NA | NA | + | NA |
| OTU4208 | NA | + | NA | NA | NA | NA |
| OTU7117 | NA | NA | + | NA | NA | NA |
| OTU6925 | NA | NA | NA | + | NA | NA |
| OTU7599 | + | NA | NA | NA | NA | NA |
| OTU1765 | NA | + | NA | NA | NA | NA |
| OTU2663 | NA | NA | NA | NA | NA | NA |
| OTU5895 | NA | NA | NA | NA | NA | + |
| OTU7346 | NA | NA | NA | NA | + | NA |
| OTU4399 | + | NA | NA | NA | NA | NA |
| OTU4962 | NA | NA | NA | NA | NA | NA |
| OTU935 | NA | NA | + | NA | NA | NA |
| OTU2429 | NA | NA | NA | + | NA | NA |
| OTU845 | NA | NA | NA | NA | NA | NA |
| OTU704 | NA | NA | NA | NA | NA | NA |
| OTU4798 | NA | + | NA | NA | NA | + |
| OTU3668 | NA | NA | NA | NA | NA | + |
| OTU4218 | NA | + | NA | NA | NA | NA |
| OTU596 | NA | NA | + | NA | NA | NA |
| OTU7018 | NA | NA | + | NA | NA | NA |
| OTU3041 | NA | NA | NA | + | NA | NA |
| OTU3609 | NA | NA | NA | + | NA | NA |
| OTU7014 | NA | NA | + | NA | NA | NA |
| OTU2637 | + | NA | NA | NA | NA | NA |
| OTU3707 | NA | NA | + | NA | NA | NA |
| OTU1801 | + | NA | NA | NA | NA | NA |
| OTU2945 | NA | NA | NA | NA | + | NA |
| OTU7243 | NA | + | NA | NA | NA | NA |
| OTU6491 | NA | NA | + | NA | NA | NA |
| OTU6490 | NA | NA | NA | NA | NA | + |
| OTU4327 | NA | NA | NA | + | NA | NA |
| OTU950 | NA | NA | NA | + | NA | NA |
| OTU6945 | NA | NA | NA | NA | NA | + |
| OTU3484 | NA | NA | NA | + | NA | NA |
| OTU3630 | NA | NA | NA | + | NA | NA |
| OTU2526 | NA | NA | NA | NA | NA | + |
| OTU8095 | + | NA | NA | NA | NA | NA |
| OTU5484 | + | NA | NA | NA | + | NA |
| OTU1006 | NA | NA | NA | + | NA | NA |
| OTU4864 | + | NA | NA | NA | NA | NA |
| OTU7114 | NA | NA | + | NA | NA | NA |
| OTU774 | NA | NA | NA | NA | NA | NA |
| OTU5254 | NA | NA | NA | NA | + | NA |
| OTU5704 | + | NA | NA | NA | NA | NA |
| OTU4742 | NA | NA | NA | NA | + | NA |
| OTU6234 | NA | NA | NA | NA | NA | + |
| OTU5631 | NA | NA | + | NA | NA | NA |
| OTU8166 | NA | NA | NA | NA | NA | NA |
| OTU4210 | NA | + | NA | NA | NA | NA |
| OTU4171 | NA | + | NA | NA | NA | NA |
| OTU5083 | NA | NA | NA | NA | NA | NA |
| OTU5080 | NA | + | NA | NA | NA | NA |
| OTU2316 | NA | NA | NA | NA | NA | NA |
| OTU5146 | NA | NA | NA | NA | + | NA |
| OTU5149 | NA | NA | NA | NA | + | NA |
| OTU8142 | + | NA | NA | NA | NA | NA |
| OTU1104 | NA | NA | NA | NA | NA | NA |
| OTU8206 | NA | NA | NA | NA | NA | + |
| OTU1671 | NA | NA | NA | NA | NA | NA |
| OTU7200 | + | NA | NA | NA | NA | NA |
| OTU3989 | + | NA | NA | NA | NA | NA |
| OTU2422 | NA | NA | NA | + | NA | NA |
| OTU171 | NA | NA | + | NA | NA | NA |
| OTU5851 | NA | NA | NA | NA | NA | + |
| OTU5877 | NA | NA | NA | NA | NA | + |
| OTU5322 | NA | NA | NA | + | NA | NA |
| OTU5320 | NA | + | NA | NA | NA | NA |
| OTU8039 | + | NA | NA | NA | NA | NA |
| OTU4273 | NA | NA | NA | + | NA | NA |
| OTU5094 | NA | NA | + | NA | NA | NA |
| OTU5091 | NA | NA | NA | + | NA | NA |
| OTU4636 | NA | NA | NA | NA | NA | NA |
| OTU4634 | NA | + | NA | NA | NA | NA |
| OTU7560 | NA | NA | NA | NA | NA | + |
| OTU3185 | NA | NA | + | NA | NA | NA |
| OTU3252 | NA | NA | NA | + | NA | NA |
| OTU51 | NA | + | NA | NA | NA | NA |
| OTU3353 | NA | NA | NA | NA | NA | + |
| OTU4894 | + | NA | NA | NA | NA | NA |
| OTU805 | NA | NA | + | NA | NA | NA |
| OTU3404 | NA | NA | + | NA | NA | NA |
| OTU1050 | NA | NA | + | NA | NA | NA |
| OTU1309 | NA | NA | NA | NA | NA | NA |
| OTU604 | NA | NA | + | NA | NA | NA |
| OTU8045 | NA | NA | NA | + | NA | NA |
| OTU3012 | NA | NA | + | NA | NA | NA |
| OTU4093 | NA | NA | NA | NA | + | NA |
| OTU4070 | + | NA | NA | NA | NA | NA |
| OTU5460 | + | NA | NA | NA | NA | NA |
| OTU7607 | NA | NA | + | NA | NA | NA |
| OTU6118 | NA | NA | + | NA | NA | NA |
| OTU4892 | + | NA | NA | NA | NA | NA |
| OTU4914 | + | NA | NA | NA | NA | NA |
| OTU720 | NA | + | NA | NA | NA | NA |
| OTU6555 | NA | NA | + | NA | NA | + |
| OTU7211 | NA | NA | + | NA | NA | NA |
| OTU6000 | NA | + | NA | NA | NA | NA |
| OTU601 | NA | NA | + | NA | NA | NA |
| OTU6559 | NA | NA | NA | NA | NA | + |
| OTU1915 | NA | NA | NA | NA | NA | NA |
| OTU5560 | NA | NA | NA | NA | NA | NA |
| OTU83 | NA | NA | + | NA | NA | NA |
| OTU2579 | + | NA | NA | NA | NA | NA |
| OTU3187 | NA | NA | + | NA | NA | NA |
| OTU43 | NA | NA | NA | NA | NA | + |
| OTU1777 | NA | NA | NA | + | NA | NA |
| OTU3432 | NA | NA | NA | NA | NA | NA |
| OTU2823 | NA | NA | NA | + | NA | + |
| OTU4008 | NA | NA | NA | NA | NA | + |
| OTU3921 | NA | NA | NA | + | NA | NA |
| OTU6102 | NA | NA | NA | NA | NA | NA |
| OTU5629 | NA | + | NA | NA | NA | NA |
| OTU382 | NA | NA | NA | + | NA | NA |
| OTU383 | NA | NA | NA | NA | NA | NA |
| OTU4782 | NA | NA | NA | NA | + | NA |
| OTU6788 | NA | + | NA | NA | NA | NA |
| OTU4519 | + | NA | NA | NA | NA | NA |
| OTU6353 | NA | NA | NA | NA | NA | + |
| OTU1588 | NA | + | NA | NA | NA | + |
| OTU5154 | + | NA | NA | NA | NA | NA |
| OTU1583 | NA | NA | + | NA | NA | NA |
| OTU3451 | NA | NA | NA | + | NA | NA |
| OTU5409 | + | NA | NA | NA | NA | NA |
| OTU555 | NA | NA | + | NA | NA | NA |
| OTU4409 | + | NA | NA | NA | NA | NA |
| OTU293 | + | + | NA | NA | NA | NA |
| OTU3704 | + | NA | NA | NA | + | NA |
| OTU7725 | NA | NA | NA | NA | NA | NA |
| OTU7063 | NA | NA | + | NA | NA | NA |
| OTU4348 | NA | NA | NA | NA | NA | NA |
| OTU7578 | NA | + | NA | NA | NA | NA |
| OTU4819 | NA | NA | NA | NA | + | NA |
| OTU569 | NA | NA | + | NA | NA | NA |
| OTU5622 | + | NA | NA | NA | NA | NA |
| OTU1666 | NA | NA | + | NA | NA | NA |
| OTU336 | NA | NA | NA | NA | NA | NA |
| OTU3452 | NA | NA | NA | + | NA | NA |
| OTU568 | NA | NA | + | NA | NA | NA |
| OTU27 | NA | NA | + | NA | NA | NA |
| OTU504 | NA | NA | NA | NA | + | NA |
| OTU5799 | NA | NA | NA | NA | NA | + |
| OTU3291 | NA | NA | NA | + | NA | NA |
| OTU5712 | + | NA | NA | NA | NA | NA |
| OTU508 | NA | NA | + | NA | NA | NA |
| OTU4950 | NA | NA | NA | NA | NA | + |
| OTU665 | NA | NA | + | NA | NA | NA |
| OTU4791 | NA | NA | NA | NA | + | NA |
| OTU4623 | NA | NA | NA | NA | NA | NA |
| OTU7755 | NA | NA | + | NA | NA | NA |
| OTU3985 | NA | NA | NA | + | NA | NA |
| OTU5634 | NA | NA | NA | + | NA | NA |
| OTU4841 | + | NA | NA | NA | NA | NA |
| OTU3370 | NA | NA | NA | + | NA | NA |
| OTU7206 | + | NA | NA | NA | NA | NA |
| OTU7151 | NA | NA | + | NA | NA | NA |
| OTU2434 | NA | + | NA | NA | NA | NA |
| OTU118 | NA | NA | NA | + | NA | NA |
| OTU2954 | NA | NA | NA | + | NA | NA |
| OTU1385 | NA | NA | + | NA | NA | NA |
| OTU7996 | NA | NA | NA | NA | NA | NA |
| OTU3000 | NA | NA | + | NA | NA | NA |
| OTU4789 | NA | NA | NA | NA | + | NA |
| OTU1360 | NA | NA | + | NA | NA | NA |
| OTU4786 | + | NA | NA | NA | NA | NA |
| OTU2352 | + | NA | NA | NA | NA | NA |
| OTU4112 | NA | NA | + | NA | NA | NA |
| OTU229 | NA | NA | NA | NA | + | NA |
| OTU6076 | NA | NA | NA | NA | NA | NA |
| OTU4833 | + | NA | NA | NA | NA | NA |
| OTU189 | NA | NA | + | NA | NA | NA |
| OTU7858 | NA | NA | NA | NA | NA | + |
| OTU7147 | NA | NA | + | NA | NA | NA |
| OTU3122 | NA | NA | + | NA | NA | + |
| OTU2461 | NA | NA | + | NA | NA | NA |
| OTU6972 | NA | NA | NA | NA | NA | + |
| OTU960 | NA | NA | NA | NA | + | NA |
| OTU2210 | NA | NA | + | NA | NA | NA |
| OTU597 | NA | NA | + | NA | NA | NA |
| OTU72 | NA | NA | NA | NA | NA | NA |
| OTU1471 | NA | NA | + | NA | NA | NA |
| OTU4090 | NA | NA | NA | NA | + | NA |
| OTU5461 | + | NA | NA | NA | NA | NA |
| OTU5465 | + | NA | NA | NA | NA | NA |
| OTU7975 | NA | NA | NA | NA | NA | NA |
| OTU6856 | NA | NA | NA | NA | NA | + |
| OTU6614 | NA | NA | NA | NA | NA | + |
| OTU73 | NA | NA | NA | NA | NA | NA |
| OTU76 | NA | NA | NA | + | NA | NA |
| OTU4881 | + | NA | NA | NA | NA | NA |
| OTU5713 | NA | NA | NA | NA | + | NA |
| OTU3814 | NA | NA | NA | NA | NA | NA |
| OTU4542 | NA | NA | NA | NA | + | NA |
| OTU4226 | NA | NA | NA | NA | NA | NA |
| OTU628 | NA | NA | + | NA | NA | NA |
| OTU624 | NA | NA | + | NA | NA | NA |
| OTU4797 | + | NA | NA | NA | + | NA |
| OTU3933 | NA | + | NA | NA | NA | NA |
| OTU2327 | NA | NA | NA | NA | NA | NA |
| OTU3365 | NA | NA | NA | + | NA | NA |
| OTU7771 | NA | NA | + | NA | NA | NA |
| OTU8041 | NA | NA | NA | NA | NA | NA |
| OTU5188 | NA | NA | NA | NA | NA | NA |
| OTU581 | NA | NA | + | NA | NA | NA |
| OTU4546 | NA | NA | NA | NA | + | NA |
| OTU4576 | NA | NA | NA | NA | NA | NA |
| OTU7568 | NA | NA | + | NA | NA | NA |
| OTU5216 | NA | NA | NA | + | NA | NA |
| OTU6960 | NA | NA | NA | NA | NA | NA |
| OTU971 | NA | NA | + | NA | NA | NA |
| OTU4821 | NA | NA | NA | NA | + | NA |
| OTU7216 | NA | NA | NA | + | NA | NA |
| OTU5401 | NA | NA | NA | NA | + | NA |
| OTU190 | NA | NA | NA | + | NA | NA |
| OTU68 | NA | NA | + | NA | NA | NA |
| OTU401 | NA | NA | + | NA | NA | NA |
| OTU3099 | NA | NA | NA | + | NA | NA |
| OTU4725 | NA | NA | NA | + | NA | NA |
| OTU4064 | NA | + | + | NA | NA | NA |
| OTU206 | NA | NA | + | NA | NA | NA |
| OTU1417 | NA | NA | NA | NA | NA | NA |
| OTU7079 | NA | NA | NA | NA | NA | NA |
| OTU3192 | NA | NA | NA | + | NA | NA |
| OTU8156 | NA | NA | NA | NA | NA | NA |
| OTU5451 | NA | NA | NA | NA | + | NA |
| OTU5641 | NA | NA | NA | NA | + | NA |
| OTU369 | NA | NA | NA | + | NA | NA |
| OTU2383 | NA | + | NA | NA | NA | NA |
| OTU366 | NA | NA | + | NA | NA | NA |
| OTU4870 | + | NA | NA | NA | NA | NA |
| OTU6239 | NA | NA | NA | NA | NA | NA |
| OTU3371 | NA | NA | NA | + | NA | NA |
| OTU6778 | NA | NA | NA | + | NA | NA |
| OTU3055 | NA | NA | NA | + | NA | NA |
| OTU1975 | NA | NA | + | NA | NA | NA |
| OTU6023 | NA | NA | + | NA | NA | NA |
| OTU1460 | NA | NA | NA | NA | NA | NA |
| OTU2314 | NA | NA | NA | + | NA | NA |
| OTU1808 | NA | NA | + | NA | NA | NA |
| OTU2405 | + | NA | NA | NA | NA | NA |
| OTU5698 | NA | NA | NA | NA | + | NA |
| OTU5872 | NA | NA | NA | NA | NA | + |
| OTU5876 | NA | NA | NA | NA | NA | + |
| OTU1396 | NA | NA | + | NA | NA | NA |
| OTU7222 | NA | NA | NA | NA | NA | + |
| OTU4614 | + | NA | NA | NA | NA | NA |
| OTU4612 | NA | + | NA | NA | NA | NA |
| OTU1066 | NA | NA | NA | NA | NA | NA |
| OTU4237 | NA | + | NA | NA | NA | NA |
| OTU3213 | NA | NA | NA | + | NA | NA |
| OTU3435 | NA | NA | + | NA | NA | NA |
| OTU3431 | + | NA | NA | NA | NA | NA |
| OTU840 | NA | NA | + | NA | NA | NA |
| OTU2133 | NA | + | NA | NA | NA | NA |
| OTU5435 | NA | NA | NA | NA | + | NA |
| OTU186 | NA | NA | NA | NA | NA | NA |
| OTU3763 | NA | NA | NA | NA | + | NA |
| OTU3033 | NA | NA | + | NA | NA | NA |
| OTU534 | NA | NA | + | NA | NA | NA |
| OTU4930 | NA | + | NA | NA | NA | NA |
| OTU4142 | NA | NA | NA | NA | NA | NA |
| OTU7422 | NA | NA | NA | NA | + | NA |
| OTU4494 | + | NA | NA | NA | NA | NA |
| OTU6084 | + | NA | NA | NA | NA | NA |
| OTU1369 | NA | NA | + | NA | NA | NA |
| OTU4492 | + | NA | NA | NA | NA | NA |
| OTU7224 | NA | NA | NA | + | NA | NA |
| OTU7220 | NA | NA | NA | NA | NA | NA |
| OTU4240 | NA | + | NA | NA | NA | NA |
| OTU2361 | NA | NA | NA | + | NA | NA |
| OTU4243 | NA | NA | NA | NA | NA | NA |
| OTU1208 | NA | NA | NA | NA | NA | + |
| OTU4087 | NA | NA | NA | NA | NA | NA |
| OTU563 | NA | NA | + | NA | NA | NA |
| OTU2614 | NA | NA | + | NA | NA | NA |
| OTU3284 | NA | + | NA | NA | NA | NA |
| OTU4393 | NA | NA | + | NA | NA | NA |
| OTU7682 | NA | NA | NA | NA | + | NA |
| OTU1493 | NA | NA | NA | NA | NA | NA |
| OTU6094 | NA | NA | NA | NA | NA | NA |
| OTU4853 | + | NA | NA | NA | NA | NA |
| OTU5719 | NA | NA | NA | NA | NA | NA |
| OTU6575 | NA | NA | NA | NA | NA | + |
| OTU8257 | NA | NA | + | NA | NA | NA |
| OTU5310 | NA | NA | + | NA | NA | NA |
| OTU3357 | NA | NA | NA | + | NA | NA |
| OTU3697 | NA | NA | NA | NA | + | NA |
| OTU2676 | NA | NA | + | NA | NA | NA |
| OTU1547 | NA | NA | + | NA | NA | NA |
| OTU1813 | NA | NA | + | NA | NA | NA |
| OTU2629 | + | NA | NA | NA | NA | NA |
| OTU6615 | NA | NA | NA | + | NA | NA |
| OTU4025 | NA | + | NA | NA | NA | NA |
| OTU7910 | NA | NA | NA | NA | NA | + |
| OTU4883 | + | NA | NA | NA | NA | NA |
| OTU5482 | + | NA | NA | NA | NA | NA |
| OTU5487 | + | NA | NA | NA | + | NA |
| OTU4558 | NA | NA | NA | NA | + | NA |
| OTU7059 | NA | NA | + | NA | NA | NA |
| OTU7668 | NA | NA | NA | NA | NA | + |
| OTU3978 | NA | + | NA | NA | NA | NA |
| OTU4432 | + | NA | NA | NA | NA | NA |
| OTU4420 | + | NA | NA | NA | NA | NA |
| OTU5717 | NA | NA | NA | NA | + | NA |
| OTU4877 | NA | NA | NA | NA | NA | NA |
| OTU3140 | + | NA | NA | NA | NA | NA |
| OTU1372 | NA | NA | NA | NA | NA | NA |
| OTU4251 | NA | NA | NA | + | NA | NA |
| OTU4845 | + | NA | NA | NA | NA | NA |
| OTU5260 | NA | + | NA | NA | NA | NA |
| OTU4084 | NA | NA | + | NA | NA | NA |
| OTU6967 | NA | NA | NA | NA | NA | + |
| OTU195 | NA | NA | + | NA | NA | NA |
| OTU732 | NA | + | NA | NA | NA | NA |
| OTU3485 | NA | NA | NA | + | NA | NA |
| OTU1904 | NA | NA | NA | NA | NA | NA |
| OTU4619 | NA | + | NA | NA | NA | NA |
| OTU365 | NA | NA | NA | + | NA | NA |
| OTU6903 | NA | NA | NA | NA | NA | + |
| OTU992 | NA | + | NA | NA | NA | NA |
| OTU995 | NA | + | NA | NA | NA | NA |
| OTU3938 | NA | NA | NA | NA | + | NA |
| OTU1848 | NA | NA | + | NA | NA | NA |
| OTU5660 | + | NA | NA | NA | NA | NA |
| OTU8102 | NA | NA | NA | NA | + | NA |
| OTU6011 | NA | NA | NA | NA | NA | NA |
| OTU5182 | + | NA | NA | NA | NA | NA |
| OTU2843 | NA | NA | NA | NA | + | NA |
| OTU4330 | NA | NA | NA | + | NA | NA |
| OTU1815 | NA | NA | + | NA | NA | NA |
| OTU6907 | NA | + | NA | NA | NA | NA |
| OTU1345 | NA | NA | NA | NA | NA | NA |
| OTU116 | + | NA | NA | + | NA | NA |
| OTU1440 | NA | NA | + | NA | NA | NA |
| OTU608 | NA | NA | + | NA | NA | NA |
| OTU6268 | NA | NA | + | NA | NA | NA |
| OTU3640 | NA | NA | + | NA | NA | NA |
| OTU4328 | NA | NA | NA | + | NA | NA |
| OTU1628 | NA | NA | + | NA | NA | NA |
| OTU489 | NA | + | NA | NA | NA | NA |
| OTU1828 | NA | + | NA | NA | NA | NA |
| OTU1620 | NA | NA | NA | NA | NA | NA |
| OTU4118 | NA | NA | NA | NA | + | NA |
| OTU2927 | NA | NA | + | NA | NA | NA |
| OTU3056 | + | NA | NA | NA | NA | NA |
| OTU5328 | NA | + | NA | NA | NA | NA |
| OTU4971 | NA | NA | NA | NA | NA | NA |
| OTU5819 | NA | NA | NA | NA | NA | NA |
| OTU1069 | NA | NA | NA | NA | + | NA |
| OTU4249 | NA | NA | NA | + | NA | NA |
| OTU5813 | NA | NA | NA | NA | NA | + |
| OTU411 | NA | NA | NA | + | NA | NA |
| OTU6488 | NA | + | NA | NA | NA | NA |
| OTU4679 | NA | + | NA | NA | NA | NA |
| OTU6483 | NA | NA | NA | NA | + | NA |
| OTU3415 | NA | NA | NA | + | NA | NA |
| OTU3207 | NA | + | NA | NA | NA | NA |
| OTU4437 | NA | NA | NA | NA | + | NA |
| OTU7176 | NA | NA | + | NA | NA | NA |
| OTU7541 | NA | + | NA | NA | NA | NA |
| OTU6639 | NA | NA | NA | NA | NA | NA |
| OTU5773 | + | NA | NA | NA | NA | NA |
| OTU5389 | NA | NA | + | NA | NA | NA |
| OTU7462 | NA | NA | NA | NA | NA | + |
| OTU6978 | NA | NA | NA | NA | NA | NA |
| OTU3377 | NA | NA | + | NA | NA | NA |
| OTU3378 | NA | NA | NA | + | NA | NA |
| OTU7171 | NA | NA | NA | NA | NA | NA |
| OTU3624 | NA | NA | NA | NA | + | NA |
| OTU4244 | NA | NA | NA | NA | NA | NA |
| OTU3165 | NA | NA | + | NA | NA | NA |
| OTU7179 | NA | NA | + | NA | NA | NA |
| OTU4351 | NA | NA | NA | + | NA | NA |
| OTU6017 | NA | NA | + | NA | NA | NA |
| OTU4793 | NA | NA | NA | NA | + | NA |
| OTU4997 | + | NA | NA | NA | NA | NA |
| OTU2754 | + | NA | NA | NA | NA | NA |
| OTU5697 | + | NA | NA | NA | NA | NA |
| OTU2008 | NA | NA | NA | NA | NA | + |
| OTU6997 | NA | NA | NA | NA | NA | + |
| OTU5499 | + | NA | NA | NA | NA | NA |
| OTU1734 | + | + | NA | NA | NA | NA |
| OTU201 | NA | NA | NA | NA | NA | NA |
| OTU203 | + | NA | + | NA | NA | NA |
| OTU2980 | NA | NA | + | NA | NA | NA |
| OTU2982 | NA | NA | + | NA | NA | NA |
| OTU2930 | NA | NA | + | NA | NA | NA |
| OTU1126 | NA | NA | NA | NA | NA | NA |
| OTU3180 | NA | NA | + | NA | NA | NA |
| OTU3911 | NA | NA | NA | NA | NA | NA |
| OTU7600 | NA | NA | NA | NA | NA | NA |
| OTU4490 | NA | NA | NA | NA | NA | NA |
| OTU3127 | NA | NA | NA | + | NA | NA |
| OTU344 | NA | NA | NA | + | NA | NA |
| OTU1846 | NA | + | NA | NA | NA | NA |
| OTU347 | NA | NA | NA | + | NA | NA |
| OTU6257 | NA | NA | NA | NA | NA | + |
| OTU1402 | NA | NA | + | NA | NA | NA |
| OTU3352 | NA | NA | NA | + | NA | NA |
| OTU5340 | NA | NA | NA | NA | NA | NA |
| OTU4153 | NA | + | NA | NA | NA | NA |
| OTU2084 | NA | NA | NA | NA | + | NA |
| OTU5426 | NA | NA | NA | NA | + | NA |
| OTU8137 | NA | NA | + | NA | NA | NA |
| OTU4444 | + | NA | NA | NA | NA | + |
| OTU1901 | NA | NA | + | NA | NA | NA |
| OTU4441 | NA | NA | NA | NA | NA | NA |
| OTU6445 | NA | NA | + | NA | NA | NA |
| OTU4245 | NA | NA | NA | + | NA | NA |
| OTU7538 | NA | NA | NA | NA | + | NA |
| OTU7863 | + | NA | NA | NA | NA | NA |
| OTU2870 | NA | NA | NA | + | NA | NA |
| OTU5624 | + | NA | NA | NA | NA | NA |
| OTU4966 | NA | NA | NA | NA | + | NA |
| OTU5365 | NA | NA | NA | NA | + | NA |
| OTU205 | NA | NA | + | NA | NA | NA |
| OTU869 | NA | NA | + | NA | NA | NA |
| OTU3418 | NA | NA | NA | + | NA | NA |
| OTU3416 | NA | NA | NA | NA | NA | NA |
| OTU3141 | NA | NA | NA | NA | NA | NA |
| OTU3412 | NA | NA | NA | + | NA | NA |
| OTU3410 | NA | NA | + | NA | NA | NA |
| OTU3421 | NA | NA | NA | + | NA | NA |
| OTU6794 | + | NA | NA | NA | NA | NA |
| OTU3094 | NA | NA | NA | NA | NA | NA |
| OTU7247 | NA | NA | NA | NA | NA | NA |
| OTU7713 | NA | NA | NA | NA | NA | NA |
| OTU5703 | + | NA | NA | NA | NA | NA |
| OTU5905 | NA | NA | NA | NA | NA | NA |
| OTU1549 | NA | NA | NA | NA | NA | NA |
| OTU8278 | NA | NA | + | NA | NA | NA |
| OTU961 | NA | NA | + | NA | NA | NA |
| OTU3026 | NA | NA | + | NA | NA | NA |
| OTU7131 | NA | NA | + | NA | NA | NA |
| OTU3604 | NA | NA | NA | + | NA | NA |
| OTU7139 | NA | NA | + | NA | NA | NA |
| OTU4121 | NA | + | NA | NA | NA | NA |
| OTU938 | NA | + | + | NA | NA | NA |
| OTU5398 | + | NA | NA | NA | NA | NA |
| OTU7192 | NA | NA | NA | + | NA | NA |
| OTU8230 | NA | NA | NA | NA | NA | NA |
| OTU2821 | NA | NA | NA | + | NA | NA |
| OTU3310 | NA | NA | NA | NA | NA | + |
| OTU5207 | NA | NA | NA | NA | + | NA |
| OTU2878 | NA | NA | NA | + | NA | NA |
| OTU3955 | NA | NA | + | NA | NA | NA |
| OTU5203 | NA | NA | NA | NA | + | NA |
| OTU523 | NA | NA | NA | NA | NA | NA |
| OTU4154 | NA | NA | + | NA | NA | NA |
| OTU7285 | NA | NA | NA | NA | NA | + |
| OTU2815 | NA | NA | + | NA | NA | NA |
| OTU4550 | NA | NA | NA | NA | + | NA |
| OTU3372 | NA | NA | NA | + | NA | NA |
| OTU6679 | NA | NA | NA | NA | NA | + |
| OTU941 | NA | NA | + | NA | NA | NA |
| OTU5462 | NA | NA | + | NA | NA | NA |
| OTU4626 | NA | + | NA | NA | NA | NA |
| OTU2740 | NA | NA | NA | NA | NA | NA |
| OTU2898 | + | NA | NA | NA | NA | NA |
| OTU5714 | NA | NA | NA | + | NA | NA |
| OTU2566 | NA | + | NA | NA | NA | NA |
| OTU6155 | NA | NA | + | NA | NA | NA |
| OTU4175 | NA | + | + | NA | NA | NA |
| OTU4204 | NA | + | NA | + | NA | NA |
| OTU2577 | NA | + | NA | NA | NA | NA |
| OTU6653 | + | NA | NA | NA | NA | NA |
| OTU457 | NA | NA | NA | NA | NA | NA |
| OTU4879 | NA | NA | NA | NA | + | NA |
| OTU4878 | NA | NA | + | NA | NA | NA |
| OTU4874 | NA | NA | NA | NA | NA | NA |
| OTU4384 | NA | NA | NA | NA | NA | NA |
| OTU4873 | + | NA | NA | NA | NA | NA |
| OTU3092 | NA | NA | NA | + | NA | NA |
| OTU3614 | NA | NA | + | NA | NA | NA |
| OTU360 | + | NA | NA | NA | NA | NA |
| OTU4630 | NA | NA | NA | NA | NA | NA |
| OTU5568 | NA | + | NA | NA | NA | NA |
| OTU3374 | NA | NA | + | NA | NA | NA |
| OTU3358 | NA | NA | NA | + | NA | NA |
| OTU7384 | NA | NA | NA | + | NA | NA |
| OTU6937 | NA | NA | NA | NA | NA | + |
| OTU2686 | NA | NA | NA | + | NA | NA |
| OTU398 | NA | NA | NA | NA | NA | NA |
| OTU4517 | NA | NA | NA | NA | + | NA |
| OTU4640 | + | NA | NA | NA | + | NA |
| OTU4512 | + | NA | NA | NA | NA | NA |
| OTU5385 | NA | NA | NA | NA | NA | NA |
| OTU2718 | NA | NA | NA | NA | NA | NA |
| OTU4124 | + | NA | NA | NA | NA | NA |
| OTU8179 | NA | + | NA | NA | NA | NA |
| OTU2017 | NA | + | NA | NA | NA | NA |
| OTU5894 | + | NA | NA | NA | NA | NA |
| OTU3391 | NA | NA | NA | NA | + | NA |
| OTU693 | NA | NA | NA | NA | NA | NA |
| OTU6709 | NA | NA | NA | NA | + | NA |
| OTU6745 | + | NA | NA | NA | NA | NA |
| OTU5277 | NA | NA | NA | NA | + | NA |
| OTU3447 | + | NA | NA | NA | NA | NA |
| OTU5849 | NA | NA | NA | NA | NA | + |
| OTU615 | NA | NA | + | NA | NA | NA |
| OTU4665 | NA | NA | + | NA | NA | NA |
| OTU7830 | NA | NA | NA | NA | NA | + |
| OTU2083 | + | NA | NA | NA | NA | NA |
| OTU6432 | NA | NA | NA | NA | NA | NA |
| OTU4667 | NA | NA | NA | + | NA | NA |
| OTU6388 | NA | NA | + | NA | NA | NA |
| OTU6557 | NA | NA | NA | NA | NA | NA |
| OTU4291 | NA | NA | NA | + | NA | NA |
| OTU7149 | NA | NA | + | NA | NA | NA |
| OTU5995 | NA | NA | NA | + | NA | NA |
| OTU2436 | NA | NA | + | NA | NA | NA |
| OTU4499 | + | NA | NA | NA | NA | NA |
| OTU386 | NA | NA | + | NA | NA | NA |
| OTU2782 | NA | NA | NA | NA | + | NA |
| OTU227 | NA | NA | NA | + | NA | NA |
| OTU7504 | NA | NA | NA | NA | NA | NA |
| OTU1134 | NA | NA | + | NA | NA | NA |
| OTU2388 | NA | NA | + | NA | NA | NA |
| OTU2449 | NA | NA | NA | NA | NA | NA |
| OTU2386 | NA | + | NA | + | NA | NA |
| OTU505 | NA | NA | + | NA | NA | NA |
| OTU4861 | + | NA | NA | NA | NA | NA |
| OTU5523 | NA | + | NA | NA | NA | NA |
| OTU322 | NA | NA | + | NA | NA | NA |
| OTU5526 | NA | NA | NA | NA | NA | + |
| OTU321 | NA | NA | NA | NA | NA | + |
| OTU7510 | NA | + | NA | NA | NA | NA |
| OTU8109 | NA | NA | NA | NA | NA | NA |
| OTU4855 | + | NA | NA | NA | NA | NA |
| OTU3339 | NA | NA | NA | + | NA | NA |
| OTU4324 | NA | NA | NA | + | NA | NA |
| OTU1841 | + | NA | NA | NA | NA | NA |
| OTU4294 | NA | NA | NA | + | NA | NA |
| OTU7120 | NA | NA | + | NA | NA | NA |
| OTU2116 | NA | NA | + | NA | NA | NA |
| OTU4389 | NA | NA | NA | NA | + | NA |
| OTU1091 | + | NA | NA | NA | NA | NA |
| OTU4872 | NA | NA | NA | NA | NA | NA |
| OTU898 | NA | NA | NA | + | NA | NA |
| OTU525 | NA | NA | + | NA | NA | NA |
| OTU527 | NA | NA | + | NA | NA | NA |
| OTU509 | NA | NA | + | NA | NA | NA |
| OTU4618 | NA | + | NA | NA | NA | NA |
| OTU5391 | + | NA | NA | NA | NA | NA |
| OTU2188 | + | NA | NA | NA | NA | NA |
| OTU8271 | NA | + | NA | NA | NA | NA |
| OTU5830 | NA | NA | NA | + | NA | NA |
| OTU4435 | + | NA | NA | NA | NA | NA |
| OTU4656 | + | NA | NA | NA | + | NA |
| OTU4128 | NA | NA | NA | NA | NA | NA |
| OTU586 | NA | NA | NA | NA | NA | NA |
| OTU4694 | NA | NA | + | NA | NA | NA |
| OTU2966 | NA | NA | + | NA | NA | NA |
| OTU5493 | + | NA | NA | NA | NA | NA |
| OTU4581 | NA | NA | NA | + | NA | NA |
| OTU202 | NA | NA | NA | + | NA | NA |
| OTU4552 | NA | NA | NA | NA | + | NA |
| OTU5692 | + | NA | NA | NA | NA | NA |
| OTU194 | NA | NA | + | NA | NA | NA |
| OTU1850 | NA | NA | NA | + | NA | NA |
| OTU2716 | NA | NA | NA | NA | NA | NA |
| OTU7262 | NA | NA | NA | + | NA | NA |
| OTU5450 | + | NA | NA | NA | NA | NA |
| OTU1286 | NA | NA | NA | NA | NA | NA |
| OTU4491 | NA | NA | NA | NA | NA | NA |
| OTU891 | NA | NA | NA | NA | NA | NA |
| OTU4400 | + | NA | NA | NA | NA | NA |
| OTU2347 | NA | NA | NA | NA | NA | NA |
| OTU4801 | NA | NA | NA | NA | + | NA |
| OTU1652 | NA | + | NA | NA | NA | NA |
| OTU5445 | NA | + | NA | NA | NA | NA |
| OTU4077 | NA | + | NA | NA | NA | NA |
| OTU5914 | NA | NA | NA | NA | NA | + |
| OTU144 | NA | NA | NA | + | NA | NA |
| OTU1654 | NA | NA | NA | + | NA | NA |
| OTU2832 | NA | NA | NA | NA | NA | NA |
| OTU7408 | NA | NA | NA | NA | NA | + |
| OTU5346 | NA | NA | NA | NA | NA | NA |
| OTU3796 | NA | NA | NA | + | NA | NA |
| OTU6440 | NA | NA | NA | NA | NA | NA |
| OTU2570 | NA | NA | NA | NA | + | NA |
| OTU4979 | NA | NA | NA | NA | NA | + |
| OTU6744 | NA | NA | NA | NA | NA | + |
| OTU6572 | + | NA | NA | NA | NA | NA |
| OTU6916 | NA | NA | NA | NA | NA | + |
| OTU5739 | NA | NA | NA | NA | + | NA |
| OTU5737 | NA | + | NA | NA | NA | NA |
| OTU5731 | NA | NA | NA | NA | NA | NA |
| OTU6796 | NA | NA | NA | + | NA | NA |
| OTU4812 | NA | NA | NA | NA | + | NA |
| OTU6039 | NA | NA | + | NA | NA | NA |
| OTU6336 | NA | NA | + | NA | NA | NA |
| OTU7020 | NA | NA | + | NA | NA | NA |
| OTU7311 | NA | NA | NA | NA | NA | + |
| OTU1619 | NA | NA | NA | NA | NA | + |
| OTU2781 | NA | NA | NA | + | NA | NA |
| OTU4847 | NA | NA | NA | NA | NA | NA |
| OTU6048 | NA | + | NA | NA | NA | NA |
| OTU1708 | + | NA | NA | NA | NA | NA |
| OTU2809 | NA | NA | NA | NA | NA | + |
| OTU730 | NA | NA | + | NA | NA | NA |
| OTU4566 | + | NA | NA | NA | NA | NA |
| OTU4522 | NA | NA | NA | + | NA | NA |
| OTU4653 | + | NA | NA | NA | NA | NA |
| OTU3835 | + | NA | NA | NA | NA | NA |
| OTU7048 | NA | NA | + | NA | NA | NA |
| OTU5628 | NA | NA | NA | NA | + | NA |
| OTU5843 | NA | NA | NA | NA | NA | + |
| OTU862 | NA | NA | NA | + | NA | NA |
| OTU4852 | + | NA | NA | NA | NA | NA |
| OTU1947 | NA | + | NA | NA | NA | NA |
| OTU4582 | NA | NA | NA | NA | + | NA |
| OTU7667 | NA | NA | NA | NA | + | NA |
| OTU4497 | + | NA | NA | NA | NA | NA |
| OTU6411 | NA | NA | NA | NA | NA | + |
| OTU4617 | NA | + | NA | NA | NA | NA |
| OTU627 | NA | NA | + | NA | NA | NA |
| OTU975 | NA | NA | + | NA | NA | NA |
| OTU5084 | NA | NA | + | NA | NA | NA |
| OTU6761 | + | NA | NA | NA | NA | NA |
| OTU7976 | + | NA | NA | NA | NA | NA |
| OTU5257 | NA | NA | NA | NA | + | NA |
| OTU422 | NA | NA | NA | + | NA | NA |
| OTU2990 | NA | NA | + | NA | NA | NA |
| OTU716 | NA | NA | NA | + | NA | NA |
| OTU3063 | NA | NA | NA | + | NA | NA |
| OTU7115 | NA | NA | + | NA | NA | NA |
| OTU933 | NA | NA | + | NA | NA | NA |
| OTU5267 | NA | NA | NA | NA | + | NA |
| OTU2005 | NA | NA | + | NA | NA | NA |
| OTU932 | + | NA | NA | NA | NA | NA |
| OTU4926 | + | NA | NA | NA | NA | NA |
| OTU4762 | NA | NA | NA | NA | + | NA |
| OTU2631 | + | NA | NA | NA | NA | NA |
| OTU2632 | + | NA | NA | NA | NA | NA |
| OTU776 | NA | NA | + | NA | NA | NA |
| OTU2227 | NA | NA | NA | NA | + | NA |
| OTU1776 | NA | NA | NA | NA | + | NA |
| OTU7102 | NA | NA | + | NA | NA | NA |
| OTU7108 | NA | NA | + | NA | NA | NA |
| OTU7875 | NA | NA | NA | NA | NA | NA |
| OTU1661 | NA | NA | NA | NA | NA | + |
| OTU5727 | + | + | NA | NA | NA | NA |
| OTU1180 | NA | NA | NA | + | NA | NA |
| OTU7127 | NA | NA | + | NA | NA | NA |
| OTU8130 | NA | NA | NA | NA | NA | NA |
| OTU7029 | NA | NA | + | NA | NA | NA |
| OTU2523 | NA | NA | NA | NA | NA | NA |
| OTU3110 | NA | NA | NA | + | NA | NA |
| OTU1357 | NA | NA | NA | + | NA | NA |
| OTU795 | NA | + | NA | NA | NA | NA |
| OTU1140 | NA | NA | NA | + | NA | NA |
| OTU4106 | NA | + | NA | NA | NA | NA |
| OTU4108 | NA | NA | NA | NA | + | NA |
| OTU6674 | NA | NA | NA | NA | NA | + |
| OTU3286 | NA | NA | NA | + | NA | NA |
| OTU4816 | + | NA | NA | NA | NA | NA |
| OTU4814 | NA | NA | NA | NA | + | NA |
| OTU4811 | NA | NA | NA | NA | + | NA |
| OTU7028 | NA | NA | + | NA | NA | NA |
| OTU2389 | NA | NA | NA | NA | NA | + |
| OTU2064 | NA | NA | + | NA | NA | NA |
| OTU3622 | NA | NA | NA | + | NA | NA |
| OTU139 | NA | + | NA | NA | NA | NA |
| OTU4750 | NA | NA | NA | NA | + | NA |
| OTU6032 | NA | NA | + | NA | NA | NA |
| OTU4570 | + | NA | NA | NA | NA | NA |
| OTU5594 | + | NA | NA | NA | NA | NA |
| OTU6474 | NA | NA | NA | + | NA | NA |
| OTU4893 | + | NA | NA | NA | NA | NA |
| OTU4071 | NA | NA | NA | NA | NA | NA |
| OTU1089 | NA | NA | + | NA | NA | NA |
| OTU5273 | NA | NA | NA | NA | + | NA |
| OTU2337 | NA | NA | NA | NA | NA | + |
| OTU4315 | NA | NA | NA | + | NA | NA |
| OTU1984 | NA | NA | NA | + | NA | NA |
| OTU1478 | NA | NA | NA | NA | + | NA |
| OTU6789 | NA | NA | NA | NA | NA | + |
| OTU8199 | NA | NA | NA | + | NA | NA |
| OTU4176 | NA | + | NA | NA | NA | NA |
| OTU3383 | NA | NA | NA | + | NA | + |
| OTU5834 | NA | NA | NA | NA | NA | + |
| OTU1281 | NA | + | NA | NA | NA | NA |
| OTU2411 | NA | NA | + | NA | NA | NA |
| OTU918 | NA | NA | NA | + | NA | NA |
| OTU363 | NA | NA | + | NA | NA | NA |
| OTU2414 | NA | NA | + | NA | NA | NA |
| OTU2416 | NA | NA | NA | NA | NA | + |
| OTU2320 | NA | NA | NA | NA | NA | + |
| OTU243 | NA | + | + | NA | NA | NA |
| OTU4846 | + | NA | NA | NA | NA | NA |
| OTU6509 | NA | NA | + | NA | NA | NA |
| OTU2641 | NA | NA | + | NA | NA | NA |
| OTU6847 | NA | NA | NA | + | NA | NA |
| OTU4514 | + | NA | NA | NA | NA | NA |
| OTU3454 | NA | NA | NA | + | NA | NA |
| OTU974 | NA | NA | + | NA | NA | NA |
| OTU6862 | NA | NA | NA | NA | NA | NA |
| OTU1444 | NA | NA | NA | + | NA | NA |
| OTU3475 | NA | NA | NA | + | NA | NA |
| OTU308 | NA | NA | + | NA | NA | NA |
| OTU1100 | NA | NA | + | NA | NA | NA |
| OTU3944 | NA | NA | NA | NA | NA | NA |
| OTU1886 | NA | NA | + | NA | NA | NA |
| OTU1997 | NA | + | NA | NA | NA | NA |
| OTU7170 | NA | NA | + | NA | NA | NA |
| OTU7733 | NA | NA | NA | NA | NA | + |
| OTU326 | NA | NA | + | NA | NA | NA |
| OTU6355 | NA | NA | NA | NA | + | NA |
| OTU4488 | + | NA | NA | NA | NA | NA |
| OTU1726 | NA | NA | + | NA | NA | NA |
| OTU1648 | NA | NA | + | NA | NA | NA |
| OTU1640 | NA | NA | NA | NA | + | NA |
| OTU6924 | NA | NA | NA | + | NA | + |
| OTU354 | NA | NA | + | NA | NA | NA |
| OTU3813 | NA | NA | + | NA | NA | NA |
| OTU7507 | NA | NA | NA | NA | NA | + |
| OTU7090 | NA | NA | + | NA | NA | NA |
| OTU4869 | NA | NA | NA | NA | NA | NA |
| OTU490 | NA | NA | NA | + | NA | NA |
| OTU7168 | NA | NA | + | NA | NA | NA |
| OTU3219 | NA | NA | NA | + | NA | NA |
| OTU305 | NA | NA | NA | NA | NA | NA |
| OTU5578 | + | NA | NA | NA | NA | NA |
| OTU8277 | NA | NA | + | NA | NA | + |
| OTU3106 | NA | NA | NA | + | NA | NA |
| OTU3100 | NA | NA | NA | + | NA | NA |
| OTU7424 | NA | + | NA | NA | NA | NA |
| OTU7918 | NA | + | NA | NA | NA | NA |
| OTU376 | NA | NA | + | NA | NA | NA |
| OTU2305 | NA | + | NA | NA | NA | NA |
| OTU2499 | NA | NA | NA | + | NA | NA |
| OTU3712 | NA | NA | + | NA | NA | NA |
| OTU1260 | NA | + | NA | NA | + | NA |
| OTU2453 | NA | NA | + | NA | NA | NA |
| OTU4466 | NA | NA | NA | NA | NA | NA |
| OTU1308 | NA | NA | NA | + | NA | NA |
| OTU4167 | NA | + | NA | NA | NA | NA |
| OTU5274 | NA | NA | NA | NA | + | NA |
| OTU837 | NA | NA | + | NA | NA | NA |
| OTU786 | NA | + | NA | NA | NA | NA |
| OTU3439 | NA | NA | NA | + | NA | NA |
| OTU784 | + | NA | NA | NA | NA | NA |
| OTU2244 | NA | NA | NA | + | NA | NA |
| OTU2991 | NA | NA | + | NA | NA | NA |
| OTU2995 | NA | NA | + | NA | NA | NA |
| OTU2997 | NA | NA | + | NA | NA | NA |
| OTU4998 | NA | NA | NA | NA | + | NA |
| OTU594 | NA | NA | + | NA | NA | NA |
| OTU423 | NA | NA | NA | NA | + | NA |
| OTU113 | NA | NA | + | NA | NA | NA |
| OTU1631 | NA | NA | NA | + | NA | NA |
| OTU4627 | NA | NA | NA | NA | + | NA |
| OTU3495 | NA | NA | NA | NA | + | NA |
| OTU701 | NA | NA | + | NA | NA | NA |
| OTU1941 | NA | NA | NA | NA | NA | NA |
| OTU982 | NA | NA | + | NA | NA | NA |
| OTU811 | NA | + | NA | NA | NA | NA |
| OTU3732 | NA | NA | NA | + | + | NA |
| OTU2199 | NA | NA | NA | + | NA | NA |
| OTU3423 | NA | NA | NA | + | NA | NA |
| OTU6093 | NA | + | NA | NA | NA | NA |
| OTU5513 | NA | NA | NA | + | NA | NA |
| OTU6082 | NA | NA | NA | NA | NA | + |
| OTU585 | NA | NA | + | NA | NA | NA |
| OTU587 | NA | NA | + | NA | NA | NA |
| OTU8119 | NA | NA | NA | NA | NA | NA |
| OTU5855 | NA | NA | NA | NA | NA | + |
| OTU6757 | NA | NA | + | NA | NA | NA |
| OTU4464 | + | NA | NA | NA | NA | NA |
| OTU7684 | NA | NA | NA | NA | NA | NA |
| OTU1083 | NA | NA | + | NA | NA | NA |
| OTU5754 | NA | NA | NA | NA | NA | NA |
| OTU6941 | NA | NA | NA | NA | + | NA |
| OTU5975 | NA | + | NA | NA | NA | NA |
| OTU3366 | NA | NA | NA | + | NA | NA |
| OTU5758 | + | NA | NA | NA | NA | NA |
| OTU5745 | NA | + | NA | NA | NA | + |
| OTU7467 | NA | NA | NA | NA | NA | NA |
| OTU7463 | + | NA | NA | NA | NA | NA |
| OTU5480 | NA | NA | NA | NA | NA | + |
| OTU1667 | NA | NA | + | NA | NA | NA |
| OTU5381 | NA | NA | NA | NA | NA | NA |
| OTU7233 | NA | NA | NA | NA | NA | NA |
| OTU6692 | NA | NA | NA | + | NA | NA |
| OTU6691 | NA | NA | + | NA | NA | NA |
| OTU6694 | NA | NA | NA | NA | NA | + |
| OTU4831 | + | NA | NA | NA | NA | NA |
| OTU4830 | + | NA | NA | NA | NA | NA |
| OTU4832 | + | NA | NA | NA | NA | NA |
| OTU4834 | + | NA | NA | NA | NA | NA |
| OTU729 | NA | NA | NA | NA | NA | NA |
| OTU3649 | NA | NA | NA | + | NA | NA |
| OTU5610 | NA | NA | NA | NA | NA | NA |
| OTU6377 | NA | NA | NA | NA | NA | + |
| OTU4780 | NA | NA | NA | NA | + | NA |
| OTU3683 | NA | + | NA | NA | NA | NA |
| OTU7239 | NA | NA | NA | NA | NA | + |
| OTU125 | NA | NA | + | NA | NA | NA |
| OTU4536 | + | NA | NA | NA | NA | NA |
| OTU2739 | NA | NA | NA | + | NA | NA |
| OTU7570 | NA | NA | NA | NA | NA | + |
| OTU2254 | + | NA | NA | NA | NA | NA |
| OTU1214 | NA | NA | NA | NA | NA | + |
| OTU5178 | + | NA | NA | NA | NA | NA |
| OTU1843 | NA | NA | + | NA | NA | NA |
| OTU5172 | + | NA | NA | NA | NA | NA |
| OTU3550 | NA | NA | NA | + | NA | NA |
| OTU6473 | NA | NA | NA | NA | NA | + |
| OTU256 | NA | NA | + | NA | NA | NA |
| OTU4888 | + | NA | NA | NA | NA | NA |
| OTU2737 | NA | NA | NA | NA | NA | NA |
| OTU4227 | NA | + | NA | NA | NA | NA |
| OTU2255 | NA | + | NA | NA | NA | NA |
| OTU3458 | NA | NA | NA | + | NA | NA |
| OTU265 | NA | NA | + | NA | NA | NA |
| OTU5318 | NA | NA | + | NA | NA | NA |
| OTU5319 | + | NA | NA | NA | NA | NA |
| OTU5294 | NA | NA | + | NA | NA | NA |
| OTU2639 | NA | NA | NA | NA | + | NA |
| OTU3014 | NA | NA | + | NA | NA | NA |
| OTU6517 | NA | NA | NA | NA | NA | + |
| OTU182 | NA | NA | NA | NA | NA | NA |
| OTU1222 | NA | NA | + | NA | NA | NA |
| OTU5120 | NA | NA | NA | NA | NA | NA |
| OTU6543 | NA | NA | + | NA | NA | NA |
| OTU660 | NA | NA | + | NA | + | NA |
| OTU5198 | + | NA | NA | NA | NA | NA |
| OTU5748 | NA | NA | NA | NA | + | NA |
| OTU3482 | NA | NA | NA | + | NA | NA |
| OTU2112 | + | NA | NA | NA | NA | NA |
| OTU7121 | NA | NA | + | NA | NA | NA |
| OTU7124 | NA | NA | + | NA | NA | NA |
| OTU7125 | NA | NA | + | NA | NA | NA |
| OTU1649 | NA | NA | NA | NA | NA | + |
| OTU7016 | NA | NA | + | NA | NA | NA |
| OTU1643 | NA | NA | NA | NA | + | NA |
| OTU1229 | + | NA | NA | NA | NA | NA |
| OTU5169 | NA | NA | NA | NA | + | NA |
| OTU4349 | NA | NA | NA | + | NA | NA |
| OTU8189 | NA | NA | NA | + | NA | NA |
| OTU4343 | + | NA | NA | NA | NA | NA |
| OTU4342 | NA | NA | NA | NA | NA | NA |
| OTU4346 | NA | NA | NA | + | NA | NA |
| OTU4345 | NA | + | NA | NA | NA | NA |
| OTU2694 | NA | NA | NA | NA | + | NA |
| OTU3873 | NA | NA | NA | + | NA | NA |
| OTU3020 | NA | NA | + | NA | NA | NA |
| OTU6810 | NA | NA | + | NA | NA | NA |
| OTU3027 | NA | NA | + | NA | NA | NA |
| OTU1275 | NA | NA | NA | NA | NA | + |
| OTU5024 | + | NA | NA | NA | NA | NA |
| OTU1539 | NA | NA | NA | + | NA | NA |
| OTU2185 | NA | NA | NA | NA | + | NA |
| OTU2021 | + | NA | NA | NA | NA | NA |
| OTU6373 | NA | NA | NA | + | NA | NA |
| OTU3050 | NA | NA | NA | + | NA | NA |
| OTU4164 | NA | + | NA | NA | NA | NA |
| OTU4163 | NA | + | NA | NA | NA | NA |
| OTU4690 | NA | NA | NA | NA | + | NA |
| OTU3996 | NA | NA | NA | NA | NA | NA |
| OTU4000 | + | NA | NA | NA | NA | NA |
| OTU3356 | NA | NA | NA | + | NA | NA |
| OTU7052 | NA | NA | + | NA | NA | NA |
| OTU7809 | NA | + | NA | NA | NA | NA |
| OTU4548 | NA | NA | NA | NA | NA | NA |
| OTU8207 | NA | NA | NA | NA | NA | NA |
| OTU6553 | NA | NA | NA | NA | NA | + |
| OTU6868 | NA | NA | NA | + | NA | NA |
| OTU8129 | NA | + | NA | NA | NA | NA |
| OTU1116 | NA | NA | NA | NA | + | NA |
| OTU853 | NA | + | NA | NA | NA | NA |
| OTU854 | NA | NA | NA | + | NA | NA |
| OTU2615 | NA | NA | NA | NA | + | NA |
| OTU3196 | NA | NA | + | NA | NA | NA |
| OTU3109 | NA | NA | NA | + | NA | NA |
| OTU4764 | NA | NA | NA | NA | + | NA |
| OTU4767 | NA | NA | NA | NA | + | NA |
| OTU4760 | NA | NA | NA | NA | + | NA |
| OTU4339 | NA | NA | NA | NA | NA | NA |
| OTU6645 | NA | + | NA | NA | NA | NA |
| OTU1849 | NA | NA | + | NA | NA | NA |
| OTU4574 | NA | + | NA | NA | NA | NA |
| OTU4606 | NA | NA | NA | NA | + | NA |
| OTU7966 | NA | NA | + | NA | NA | NA |
| OTU2516 | NA | NA | NA | + | NA | NA |
| OTU4319 | NA | NA | NA | + | NA | NA |
| OTU2507 | NA | + | NA | NA | NA | NA |
| OTU3674 | NA | NA | + | NA | NA | NA |
| OTU5360 | NA | NA | + | NA | NA | NA |
| OTU2034 | NA | NA | NA | NA | NA | + |
| OTU1007 | NA | NA | + | NA | NA | NA |
| OTU4473 | + | NA | NA | NA | NA | NA |
| OTU1799 | NA | NA | + | NA | NA | NA |
| OTU1960 | NA | NA | NA | NA | NA | NA |
| OTU4195 | NA | + | NA | NA | NA | NA |
| OTU6123 | NA | NA | + | NA | NA | NA |
| OTU1250 | NA | NA | + | NA | NA | NA |
| OTU3802 | NA | NA | NA | NA | NA | NA |
| OTU8126 | NA | NA | + | NA | NA | NA |
| OTU4329 | NA | NA | NA | + | NA | NA |
| OTU4563 | NA | + | NA | NA | NA | NA |
| OTU5246 | NA | NA | NA | NA | NA | NA |
| OTU5778 | NA | NA | NA | NA | NA | + |
| OTU1132 | NA | NA | + | NA | NA | NA |
| OTU5958 | NA | NA | NA | NA | NA | NA |
| OTU5776 | NA | + | NA | NA | NA | NA |
| OTU1973 | NA | NA | NA | NA | NA | NA |
| OTU6611 | NA | NA | NA | NA | NA | + |
| OTU4572 | NA | NA | NA | NA | + | NA |
| OTU4573 | NA | + | NA | NA | + | NA |
| OTU7448 | NA | NA | + | NA | NA | NA |
| OTU4575 | NA | + | NA | NA | NA | NA |
| OTU4578 | NA | NA | NA | NA | NA | NA |
| OTU4579 | NA | NA | NA | NA | + | NA |
| OTU4021 | NA | NA | + | NA | NA | NA |
| OTU833 | NA | NA | NA | NA | NA | NA |
| OTU5423 | + | NA | NA | NA | NA | NA |
| OTU4593 | NA | NA | NA | NA | + | NA |
| OTU2625 | NA | + | NA | NA | NA | NA |
| OTU5858 | NA | NA | NA | NA | NA | + |
| OTU4583 | NA | NA | NA | NA | + | NA |
| OTU1172 | NA | NA | + | NA | NA | NA |
| OTU651 | NA | NA | NA | NA | NA | NA |
| OTU652 | NA | NA | + | NA | NA | NA |
| OTU7998 | + | NA | NA | NA | NA | NA |
| OTU7990 | + | NA | NA | NA | NA | NA |
| OTU3874 | NA | NA | NA | + | NA | NA |
| OTU7433 | NA | NA | NA | NA | + | NA |
| OTU4766 | NA | NA | NA | NA | + | NA |
| OTU1167 | + | NA | NA | NA | + | NA |
| OTU7138 | NA | NA | + | NA | NA | NA |
| OTU3870 | NA | NA | NA | NA | NA | + |
| OTU7800 | NA | NA | NA | + | NA | NA |
| OTU4730 | NA | + | NA | NA | + | NA |
| OTU4125 | NA | NA | NA | + | NA | NA |
| OTU2378 | NA | NA | NA | + | NA | NA |
| OTU7364 | NA | NA | NA | NA | NA | NA |
| OTU5984 | NA | + | NA | NA | NA | NA |
| OTU6554 | NA | NA | NA | NA | NA | + |
| OTU7625 | + | NA | NA | + | NA | NA |
| OTU5308 | NA | NA | + | NA | NA | NA |
| OTU5585 | + | NA | NA | NA | NA | NA |
| OTU2713 | NA | NA | NA | + | NA | NA |
| OTU2711 | + | NA | NA | NA | NA | NA |
| OTU7752 | NA | + | NA | NA | NA | NA |
| OTU899 | NA | NA | + | NA | NA | NA |
| OTU4585 | NA | + | NA | NA | NA | NA |
| OTU3011 | NA | NA | NA | + | NA | NA |
| OTU8003 | NA | NA | NA | NA | NA | NA |
| OTU5635 | + | NA | NA | NA | NA | NA |
| OTU6324 | NA | NA | NA | + | NA | NA |
| OTU5241 | NA | NA | NA | NA | + | NA |
| OTU7588 | NA | + | NA | NA | NA | NA |
| OTU5570 | NA | NA | NA | NA | + | NA |
| OTU5574 | NA | NA | NA | NA | + | NA |
| OTU4297 | NA | NA | NA | + | NA | NA |
| OTU5187 | NA | + | NA | NA | NA | NA |
| OTU4403 | + | NA | NA | NA | NA | NA |
| OTU896 | NA | NA | + | NA | NA | NA |
| OTU1032 | NA | NA | NA | NA | NA | NA |
| OTU2891 | NA | NA | NA | + | NA | NA |
| OTU6501 | NA | NA | + | NA | NA | NA |
| OTU2179 | NA | NA | NA | + | NA | NA |
| OTU2209 | + | NA | NA | NA | NA | NA |
| OTU3521 | NA | NA | NA | NA | NA | + |
| OTU661 | NA | NA | + | NA | NA | NA |
| OTU680 | NA | NA | + | NA | NA | NA |
| OTU8050 | NA | NA | NA | NA | NA | + |
| OTU7141 | NA | NA | + | NA | NA | NA |
| OTU2673 | NA | NA | NA | + | NA | NA |
| OTU7148 | NA | NA | + | NA | NA | NA |
| OTU7144 | NA | NA | NA | NA | NA | NA |
| OTU7145 | NA | NA | + | NA | NA | NA |
| OTU3031 | NA | NA | NA | NA | NA | + |
| OTU5464 | + | NA | NA | NA | NA | NA |
| OTU3163 | NA | + | NA | NA | NA | NA |
| OTU5153 | + | NA | NA | NA | NA | NA |
| OTU3782 | NA | NA | NA | + | NA | NA |
| OTU1063 | NA | + | NA | NA | NA | NA |
| OTU3003 | + | NA | NA | NA | NA | NA |
| OTU876 | NA | NA | + | NA | NA | NA |
| OTU5891 | NA | NA | NA | NA | NA | + |
| OTU5892 | NA | NA | NA | NA | NA | + |
| OTU8151 | NA | NA | NA | + | NA | NA |
| OTU5263 | NA | NA | NA | NA | + | NA |
| OTU3030 | NA | NA | + | NA | NA | NA |
| OTU101 | NA | NA | NA | + | NA | NA |
| OTU2550 | NA | NA | NA | + | NA | NA |
| OTU6570 | NA | NA | NA | NA | NA | + |
| OTU2045 | NA | NA | NA | NA | NA | NA |
| OTU4704 | NA | + | NA | + | NA | NA |
| OTU3666 | NA | NA | NA | + | NA | NA |
| OTU5174 | + | NA | NA | NA | NA | NA |
| OTU4800 | NA | NA | NA | NA | + | NA |
| OTU7133 | NA | NA | + | NA | NA | NA |
| OTU5232 | NA | NA | NA | NA | + | NA |
| OTU355 | NA | NA | + | NA | NA | NA |
| OTU3290 | NA | NA | NA | NA | NA | NA |
| OTU2282 | NA | NA | NA | NA | + | NA |
| OTU583 | NA | NA | + | NA | NA | NA |
| OTU5792 | NA | NA | NA | + | NA | NA |
| OTU4896 | + | NA | NA | NA | NA | NA |
| OTU3476 | NA | NA | NA | + | NA | NA |
| OTU2734 | NA | NA | NA | + | NA | NA |
| OTU6206 | NA | NA | + | NA | NA | NA |
| OTU1057 | + | NA | NA | NA | NA | NA |
| OTU7128 | NA | NA | + | NA | NA | NA |
| OTU2714 | NA | NA | NA | + | NA | NA |
| OTU7622 | NA | NA | NA | NA | NA | + |
| OTU7623 | NA | NA | NA | + | NA | NA |
| OTU2018 | NA | NA | + | NA | NA | NA |
| OTU3449 | NA | NA | NA | + | NA | NA |
| OTU4141 | NA | + | NA | NA | NA | NA |
| OTU4144 | NA | NA | NA | + | NA | NA |
| OTU4147 | NA | + | NA | NA | NA | NA |
| OTU4149 | NA | + | NA | NA | NA | NA |
| OTU4148 | NA | + | NA | NA | NA | NA |
| OTU4829 | + | NA | NA | NA | NA | NA |
| OTU2575 | NA | NA | NA | NA | NA | NA |
| OTU6712 | NA | NA | NA | NA | NA | + |
| OTU3450 | NA | NA | NA | + | NA | NA |
| OTU289 | NA | NA | NA | NA | NA | NA |
| OTU498 | NA | NA | NA | NA | NA | NA |
| OTU4673 | NA | NA | + | NA | NA | NA |
| OTU284 | NA | NA | + | NA | NA | NA |
| OTU5138 | NA | NA | NA | NA | + | NA |
| OTU7161 | NA | NA | + | NA | NA | NA |
| OTU5133 | NA | NA | NA | NA | + | NA |
| OTU3150 | NA | NA | NA | NA | NA | NA |
| OTU3077 | NA | NA | NA | + | NA | NA |
| OTU809 | NA | NA | NA | NA | NA | NA |
| OTU2418 | NA | + | NA | NA | NA | NA |
| OTU6783 | NA | NA | NA | + | NA | NA |
| OTU5250 | NA | NA | NA | NA | + | NA |
| OTU1473 | NA | NA | NA | NA | NA | NA |
| OTU5230 | NA | NA | NA | NA | + | NA |
| OTU7787 | NA | NA | NA | NA | NA | NA |
| OTU940 | + | NA | NA | NA | NA | NA |
| OTU8060 | NA | NA | NA | NA | NA | + |
| OTU6150 | NA | + | NA | NA | NA | NA |
| OTU4835 | + | NA | NA | NA | NA | NA |
| OTU3981 | NA | NA | NA | NA | NA | NA |
| OTU2466 | NA | NA | NA | NA | NA | NA |
| OTU4520 | + | NA | NA | NA | NA | NA |
| OTU682 | NA | NA | + | NA | NA | NA |
| OTU5240 | NA | NA | NA | NA | + | NA |
| OTU299 | NA | NA | NA | NA | + | NA |
| OTU8276 | NA | NA | + | NA | NA | NA |
| OTU3508 | + | + | NA | + | NA | NA |
| OTU6970 | NA | NA | NA | NA | NA | + |
| OTU5361 | NA | NA | NA | NA | NA | NA |
| OTU2855 | NA | NA | NA | NA | NA | NA |
| OTU1519 | NA | NA | + | NA | NA | NA |
| OTU7683 | NA | NA | + | NA | NA | NA |
| OTU3490 | NA | NA | NA | + | NA | NA |
| OTU3151 | NA | + | NA | NA | NA | NA |
| OTU211 | NA | NA | + | NA | NA | NA |
| OTU7648 | NA | + | NA | NA | NA | NA |
| OTU6841 | NA | + | + | NA | NA | NA |
| OTU6848 | NA | + | NA | NA | NA | NA |
| OTU3235 | + | NA | NA | NA | NA | + |
| OTU5888 | NA | NA | NA | NA | NA | + |
| OTU4952 | + | NA | NA | NA | NA | NA |
| OTU97 | NA | NA | + | NA | NA | NA |
| OTU6795 | NA | NA | NA | NA | NA | + |
| OTU582 | NA | NA | NA | NA | NA | NA |
| OTU699 | NA | NA | NA | NA | + | NA |
| OTU8019 | NA | NA | NA | NA | NA | + |
| OTU5688 | NA | NA | NA | + | NA | NA |
| OTU567 | NA | NA | NA | NA | NA | NA |
| OTU543 | NA | NA | + | NA | NA | NA |
| OTU540 | NA | NA | + | NA | NA | NA |
| OTU541 | NA | + | + | NA | NA | NA |
| OTU547 | NA | NA | + | NA | NA | NA |
| OTU548 | NA | NA | NA | NA | NA | NA |
| OTU225 | NA | NA | + | NA | NA | NA |
| OTU2763 | NA | + | NA | NA | NA | NA |
| OTU3893 | NA | NA | NA | NA | + | NA |
| OTU1636 | NA | + | NA | NA | NA | NA |
| OTU1048 | NA | NA | + | NA | NA | NA |
| OTU1909 | NA | NA | NA | NA | NA | + |
| OTU996 | NA | NA | + | NA | NA | NA |
| OTU6096 | NA | NA | + | NA | NA | NA |
| OTU5121 | + | NA | NA | NA | NA | NA |
| OTU8091 | + | NA | NA | NA | NA | NA |
| OTU4562 | NA | NA | NA | NA | + | NA |
| OTU5129 | + | NA | NA | NA | NA | NA |
| OTU1236 | NA | NA | NA | NA | + | NA |
| OTU6346 | NA | NA | NA | NA | NA | + |
| OTU1231 | NA | NA | NA | NA | NA | + |
| OTU3302 | NA | NA | NA | + | + | NA |
| OTU6932 | NA | NA | NA | NA | NA | + |
| OTU4592 | NA | NA | NA | NA | + | NA |
| OTU7509 | NA | NA | NA | + | NA | + |
| OTU4556 | + | NA | NA | NA | NA | NA |
| OTU4188 | NA | + | NA | NA | NA | NA |
| OTU4478 | + | NA | NA | NA | NA | NA |
| OTU3121 | NA | + | NA | NA | NA | NA |
| OTU4166 | NA | + | NA | NA | NA | NA |
| OTU5552 | NA | NA | + | NA | NA | NA |
| OTU4300 | NA | NA | NA | + | NA | NA |
| OTU7013 | NA | NA | + | NA | NA | NA |
| OTU8222 | NA | NA | + | NA | NA | NA |
| OTU7077 | NA | NA | + | NA | NA | NA |
| OTU5854 | NA | NA | NA | NA | NA | + |
| OTU4266 | NA | NA | NA | + | NA | NA |
| OTU4802 | + | NA | NA | NA | NA | NA |
| OTU7309 | NA | + | NA | NA | NA | NA |
| OTU4705 | NA | + | NA | NA | NA | NA |
| OTU1968 | NA | NA | NA | + | NA | NA |
| OTU4554 | NA | NA | NA | NA | + | NA |
| OTU860 | NA | NA | NA | NA | NA | NA |
| OTU3038 | NA | NA | + | NA | NA | NA |
| OTU5242 | NA | NA | NA | NA | + | NA |
| OTU1074 | NA | NA | + | NA | NA | NA |
| OTU1311 | NA | NA | NA | NA | NA | NA |
| OTU214 | NA | NA | NA | NA | NA | NA |
| OTU7248 | + | NA | NA | NA | NA | NA |
| OTU4193 | NA | NA | + | NA | NA | NA |
| OTU4465 | + | NA | NA | NA | NA | NA |
| OTU3670 | NA | NA | NA | NA | + | NA |
| OTU7837 | NA | NA | NA | + | NA | NA |
| OTU7739 | NA | NA | NA | NA | NA | + |
| OTU7213 | NA | NA | + | NA | NA | NA |
| OTU4898 | + | NA | NA | NA | NA | NA |
| OTU1415 | NA | NA | + | NA | + | NA |
| OTU1579 | NA | NA | NA | + | NA | NA |
| OTU4174 | NA | + | NA | NA | NA | NA |
| OTU7934 | NA | NA | NA | + | NA | NA |
| OTU4317 | NA | NA | NA | + | NA | NA |
| OTU5497 | NA | + | NA | NA | NA | NA |
| OTU524 | NA | NA | + | NA | NA | NA |
| OTU3910 | NA | NA | NA | NA | + | NA |
| OTU2007 | NA | NA | NA | NA | NA | NA |
| OTU4895 | + | NA | NA | NA | NA | NA |
| OTU4336 | NA | NA | NA | + | NA | NA |
| OTU7534 | NA | NA | NA | + | NA | NA |
| OTU3051 | NA | NA | NA | + | NA | NA |
| OTU4541 | NA | NA | NA | NA | + | NA |
| OTU7767 | NA | NA | NA | + | NA | NA |
| OTU7814 | NA | NA | + | NA | NA | NA |
| OTU242 | NA | NA | NA | + | NA | NA |
| OTU7908 | NA | NA | NA | + | NA | NA |
| OTU7166 | NA | NA | + | NA | NA | NA |
| OTU7165 | NA | NA | NA | NA | NA | NA |
| OTU7160 | NA | NA | + | NA | NA | NA |
| OTU622 | NA | NA | + | NA | NA | NA |
| OTU7516 | NA | NA | NA | NA | + | NA |
| OTU819 | NA | NA | NA | + | NA | NA |
| OTU4826 | NA | NA | NA | NA | + | NA |
| OTU5890 | NA | NA | NA | NA | NA | + |
| OTU4303 | NA | NA | NA | + | NA | NA |
| OTU4302 | NA | NA | NA | + | NA | NA |
| OTU6091 | NA | NA | + | NA | NA | NA |
| OTU3067 | NA | NA | NA | NA | NA | NA |
| OTU5374 | NA | NA | NA | + | NA | NA |
| OTU733 | NA | NA | NA | + | NA | NA |
| OTU737 | NA | NA | NA | NA | NA | NA |
| OTU793 | NA | + | NA | NA | NA | NA |
| OTU5248 | NA | NA | NA | NA | + | NA |
| OTU3960 | NA | NA | NA | + | NA | NA |
| OTU2139 | NA | + | NA | NA | NA | NA |
| OTU1659 | NA | NA | + | NA | NA | NA |
| OTU3255 | + | NA | NA | NA | NA | NA |
| OTU4427 | + | NA | NA | NA | NA | NA |
| OTU4752 | NA | NA | NA | NA | + | NA |
| OTU5050 | NA | NA | NA | NA | + | NA |
| OTU6827 | NA | NA | NA | NA | NA | NA |
| OTU6821 | NA | + | NA | NA | NA | NA |
| OTU6791 | NA | NA | NA | + | NA | NA |
| OTU3078 | NA | NA | NA | + | NA | NA |
| OTU2795 | NA | NA | + | NA | NA | NA |
| OTU5916 | NA | NA | + | NA | NA | NA |
| OTU1898 | NA | NA | NA | + | NA | NA |
| OTU6367 | NA | NA | NA | NA | NA | + |
| OTU1033 | NA | NA | + | NA | NA | NA |
| OTU5735 | NA | + | NA | NA | NA | NA |
| OTU4911 | NA | NA | NA | + | NA | NA |
| OTU7295 | NA | NA | + | NA | NA | NA |
| OTU2880 | NA | + | + | NA | NA | NA |
| OTU5886 | NA | NA | NA | + | NA | NA |
| OTU7269 | NA | NA | NA | NA | NA | NA |
| OTU437 | NA | NA | + | NA | NA | NA |
| OTU1297 | NA | NA | NA | + | NA | NA |
| OTU8116 | NA | NA | NA | NA | NA | + |
| OTU349 | NA | NA | NA | NA | + | NA |
| OTU5235 | NA | NA | NA | NA | + | NA |
| OTU2126 | NA | + | NA | NA | NA | NA |
| OTU1219 | NA | NA | NA | NA | NA | NA |
| OTU5394 | NA | NA | + | NA | NA | NA |
| OTU1210 | NA | NA | NA | + | NA | NA |
| OTU1740 | NA | NA | NA | NA | NA | NA |
| OTU4422 | + | NA | NA | NA | NA | NA |
| OTU4528 | + | NA | NA | NA | NA | NA |
| OTU1142 | NA | NA | + | NA | NA | NA |
| OTU5911 | NA | + | NA | NA | NA | NA |
| OTU1935 | NA | NA | + | NA | NA | NA |
| OTU4539 | NA | NA | NA | NA | + | NA |
| OTU161 | NA | + | NA | NA | NA | NA |
| OTU4535 | + | NA | NA | NA | NA | NA |
| OTU4537 | + | NA | NA | NA | NA | NA |
| OTU4531 | + | NA | NA | NA | NA | NA |
| OTU6178 | NA | NA | NA | + | NA | NA |
| OTU966 | NA | NA | + | NA | NA | NA |
| OTU8114 | NA | NA | NA | NA | NA | NA |
| OTU6579 | + | NA | NA | NA | NA | NA |
| OTU3082 | NA | NA | NA | + | NA | NA |
| OTU561 | NA | NA | + | NA | NA | NA |
| OTU6991 | NA | NA | NA | NA | NA | NA |
| OTU7959 | NA | NA | NA | NA | NA | NA |
| OTU2231 | NA | NA | NA | + | NA | NA |
| OTU5302 | NA | NA | NA | NA | NA | + |
| OTU605 | NA | NA | NA | NA | NA | NA |
| OTU4948 | NA | NA | NA | NA | + | NA |
| OTU6739 | NA | + | NA | NA | NA | NA |
| OTU1767 | NA | NA | + | NA | NA | NA |
| OTU5222 | NA | NA | NA | NA | NA | NA |
| OTU3908 | NA | NA | NA | NA | + | NA |
| OTU4503 | + | NA | NA | NA | NA | NA |
| OTU3644 | NA | NA | + | NA | NA | NA |
| OTU4443 | + | NA | NA | NA | NA | NA |
| OTU5276 | NA | NA | NA | NA | + | NA |
| OTU5271 | NA | NA | NA | NA | + | NA |
| OTU2593 | NA | NA | NA | NA | + | NA |
| OTU8158 | NA | NA | + | NA | NA | NA |
| OTU2456 | + | NA | NA | NA | NA | NA |
| OTU151 | + | + | NA | NA | NA | NA |
| OTU7522 | NA | NA | NA | NA | + | NA |
| OTU3680 | + | NA | NA | + | NA | NA |
| OTU3112 | NA | NA | NA | + | NA | NA |
| OTU433 | NA | NA | NA | + | NA | NA |
| OTU2949 | NA | NA | + | NA | NA | NA |
| OTU6365 | NA | NA | NA | NA | NA | NA |
| OTU3918 | NA | NA | NA | NA | NA | NA |
| OTU5990 | NA | NA | + | NA | NA | NA |
| OTU7785 | NA | NA | NA | + | NA | NA |
| OTU1367 | NA | NA | NA | + | NA | NA |
| OTU6290 | NA | NA | NA | + | NA | NA |
| OTU2796 | NA | NA | NA | NA | NA | NA |
| OTU507 | NA | NA | + | NA | NA | NA |
| OTU2444 | NA | NA | NA | NA | NA | NA |
| OTU565 | NA | NA | + | NA | NA | NA |
| OTU562 | NA | NA | + | NA | NA | NA |
| OTU52 | NA | NA | NA | NA | NA | + |
| OTU4777 | NA | NA | NA | NA | + | NA |
| OTU7129 | NA | NA | NA | NA | NA | NA |
| OTU4092 | NA | NA | NA | NA | + | NA |
| OTU4321 | NA | NA | NA | + | NA | NA |
| OTU292 | NA | NA | NA | + | NA | NA |
| OTU7596 | NA | NA | NA | NA | NA | + |
| OTU3083 | NA | NA | NA | + | + | NA |
| OTU7055 | NA | NA | + | NA | NA | NA |
| OTU4969 | NA | NA | NA | NA | NA | NA |
| OTU343 | NA | NA | + | NA | NA | NA |
| OTU5701 | + | NA | NA | NA | NA | NA |
| OTU3825 | NA | NA | + | NA | NA | NA |
| OTU6343 | NA | NA | NA | NA | NA | NA |
| OTU1946 | NA | NA | + | NA | NA | NA |
| OTU7189 | NA | + | NA | NA | NA | NA |
| OTU7180 | NA | NA | + | NA | NA | NA |
| OTU7183 | NA | NA | + | NA | NA | NA |
| OTU5775 | NA | NA | NA | NA | NA | NA |
| OTU4980 | + | NA | NA | NA | NA | NA |
| OTU4320 | NA | NA | NA | + | NA | NA |
| OTU4323 | NA | NA | NA | + | NA | NA |
| OTU4145 | NA | + | NA | NA | NA | NA |
| OTU7109 | NA | NA | + | NA | NA | NA |
| OTU7983 | NA | NA | NA | NA | NA | + |
| OTU7985 | NA | NA | NA | NA | NA | + |
| OTU705 | NA | NA | + | NA | NA | NA |
| OTU710 | NA | NA | + | NA | NA | NA |
| OTU6728 | NA | NA | + | NA | NA | NA |
| OTU2376 | NA | NA | NA | + | NA | NA |
| OTU3598 | NA | NA | + | NA | NA | NA |
| OTU4241 | NA | NA | NA | + | NA | NA |
| OTU4247 | NA | NA | NA | + | NA | NA |
| OTU8042 | + | NA | NA | NA | NA | NA |
| OTU3243 | NA | + | NA | NA | NA | NA |
| OTU7525 | NA | + | NA | NA | NA | NA |
| OTU3043 | NA | NA | NA | + | NA | NA |
| OTU6581 | NA | NA | NA | NA | NA | + |
| OTU5865 | NA | NA | NA | NA | NA | + |
| OTU1612 | NA | NA | NA | NA | + | NA |
| OTU4518 | NA | NA | NA | NA | NA | NA |
| OTU671 | + | NA | NA | NA | NA | NA |
| OTU4666 | NA | + | NA | NA | NA | NA |
| OTU410 | NA | NA | NA | + | NA | NA |
| OTU3380 | NA | NA | NA | NA | NA | + |
| OTU2585 | NA | NA | NA | + | NA | NA |
| OTU2581 | NA | + | NA | NA | NA | NA |
| OTU7091 | NA | NA | NA | + | NA | NA |
| OTU7804 | NA | NA | NA | NA | NA | + |
| OTU6378 | + | NA | NA | + | NA | NA |
| OTU6276 | NA | + | NA | NA | NA | NA |
| OTU2163 | NA | NA | NA | + | + | NA |
| OTU2726 | NA | NA | + | NA | NA | NA |
| OTU2724 | + | NA | NA | NA | NA | NA |
| OTU5157 | NA | NA | NA | NA | + | NA |
| OTU3540 | NA | NA | + | NA | NA | NA |
| OTU2717 | NA | NA | NA | + | NA | NA |
| OTU4183 | NA | + | NA | NA | NA | NA |
| OTU3060 | NA | NA | NA | + | NA | NA |
| OTU6953 | NA | NA | NA | + | NA | NA |
| OTU3209 | + | NA | NA | NA | NA | NA |
| OTU7468 | NA | NA | NA | + | NA | NA |
| OTU4246 | NA | NA | NA | + | NA | NA |
| OTU4783 | NA | NA | NA | NA | + | NA |
| OTU3438 | NA | NA | NA | + | NA | NA |
| OTU4663 | NA | NA | NA | NA | NA | NA |
| OTU3405 | NA | NA | NA | + | NA | NA |
| OTU7310 | NA | NA | + | NA | NA | NA |
| OTU577 | NA | NA | + | NA | NA | NA |
| OTU659 | NA | NA | + | NA | NA | NA |
| OTU1003 | NA | NA | NA | NA | NA | + |
| OTU1002 | NA | NA | + | NA | NA | NA |
| OTU5056 | NA | NA | NA | NA | + | NA |
| OTU719 | NA | NA | NA | + | NA | NA |
| OTU3399 | NA | NA | NA | + | NA | NA |
| OTU5179 | NA | NA | NA | + | NA | NA |
| OTU6735 | NA | NA | NA | + | NA | NA |
| OTU4629 | + | NA | NA | NA | NA | NA |
| OTU2159 | NA | NA | NA | NA | + | NA |
| OTU6222 | NA | NA | NA | NA | NA | + |
| OTU6333 | NA | NA | NA | NA | + | NA |
| OTU6114 | NA | NA | NA | + | NA | NA |
| OTU5278 | NA | NA | NA | NA | + | NA |
| OTU909 | NA | + | NA | NA | NA | NA |
| OTU1554 | NA | NA | NA | NA | NA | NA |
| OTU6913 | NA | NA | NA | + | NA | NA |
| OTU635 | NA | NA | + | NA | NA | NA |
| OTU1684 | NA | NA | NA | NA | NA | NA |
| OTU3687 | + | NA | NA | NA | NA | NA |
| OTU3098 | NA | NA | NA | NA | NA | NA |
| OTU3722 | NA | NA | NA | NA | NA | NA |
| OTU3096 | NA | NA | NA | NA | + | NA |
| OTU3095 | NA | NA | NA | + | NA | NA |
| OTU5200 | NA | NA | NA | NA | + | NA |
| OTU232 | NA | + | NA | + | NA | NA |
| OTU2974 | NA | NA | + | NA | NA | NA |
| OTU4139 | NA | NA | NA | NA | NA | NA |
| OTU2118 | NA | NA | NA | NA | NA | NA |
| OTU3549 | NA | + | + | NA | NA | NA |
| OTU3543 | NA | NA | + | NA | NA | NA |
| OTU359 | NA | NA | NA | NA | NA | NA |
| OTU4281 | NA | NA | NA | + | NA | NA |
| OTU1774 | NA | NA | NA | NA | NA | NA |
| OTU3829 | NA | NA | NA | NA | NA | NA |
| OTU1178 | + | NA | NA | NA | NA | NA |
| OTU3793 | NA | NA | NA | NA | NA | NA |
| OTU4408 | + | NA | NA | NA | NA | NA |
| OTU5842 | NA | NA | NA | NA | NA | + |
| OTU6809 | NA | NA | NA | + | NA | NA |
| OTU6806 | NA | NA | NA | NA | NA | + |
| OTU6807 | NA | NA | + | NA | NA | NA |
| OTU6803 | NA | NA | NA | NA | NA | + |
| OTU7235 | NA | NA | NA | NA | NA | + |
| OTU159 | NA | NA | NA | + | NA | NA |
| OTU8211 | NA | NA | + | NA | NA | NA |
| OTU2264 | NA | NA | NA | NA | + | NA |
| OTU526 | NA | NA | NA | NA | NA | NA |
| OTU1194 | NA | NA | + | NA | NA | NA |
| OTU3167 | NA | NA | NA | NA | NA | NA |
| OTU502 | NA | NA | + | NA | NA | NA |
| OTU3877 | NA | NA | NA | + | NA | NA |
| OTU4907 | + | NA | NA | NA | NA | NA |
| OTU3677 | NA | NA | NA | + | NA | NA |
| OTU4214 | NA | + | NA | NA | NA | NA |
| OTU7663 | NA | NA | + | NA | NA | NA |
| OTU7212 | + | NA | NA | NA | NA | NA |
| OTU783 | NA | NA | + | NA | NA | NA |
| OTU804 | NA | NA | NA | + | NA | NA |
| OTU2913 | + | NA | NA | NA | NA | NA |
| OTU1604 | NA | NA | NA | + | NA | NA |
| OTU6479 | NA | NA | + | NA | NA | NA |
| OTU3198 | NA | NA | NA | NA | + | NA |
| OTU6558 | NA | NA | NA | NA | NA | + |
| OTU6408 | NA | NA | NA | NA | NA | NA |
| OTU7520 | NA | NA | NA | NA | NA | NA |
| OTU6697 | NA | NA | NA | NA | NA | NA |
| OTU6495 | NA | NA | NA | + | NA | NA |
| OTU2865 | NA | NA | NA | + | NA | NA |
| OTU3025 | NA | NA | + | NA | NA | NA |
| OTU3021 | NA | NA | + | NA | NA | NA |
| OTU84 | NA | NA | NA | NA | NA | + |
| OTU4553 | NA | NA | NA | NA | + | NA |
| OTU1101 | NA | NA | NA | NA | NA | + |
| OTU7306 | NA | NA | NA | NA | NA | NA |
| OTU8101 | NA | NA | + | NA | NA | NA |
| OTU2151 | NA | NA | NA | NA | + | NA |
| OTU1492 | NA | NA | NA | NA | NA | NA |
| OTU5985 | NA | NA | + | NA | NA | NA |
| OTU4516 | + | NA | NA | NA | NA | NA |
| OTU4515 | + | NA | NA | NA | NA | NA |
| OTU4510 | + | NA | NA | NA | NA | NA |
| OTU260 | NA | NA | NA | + | NA | NA |
| OTU4595 | + | NA | NA | NA | + | NA |
| OTU4534 | NA | NA | NA | NA | NA | NA |
| OTU1413 | + | NA | NA | NA | NA | NA |
| OTU1021 | NA | NA | NA | NA | NA | NA |
| OTU3224 | + | NA | NA | NA | NA | NA |
| OTU5924 | NA | NA | NA | NA | + | NA |
| OTU6468 | NA | NA | NA | + | NA | NA |
| OTU4207 | NA | + | NA | NA | NA | NA |
| OTU7438 | NA | + | NA | NA | NA | NA |
| OTU7431 | NA | NA | NA | + | NA | NA |
| OTU4527 | + | NA | NA | NA | NA | NA |
| OTU3394 | NA | NA | NA | + | NA | NA |
| OTU2364 | NA | NA | + | NA | NA | NA |
| OTU5472 | + | NA | NA | NA | NA | NA |
| OTU7912 | + | NA | NA | NA | NA | NA |
| OTU2623 | NA | + | NA | NA | NA | NA |
| OTU5436 | + | NA | NA | NA | NA | + |
| OTU788 | + | NA | NA | NA | NA | NA |
| OTU4635 | NA | + | NA | NA | NA | NA |
| OTU7447 | NA | NA | NA | + | NA | NA |
| OTU178 | NA | NA | + | + | NA | NA |
| OTU8233 | NA | NA | + | NA | NA | NA |
| OTU170 | + | NA | NA | NA | + | NA |
| OTU8241 | NA | NA | + | NA | NA | NA |
| OTU3525 | NA | NA | NA | NA | NA | NA |
| OTU7430 | NA | + | NA | NA | NA | NA |
| OTU2961 | NA | NA | + | NA | NA | NA |
| OTU3875 | NA | + | NA | NA | NA | NA |
| OTU3048 | NA | NA | NA | + | NA | NA |
| OTU497 | NA | NA | NA | NA | NA | NA |
| OTU3360 | NA | NA | NA | NA | NA | NA |
| OTU8013 | NA | NA | NA | NA | NA | NA |
| OTU520 | NA | NA | + | NA | NA | NA |
| OTU1107 | NA | NA | NA | + | NA | NA |
| OTU2984 | NA | NA | + | NA | NA | NA |
| OTU1535 | NA | NA | NA | NA | NA | NA |
| OTU1020 | NA | NA | + | NA | + | NA |
| OTU1022 | NA | NA | NA | NA | NA | + |
| OTU5077 | NA | NA | + | NA | NA | NA |
| OTU7491 | NA | NA | NA | NA | NA | + |
| OTU5078 | NA | NA | NA | + | NA | NA |
| OTU2598 | NA | + | NA | NA | + | NA |
| OTU7724 | + | NA | NA | NA | NA | NA |
| OTU1438 | NA | NA | NA | NA | NA | NA |
| OTU5054 | NA | + | NA | NA | NA | NA |
| OTU6758 | NA | NA | NA | NA | NA | NA |
| OTU7686 | NA | NA | NA | NA | NA | + |
| OTU187 | NA | NA | NA | + | NA | NA |
| OTU6129 | NA | NA | + | NA | NA | NA |
| OTU2142 | NA | + | NA | NA | NA | NA |
| OTU513 | NA | NA | + | NA | NA | NA |
| OTU981 | NA | NA | NA | NA | NA | NA |
| OTU8011 | NA | NA | + | NA | NA | NA |
| OTU8010 | NA | NA | NA | NA | NA | NA |
| OTU1331 | NA | NA | NA | NA | NA | NA |
| OTU775 | NA | NA | NA | NA | NA | + |
| OTU2653 | NA | NA | NA | NA | + | NA |
| OTU551 | NA | NA | + | NA | NA | NA |
| OTU8237 | NA | NA | + | NA | NA | NA |
| OTU8235 | NA | NA | + | NA | NA | NA |
| OTU5893 | NA | NA | NA | NA | NA | + |
| OTU4429 | NA | NA | NA | NA | NA | NA |
| OTU654 | NA | NA | + | NA | NA | NA |
| OTU6751 | NA | NA | NA | NA | NA | + |
| OTU8163 | NA | NA | + | NA | NA | NA |
| OTU6825 | NA | NA | NA | NA | NA | NA |
| OTU1446 | NA | NA | + | NA | NA | NA |
| OTU2487 | NA | NA | NA | NA | NA | NA |
| OTU1857 | NA | NA | + | NA | NA | NA |
| OTU3693 | + | NA | NA | NA | + | NA |
| OTU5279 | NA | NA | + | NA | NA | NA |
| OTU1454 | NA | NA | + | NA | NA | NA |
| OTU630 | NA | NA | + | NA | NA | NA |
| OTU5253 | NA | NA | NA | NA | + | NA |
| OTU5151 | NA | NA | NA | + | NA | NA |
| OTU5258 | NA | NA | NA | NA | + | NA |
| OTU621 | NA | NA | + | NA | NA | NA |
| OTU1625 | NA | + | NA | NA | NA | NA |
| OTU6861 | + | NA | NA | NA | NA | NA |
| OTU2592 | NA | NA | + | NA | NA | NA |
| OTU5013 | + | NA | NA | NA | NA | NA |
| OTU1991 | NA | NA | NA | NA | + | NA |
| OTU3455 | NA | NA | NA | + | NA | NA |
| OTU7635 | NA | NA | NA | NA | NA | + |
| OTU599 | NA | NA | NA | NA | NA | NA |
| OTU5673 | NA | + | NA | NA | NA | NA |
| OTU1702 | NA | NA | NA | + | NA | NA |
| OTU4749 | NA | NA | NA | NA | + | NA |
| OTU2645 | NA | NA | + | NA | + | NA |
| OTU1709 | NA | NA | + | NA | NA | NA |
| OTU235 | + | NA | NA | NA | NA | NA |
| OTU4286 | NA | NA | NA | NA | NA | + |
| OTU4284 | NA | NA | NA | + | NA | NA |
| OTU4288 | NA | NA | NA | + | NA | NA |
| OTU4289 | NA | NA | NA | + | NA | NA |
| OTU6381 | NA | NA | NA | NA | NA | + |
| OTU755 | NA | NA | + | NA | NA | NA |
| OTU696 | NA | NA | + | NA | NA | NA |
| OTU694 | NA | NA | + | NA | NA | NA |
| OTU695 | NA | NA | + | NA | NA | NA |
| OTU690 | NA | NA | + | NA | NA | NA |
| OTU691 | NA | NA | + | NA | NA | NA |
| OTU1879 | NA | NA | + | NA | NA | NA |
| OTU6958 | NA | NA | NA | NA | NA | NA |
| OTU4062 | NA | NA | NA | NA | + | NA |
| OTU6139 | NA | NA | NA | NA | NA | + |
| OTU8155 | NA | NA | + | NA | NA | NA |
| OTU7083 | NA | NA | + | NA | NA | NA |
| OTU7082 | NA | NA | + | NA | NA | NA |
| OTU3706 | NA | NA | NA | + | NA | NA |
| OTU4428 | + | NA | NA | NA | NA | NA |
| OTU7086 | NA | NA | + | NA | NA | + |
| OTU8136 | NA | NA | NA | NA | + | NA |
| OTU5025 | NA | NA | NA | NA | NA | NA |
| OTU1491 | NA | NA | + | NA | NA | NA |
| OTU8187 | NA | NA | + | NA | NA | NA |
| OTU2012 | NA | NA | + | NA | NA | NA |
| OTU3949 | + | NA | NA | NA | NA | NA |
| OTU6705 | NA | NA | NA | NA | NA | + |
| OTU5001 | NA | NA | NA | NA | NA | NA |
| OTU5508 | NA | NA | NA | NA | + | NA |
| OTU3713 | NA | + | NA | + | NA | NA |
| OTU777 | NA | NA | + | NA | NA | NA |
| OTU3325 | NA | + | NA | NA | NA | NA |
| OTU3537 | NA | NA | NA | NA | NA | NA |
| OTU7626 | NA | NA | NA | NA | NA | NA |
| OTU5878 | NA | NA | NA | NA | NA | + |
| OTU3445 | NA | NA | NA | NA | NA | + |
| OTU5977 | NA | + | NA | NA | NA | NA |
| OTU1075 | NA | NA | NA | + | NA | NA |
| OTU7451 | NA | NA | NA | + | NA | NA |
| OTU7503 | + | NA | NA | NA | NA | NA |
| OTU610 | NA | NA | + | NA | NA | NA |
| OTU1518 | + | NA | NA | NA | NA | NA |
| OTU1041 | NA | + | NA | NA | NA | NA |
| OTU4411 | + | NA | NA | NA | NA | NA |
| OTU5476 | + | NA | NA | NA | NA | NA |
| OTU928 | NA | NA | NA | + | NA | NA |
| OTU2223 | NA | NA | NA | + | NA | NA |
| OTU625 | NA | NA | + | NA | NA | NA |
| OTU539 | NA | NA | + | NA | NA | NA |
| OTU7265 | NA | NA | + | NA | NA | NA |
| OTU522 | NA | NA | + | NA | NA | NA |
| OTU3797 | NA | NA | NA | NA | NA | NA |
| OTU529 | NA | NA | + | NA | NA | NA |
| OTU3471 | NA | NA | NA | + | NA | NA |
| OTU4974 | + | NA | NA | NA | NA | NA |
| OTU4699 | NA | NA | NA | + | NA | NA |
| OTU5921 | NA | NA | NA | NA | NA | + |
| OTU839 | NA | NA | NA | NA | + | NA |
| OTU6461 | NA | NA | NA | NA | NA | + |
| OTU620 | NA | NA | + | NA | NA | NA |
| OTU7574 | NA | NA | NA | NA | + | NA |
| OTU7078 | NA | NA | + | NA | NA | NA |
| OTU1694 | NA | NA | + | NA | NA | NA |
| OTU3486 | NA | NA | NA | + | NA | NA |
| OTU4001 | + | NA | NA | NA | NA | NA |
| OTU4610 | NA | + | NA | NA | NA | NA |
| OTU721 | NA | NA | NA | NA | NA | NA |
| OTU2998 | NA | NA | NA | NA | NA | NA |
| OTU8205 | NA | NA | NA | NA | NA | NA |
| OTU2463 | + | NA | NA | NA | NA | NA |
| OTU2464 | NA | NA | + | NA | NA | NA |
| OTU7199 | NA | NA | NA | NA | NA | + |
| OTU6737 | NA | NA | NA | + | NA | NA |
| OTU4397 | NA | + | NA | NA | NA | NA |
| OTU3733 | NA | + | NA | NA | NA | NA |
| OTU6414 | NA | NA | NA | NA | NA | + |
| OTU2874 | NA | NA | NA | NA | NA | + |
| OTU2707 | NA | NA | NA | NA | NA | NA |
| OTU3594 | NA | NA | + | NA | NA | NA |
| OTU7653 | NA | NA | + | NA | NA | NA |
| OTU4224 | NA | + | NA | NA | NA | NA |
| OTU3912 | + | NA | NA | NA | NA | NA |
| OTU5494 | + | NA | NA | NA | NA | NA |
| OTU4897 | + | NA | NA | NA | NA | NA |
| OTU3556 | NA | NA | + | NA | NA | NA |
| OTU4477 | + | NA | NA | NA | NA | NA |
| OTU82 | NA | NA | NA | NA | NA | NA |
| OTU1051 | NA | NA | NA | NA | NA | NA |
| OTU6985 | NA | NA | + | NA | NA | NA |
| OTU4283 | NA | NA | NA | + | NA | NA |
| OTU993 | NA | NA | + | NA | NA | NA |
| OTU4884 | + | NA | NA | NA | NA | NA |
| OTU773 | NA | NA | NA | NA | NA | NA |
| OTU4840 | + | NA | NA | NA | NA | NA |
| OTU8037 | NA | NA | NA | NA | NA | NA |
| OTU7418 | NA | NA | NA | NA | + | NA |
| OTU647 | NA | NA | NA | NA | + | NA |
| OTU4027 | NA | NA | NA | NA | NA | NA |
| OTU2838 | NA | NA | NA | NA | + | NA |
| OTU3053 | NA | NA | NA | NA | NA | NA |
| OTU2542 | NA | NA | NA | + | NA | NA |
| OTU5740 | NA | + | NA | NA | NA | NA |
| OTU4687 | NA | NA | NA | NA | NA | NA |
| OTU470 | NA | NA | + | NA | NA | NA |
| OTU5034 | + | NA | NA | NA | NA | NA |
| OTU474 | NA | NA | + | NA | NA | NA |
| OTU2895 | NA | NA | NA | + | NA | NA |
| OTU183 | NA | NA | NA | + | NA | NA |
| OTU4483 | + | NA | NA | NA | NA | NA |
| OTU6285 | NA | NA | NA | NA | + | NA |
| OTU1206 | NA | NA | + | NA | NA | NA |
| OTU5313 | NA | NA | NA | NA | NA | + |
| OTU2769 | + | NA | NA | NA | NA | NA |
| OTU2767 | NA | NA | NA | + | NA | NA |
| OTU2020 | NA | NA | NA | + | NA | NA |
| OTU6070 | NA | NA | NA | NA | NA | + |
| OTU5769 | NA | NA | + | NA | NA | NA |
| OTU2755 | NA | NA | NA | NA | + | NA |
| OTU4751 | NA | NA | NA | NA | + | NA |
| OTU7886 | NA | NA | NA | NA | NA | + |
| OTU8040 | + | NA | NA | NA | NA | NA |
| OTU8068 | NA | NA | NA | NA | NA | + |
| OTU1514 | NA | + | NA | NA | NA | NA |
| OTU5012 | + | NA | NA | NA | NA | NA |
| OTU2 | NA | NA | NA | NA | NA | NA |
| OTU5270 | NA | NA | NA | NA | + | NA |
| OTU937 | NA | NA | + | NA | NA | NA |
| OTU2505 | NA | + | NA | NA | NA | NA |
| OTU5475 | NA | NA | NA | NA | NA | NA |
| OTU4306 | NA | NA | NA | + | NA | NA |
| OTU4603 | NA | NA | NA | NA | NA | NA |
| OTU4600 | NA | + | NA | NA | NA | NA |
| OTU2538 | + | NA | NA | NA | NA | NA |
| OTU3411 | NA | NA | NA | NA | NA | NA |
| OTU5448 | + | NA | NA | NA | NA | NA |
| OTU6217 | NA | NA | NA | NA | NA | NA |
| OTU7344 | + | NA | NA | NA | NA | + |
| OTU64 | NA | NA | NA | NA | NA | + |
| OTU7069 | NA | NA | + | NA | NA | NA |
| OTU7068 | NA | NA | + | NA | NA | NA |
| OTU4613 | NA | + | NA | NA | NA | NA |
| OTU7062 | NA | NA | + | NA | NA | NA |
| OTU7061 | NA | NA | + | NA | NA | NA |
| OTU7060 | NA | NA | + | NA | NA | NA |
| OTU7067 | NA | NA | + | NA | NA | NA |
| OTU7064 | NA | NA | + | NA | NA | NA |
| OTU1920 | NA | NA | + | NA | NA | NA |
| OTU7143 | NA | + | NA | NA | NA | NA |
| OTU5683 | NA | NA | NA | NA | + | NA |
| OTU4757 | NA | NA | NA | NA | + | NA |
| OTU7007 | NA | NA | NA | NA | + | NA |
| OTU396 | NA | NA | + | NA | NA | NA |
| OTU5351 | + | NA | NA | NA | NA | NA |
| OTU4094 | NA | NA | NA | NA | NA | NA |
| OTU453 | NA | NA | NA | NA | NA | + |
| OTU1525 | NA | NA | + | NA | NA | NA |
| OTU1035 | NA | NA | + | NA | NA | NA |
| OTU6700 | NA | NA | + | NA | NA | NA |
| OTU7714 | NA | + | NA | + | NA | NA |
| OTU6 | NA | NA | + | NA | NA | NA |
| OTU1044 | NA | + | NA | NA | NA | NA |
| OTU7096 | NA | NA | + | NA | NA | NA |
| OTU1040 | NA | + | NA | NA | NA | NA |
| OTU2335 | NA | NA | + | NA | NA | NA |
| OTU3073 | NA | NA | NA | + | NA | NA |
| OTU6731 | + | NA | NA | NA | NA | NA |
| OTU3489 | NA | NA | NA | + | NA | NA |
| OTU335 | NA | NA | + | NA | NA | NA |
| OTU4775 | NA | NA | NA | NA | + | NA |
| OTU542 | NA | NA | NA | NA | NA | NA |
| OTU4480 | + | NA | NA | NA | NA | NA |
| OTU2175 | NA | NA | + | NA | NA | NA |
| OTU5479 | + | NA | NA | NA | NA | NA |
| OTU678 | NA | NA | + | NA | NA | NA |
| OTU5457 | + | NA | NA | NA | NA | NA |
| OTU2423 | NA | NA | + | NA | NA | NA |
| OTU5663 | + | NA | NA | NA | NA | NA |
| OTU930 | NA | + | NA | NA | + | NA |
| OTU7277 | NA | NA | NA | + | NA | NA |
| OTU5489 | + | NA | NA | NA | NA | NA |
| OTU1183 | NA | NA | + | NA | NA | NA |
| OTU1772 | NA | NA | + | NA | NA | NA |
| OTU3233 | + | NA | NA | NA | NA | NA |
| OTU632 | NA | + | NA | NA | NA | NA |
| OTU2955 | NA | NA | + | NA | NA | NA |
| OTU2950 | NA | NA | + | NA | NA | NA |
| OTU2953 | NA | NA | + | NA | NA | NA |
| OTU4695 | NA | NA | NA | + | NA | NA |
| OTU5600 | NA | NA | NA | NA | + | NA |
| OTU4876 | NA | NA | NA | NA | NA | NA |
| OTU5602 | NA | NA | NA | NA | + | NA |
| OTU1986 | + | NA | NA | NA | NA | NA |
| OTU1985 | NA | NA | NA | + | NA | NA |
| OTU2306 | NA | NA | NA | NA | NA | + |
| OTU2931 | NA | NA | NA | NA | NA | NA |
| OTU3948 | NA | NA | NA | NA | NA | NA |
| OTU3013 | + | NA | NA | NA | NA | NA |
| OTU1723 | NA | NA | NA | + | NA | NA |
| OTU1720 | NA | NA | NA | NA | + | NA |
| OTU2983 | NA | NA | + | NA | NA | NA |
| OTU4269 | NA | NA | NA | + | NA | NA |
| OTU4262 | NA | NA | NA | + | NA | NA |
| OTU1162 | + | NA | NA | NA | NA | NA |
| OTU4265 | NA | NA | NA | + | NA | NA |
| OTU4714 | NA | NA | NA | NA | NA | NA |
| OTU4023 | NA | NA | NA | NA | NA | NA |
| OTU4908 | + | NA | NA | NA | NA | NA |
| OTU4298 | NA | NA | NA | NA | NA | NA |
| OTU7307 | NA | + | NA | NA | NA | NA |
| OTU4006 | NA | NA | NA | + | NA | NA |
| OTU7449 | NA | NA | NA | + | NA | NA |
| OTU4003 | NA | NA | + | NA | NA | NA |
| OTU4899 | NA | NA | NA | NA | NA | NA |
| OTU5759 | NA | NA | NA | NA | NA | + |
| OTU5751 | + | NA | NA | NA | NA | NA |
| OTU2668 | NA | NA | NA | NA | + | NA |
| OTU6949 | NA | NA | NA | NA | + | NA |
| OTU3037 | NA | NA | + | NA | NA | NA |
| OTU2212 | NA | NA | + | NA | NA | NA |
| OTU4197 | NA | + | NA | NA | NA | NA |
| OTU7586 | NA | NA | NA | NA | NA | NA |
| OTU1476 | NA | NA | NA | NA | + | NA |
| OTU4013 | NA | NA | NA | NA | NA | NA |
| OTU7472 | NA | NA | NA | NA | NA | + |
| OTU7473 | NA | NA | NA | NA | NA | NA |
| OTU6935 | NA | NA | NA | NA | NA | + |
| OTU7487 | NA | NA | NA | NA | + | NA |
| OTU7003 | NA | NA | NA | NA | NA | + |
| OTU2780 | + | NA | NA | NA | NA | NA |
| OTU7839 | + | NA | NA | NA | NA | NA |
| OTU1512 | NA | NA | NA | NA | NA | NA |
| OTU4828 | + | NA | NA | NA | NA | NA |
| OTU1209 | NA | NA | + | NA | NA | NA |
| OTU6638 | NA | NA | NA | NA | NA | + |
| OTU852 | NA | NA | NA | + | NA | NA |
| OTU4580 | NA | NA | NA | NA | + | NA |
| OTU1071 | NA | NA | NA | NA | NA | + |
| OTU8120 | NA | NA | NA | NA | NA | NA |
| OTU4928 | NA | NA | NA | NA | NA | NA |
| OTU1698 | NA | NA | NA | + | NA | NA |
| OTU2379 | NA | NA | NA | NA | + | NA |
| OTU595 | NA | NA | + | NA | NA | NA |
| OTU5850 | NA | NA | NA | NA | NA | + |
| OTU7033 | NA | NA | + | NA | NA | NA |
| OTU1431 | NA | NA | NA | NA | NA | NA |
| OTU3362 | NA | NA | + | + | NA | NA |
| OTU4453 | NA | NA | NA | NA | NA | NA |
| OTU5081 | NA | NA | NA | NA | NA | + |
| OTU6508 | NA | NA | NA | + | NA | NA |
| OTU4274 | NA | NA | NA | + | NA | NA |
| OTU287 | + | NA | NA | + | NA | NA |
| OTU7042 | NA | NA | + | NA | NA | NA |
| OTU6957 | NA | NA | NA | NA | NA | + |
| OTU1062 | NA | NA | + | NA | NA | NA |
| OTU4201 | NA | + | NA | NA | NA | NA |
| OTU2903 | NA | + | NA | NA | NA | NA |
| OTU4085 | + | NA | NA | NA | NA | NA |
| OTU6711 | NA | NA | NA | NA | NA | + |
| OTU6077 | NA | NA | + | NA | NA | NA |
| OTU7289 | + | NA | NA | NA | NA | NA |
| OTU7107 | NA | NA | + | NA | NA | NA |
| OTU4401 | + | NA | NA | NA | NA | NA |
| OTU5653 | NA | NA | + | NA | NA | NA |
| OTU2833 | NA | + | NA | NA | NA | NA |
| OTU5039 | NA | NA | NA | NA | + | NA |
| OTU6840 | NA | NA | + | NA | NA | NA |
| OTU5682 | NA | NA | NA | + | NA | NA |
| OTU5210 | + | NA | NA | NA | NA | NA |
| OTU4371 | NA | + | NA | NA | NA | NA |
| OTU7099 | NA | NA | + | NA | NA | NA |
| OTU3317 | NA | NA | NA | NA | + | NA |
| OTU4920 | NA | NA | NA | NA | + | NA |
| OTU4925 | + | NA | NA | NA | NA | NA |
| OTU4924 | + | NA | NA | NA | NA | NA |
| OTU3842 | NA | NA | + | NA | NA | NA |
| OTU1782 | NA | NA | + | NA | NA | NA |
| OTU8058 | NA | NA | NA | + | NA | NA |
| OTU3861 | NA | NA | + | NA | NA | NA |
| OTU3930 | + | NA | NA | NA | NA | NA |
| OTU7294 | NA | NA | NA | + | NA | NA |
| OTU4184 | NA | + | NA | NA | NA | NA |
| OTU2743 | NA | NA | NA | NA | NA | + |
| OTU2746 | NA | NA | NA | + | NA | NA |
| OTU4189 | NA | + | NA | NA | NA | NA |
| OTU4839 | + | NA | NA | NA | NA | NA |
| OTU5586 | + | NA | NA | NA | NA | NA |
| OTU8213 | NA | NA | NA | + | NA | NA |
| OTU4988 | NA | NA | NA | NA | + | NA |
| OTU6719 | NA | NA | NA | + | NA | NA |
| OTU1820 | + | NA | NA | NA | NA | NA |
| OTU1812 | + | NA | NA | NA | NA | NA |
| OTU2256 | + | NA | NA | NA | NA | NA |
| OTU28 | NA | + | NA | NA | NA | NA |
| OTU2777 | NA | NA | NA | NA | + | NA |
| OTU790 | + | NA | + | NA | NA | NA |
| OTU4496 | NA | NA | NA | NA | + | NA |
| OTU5998 | NA | NA | NA | NA | NA | + |
| OTU1541 | NA | NA | NA | + | NA | NA |
| OTU5059 | + | NA | NA | NA | NA | NA |
| OTU5127 | NA | NA | NA | NA | NA | NA |
| OTU4747 | NA | NA | NA | NA | + | NA |
| OTU4746 | NA | NA | NA | NA | + | NA |
| OTU4740 | NA | + | NA | NA | NA | NA |
| OTU2569 | + | NA | NA | NA | NA | NA |
| OTU2568 | + | NA | NA | NA | NA | NA |
| OTU7093 | NA | NA | + | NA | NA | NA |
| OTU2229 | NA | NA | + | NA | NA | NA |
| OTU4918 | NA | NA | NA | NA | NA | NA |
| OTU4737 | + | NA | NA | NA | NA | NA |
| OTU5841 | NA | NA | NA | NA | NA | + |
| OTU6051 | NA | NA | NA | + | NA | NA |
| OTU4827 | NA | NA | NA | + | NA | NA |
| OTU5983 | NA | NA | NA | NA | NA | + |
| OTU7957 | NA | NA | NA | NA | NA | + |
| OTU2818 | + | NA | NA | NA | NA | NA |
| OTU2234 | + | NA | NA | NA | NA | NA |
| OTU5442 | NA | NA | NA | + | NA | NA |
| OTU7624 | NA | NA | NA | NA | NA | + |
| OTU5706 | + | NA | NA | NA | NA | NA |
| OTU327 | NA | NA | NA | + | NA | NA |
| OTU1711 | NA | NA | NA | NA | NA | + |
| OTU4242 | NA | + | NA | NA | NA | NA |
| OTU4248 | NA | NA | NA | + | NA | NA |
| OTU8178 | NA | NA | NA | NA | NA | NA |
| OTU4052 | NA | NA | NA | + | NA | NA |
| OTU5999 | NA | NA | + | NA | NA | NA |
| OTU5993 | NA | NA | NA | NA | NA | + |
| OTU6756 | NA | NA | NA | NA | NA | NA |
| OTU655 | NA | NA | NA | NA | NA | NA |
| OTU4701 | + | NA | NA | NA | NA | NA |
| OTU39 | NA | NA | NA | NA | NA | + |
| OTU4803 | NA | + | NA | NA | + | NA |
| OTU4307 | NA | NA | NA | + | NA | NA |
| OTU4009 | NA | NA | NA | NA | NA | + |
| OTU2215 | NA | NA | NA | + | NA | NA |
| OTU3369 | NA | NA | NA | + | NA | NA |
| OTU3661 | + | NA | NA | NA | NA | NA |
| OTU2266 | NA | NA | NA | + | NA | NA |
| OTU3039 | NA | NA | + | NA | NA | + |
| OTU7049 | NA | NA | + | NA | NA | NA |
| OTU3032 | NA | NA | + | NA | NA | NA |
| OTU6064 | NA | NA | + | NA | NA | NA |
| OTU5244 | NA | NA | NA | NA | + | NA |
| OTU6965 | + | NA | NA | NA | NA | NA |
| OTU4807 | NA | NA | NA | NA | + | NA |
| OTU3390 | NA | NA | NA | + | NA | NA |
| OTU172 | NA | + | NA | NA | NA | NA |
| OTU7952 | NA | NA | NA | NA | NA | + |
| OTU5204 | NA | NA | NA | NA | + | NA |
| OTU2912 | NA | NA | NA | NA | NA | NA |
| OTU4545 | NA | NA | NA | NA | + | NA |
| OTU2279 | NA | NA | NA | NA | + | NA |
| OTU2273 | NA | NA | NA | NA | + | NA |
| OTU5913 | NA | NA | NA | NA | NA | + |
| OTU5915 | NA | NA | NA | NA | NA | + |
| OTU2222 | + | NA | NA | NA | NA | NA |
| OTU5919 | NA | NA | NA | NA | NA | + |
| OTU6567 | + | NA | NA | NA | NA | NA |
| OTU6812 | NA | NA | NA | NA | NA | NA |
| OTU2881 | NA | NA | NA | + | NA | NA |
| OTU3465 | NA | NA | NA | + | NA | NA |
| OTU4022 | NA | NA | NA | + | NA | NA |
| OTU5337 | NA | NA | + | NA | NA | NA |
| OTU7877 | NA | NA | + | NA | NA | NA |
| OTU5827 | NA | NA | NA | NA | NA | NA |
| OTU5231 | NA | NA | NA | + | NA | NA |
| OTU5195 | + | NA | NA | NA | NA | NA |
| OTU5199 | NA | NA | NA | NA | NA | NA |
| OTU7044 | NA | NA | + | NA | NA | NA |
| OTU7515 | + | NA | NA | NA | NA | NA |
| OTU7882 | NA | NA | NA | NA | NA | NA |
| OTU1124 | NA | NA | + | NA | NA | NA |
| OTU3768 | + | NA | NA | NA | NA | NA |
| OTU1303 | NA | + | NA | NA | NA | NA |
| OTU5371 | + | NA | NA | + | NA | NA |
| OTU5899 | NA | NA | NA | + | NA | NA |
| OTU133 | NA | NA | NA | + | NA | NA |
| OTU6504 | NA | NA | NA | + | NA | NA |
| OTU4424 | + | NA | NA | NA | NA | NA |
| OTU1368 | NA | NA | NA | NA | + | NA |
| OTU3483 | NA | NA | NA | + | NA | NA |
| OTU2959 | NA | NA | + | NA | NA | NA |
| OTU3488 | NA | NA | NA | + | NA | NA |
| OTU4260 | NA | NA | NA | + | NA | NA |
| OTU4799 | NA | NA | NA | NA | + | NA |
| OTU4461 | + | NA | NA | NA | NA | NA |
| OTU5525 | NA | NA | NA | NA | + | NA |
| OTU4903 | NA | NA | NA | + | NA | NA |
| OTU4902 | + | NA | NA | NA | NA | NA |
| OTU5757 | NA | NA | NA | NA | + | NA |
| OTU3261 | NA | NA | + | NA | NA | NA |
| OTU3260 | NA | NA | NA | NA | NA | NA |
| OTU5777 | NA | NA | + | NA | NA | NA |
| OTU2748 | NA | NA | NA | + | NA | NA |
| OTU4642 | NA | NA | NA | + | NA | NA |
| OTU3307 | NA | NA | NA | + | NA | NA |
| OTU8113 | NA | + | NA | NA | NA | NA |
| OTU2186 | NA | NA | NA | NA | + | NA |
| OTU8100 | + | NA | NA | NA | NA | NA |
| OTU2054 | NA | NA | NA | + | NA | NA |
| OTU3634 | NA | NA | NA | NA | + | NA |
| OTU5909 | NA | NA | NA | NA | NA | + |
| OTU1839 | NA | NA | NA | + | NA | NA |
| OTU1432 | NA | NA | NA | NA | NA | NA |
| OTU4761 | NA | NA | NA | NA | + | NA |
| OTU6478 | NA | + | NA | NA | NA | NA |
| OTU5193 | NA | NA | NA | NA | + | NA |
| OTU5419 | NA | NA | + | NA | NA | NA |
| OTU7053 | NA | NA | + | NA | NA | NA |
| OTU3441 | NA | NA | NA | + | NA | NA |
| OTU1257 | NA | NA | + | NA | NA | NA |
| OTU1389 | NA | NA | NA | + | NA | NA |
| OTU3308 | NA | NA | NA | + | NA | NA |
| OTU4844 | + | NA | NA | NA | NA | NA |
| OTU4770 | NA | NA | NA | NA | + | NA |
| OTU4754 | NA | NA | NA | NA | NA | NA |
| OTU2301 | NA | NA | NA | NA | NA | NA |
| OTU460 | NA | NA | + | NA | NA | NA |
| OTU430 | NA | NA | + | NA | NA | NA |
| OTU467 | NA | + | NA | NA | NA | NA |
| OTU468 | NA | NA | + | NA | NA | NA |
| OTU6610 | NA | NA | NA | + | NA | NA |
| OTU4763 | NA | NA | NA | NA | + | NA |
| OTU3059 | NA | NA | NA | NA | NA | NA |
| OTU4765 | NA | NA | NA | NA | + | NA |
| OTU3065 | NA | NA | NA | + | NA | NA |
| OTU4769 | NA | NA | NA | NA | NA | + |
| OTU1241 | NA | NA | NA | NA | NA | + |
| OTU629 | NA | NA | + | NA | NA | NA |
| OTU7057 | NA | NA | + | NA | NA | NA |
| OTU5516 | NA | NA | NA | + | NA | NA |
| OTU2942 | NA | NA | NA | NA | NA | NA |
| OTU673 | NA | NA | + | NA | NA | NA |
| OTU662 | NA | NA | + | NA | NA | NA |
| OTU197 | NA | NA | NA | NA | NA | + |
| OTU7174 | NA | NA | + | NA | NA | NA |
| OTU1408 | NA | NA | + | NA | NA | NA |
| OTU3320 | NA | NA | NA | + | NA | NA |
| OTU1878 | NA | + | NA | NA | NA | NA |
| OTU5312 | NA | NA | NA | NA | NA | NA |
| OTU1962 | NA | NA | + | NA | NA | NA |
| OTU1348 | NA | NA | NA | NA | NA | NA |
| OTU6686 | NA | NA | NA | NA | NA | NA |
| OTU1827 | NA | NA | NA | NA | NA | NA |
| OTU4781 | NA | NA | NA | NA | + | NA |
| OTU2908 | NA | NA | NA | + | NA | NA |
| OTU1268 | NA | NA | NA | NA | NA | NA |
| OTU6348 | + | NA | NA | NA | NA | NA |
| OTU8181 | NA | NA | + | NA | NA | NA |
| OTU1337 | NA | + | NA | + | NA | NA |
| OTU3601 | NA | NA | NA | NA | NA | + |
| OTU6194 | NA | NA | NA | NA | NA | + |
| OTU6197 | NA | NA | NA | NA | NA | + |
| OTU6190 | NA | NA | NA | NA | NA | + |
| OTU574 | NA | NA | + | NA | NA | NA |
| OTU8169 | + | NA | NA | NA | NA | NA |
| OTU5738 | NA | + | NA | NA | NA | NA |
| OTU7802 | NA | NA | + | NA | NA | NA |
| OTU7027 | NA | NA | + | NA | NA | NA |
| OTU429 | NA | NA | + | NA | NA | NA |
| OTU3010 | NA | NA | + | NA | NA | NA |
| OTU7024 | NA | NA | + | NA | NA | NA |
| OTU3016 | NA | NA | + | NA | NA | NA |
| OTU537 | NA | NA | + | NA | NA | NA |
| OTU4005 | NA | NA | NA | NA | NA | NA |
| OTU4526 | + | NA | NA | NA | NA | NA |
| OTU7960 | NA | NA | NA | NA | + | NA |
| OTU5103 | NA | NA | NA | NA | NA | + |
| OTU7021 | NA | NA | + | NA | NA | NA |
| OTU5412 | + | NA | NA | NA | NA | NA |
| OTU5256 | NA | + | NA | NA | + | NA |
| OTU7950 | + | NA | NA | NA | NA | NA |
| OTU5832 | NA | NA | NA | + | NA | NA |
| OTU1272 | NA | + | NA | NA | NA | NA |
| OTU4325 | NA | NA | NA | + | NA | NA |
| OTU4947 | NA | NA | NA | + | NA | NA |
| OTU4753 | NA | NA | NA | NA | + | NA |
| OTU5176 | NA | NA | NA | NA | + | NA |
| OTU3047 | NA | NA | NA | + | NA | NA |
| OTU2635 | NA | NA | NA | NA | NA | NA |
| OTU2876 | + | NA | NA | NA | NA | NA |
| OTU6262 | NA | NA | + | NA | NA | NA |
| OTU5095 | NA | + | NA | NA | NA | NA |
| OTU3703 | NA | NA | NA | NA | NA | NA |
| OTU4961 | NA | NA | + | NA | NA | NA |
| OTU2627 | NA | NA | NA | NA | + | NA |
| OTU5002 | NA | NA | + | NA | NA | NA |
| OTU4631 | NA | + | NA | NA | NA | NA |
| OTU6886 | NA | NA | NA | NA | NA | + |
| OTU4604 | NA | NA | + | NA | NA | NA |
| OTU4109 | NA | NA | + | NA | NA | NA |
| OTU6592 | NA | NA | + | NA | NA | NA |
| OTU8174 | NA | NA | + | NA | NA | NA |
| OTU1610 | NA | NA | NA | NA | NA | NA |
| OTU7022 | NA | NA | + | NA | NA | NA |
| OTU7505 | NA | NA | NA | NA | NA | NA |
| OTU688 | NA | NA | + | NA | NA | NA |
| OTU612 | NA | NA | NA | NA | NA | NA |
| OTU2077 | NA | NA | NA | + | NA | NA |
| OTU3005 | NA | NA | + | NA | NA | NA |
| OTU1411 | NA | NA | NA | + | NA | NA |
| OTU880 | NA | NA | + | NA | NA | NA |
| OTU1321 | + | NA | NA | NA | NA | NA |
| OTU4019 | NA | NA | NA | NA | NA | NA |
| OTU1987 | NA | NA | + | NA | NA | NA |
| OTU2807 | NA | NA | + | NA | NA | NA |
| OTU8254 | NA | NA | NA | NA | NA | + |
| OTU2831 | NA | NA | NA | NA | + | NA |
| OTU4564 | NA | NA | NA | NA | + | NA |
| OTU7031 | NA | NA | + | NA | NA | NA |
| OTU4220 | NA | + | NA | NA | NA | NA |
| OTU4221 | NA | + | NA | NA | NA | NA |
| OTU4222 | NA | + | NA | NA | NA | NA |
| OTU3656 | + | NA | NA | NA | NA | NA |
| OTU4228 | NA | + | NA | NA | NA | NA |
| OTU417 | NA | + | + | NA | NA | NA |
| OTU675 | NA | NA | + | NA | NA | NA |
| OTU1509 | + | NA | NA | NA | NA | NA |
| OTU60 | NA | NA | + | NA | NA | NA |
| OTU5797 | NA | NA | NA | NA | NA | + |
| OTU5214 | NA | NA | NA | NA | + | NA |
| OTU5159 | NA | NA | NA | NA | + | NA |
| OTU123 | NA | NA | NA | NA | NA | NA |
| OTU5236 | NA | NA | NA | NA | + | NA |
| OTU910 | NA | NA | + | NA | NA | NA |
| OTU2319 | + | NA | NA | NA | NA | NA |
| OTU2030 | NA | NA | + | NA | NA | NA |
| OTU797 | NA | NA | NA | NA | + | NA |
| OTU4036 | NA | NA | NA | NA | + | NA |
| OTU8270 | NA | NA | NA | + | NA | NA |
| OTU2582 | NA | NA | NA | NA | + | NA |
| OTU6779 | NA | NA | NA | NA | NA | NA |
| OTU2972 | NA | + | NA | NA | NA | NA |
| OTU4181 | NA | NA | NA | NA | NA | NA |
| OTU6394 | NA | NA | NA | + | NA | NA |
| OTU3878 | + | NA | NA | NA | NA | NA |
| OTU890 | NA | + | NA | NA | NA | NA |
| OTU7815 | NA | NA | NA | NA | NA | + |
| OTU2332 | NA | NA | + | NA | NA | NA |
| OTU7923 | NA | + | NA | NA | NA | NA |
| OTU5897 | NA | NA | NA | NA | NA | + |
| OTU5335 | NA | NA | NA | NA | NA | + |
| OTU7045 | NA | + | NA | NA | NA | NA |
| OTU3619 | NA | NA | NA | NA | NA | NA |
| OTU7163 | NA | NA | NA | NA | NA | NA |
| OTU4987 | NA | + | NA | NA | NA | NA |
| OTU1258 | NA | NA | NA | NA | NA | NA |
| OTU7181 | NA | NA | + | NA | NA | NA |
| OTU5608 | NA | NA | NA | NA | + | NA |
| OTU1511 | NA | NA | NA | NA | NA | NA |
| OTU4875 | NA | NA | NA | NA | NA | NA |
| OTU3323 | NA | + | NA | NA | NA | NA |
| OTU271 | NA | NA | NA | + | NA | NA |
| OTU4745 | NA | NA | NA | NA | + | NA |
| OTU5197 | + | NA | NA | NA | NA | NA |
| OTU879 | NA | NA | NA | NA | NA | + |
| OTU4493 | + | NA | NA | NA | NA | NA |
| OTU3002 | NA | NA | + | NA | NA | NA |
| OTU6628 | NA | NA | NA | NA | NA | NA |
| OTU6525 | NA | NA | + | NA | NA | NA |
| OTU5406 | NA | + | NA | NA | NA | NA |
| OTU5073 | + | + | NA | + | NA | NA |
| OTU4449 | NA | NA | NA | NA | NA | NA |
| OTU4448 | + | NA | NA | NA | NA | NA |
| OTU4442 | + | NA | NA | NA | NA | NA |
| OTU3792 | NA | NA | NA | NA | NA | + |
| OTU5456 | + | NA | NA | NA | NA | NA |
| OTU3159 | NA | NA | NA | NA | NA | + |
| OTU3720 | NA | NA | NA | NA | + | NA |
| OTU268 | NA | + | NA | NA | NA | NA |
| OTU3023 | NA | NA | + | NA | NA | NA |
| OTU4565 | NA | NA | NA | NA | + | NA |
| OTU4111 | + | NA | NA | NA | NA | NA |
| OTU4529 | + | NA | NA | NA | NA | NA |
| OTU7853 | NA | NA | NA | + | NA | NA |
| OTU4647 | NA | NA | + | NA | NA | NA |
| OTU4960 | NA | NA | NA | NA | NA | NA |
| OTU709 | NA | NA | NA | + | NA | NA |
| OTU5538 | NA | + | NA | NA | NA | NA |
| OTU1787 | NA | NA | + | NA | NA | NA |
| OTU3200 | NA | NA | + | NA | NA | NA |
| OTU5512 | NA | NA | NA | NA | NA | + |
| OTU3105 | NA | NA | + | + | NA | NA |
| OTU5097 | NA | + | NA | NA | NA | NA |
| OTU4134 | + | NA | NA | NA | NA | NA |
| OTU3293 | + | NA | NA | NA | NA | NA |
| OTU5156 | NA | NA | NA | NA | + | NA |
| OTU1699 | NA | NA | NA | NA | NA | NA |
| OTU5234 | NA | NA | NA | NA | + | NA |
| OTU2371 | NA | NA | + | NA | NA | NA |
| OTU6201 | NA | NA | NA | NA | NA | + |
| OTU3384 | NA | NA | NA | + | NA | NA |
| OTU3153 | NA | NA | NA | NA | + | NA |
| OTU1240 | NA | NA | NA | + | NA | NA |
| OTU5922 | + | NA | NA | NA | NA | NA |
| OTU5468 | + | NA | NA | NA | NA | NA |
| OTU7043 | NA | NA | NA | NA | NA | NA |
| OTU666 | NA | NA | NA | NA | NA | + |
| OTU5979 | NA | NA | NA | + | NA | NA |
| OTU7561 | NA | NA | NA | NA | NA | + |
| OTU1243 | + | NA | NA | NA | NA | NA |
| OTU4849 | + | NA | NA | NA | NA | NA |
| OTU6511 | NA | NA | NA | NA | NA | NA |
| OTU3576 | NA | NA | NA | NA | NA | NA |
| OTU1199 | + | NA | NA | NA | NA | NA |
| OTU4784 | NA | NA | NA | NA | + | NA |
| OTU2529 | NA | NA | + | NA | NA | NA |
| OTU5668 | + | NA | NA | NA | NA | NA |
| OTU3142 | + | NA | NA | NA | NA | NA |
| OTU5009 | NA | NA | NA | NA | NA | NA |
| OTU5881 | NA | NA | NA | NA | NA | + |
| OTU8263 | NA | + | NA | NA | NA | NA |
| OTU8138 | NA | NA | + | NA | NA | NA |
| OTU4219 | NA | + | NA | NA | NA | NA |
| OTU5029 | NA | NA | NA | NA | NA | NA |
| OTU657 | NA | NA | + | NA | NA | NA |
| OTU6390 | NA | NA | NA | NA | + | NA |
| OTU25 | NA | + | NA | NA | NA | NA |
| OTU117 | NA | NA | + | NA | NA | NA |
| OTU4205 | NA | + | NA | NA | NA | NA |
| OTU4202 | NA | + | NA | NA | NA | NA |
| OTU4200 | NA | + | NA | NA | NA | NA |
| OTU2370 | NA | NA | NA | NA | NA | NA |
| OTU1615 | NA | NA | + | NA | NA | NA |
| OTU7674 | NA | NA | NA | NA | NA | NA |
| OTU4776 | NA | NA | NA | NA | + | NA |
| OTU4270 | NA | NA | NA | NA | NA | NA |
| OTU617 | NA | NA | + | NA | NA | NA |
| OTU614 | NA | NA | + | NA | NA | NA |
| OTU1555 | NA | NA | NA | NA | + | NA |
| OTU613 | NA | NA | + | NA | NA | NA |
| OTU2725 | NA | NA | NA | NA | + | NA |
| OTU5237 | NA | NA | NA | NA | + | NA |
| OTU619 | NA | NA | + | NA | NA | NA |
| OTU3493 | NA | NA | NA | + | NA | NA |
| OTU519 | NA | NA | + | NA | NA | NA |
| OTU5392 | NA | + | NA | NA | NA | NA |
| OTU5633 | NA | NA | NA | NA | NA | NA |
| OTU4412 | + | NA | NA | NA | NA | NA |
| OTU3427 | NA | NA | NA | NA | NA | NA |
| OTU618 | NA | NA | + | NA | NA | NA |
| OTU4209 | NA | + | NA | NA | NA | NA |
| OTU3215 | NA | NA | + | NA | NA | NA |
| OTU4856 | + | NA | NA | NA | NA | NA |
| OTU1982 | NA | NA | NA | NA | NA | NA |
| OTU4859 | + | NA | NA | NA | NA | NA |
| OTU7527 | + | NA | NA | NA | + | NA |
| OTU2712 | + | NA | NA | NA | NA | NA |
| OTU6902 | + | NA | NA | NA | NA | NA |
| OTU3074 | NA | NA | NA | + | NA | NA |
| OTU7559 | NA | NA | NA | NA | NA | + |
| OTU7558 | NA | NA | + | NA | NA | NA |
| OTU7005 | NA | NA | NA | NA | NA | + |
| OTU436 | NA | NA | + | NA | NA | NA |
| OTU4103 | + | NA | NA | NA | NA | NA |
| OTU4368 | + | NA | NA | NA | NA | NA |
| OTU5332 | NA | NA | NA | NA | NA | NA |
| OTU6255 | NA | NA | + | NA | NA | NA |
| OTU5846 | + | NA | NA | NA | NA | NA |
| OTU5845 | NA | NA | NA | NA | NA | + |
| OTU4972 | NA | NA | NA | NA | + | NA |
| OTU4772 | + | NA | NA | NA | + | NA |
| OTU7530 | NA | NA | NA | NA | NA | + |
| OTU2951 | NA | NA | + | NA | NA | NA |
| OTU4133 | NA | NA | + | NA | NA | + |
| OTU5840 | NA | NA | NA | NA | NA | NA |
| OTU2235 | + | NA | NA | NA | NA | NA |
| OTU6753 | + | NA | NA | NA | NA | NA |
| OTU2981 | NA | NA | + | NA | NA | NA |
| OTU3351 | NA | NA | NA | NA | + | NA |
| OTU6873 | NA | NA | NA | NA | NA | NA |
| OTU6364 | NA | NA | NA | NA | NA | NA |
| OTU2317 | + | NA | NA | + | NA | NA |
| OTU2457 | NA | + | NA | NA | NA | NA |
| OTU2994 | NA | NA | NA | + | NA | NA |
| OTU5859 | NA | NA | NA | NA | NA | + |
| OTU2182 | NA | NA | NA | NA | + | NA |
| OTU1696 | NA | NA | + | NA | NA | NA |
| OTU7009 | NA | NA | NA | + | NA | NA |
| OTU5158 | + | NA | NA | NA | NA | NA |
| OTU5155 | NA | NA | NA | NA | NA | NA |
| OTU7156 | NA | NA | + | NA | NA | NA |
| OTU769 | NA | NA | NA | + | NA | NA |
| OTU4277 | NA | NA | NA | + | NA | NA |
| OTU4215 | NA | + | NA | NA | NA | NA |
| OTU4253 | NA | NA | NA | + | NA | NA |
| OTU3428 | NA | NA | NA | + | NA | NA |
| OTU389 | NA | NA | + | NA | NA | NA |
| OTU1349 | NA | NA | NA | + | NA | NA |
| OTU1344 | + | NA | + | NA | NA | NA |
| OTU1347 | NA | NA | NA | NA | NA | + |
| OTU4467 | + | NA | NA | NA | NA | NA |
| OTU4469 | + | NA | NA | NA | NA | NA |
| OTU2385 | NA | NA | + | NA | NA | NA |
| OTU6015 | NA | NA | + | NA | NA | NA |
| OTU6542 | NA | NA | NA | NA | NA | + |
| OTU2390 | NA | NA | NA | NA | NA | NA |
| OTU7351 | NA | + | NA | NA | NA | NA |
| OTU3203 | NA | NA | + | NA | NA | NA |
| OTU3444 | NA | NA | NA | + | NA | NA |
| OTU3446 | NA | NA | NA | + | NA | NA |
| OTU3443 | NA | NA | NA | + | NA | NA |
| OTU2586 | NA | NA | NA | + | NA | NA |
| OTU6763 | NA | NA | NA | NA | + | NA |
| OTU6872 | NA | NA | + | NA | NA | NA |
| OTU5789 | NA | NA | + | NA | NA | NA |
| OTU6384 | NA | + | NA | NA | NA | NA |
| OTU3828 | NA | NA | NA | + | NA | NA |
| OTU611 | NA | NA | + | NA | NA | NA |
| OTU5259 | NA | NA | NA | NA | + | NA |
| OTU1224 | NA | NA | NA | NA | NA | NA |
| OTU3148 | + | NA | NA | NA | NA | NA |
| OTU4161 | NA | + | NA | NA | NA | NA |
| OTU4404 | + | NA | NA | NA | NA | NA |
| OTU3892 | NA | NA | + | NA | NA | NA |
| OTU3795 | + | NA | NA | NA | NA | NA |
| OTU4584 | NA | NA | NA | NA | + | NA |
| OTU5949 | NA | NA | NA | + | NA | NA |
| OTU4886 | + | NA | NA | NA | NA | NA |
| OTU4986 | NA | NA | NA | NA | NA | NA |
| OTU4906 | + | NA | NA | NA | NA | NA |
| OTU2268 | NA | NA | NA | NA | NA | + |
| OTU2819 | NA | + | NA | NA | NA | NA |
| OTU7860 | NA | NA | NA | + | NA | NA |
| OTU5008 | + | NA | NA | NA | NA | NA |
| OTU7012 | + | NA | NA | NA | NA | NA |
| OTU745 | NA | NA | NA | NA | NA | + |
| OTU2979 | NA | NA | + | NA | NA | NA |
| OTU7100 | NA | NA | + | NA | NA | NA |
| OTU1930 | NA | NA | NA | NA | NA | NA |
| OTU1796 | NA | NA | NA | NA | NA | NA |
| OTU2417 | NA | NA | NA | NA | NA | NA |
| OTU7150 | NA | NA | + | NA | NA | NA |
| OTU3994 | NA | NA | NA | + | NA | NA |
| OTU3226 | NA | NA | NA | NA | NA | NA |
| OTU3229 | NA | + | NA | NA | NA | NA |
| OTU7948 | NA | NA | + | NA | NA | NA |
| OTU780 | NA | NA | + | NA | NA | NA |
| OTU3111 | + | NA | NA | NA | NA | NA |
| OTU5871 | NA | NA | NA | NA | NA | + |
| OTU3419 | NA | NA | NA | + | NA | NA |
| OTU4655 | + | NA | NA | NA | + | NA |
| OTU4046 | NA | NA | NA | NA | NA | NA |
| OTU7074 | NA | NA | + | NA | NA | NA |
| OTU2355 | NA | NA | + | NA | NA | NA |
| OTU1215 | + | NA | NA | NA | NA | NA |
| OTU226 | NA | NA | + | NA | NA | NA |
| OTU4744 | NA | NA | NA | NA | NA | NA |
| OTU812 | NA | NA | + | NA | NA | NA |
| OTU7461 | NA | NA | NA | NA | + | NA |
| OTU2127 | NA | NA | NA | + | NA | NA |
| OTU8274 | NA | NA | + | NA | NA | NA |
| OTU4231 | NA | + | NA | NA | NA | NA |
| OTU4417 | + | NA | NA | NA | NA | NA |
| OTU4415 | + | NA | NA | NA | NA | NA |
| OTU6574 | NA | NA | NA | NA | NA | NA |
| OTU3473 | NA | NA | NA | + | NA | NA |
| OTU5616 | NA | NA | + | NA | NA | NA |
| OTU3824 | NA | NA | + | NA | NA | NA |
| OTU5614 | NA | + | NA | NA | NA | NA |
| OTU566 | NA | NA | + | NA | NA | NA |
| OTU2933 | NA | NA | NA | + | NA | NA |
| OTU3788 | NA | NA | NA | + | NA | NA |
| OTU7805 | NA | NA | NA | NA | + | NA |
| OTU3057 | NA | NA | NA | + | NA | NA |
| OTU3052 | NA | NA | NA | + | NA | NA |
| OTU7140 | NA | NA | + | NA | NA | NA |
| OTU5950 | NA | NA | NA | NA | NA | NA |
| OTU533 | NA | + | NA | NA | NA | NA |
| OTU3977 | NA | NA | + | NA | NA | NA |
| OTU6959 | NA | + | NA | NA | NA | NA |
| OTU6489 | NA | NA | NA | + | NA | NA |
| OTU4267 | NA | NA | NA | + | NA | NA |
| OTU6956 | NA | NA | NA | NA | NA | + |
| OTU4081 | NA | NA | + | NA | NA | NA |
| OTU3900 | NA | NA | NA | NA | NA | NA |
| OTU7065 | NA | NA | NA | NA | NA | NA |
| OTU5453 | + | NA | NA | NA | NA | NA |
| OTU5137 | NA | NA | NA | NA | NA | + |
| OTU8153 | NA | NA | NA | NA | NA | + |
| OTU7116 | NA | NA | + | NA | NA | NA |
| OTU6131 | NA | NA | NA | NA | NA | NA |
| OTU3085 | NA | NA | NA | NA | + | NA |
| OTU7962 | NA | NA | NA | NA | NA | NA |
| OTU6541 | NA | NA | + | NA | NA | NA |
| OTU8285 | + | NA | NA | + | NA | NA |
| OTU2839 | NA | NA | NA | NA | NA | NA |
| OTU6105 | NA | NA | NA | + | NA | NA |
| OTU6229 | NA | NA | NA | + | NA | NA |
| OTU681 | NA | NA | NA | NA | NA | NA |
| OTU4919 | NA | NA | NA | NA | NA | NA |
| OTU7615 | NA | + | NA | NA | NA | NA |
| OTU3201 | NA | + | + | NA | NA | NA |
| OTU5883 | NA | NA | NA | NA | NA | + |
| OTU3283 | NA | + | NA | NA | NA | NA |
| OTU8284 | NA | NA | NA | NA | NA | NA |
| OTU8289 | NA | NA | + | NA | NA | NA |
| OTU96 | NA | NA | NA | NA | NA | NA |
| OTU91 | NA | NA | NA | NA | NA | NA |
| OTU6563 | NA | NA | NA | NA | NA | + |
| OTU7452 | NA | NA | NA | NA | NA | + |
| OTU2302 | + | NA | NA | NA | NA | NA |
| OTU7498 | NA | NA | NA | NA | NA | NA |
| OTU8103 | NA | NA | + | NA | NA | NA |
| OTU4813 | NA | NA | NA | NA | + | NA |
| OTU5280 | NA | NA | NA | NA | NA | NA |
| OTU962 | NA | NA | NA | + | NA | NA |
| OTU6774 | NA | NA | NA | NA | NA | + |
| OTU2027 | + | NA | NA | NA | NA | NA |
| OTU554 | NA | NA | + | NA | NA | NA |
| OTU7073 | NA | NA | + | NA | NA | NA |
| OTU6547 | NA | NA | NA | NA | NA | NA |
| OTU5345 | NA | NA | NA | NA | + | NA |
| OTU4778 | NA | NA | NA | NA | + | NA |
| OTU2555 | NA | NA | NA | NA | NA | NA |
| OTU2520 | NA | NA | + | NA | NA | NA |
| OTU45 | NA | NA | + | NA | NA | NA |
| OTU40 | NA | NA | NA | NA | NA | + |
| OTU4880 | + | NA | NA | NA | NA | NA |
| OTU5864 | NA | NA | NA | NA | NA | NA |
| OTU4276 | NA | NA | NA | + | NA | NA |
| OTU638 | NA | NA | + | NA | NA | NA |
| OTU631 | NA | NA | + | NA | NA | NA |
| OTU2285 | NA | NA | NA | + | NA | NA |
| OTU600 | NA | NA | + | NA | NA | NA |
| OTU636 | NA | NA | + | NA | NA | NA |
| OTU8209 | + | NA | NA | NA | NA | NA |
| OTU1769 | NA | NA | + | NA | NA | NA |
| OTU1092 | + | NA | NA | NA | NA | NA |
| OTU528 | NA | NA | + | NA | NA | NA |
| OTU6081 | NA | NA | NA | + | NA | NA |
| OTU2918 | NA | NA | NA | + | NA | NA |
| OTU7871 | + | NA | NA | NA | NA | NA |
| OTU5036 | NA | NA | + | NA | NA | NA |
| OTU3665 | NA | NA | NA | + | NA | NA |
| OTU820 | NA | NA | NA | NA | NA | NA |
| OTU4292 | NA | NA | NA | + | NA | NA |
| OTU2085 | NA | NA | NA | NA | NA | + |
| OTU1036 | NA | NA | + | NA | NA | NA |
| OTU4073 | NA | NA | + | NA | NA | NA |
| OTU4787 | NA | NA | NA | NA | + | NA |
| OTU5060 | NA | + | NA | NA | NA | NA |
| OTU6258 | NA | + | NA | NA | NA | NA |
| OTU3950 | NA | NA | NA | NA | NA | NA |
| OTU4304 | NA | NA | NA | + | NA | NA |
| OTU5860 | NA | NA | NA | NA | NA | + |
| OTU5869 | NA | NA | NA | NA | NA | + |
| OTU5687 | + | + | NA | NA | NA | NA |
| OTU806 | NA | NA | + | NA | NA | NA |
| OTU4162 | NA | + | NA | NA | NA | NA |
| OTU88 | NA | NA | NA | + | NA | NA |
| OTU2166 | NA | NA | NA | NA | + | NA |
| OTU4628 | NA | NA | NA | NA | NA | NA |
| OTU4905 | NA | NA | NA | NA | + | NA |
| OTU4406 | + | NA | NA | NA | NA | NA |
| OTU5359 | NA | NA | + | NA | NA | NA |
| OTU3667 | + | NA | NA | NA | NA | NA |
| OTU3361 | NA | NA | NA | NA | NA | NA |
| OTU2265 | NA | NA | NA | NA | + | NA |
| OTU5944 | NA | + | + | NA | + | NA |
| OTU1912 | + | NA | NA | NA | NA | NA |
| OTU6676 | NA | NA | + | NA | NA | NA |
| OTU2665 | NA | NA | NA | NA | NA | NA |
| OTU7691 | NA | NA | NA | + | + | NA |
| OTU2181 | NA | NA | NA | + | NA | NA |
| OTU8194 | NA | NA | NA | NA | NA | NA |
| OTU3134 | + | + | NA | NA | NA | NA |
| OTU7072 | NA | NA | + | NA | NA | NA |
| OTU874 | NA | NA | NA | + | NA | NA |
| OTU1490 | NA | NA | NA | NA | NA | NA |
| OTU607 | NA | NA | + | NA | NA | NA |
| OTU832 | NA | NA | + | NA | NA | NA |
| OTU1043 | NA | NA | NA | + | NA | NA |
| OTU2219 | NA | NA | NA | NA | + | NA |
| OTU3288 | NA | NA | NA | NA | NA | + |
| OTU2709 | NA | NA | NA | NA | + | NA |
| OTU4436 | + | NA | NA | NA | NA | NA |
| OTU4489 | NA | NA | NA | NA | NA | NA |
| OTU5474 | + | NA | NA | NA | NA | NA |
| OTU4818 | NA | NA | NA | NA | + | NA |
| OTU7134 | NA | NA | + | NA | NA | NA |
| OTU4482 | NA | NA | NA | NA | NA | NA |
| OTU4485 | + | NA | NA | NA | NA | NA |
| OTU6569 | NA | NA | NA | NA | NA | + |
| OTU6568 | NA | NA | NA | NA | NA | + |
| OTU6561 | NA | NA | NA | NA | NA | + |
| OTU6566 | NA | NA | NA | NA | NA | + |
| OTU2187 | NA | NA | NA | NA | NA | NA |
| OTU4923 | + | NA | NA | NA | NA | NA |
| OTU3426 | NA | NA | NA | + | NA | NA |
| OTU3424 | NA | NA | NA | + | NA | NA |
| OTU5478 | + | NA | NA | NA | NA | NA |
| OTU5870 | NA | NA | NA | NA | NA | + |
| OTU5473 | + | NA | NA | NA | NA | NA |
| OTU2224 | NA | NA | NA | NA | NA | NA |
| OTU5647 | NA | NA | NA | + | + | NA |
| OTU1009 | NA | + | NA | NA | NA | NA |
| OTU3392 | NA | NA | NA | + | NA | NA |
| OTU3976 | NA | NA | NA | NA | NA | NA |
| OTU4011 | NA | + | NA | NA | NA | NA |
| OTU884 | NA | NA | NA | + | NA | NA |
| OTU3093 | NA | NA | NA | + | NA | NA |
| OTU3464 | NA | NA | NA | + | NA | NA |
| OTU6658 | NA | NA | NA | NA | NA | + |
| OTU889 | NA | NA | + | NA | NA | NA |
| OTU5837 | NA | NA | NA | NA | + | NA |
| OTU4817 | NA | NA | NA | NA | + | NA |
| OTU4257 | NA | NA | NA | + | NA | NA |
| OTU5853 | NA | NA | NA | NA | NA | + |
| OTU5852 | NA | NA | NA | NA | NA | + |
| OTU1883 | NA | NA | + | NA | NA | NA |
| OTU872 | NA | NA | + | NA | NA | + |
| OTU4596 | NA | NA | NA | NA | + | NA |
| OTU4779 | NA | NA | NA | NA | + | NA |
| OTU499 | NA | NA | + | NA | NA | NA |
| OTU3848 | NA | NA | NA | + | NA | NA |
| OTU408 | NA | NA | NA | + | NA | NA |
| OTU2138 | NA | NA | NA | NA | + | NA |
| OTU4165 | NA | + | NA | NA | NA | NA |
| OTU5108 | NA | NA | NA | NA | + | NA |
| OTU4305 | NA | NA | NA | + | NA | NA |
| OTU7612 | NA | + | NA | NA | NA | NA |
| OTU4160 | NA | NA | NA | NA | NA | NA |
| OTU2877 | NA | NA | NA | + | NA | NA |
| OTU2872 | NA | NA | NA | NA | NA | NA |
| OTU2871 | NA | NA | + | NA | NA | NA |
| OTU2841 | NA | NA | NA | NA | NA | NA |
| OTU6993 | NA | NA | NA | + | NA | NA |
| OTU5971 | NA | NA | NA | NA | NA | + |
| OTU4587 | NA | + | NA | NA | NA | NA |
| OTU8184 | NA | NA | NA | NA | NA | NA |
| OTU988 | NA | NA | + | NA | NA | NA |
| OTU6971 | NA | NA | NA | NA | NA | NA |
| OTU1307 | NA | NA | + | NA | NA | NA |
| OTU980 | NA | NA | NA | NA | NA | NA |
| OTU3018 | NA | NA | + | NA | NA | NA |
| OTU7026 | NA | NA | + | NA | NA | NA |
| OTU2503 | NA | NA | NA | NA | + | NA |
| OTU3642 | NA | NA | NA | NA | + | NA |
| OTU2785 | NA | NA | NA | NA | NA | + |
| OTU7058 | NA | NA | + | NA | NA | NA |
| OTU7056 | NA | NA | + | NA | NA | NA |
| OTU6759 | NA | NA | + | NA | NA | NA |
| OTU2742 | NA | NA | NA | NA | NA | NA |
| OTU3089 | NA | NA | NA | NA | NA | NA |
| OTU5303 | NA | NA | NA | + | NA | NA |
| OTU6608 | NA | NA | + | NA | NA | NA |
| OTU61 | + | NA | NA | NA | NA | NA |
| OTU7088 | NA | NA | + | NA | NA | NA |
| OTU6387 | NA | NA | NA | NA | NA | + |
| OTU1964 | NA | NA | + | NA | NA | NA |
| OTU1781 | NA | NA | NA | NA | NA | NA |
| OTU4815 | NA | NA | NA | NA | + | NA |
| OTU5026 | + | NA | NA | NA | NA | NA |
| OTU4900 | + | NA | NA | NA | NA | NA |
| OTU5247 | NA | NA | NA | NA | + | NA |
| OTU1153 | NA | NA | + | NA | NA | NA |
| OTU30 | NA | NA | + | NA | NA | NA |
| OTU8143 | NA | NA | NA | + | NA | NA |
| OTU5917 | NA | NA | NA | NA | NA | + |
| OTU572 | NA | NA | NA | + | NA | NA |
| OTU7455 | + | NA | NA | NA | NA | NA |
| OTU5675 | NA | NA | NA | NA | NA | NA |
| OTU4538 | + | NA | NA | NA | NA | NA |
| OTU2121 | NA | NA | NA | + | NA | NA |
| OTU1670 | NA | NA | NA | NA | NA | NA |
| OTU1235 | NA | NA | + | NA | NA | NA |
| OTU2129 | NA | NA | NA | + | NA | NA |
| OTU7301 | NA | NA | + | NA | NA | NA |
| OTU244 | NA | NA | NA | NA | NA | NA |
| OTU757 | NA | NA | NA | NA | NA | NA |
| OTU1392 | NA | NA | NA | NA | NA | NA |
| OTU1395 | NA | NA | + | NA | NA | NA |
| OTU3740 | NA | NA | NA | NA | NA | NA |
| OTU7517 | NA | NA | NA | NA | NA | + |
| OTU7514 | NA | NA | NA | NA | NA | + |
| OTU6344 | NA | NA | + | NA | NA | NA |
| OTU5874 | NA | NA | NA | NA | NA | NA |
| OTU7519 | NA | NA | NA | NA | NA | NA |
| OTU7518 | NA | NA | NA | NA | NA | NA |
|  |  |  |  |  |  |  |

**Table S3.** Significant associations of soil fungi with the six types of communities (P ≤ 0.05 level of significance for torus-translation test). QAV, QSV, LAG, PIA, FOS, and SOH represent *Quercus aliena* var. *acutiserrata*, *Quercus serrata* var. *brevipetiolata*, *Larix gmelinii*, *Pinus armandii*, *Forsythia suspensa*, and *Sorbus hupehensis*, respectively. NA represents no significant correlation. (+) indicates positive correlation.

| Species | Community | | | | | |
| --- | --- | --- | --- | --- | --- | --- |
|  | QAV | QSV | LAG | PIA | FOS | SOH |
| OTU270 | NA | NA | + | NA | NA | NA |
| OTU6325 | NA | NA | NA | + | NA | NA |
| OTU4608 | NA | + | + | NA | NA | NA |
| OTU1342 | NA | NA | NA | NA | NA | + |
| OTU4605 | NA | + | NA | + | NA | NA |
| OTU2468 | + | NA | NA | + | NA | NA |
| OTU4279 | NA | NA | NA | NA | NA | NA |
| OTU122 | NA | NA | NA | NA | + | NA |
| OTU6917 | NA | NA | NA | NA | NA | NA |
| OTU6740 | NA | NA | NA | NA | NA | + |
| OTU236 | NA | NA | NA | + | NA | NA |
| OTU7142 | NA | NA | + | NA | NA | NA |
| OTU2909 | NA | NA | + | NA | NA | NA |
| OTU3108 | NA | NA | NA | + | NA | NA |
| OTU1567 | NA | NA | NA | NA | + | NA |
| OTU4365 | NA | NA | NA | + | NA | NA |
| OTU4590 | NA | NA | NA | + | NA | NA |
| OTU5173 | NA | NA | NA | NA | + | NA |
| OTU4340 | NA | NA | + | NA | NA | NA |
| OTU7671 | NA | NA | NA | NA | NA | NA |
| OTU1177 | + | NA | NA | NA | NA | NA |
| OTU4507 | + | NA | NA | NA | NA | NA |
| OTU5485 | + | NA | NA | NA | NA | NA |
| OTU5114 | NA | NA | NA | + | NA | NA |
| OTU6896 | NA | NA | NA | + | NA | NA |
| OTU6593 | NA | NA | NA | + | NA | NA |
| OTU3193 | NA | NA | NA | NA | + | NA |
| OTU6911 | NA | NA | NA | + | NA | NA |
| OTU2533 | + | NA | NA | NA | NA | NA |
| OTU6433 | NA | NA | NA | + | NA | NA |
| OTU5728 | NA | + | NA | NA | NA | NA |
| OTU1966 | NA | NA | NA | NA | NA | + |
| OTU2561 | NA | NA | NA | NA | NA | NA |
| OTU3927 | NA | NA | NA | NA | + | NA |
| OTU7652 | NA | NA | NA | NA | NA | NA |
| OTU3400 | NA | NA | NA | NA | NA | NA |
| OTU3403 | NA | NA | NA | + | NA | NA |
| OTU2428 | NA | + | NA | + | NA | NA |
| OTU3154 | NA | NA | NA | NA | NA | NA |
| OTU2373 | NA | NA | + | NA | NA | NA |
| OTU871 | NA | NA | + | NA | NA | NA |
| OTU5213 | NA | NA | NA | NA | + | NA |
| OTU5584 | + | NA | NA | NA | NA | NA |
| OTU5564 | NA | NA | NA | + | NA | NA |
| OTU911 | NA | NA | + | NA | NA | NA |
| OTU7340 | NA | + | NA | NA | NA | NA |
| OTU4254 | NA | NA | NA | NA | NA | NA |
| OTU1176 | NA | + | NA | NA | NA | NA |
| OTU1832 | NA | NA | NA | NA | NA | NA |
| OTU2059 | NA | NA | NA | + | NA | NA |
| OTU1831 | + | NA | NA | NA | NA | NA |
| OTU377 | NA | + | NA | NA | NA | NA |
| OTU2157 | NA | NA | NA | NA | + | NA |
| OTU380 | NA | NA | NA | NA | NA | NA |
| OTU8260 | NA | NA | + | NA | NA | NA |
| OTU5251 | NA | NA | NA | NA | + | NA |
| OTU824 | NA | NA | NA | NA | NA | NA |
| OTU674 | NA | NA | + | NA | NA | NA |
| OTU1963 | NA | NA | + | NA | NA | NA |
| OTU3970 | NA | NA | NA | + | NA | NA |
| OTU742 | NA | NA | NA | + | NA | NA |
| OTU269 | NA | NA | NA | + | NA | + |
| OTU2589 | + | NA | NA | NA | NA | NA |
| OTU4451 | + | NA | NA | NA | NA | NA |
| OTU4458 | + | NA | NA | NA | + | NA |
| OTU7587 | NA | NA | NA | NA | NA | + |
| OTU7566 | NA | NA | NA | NA | NA | NA |
| OTU2269 | NA | NA | + | NA | NA | NA |
| OTU3585 | NA | NA | NA | NA | NA | NA |
| OTU3042 | NA | NA | NA | NA | NA | NA |
| OTU3355 | NA | NA | NA | + | NA | NA |
| OTU633 | NA | NA | + | NA | NA | NA |
| OTU3076 | NA | NA | NA | + | NA | NA |
| OTU8221 | NA | NA | + | NA | NA | NA |
| OTU1442 | NA | NA | + | NA | NA | NA |
| OTU3389 | NA | NA | NA | + | NA | NA |
| OTU7720 | NA | NA | NA | NA | NA | + |
| OTU7231 | NA | NA | + | NA | NA | NA |
| OTU7236 | NA | + | NA | NA | + | NA |
| OTU4743 | NA | NA | NA | NA | + | NA |
| OTU700 | NA | NA | NA | + | NA | NA |
| OTU7084 | NA | NA | + | NA | NA | NA |
| OTU3864 | NA | NA | NA | NA | + | NA |
| OTU549 | NA | NA | + | NA | NA | NA |
| OTU5150 | + | NA | NA | NA | NA | NA |
| OTU4729 | NA | NA | NA | NA | + | NA |
| OTU5906 | NA | NA | NA | NA | + | NA |
| OTU6598 | NA | NA | NA | NA | NA | NA |
| OTU727 | NA | NA | + | NA | NA | NA |
| OTU4387 | + | NA | NA | + | NA | NA |
| OTU5685 | NA | NA | NA | + | NA | + |
| OTU288 | NA | NA | + | NA | NA | NA |
| OTU2144 | NA | NA | NA | NA | + | NA |
| OTU4838 | + | NA | NA | NA | NA | NA |
| OTU506 | NA | NA | + | NA | NA | NA |
| OTU4978 | NA | NA | NA | NA | + | NA |
| OTU1098 | + | NA | + | NA | NA | NA |
| OTU5162 | + | NA | NA | NA | NA | NA |
| OTU1805 | + | NA | NA | NA | NA | NA |
| OTU2952 | NA | NA | + | NA | NA | NA |
| OTU3509 | NA | NA | + | NA | NA | NA |
| OTU6921 | NA | NA | NA | + | NA | NA |
| OTU5043 | + | NA | NA | NA | NA | NA |
| OTU616 | NA | + | NA | NA | NA | NA |
| OTU6212 | NA | NA | + | NA | NA | NA |
| OTU2609 | NA | + | + | NA | NA | NA |
| OTU6068 | NA | + | NA | NA | NA | NA |
| OTU1528 | NA | NA | NA | NA | NA | NA |
| OTU1355 | NA | NA | NA | NA | NA | NA |
| OTU5875 | NA | NA | NA | NA | NA | + |
| OTU1353 | NA | NA | + | NA | NA | NA |
| OTU6772 | NA | NA | NA | NA | NA | + |
| OTU7658 | NA | + | NA | NA | NA | NA |
| OTU4187 | NA | + | NA | NA | NA | NA |
| OTU3777 | + | NA | NA | NA | NA | NA |
| OTU6113 | NA | NA | + | NA | NA | NA |
| OTU4101 | NA | NA | NA | NA | NA | + |
| OTU3676 | NA | NA | + | NA | NA | NA |
| OTU4369 | NA | NA | NA | NA | NA | + |
| OTU3070 | NA | NA | NA | NA | NA | + |
| OTU3222 | NA | NA | + | NA | NA | NA |
| OTU3221 | NA | NA | NA | + | NA | NA |
| OTU8006 | NA | NA | NA | NA | NA | NA |
| OTU2567 | NA | NA | NA | NA | + | NA |
| OTU5863 | NA | NA | NA | + | NA | + |
| OTU1639 | NA | NA | NA | NA | NA | + |
| OTU1149 | NA | + | NA | NA | NA | NA |
| OTU3022 | NA | + | NA | NA | NA | NA |
| OTU1632 | NA | + | NA | NA | NA | NA |
| OTU378 | NA | NA | + | NA | NA | NA |
| OTU1939 | NA | NA | + | NA | NA | NA |
| OTU5053 | + | NA | NA | NA | NA | NA |
| OTU4741 | NA | NA | NA | NA | + | NA |
| OTU1616 | + | NA | NA | NA | NA | NA |
| OTU5605 | NA | NA | NA | NA | NA | NA |
| OTU5490 | + | NA | NA | NA | NA | NA |
| OTU223 | NA | NA | + | NA | NA | NA |
| OTU6918 | NA | NA | NA | NA | NA | + |
| OTU4523 | + | NA | NA | NA | NA | NA |
| OTU6998 | NA | NA | NA | NA | NA | NA |
| OTU7991 | + | NA | NA | NA | NA | NA |
| OTU6651 | NA | NA | NA | NA | NA | NA |
| OTU3425 | NA | NA | NA | + | NA | NA |
| OTU1897 | NA | + | NA | NA | NA | NA |
| OTU2595 | NA | + | NA | NA | NA | NA |
| OTU2599 | NA | + | NA | NA | NA | NA |
| OTU1200 | NA | NA | NA | NA | NA | NA |
| OTU5110 | + | NA | NA | NA | NA | NA |
| OTU1169 | NA | NA | + | NA | NA | NA |
| OTU2315 | NA | NA | NA | NA | + | NA |
| OTU7066 | NA | NA | + | NA | NA | NA |
| OTU6627 | NA | NA | + | NA | NA | NA |
| OTU4848 | + | NA | NA | NA | NA | NA |
| OTU7036 | NA | NA | + | NA | NA | NA |
| OTU643 | NA | NA | + | NA | NA | NA |
| OTU257 | NA | NA | NA | + | NA | NA |
| OTU3364 | NA | NA | NA | + | NA | NA |
| OTU3368 | NA | NA | NA | + | NA | NA |
| OTU142 | NA | NA | + | NA | NA | NA |
| OTU550 | NA | NA | + | NA | NA | NA |
| OTU5709 | + | NA | NA | NA | NA | NA |
| OTU1463 | NA | NA | NA | + | NA | NA |
| OTU2201 | NA | NA | NA | + | NA | NA |
| OTU3460 | NA | NA | NA | + | NA | NA |
| OTU5532 | NA | NA | NA | NA | + | NA |
| OTU350 | NA | NA | NA | NA | NA | NA |
| OTU4733 | NA | + | NA | NA | NA | NA |
| OTU5466 | NA | NA | NA | NA | NA | NA |
| OTU5467 | + | NA | NA | NA | NA | NA |
| OTU3636 | NA | NA | NA | NA | + | NA |
| OTU5495 | + | NA | NA | NA | NA | NA |
| OTU3754 | NA | + | NA | NA | NA | NA |
| OTU276 | NA | NA | + | NA | NA | NA |
| OTU3408 | NA | NA | NA | + | NA | NA |
| OTU3034 | NA | NA | + | NA | NA | NA |
| OTU5470 | + | NA | NA | NA | NA | NA |
| OTU8219 | NA | NA | + | NA | NA | NA |
| OTU2548 | + | NA | NA | NA | NA | NA |
| OTU5393 | NA | NA | + | NA | NA | NA |
| OTU5826 | NA | NA | NA | NA | NA | NA |
| OTU4376 | NA | NA | NA | + | NA | NA |
| OTU4669 | NA | NA | + | NA | NA | NA |
| OTU5166 | NA | NA | NA | NA | + | NA |
| OTU6755 | NA | NA | + | NA | NA | NA |
| OTU230 | NA | + | NA | NA | NA | NA |
| OTU7598 | NA | NA | NA | NA | NA | NA |
| OTU5228 | NA | NA | NA | NA | + | NA |
| OTU1465 | NA | NA | + | NA | NA | NA |
| OTU546 | NA | NA | + | NA | NA | NA |
| OTU7391 | NA | NA | NA | + | NA | NA |
| OTU4282 | NA | + | NA | NA | NA | NA |
| OTU6126 | NA | NA | + | NA | NA | NA |
| OTU4521 | + | NA | NA | NA | NA | NA |
| OTU2894 | NA | NA | + | NA | NA | NA |
| OTU5070 | NA | + | NA | NA | NA | NA |
| OTU3287 | NA | NA | + | NA | NA | NA |
| OTU7707 | NA | NA | NA | NA | NA | + |
| OTU5305 | NA | NA | NA | NA | NA | NA |
| OTU4470 | NA | NA | NA | NA | NA | NA |
| OTU1262 | NA | NA | NA | NA | NA | NA |
| OTU2431 | NA | NA | NA | + | NA | NA |
| OTU4476 | NA | NA | NA | NA | NA | + |
| OTU1705 | + | NA | NA | NA | NA | NA |
| OTU7636 | NA | NA | + | NA | NA | NA |
| OTU2624 | NA | NA | NA | + | NA | NA |
| OTU6311 | + | NA | NA | NA | + | NA |
| OTU4433 | NA | + | NA | NA | NA | NA |
| OTU7010 | NA | NA | NA | + | NA | NA |
| OTU1600 | NA | NA | NA | NA | NA | NA |
| OTU7015 | NA | NA | + | NA | NA | NA |
| OTU6022 | NA | NA | NA | + | NA | NA |
| OTU4290 | NA | NA | NA | + | NA | NA |
| OTU3058 | NA | NA | NA | + | NA | NA |
| OTU717 | NA | NA | NA | NA | NA | NA |
| OTU3381 | NA | NA | NA | + | NA | NA |
| OTU4739 | + | NA | NA | NA | NA | NA |
| OTU5348 | NA | NA | + | NA | NA | NA |
| OTU3176 | NA | NA | NA | + | NA | NA |
| OTU7611 | NA | NA | NA | + | NA | NA |
| OTU4790 | + | NA | NA | NA | NA | NA |
| OTU827 | + | NA | NA | NA | NA | NA |
| OTU7089 | NA | NA | + | NA | NA | NA |
| OTU6054 | NA | NA | NA | + | NA | NA |
| OTU5824 | NA | + | NA | + | NA | + |
| OTU4326 | NA | NA | NA | NA | NA | NA |
| OTU4398 | + | NA | NA | NA | NA | NA |
| OTU893 | NA | NA | NA | NA | NA | NA |
| OTU7122 | NA | NA | NA | NA | NA | + |
| OTU4474 | NA | NA | NA | NA | NA | NA |
| OTU4039 | + | NA | NA | NA | NA | NA |
| OTU5970 | NA | NA | NA | NA | NA | NA |
| OTU7223 | NA | NA | + | NA | NA | NA |
| OTU5245 | NA | NA | NA | NA | + | NA |
| OTU7103 | NA | NA | + | NA | NA | NA |
| OTU1115 | NA | NA | NA | + | NA | NA |
| OTU7008 | NA | NA | NA | NA | NA | + |
| OTU5725 | NA | + | NA | NA | NA | NA |
| OTU4471 | + | NA | NA | NA | NA | NA |
| OTU672 | NA | NA | NA | NA | NA | NA |
| OTU1428 | NA | + | NA | NA | NA | NA |
| OTU4203 | NA | + | NA | NA | NA | NA |
| OTU2659 | NA | NA | NA | + | NA | NA |
| OTU7876 | NA | NA | + | NA | NA | NA |
| OTU8192 | NA | NA | NA | NA | NA | + |
| OTU4172 | NA | + | NA | NA | NA | NA |
| OTU4418 | NA | NA | NA | NA | NA | NA |
| OTU722 | NA | NA | NA | NA | NA | NA |
| OTU4255 | NA | NA | NA | + | NA | NA |
| OTU3407 | NA | NA | NA | + | NA | NA |
| OTU6912 | NA | NA | NA | + | NA | NA |
| OTU5483 | + | NA | NA | NA | NA | NA |
| OTU4472 | + | NA | NA | NA | NA | NA |
| OTU249 | NA | NA | NA | NA | NA | NA |
| OTU1462 | NA | NA | NA | + | NA | NA |
| OTU5707 | + | NA | NA | NA | NA | NA |
| OTU2482 | NA | NA | NA | + | NA | NA |
| OTU154 | NA | + | NA | NA | NA | NA |
| OTU5522 | NA | NA | NA | NA | + | NA |
| OTU6391 | NA | NA | NA | NA | NA | + |
| OTU6396 | NA | NA | NA | NA | NA | + |
| OTU6395 | NA | NA | NA | NA | NA | NA |
| OTU4508 | NA | NA | NA | NA | NA | NA |
| OTU5152 | NA | + | NA | NA | NA | NA |
| OTU4501 | + | NA | NA | NA | NA | NA |
| OTU7095 | NA | NA | + | NA | NA | NA |
| OTU5458 | NA | NA | NA | + | NA | NA |
| OTU3467 | NA | NA | NA | + | NA | NA |
| OTU1686 | NA | NA | + | NA | NA | NA |
| OTU5167 | + | NA | NA | NA | NA | NA |
| OTU6647 | NA | + | NA | NA | NA | NA |
| OTU4862 | + | NA | NA | NA | NA | NA |
| OTU4860 | + | NA | NA | NA | NA | NA |
| OTU4865 | + | NA | NA | NA | NA | NA |
| OTU4868 | + | NA | NA | NA | NA | NA |
| OTU77 | NA | NA | NA | NA | NA | NA |
| OTU3928 | NA | NA | NA | NA | NA | NA |
| OTU3612 | + | NA | NA | NA | NA | NA |
| OTU3617 | NA | + | NA | NA | NA | NA |
| OTU6312 | NA | NA | + | NA | NA | NA |
| OTU4280 | NA | + | NA | NA | NA | NA |
| OTU5272 | NA | NA | NA | NA | + | NA |
| OTU5903 | NA | NA | NA | NA | NA | + |
| OTU4637 | + | NA | NA | NA | NA | NA |
| OTU2939 | NA | NA | NA | NA | NA | NA |
| OTU2938 | NA | NA | NA | NA | + | NA |
| OTU7590 | NA | NA | NA | NA | + | NA |
| OTU5295 | NA | NA | NA | NA | NA | NA |
| OTU5194 | + | NA | NA | NA | NA | NA |
| OTU7208 | NA | NA | NA | NA | NA | NA |
| OTU6108 | NA | NA | NA | NA | NA | NA |
| OTU4454 | + | NA | NA | NA | NA | NA |
| OTU626 | NA | NA | + | NA | NA | NA |
| OTU3406 | NA | NA | NA | + | NA | NA |
| OTU1656 | NA | NA | NA | NA | NA | NA |
| OTU4646 | NA | NA | NA | NA | + | NA |
| OTU4135 | NA | NA | + | NA | NA | NA |
| OTU7654 | NA | NA | + | NA | NA | NA |
| OTU2427 | NA | NA | + | NA | NA | NA |
| OTU275 | NA | NA | + | NA | NA | NA |
| OTU3820 | NA | NA | + | NA | NA | NA |
| OTU250 | NA | + | NA | NA | NA | NA |
| OTU4024 | NA | NA | NA | + | NA | NA |
| OTU5831 | NA | + | NA | NA | NA | NA |
| OTU3554 | NA | NA | NA | + | + | NA |
| OTU5492 | + | NA | NA | NA | NA | NA |
| OTU49 | NA | NA | NA | NA | + | NA |
| OTU7080 | NA | NA | + | NA | NA | NA |
| OTU3081 | NA | NA | NA | NA | NA | NA |
| OTU346 | NA | NA | + | NA | NA | NA |
| OTU7258 | NA | NA | + | NA | NA | NA |
| OTU7111 | NA | NA | + | NA | NA | NA |
| OTU1679 | NA | NA | + | NA | NA | NA |
| OTU1601 | NA | NA | NA | NA | + | NA |
| OTU1315 | NA | NA | NA | + | NA | NA |
| OTU3535 | NA | NA | NA | NA | NA | + |
| OTU4396 | NA | NA | NA | + | NA | NA |
| OTU1481 | NA | NA | NA | + | NA | NA |
| OTU173 | NA | NA | NA | NA | NA | + |
| OTU959 | NA | NA | + | NA | NA | NA |
| OTU135 | NA | NA | NA | + | NA | NA |
| OTU6550 | NA | NA | NA | NA | NA | NA |
| OTU6097 | NA | NA | + | NA | NA | NA |
| OTU4020 | NA | NA | NA | NA | + | NA |
| OTU8229 | NA | NA | + | NA | NA | NA |
| OTU8228 | NA | NA | NA | NA | NA | NA |
| OTU1737 | NA | NA | NA | NA | NA | NA |
| OTU7928 | + | NA | NA | NA | NA | NA |
| OTU1052 | NA | NA | + | NA | NA | NA |
| OTU8226 | NA | NA | NA | + | NA | NA |
| OTU4759 | NA | NA | NA | NA | + | NA |
| OTU5434 | NA | NA | NA | NA | + | NA |
| OTU7870 | + | NA | NA | NA | NA | NA |
| OTU3114 | NA | NA | + | NA | NA | NA |
| OTU3118 | NA | NA | NA | + | NA | NA |
| OTU3463 | NA | NA | NA | + | NA | NA |
| OTU2016 | + | NA | NA | NA | NA | NA |
| OTU3553 | NA | NA | NA | NA | NA | + |
| OTU6898 | NA | NA | NA | NA | NA | NA |
| OTU800 | NA | NA | NA | NA | NA | + |
| OTU7829 | NA | NA | NA | NA | NA | NA |
| OTU6250 | NA | NA | NA | NA | + | NA |
| OTU2394 | NA | NA | NA | NA | + | NA |
| OTU2395 | NA | NA | NA | NA | + | NA |
| OTU3379 | NA | NA | NA | NA | NA | NA |
| OTU5514 | NA | NA | + | NA | NA | NA |
| OTU1870 | NA | NA | + | NA | NA | NA |
| OTU1871 | NA | NA | + | NA | NA | NA |
| OTU5326 | NA | NA | NA | NA | NA | + |
| OTU5413 | NA | NA | NA | NA | + | NA |
| OTU2762 | NA | NA | NA | NA | + | NA |
| OTU163 | NA | NA | + | NA | NA | NA |
| OTU734 | NA | NA | + | NA | NA | NA |
| OTU5973 | NA | NA | NA | NA | NA | + |
| OTU2015 | + | NA | NA | NA | NA | NA |
| OTU317 | NA | NA | + | NA | NA | NA |
| OTU3007 | NA | NA | + | NA | NA | NA |
| OTU129 | + | NA | NA | NA | NA | NA |
| OTU4180 | NA | + | NA | NA | NA | NA |
| OTU679 | NA | NA | + | NA | NA | NA |
| OTU4577 | NA | NA | NA | NA | + | NA |
| OTU7477 | NA | NA | NA | NA | NA | + |
| OTU3801 | NA | NA | + | NA | NA | NA |
| OTU7856 | + | NA | NA | NA | NA | NA |
| OTU4981 | NA | NA | NA | NA | + | NA |
| OTU4625 | NA | + | NA | NA | NA | NA |
| OTU609 | NA | NA | + | NA | NA | NA |
| OTU4866 | NA | NA | + | NA | NA | NA |
| OTU4475 | NA | NA | NA | NA | NA | NA |
| OTU3972 | NA | NA | NA | NA | NA | + |
| OTU3478 | NA | NA | NA | + | NA | NA |
| OTU1407 | NA | NA | NA | NA | + | NA |
| OTU4159 | NA | + | NA | NA | NA | NA |
| OTU6055 | NA | NA | + | NA | NA | NA |
| OTU6910 | NA | NA | NA | + | NA | NA |
| OTU296 | NA | NA | + | NA | NA | NA |
| OTU3166 | NA | + | NA | NA | NA | NA |
| OTU958 | NA | NA | + | NA | NA | NA |
| OTU858 | NA | + | NA | NA | NA | NA |
| OTU3028 | NA | NA | + | NA | NA | NA |
| OTU2410 | NA | NA | + | NA | NA | NA |
| OTU1437 | NA | NA | + | NA | NA | NA |
| OTU3991 | NA | NA | NA | NA | NA | NA |
| OTU6332 | + | NA | NA | NA | NA | NA |
| OTU5882 | NA | NA | NA | NA | NA | + |
| OTU2042 | NA | NA | NA | NA | NA | + |
| OTU1113 | NA | NA | NA | NA | NA | NA |
| OTU5848 | + | NA | NA | NA | NA | NA |
| OTU337 | NA | NA | + | NA | NA | NA |
| OTU5233 | NA | NA | NA | NA | + | NA |
| OTU2704 | NA | NA | NA | NA | + | NA |
| OTU3186 | NA | NA | NA | NA | NA | NA |
| OTU7039 | NA | NA | + | NA | NA | NA |
| OTU4559 | NA | NA | NA | NA | NA | NA |
| OTU5161 | NA | NA | NA | NA | + | NA |
| OTU5160 | + | NA | NA | NA | NA | NA |
| OTU6004 | NA | NA | + | NA | NA | NA |
| OTU4065 | NA | NA | + | NA | NA | NA |
| OTU7556 | + | NA | NA | NA | NA | NA |
| OTU1623 | NA | NA | + | NA | NA | NA |
| OTU1152 | NA | NA | + | NA | NA | NA |
| OTU2727 | NA | NA | NA | NA | NA | NA |
| OTU984 | NA | NA | + | NA | NA | NA |
| OTU6802 | NA | NA | NA | NA | NA | NA |
| OTU4505 | + | NA | NA | NA | NA | NA |
| OTU7481 | NA | NA | NA | NA | NA | NA |
| OTU302 | NA | NA | + | NA | NA | NA |
| OTU6244 | NA | NA | NA | + | NA | NA |
| OTU1313 | NA | NA | + | NA | NA | NA |
| OTU4633 | NA | + | NA | NA | NA | NA |
| OTU5729 | NA | + | NA | NA | NA | NA |
| OTU592 | NA | NA | + | NA | NA | NA |
| OTU3832 | NA | NA | NA | NA | NA | NA |
| OTU1816 | NA | NA | NA | NA | NA | NA |
| OTU2312 | NA | NA | NA | NA | NA | NA |
| OTU7232 | NA | + | NA | NA | NA | NA |
| OTU3607 | + | NA | NA | NA | NA | NA |
| OTU2901 | NA | NA | NA | + | NA | NA |
| OTU2987 | NA | NA | + | NA | NA | NA |
| OTU2985 | NA | NA | + | NA | NA | NA |
| OTU2989 | NA | NA | + | NA | NA | NA |
| OTU3847 | + | NA | NA | NA | NA | NA |
| OTU6098 | NA | NA | + | NA | NA | NA |
| OTU7618 | NA | NA | NA | NA | NA | + |
| OTU1938 | + | NA | + | NA | NA | NA |
| OTU3396 | NA | NA | NA | + | NA | NA |
| OTU3895 | NA | NA | NA | NA | NA | NA |
| OTU3363 | NA | NA | NA | + | NA | NA |
| OTU7137 | NA | NA | + | NA | NA | NA |
| OTU7136 | NA | NA | + | NA | NA | NA |
| OTU2973 | NA | NA | + | NA | NA | NA |
| OTU3017 | NA | NA | + | NA | NA | NA |
| OTU5533 | + | NA | NA | NA | NA | NA |
| OTU3386 | NA | NA | NA | + | NA | NA |
| OTU1678 | NA | NA | + | NA | NA | NA |
| OTU3079 | NA | NA | NA | NA | NA | NA |
| OTU3746 | NA | NA | NA | NA | NA | NA |
| OTU209 | + | NA | + | NA | NA | NA |
| OTU3790 | NA | NA | NA | NA | NA | + |
| OTU2532 | NA | NA | NA | NA | NA | NA |
| OTU7712 | NA | NA | + | NA | NA | NA |
| OTU4921 | NA | NA | NA | NA | NA | NA |
| OTU3623 | + | NA | NA | NA | NA | NA |
| OTU4569 | NA | NA | NA | NA | + | NA |
| OTU2723 | NA | NA | NA | NA | NA | NA |
| OTU2399 | NA | + | NA | NA | NA | NA |
| OTU3691 | NA | NA | NA | NA | NA | NA |
| OTU4682 | NA | NA | NA | NA | NA | NA |
| OTU5124 | + | NA | NA | NA | NA | NA |
| OTU6666 | NA | NA | NA | NA | NA | + |
| OTU2967 | NA | NA | + | NA | NA | NA |
| OTU5861 | NA | NA | NA | + | NA | + |
| OTU4804 | + | NA | NA | NA | + | NA |
| OTU875 | NA | + | NA | NA | NA | NA |
| OTU4808 | NA | NA | NA | NA | + | NA |
| OTU4809 | NA | NA | NA | NA | + | NA |
| OTU3328 | NA | NA | NA | + | NA | NA |
| OTU3329 | NA | NA | NA | + | NA | NA |
| OTU2611 | + | NA | NA | NA | NA | NA |
| OTU4982 | + | NA | NA | NA | NA | NA |
| OTU6397 | NA | NA | NA | NA | NA | + |
| OTU7169 | NA | NA | + | NA | NA | NA |
| OTU7167 | NA | NA | + | NA | NA | NA |
| OTU560 | NA | NA | + | NA | NA | NA |
| OTU4423 | + | NA | NA | NA | NA | NA |
| OTU2237 | NA | NA | NA | + | NA | NA |
| OTU5575 | NA | + | NA | NA | NA | NA |
| OTU4544 | NA | NA | NA | NA | + | NA |
| OTU5329 | NA | NA | + | NA | NA | NA |
| OTU4296 | NA | NA | NA | + | NA | NA |
| OTU1505 | + | NA | NA | NA | NA | NA |
| OTU7381 | NA | NA | NA | NA | NA | + |
| OTU1227 | NA | NA | NA | + | NA | NA |
| OTU7123 | NA | NA | NA | NA | NA | NA |
| OTU521 | NA | NA | + | NA | NA | NA |
| OTU4598 | NA | NA | NA | NA | NA | NA |
| OTU4677 | + | NA | NA | NA | NA | NA |
| OTU2101 | + | NA | NA | NA | NA | NA |
| OTU6240 | NA | + | NA | NA | NA | NA |
| OTU143 | NA | NA | NA | NA | NA | + |
| OTU4597 | NA | NA | + | NA | NA | NA |
| OTU544 | NA | + | NA | NA | NA | NA |
| OTU2889 | + | NA | NA | NA | NA | NA |
| OTU3596 | NA | NA | + | NA | NA | NA |
| OTU4481 | + | NA | NA | NA | + | NA |
| OTU445 | NA | NA | NA | NA | NA | NA |
| OTU2204 | NA | NA | + | NA | NA | + |
| OTU3456 | NA | NA | NA | + | NA | NA |
| OTU7261 | NA | NA | + | NA | NA | NA |
| OTU7868 | NA | NA | NA | + | NA | NA |
| OTU2408 | NA | NA | + | NA | NA | NA |
| OTU6383 | NA | NA | NA | + | NA | NA |
| OTU7861 | NA | NA | NA | NA | NA | + |
| OTU588 | NA | NA | + | NA | NA | NA |
| OTU277 | NA | NA | + | NA | NA | NA |
| OTU278 | NA | + | NA | NA | NA | NA |
| OTU5481 | + | NA | NA | NA | NA | NA |
| OTU7244 | NA | NA | NA | NA | NA | NA |
| OTU7241 | NA | + | NA | NA | NA | NA |
| OTU2432 | NA | NA | NA | NA | NA | + |
| OTU4922 | + | NA | NA | NA | NA | NA |
| OTU3090 | NA | NA | NA | + | NA | NA |
| OTU3395 | NA | NA | NA | + | NA | NA |
| OTU3251 | NA | NA | + | NA | NA | NA |
| OTU6632 | NA | NA | NA | NA | NA | + |
| OTU3387 | NA | NA | NA | + | NA | NA |
| OTU1688 | NA | NA | NA | + | NA | NA |
| OTU4361 | NA | NA | NA | NA | NA | NA |
| OTU669 | NA | NA | + | NA | NA | NA |
| OTU3204 | NA | NA | + | NA | NA | NA |
| OTU300 | NA | NA | + | NA | NA | NA |
| OTU4414 | + | NA | NA | NA | NA | NA |
| OTU3791 | NA | NA | NA | + | NA | NA |
| OTU2322 | NA | NA | NA | + | NA | NA |
| OTU4511 | + | NA | NA | NA | NA | NA |
| OTU3572 | NA | NA | NA | + | + | NA |
| OTU5959 | NA | NA | NA | NA | + | NA |
| OTU1674 | NA | NA | NA | NA | NA | NA |
| OTU3271 | NA | NA | NA | NA | NA | + |
| OTU1687 | NA | NA | + | NA | + | NA |
| OTU4773 | NA | + | NA | NA | NA | NA |
| OTU4771 | + | NA | NA | NA | + | NA |
| OTU4959 | + | NA | NA | NA | NA | NA |
| OTU4774 | NA | NA | NA | NA | + | NA |
| OTU1374 | NA | + | NA | + | NA | NA |
| OTU6565 | NA | NA | NA | + | NA | NA |
| OTU3190 | NA | NA | + | NA | NA | NA |
| OTU5264 | NA | NA | NA | NA | NA | NA |
| OTU4500 | + | NA | NA | NA | NA | NA |
| OTU4355 | + | NA | NA | NA | NA | NA |
| OTU5454 | + | NA | NA | NA | NA | NA |
| OTU1645 | NA | NA | NA | + | NA | NA |
| OTU5463 | NA | NA | + | NA | NA | NA |
| OTU4561 | NA | NA | NA | NA | + | NA |
| OTU4567 | NA | NA | NA | NA | + | NA |
| OTU8015 | NA | + | NA | NA | NA | NA |
| OTU5327 | NA | NA | NA | + | NA | NA |
| OTU1731 | + | + | NA | NA | NA | NA |
| OTU4568 | NA | NA | NA | NA | + | NA |
| OTU7250 | NA | NA | NA | NA | NA | + |
| OTU5654 | + | NA | NA | NA | NA | NA |
| OTU8204 | NA | NA | NA | NA | NA | NA |
| OTU5652 | NA | NA | NA | NA | NA | NA |
| OTU2591 | + | NA | NA | NA | NA | + |
| OTU5268 | NA | NA | NA | NA | + | NA |
| OTU4460 | + | NA | NA | NA | NA | NA |
| OTU6888 | NA | NA | NA | NA | NA | + |
| OTU5580 | NA | + | NA | NA | NA | NA |
| OTU7718 | NA | NA | NA | NA | + | NA |
| OTU2218 | NA | + | NA | NA | NA | NA |
| OTU3409 | NA | NA | NA | + | NA | NA |
| OTU8223 | NA | NA | + | NA | NA | NA |
| OTU7812 | NA | NA | NA | + | NA | NA |
| OTU3907 | NA | NA | NA | + | NA | NA |
| OTU1598 | + | NA | NA | NA | NA | NA |
| OTU4337 | NA | NA | NA | + | NA | NA |
| OTU7152 | NA | NA | + | NA | NA | NA |
| OTU7838 | NA | + | NA | NA | NA | NA |
| OTU6699 | NA | NA | NA | NA | NA | NA |
| OTU2236 | NA | NA | NA | + | NA | NA |
| OTU3718 | NA | NA | NA | NA | NA | + |
| OTU2435 | + | NA | NA | NA | NA | NA |
| OTU4615 | NA | NA | NA | + | NA | NA |
| OTU1247 | NA | NA | + | NA | NA | NA |
| OTU1794 | NA | NA | + | NA | NA | NA |
| OTU2263 | NA | + | NA | NA | NA | NA |
| OTU481 | NA | NA | NA | NA | NA | + |
| OTU4136 | + | NA | NA | NA | NA | NA |
| OTU251 | NA | + | NA | NA | NA | NA |
| OTU4622 | NA | + | NA | NA | NA | NA |
| OTU2765 | NA | NA | NA | + | NA | NA |
| OTU5422 | NA | NA | NA | + | NA | NA |
| OTU4557 | + | NA | NA | NA | NA | NA |
| OTU7993 | NA | NA | NA | NA | + | NA |
| OTU5021 | NA | NA | NA | NA | + | NA |
| OTU110 | NA | NA | NA | + | NA | NA |
| OTU3481 | NA | NA | NA | NA | NA | NA |
| OTU2907 | + | NA | NA | NA | NA | NA |
| OTU6469 | NA | NA | + | NA | NA | NA |
| OTU5645 | + | NA | NA | NA | NA | NA |
| OTU6752 | NA | + | NA | NA | NA | NA |
| OTU192 | NA | + | NA | NA | NA | NA |
| OTU4230 | NA | NA | + | NA | NA | NA |
| OTU6689 | NA | NA | NA | NA | NA | + |
| OTU580 | NA | NA | + | NA | NA | NA |
| OTU6205 | NA | NA | NA | NA | NA | + |
| OTU1599 | NA | + | NA | NA | NA | NA |
| OTU4822 | NA | NA | NA | NA | NA | NA |
| OTU4823 | NA | NA | NA | NA | + | NA |
| OTU4239 | NA | + | NA | NA | NA | NA |
| OTU4446 | + | NA | NA | NA | NA | NA |
| OTU1611 | + | NA | NA | NA | NA | NA |
| OTU8043 | NA | NA | NA | NA | NA | + |
| OTU4308 | NA | NA | NA | + | NA | NA |
| OTU4824 | NA | NA | NA | + | + | NA |
| OTU602 | NA | NA | + | NA | NA | NA |
| OTU4820 | NA | NA | NA | + | NA | NA |
| OTU1932 | NA | NA | NA | NA | + | NA |
| OTU1936 | NA | NA | NA | + | NA | NA |
| OTU222 | NA | NA | + | NA | NA | NA |
| OTU1934 | + | NA | NA | NA | NA | NA |
| OTU1663 | NA | NA | NA | + | NA | NA |
| OTU3784 | NA | NA | NA | + | NA | NA |
| OTU3477 | NA | NA | NA | + | NA | NA |
| OTU1910 | NA | NA | + | NA | NA | NA |
| OTU35 | NA | NA | + | NA | NA | NA |
| OTU4594 | NA | NA | NA | NA | + | NA |
| OTU7723 | NA | NA | NA | + | NA | NA |
| OTU100 | + | NA | NA | NA | NA | NA |
| OTU3850 | NA | NA | + | NA | NA | NA |
| OTU6142 | NA | NA | NA | NA | NA | NA |
| OTU8081 | + | NA | NA | NA | NA | NA |
| OTU8084 | NA | NA | NA | NA | NA | + |
| OTU2911 | NA | NA | NA | + | NA | NA |
| OTU589 | NA | NA | + | NA | NA | NA |
| OTU5884 | NA | NA | NA | NA | NA | + |
| OTU952 | NA | NA | + | NA | NA | NA |
| OTU5880 | NA | NA | NA | NA | NA | + |
| OTU7843 | NA | NA | + | NA | NA | NA |
| OTU3903 | NA | NA | NA | NA | NA | NA |
| OTU4173 | NA | NA | NA | + | NA | NA |
| OTU1582 | NA | NA | NA | NA | NA | NA |
| OTU4524 | NA | NA | NA | NA | NA | NA |
| OTU4029 | + | NA | NA | NA | + | NA |
| OTU4455 | + | NA | NA | NA | + | NA |
| OTU6820 | + | NA | NA | NA | NA | NA |
| OTU5261 | NA | NA | NA | NA | + | NA |
| OTU3487 | NA | NA | NA | + | NA | NA |
| OTU1292 | NA | NA | + | NA | NA | NA |
| OTU8026 | NA | NA | NA | NA | NA | + |
| OTU1911 | NA | NA | + | NA | NA | NA |
| OTU297 | NA | NA | + | NA | NA | + |
| OTU5455 | NA | NA | NA | + | NA | NA |
| OTU3303 | NA | NA | NA | + | NA | NA |
| OTU5593 | NA | NA | NA | NA | + | NA |
| OTU7333 | NA | NA | + | NA | NA | NA |
| OTU2206 | NA | NA | NA | NA | NA | NA |
| OTU3675 | NA | NA | NA | + | NA | NA |
| OTU4571 | NA | NA | NA | NA | NA | NA |
| OTU394 | NA | NA | + | NA | NA | NA |
| OTU1273 | NA | NA | NA | NA | NA | + |
| OTU3744 | NA | NA | + | NA | NA | NA |
| OTU7158 | NA | NA | + | NA | NA | NA |
| OTU1755 | NA | NA | + | NA | NA | NA |
| OTU7155 | + | NA | NA | NA | NA | NA |
| OTU7154 | NA | NA | + | NA | NA | NA |
| OTU2465 | NA | NA | NA | NA | + | NA |
| OTU6248 | NA | + | NA | NA | NA | NA |
| OTU3819 | NA | NA | NA | NA | NA | NA |
| OTU3990 | NA | NA | NA | + | NA | NA |
| OTU1651 | NA | NA | + | NA | NA | NA |
| OTU4137 | NA | NA | NA | NA | + | NA |
| OTU6983 | NA | NA | NA | NA | NA | + |
| OTU2545 | NA | NA | NA | + | NA | NA |
| OTU4963 | + | NA | NA | NA | NA | NA |
| OTU7924 | + | NA | NA | NA | NA | NA |
| OTU3664 | + | NA | NA | NA | NA | NA |
| OTU6228 | NA | NA | NA | NA | + | NA |
| OTU5104 | NA | NA | NA | NA | NA | NA |
| OTU8093 | NA | NA | NA | NA | NA | + |
| OTU973 | NA | NA | + | NA | NA | NA |
| OTU3798 | NA | NA | NA | NA | NA | NA |
| OTU2753 | NA | + | NA | + | NA | NA |
| OTU4936 | NA | NA | NA | NA | + | NA |
| OTU5866 | NA | NA | NA | NA | NA | + |
| OTU7987 | NA | NA | NA | NA | NA | + |
| OTU7984 | NA | NA | NA | NA | NA | NA |
| OTU4447 | + | NA | NA | NA | NA | NA |
| OTU3285 | NA | NA | NA | + | NA | NA |
| OTU1105 | + | NA | + | NA | NA | NA |
| OTU6591 | + | NA | NA | NA | NA | NA |
| OTU2669 | NA | NA | NA | + | NA | NA |
| OTU6858 | NA | NA | NA | NA | NA | + |
| OTU5947 | NA | NA | + | NA | NA | NA |
| OTU6851 | NA | NA | NA | NA | NA | + |
| OTU2492 | NA | + | NA | NA | NA | NA |
| OTU2858 | NA | NA | + | NA | NA | NA |
| OTU5239 | + | NA | NA | NA | NA | NA |
| OTU5227 | NA | NA | NA | NA | + | NA |
| OTU5225 | + | NA | NA | NA | NA | NA |
| OTU5559 | NA | NA | NA | NA | NA | + |
| OTU5229 | NA | + | NA | NA | + | NA |
| OTU4405 | + | NA | NA | NA | NA | NA |
| OTU6859 | + | NA | NA | NA | NA | NA |
| OTU2962 | NA | NA | + | NA | NA | NA |
| OTU1602 | NA | NA | NA | NA | NA | + |
| OTU5431 | NA | NA | NA | NA | + | NA |
| OTU2594 | + | NA | NA | NA | NA | NA |
| OTU3244 | NA | NA | NA | NA | NA | NA |
| OTU5262 | NA | NA | NA | NA | NA | NA |
| OTU7159 | NA | NA | + | NA | NA | NA |
| OTU2002 | NA | NA | NA | + | NA | NA |
| OTU3182 | NA | NA | + | NA | NA | NA |
| OTU7308 | NA | NA | NA | + | NA | NA |
| OTU5606 | NA | NA | NA | NA | + | NA |
| OTU3849 | NA | NA | NA | + | NA | NA |
| OTU4258 | NA | NA | NA | + | NA | NA |
| OTU1220 | NA | NA | NA | + | NA | NA |
| OTU307 | NA | NA | NA | + | NA | NA |
| OTU3009 | NA | NA | + | NA | NA | NA |
| OTU6696 | NA | NA | NA | + | NA | + |
| OTU8063 | NA | NA | NA | NA | NA | NA |
| OTU4213 | NA | + | NA | NA | NA | NA |
| OTU8044 | + | NA | NA | NA | NA | NA |
| OTU6144 | NA | NA | NA | NA | NA | NA |
| OTU2168 | NA | NA | NA | NA | + | NA |
| OTU5941 | + | NA | NA | NA | NA | NA |
| OTU3258 | NA | NA | NA | NA | NA | NA |
| OTU3191 | NA | NA | NA | + | NA | NA |
| OTU2685 | + | NA | NA | + | NA | NA |
| OTU6963 | NA | NA | NA | + | NA | NA |
| OTU6573 | NA | NA | NA | NA | NA | + |
| OTU2052 | NA | + | NA | NA | NA | NA |
| OTU7764 | NA | NA | NA | + | NA | NA |
| OTU3785 | NA | NA | NA | + | NA | NA |
| OTU6562 | NA | NA | NA | NA | NA | + |
| OTU1330 | NA | NA | + | NA | NA | NA |
| OTU2050 | NA | NA | NA | NA | NA | NA |
| OTU6502 | + | NA | NA | NA | NA | NA |
| OTU1970 | + | NA | NA | NA | NA | NA |
| OTU4318 | + | NA | NA | NA | NA | NA |
| OTU1626 | NA | NA | + | NA | NA | NA |
| OTU4643 | NA | NA | NA | NA | NA | NA |
| OTU5672 | + | NA | NA | NA | NA | NA |
| OTU1557 | + | NA | NA | NA | NA | NA |
| OTU1655 | NA | + | NA | NA | NA | NA |
| OTU3393 | NA | NA | NA | + | NA | NA |
| OTU124 | + | NA | + | NA | NA | NA |
| OTU2783 | NA | + | NA | NA | NA | NA |
| OTU3589 | NA | + | NA | NA | NA | NA |
| OTU7573 | NA | NA | NA | + | NA | NA |
| OTU760 | NA | NA | NA | NA | NA | NA |
| OTU768 | NA | NA | + | NA | NA | NA |
| OTU503 | + | + | NA | NA | NA | NA |
| OTU5760 | + | NA | NA | NA | NA | NA |
| OTU6195 | NA | + | NA | NA | NA | NA |
| OTU42 | NA | NA | NA | + | NA | NA |
| OTU3208 | + | NA | NA | NA | NA | NA |
| OTU4540 | NA | NA | NA | NA | + | NA |
| OTU334 | NA | NA | + | NA | NA | NA |
| OTU738 | + | NA | NA | NA | NA | NA |
| OTU656 | NA | NA | + | NA | NA | NA |
| OTU4256 | NA | NA | NA | + | NA | NA |
| OTU3856 | NA | NA | + | NA | NA | NA |
| OTU3347 | NA | NA | NA | NA | + | NA |
| OTU3401 | + | NA | NA | NA | NA | NA |
| OTU7188 | NA | NA | NA | NA | + | NA |
| OTU2238 | NA | + | NA | NA | NA | NA |
| OTU2233 | + | NA | NA | NA | NA | NA |
| OTU4229 | NA | + | NA | NA | NA | NA |
| OTU2460 | NA | NA | NA | NA | NA | NA |
| OTU3672 | NA | NA | NA | + | NA | NA |
| OTU4367 | NA | NA | NA | NA | + | NA |
| OTU2205 | NA | + | NA | NA | NA | NA |
| OTU447 | NA | NA | + | NA | NA | NA |
| OTU3281 | NA | NA | + | NA | NA | NA |
| OTU8273 | NA | NA | NA | NA | NA | NA |
| OTU3514 | NA | NA | NA | NA | NA | NA |
| OTU7034 | NA | NA | + | NA | NA | NA |
| OTU1872 | NA | NA | NA | NA | NA | NA |
| OTU7173 | NA | NA | + | NA | NA | NA |
| OTU7175 | NA | NA | NA | NA | NA | NA |
| OTU7177 | NA | NA | + | NA | NA | NA |
| OTU7178 | NA | NA | NA | NA | NA | NA |
| OTU2759 | NA | NA | NA | NA | NA | NA |
| OTU7190 | NA | + | NA | NA | NA | NA |
| OTU5517 | + | NA | NA | NA | NA | NA |
| OTU5920 | NA | NA | NA | NA | NA | NA |
| OTU1168 | NA | NA | + | NA | NA | + |
| OTU3349 | NA | NA | NA | + | NA | NA |
| OTU5646 | NA | + | NA | NA | NA | NA |
| OTU5857 | NA | NA | NA | NA | NA | + |
| OTU6334 | NA | NA | NA | + | NA | NA |
| OTU179 | NA | NA | + | NA | NA | NA |
| OTU3402 | NA | NA | NA | + | NA | NA |
| OTU4186 | NA | + | NA | NA | NA | NA |
| OTU7756 | NA | NA | NA | NA | NA | + |
| OTU4445 | + | NA | NA | NA | NA | NA |
| OTU3995 | + | NA | NA | NA | NA | NA |
| OTU4796 | NA | NA | NA | NA | + | NA |
| OTU4599 | + | NA | NA | NA | NA | NA |
| OTU3602 | NA | NA | NA | NA | NA | NA |
| OTU892 | NA | NA | NA | NA | NA | NA |
| OTU5202 | + | NA | NA | NA | NA | NA |
| OTU4851 | + | NA | NA | NA | NA | NA |
| OTU1875 | NA | NA | NA | NA | NA | NA |
| OTU4373 | + | NA | NA | NA | + | NA |
| OTU4309 | NA | NA | NA | + | NA | NA |
| OTU4377 | NA | NA | NA | NA | + | NA |
| OTU7182 | NA | NA | + | NA | NA | NA |
| OTU511 | NA | NA | NA | NA | NA | NA |
| OTU4734 | + | NA | NA | NA | NA | NA |
| OTU7783 | NA | + | NA | NA | NA | NA |
| OTU725 | NA | + | NA | NA | NA | NA |
| OTU7794 | NA | NA | NA | NA | NA | + |
| OTU7791 | NA | NA | NA | + | NA | NA |
| OTU4425 | + | NA | NA | NA | NA | NA |
| OTU4157 | NA | + | NA | NA | NA | NA |
| OTU1064 | NA | NA | + | NA | NA | NA |
| OTU4151 | NA | + | + | NA | NA | NA |
| OTU4100 | NA | NA | NA | NA | + | NA |
| OTU1343 | NA | + | NA | + | NA | NA |
| OTU3350 | + | NA | NA | NA | NA | NA |
| OTU5912 | NA | NA | NA | NA | NA | NA |
| OTU3064 | NA | NA | NA | + | NA | NA |
| OTU2603 | NA | NA | NA | NA | + | NA |
| OTU7608 | NA | NA | NA | NA | NA | NA |
| OTU3772 | + | NA | NA | NA | NA | NA |
| OTU92 | NA | NA | + | NA | NA | NA |
| OTU5502 | NA | NA | NA | NA | + | NA |
| OTU4983 | NA | + | NA | NA | NA | NA |
| OTU7450 | + | NA | NA | NA | NA | NA |
| OTU4891 | + | NA | NA | NA | NA | NA |
| OTU7434 | NA | NA | NA | NA | NA | NA |
| OTU5147 | NA | + | NA | NA | NA | NA |
| OTU6175 | NA | NA | NA | NA | NA | + |
| OTU3035 | NA | NA | + | NA | NA | NA |
| OTU606 | NA | NA | + | NA | NA | NA |
| OTU4785 | NA | NA | NA | NA | + | NA |
| OTU3738 | NA | NA | NA | + | NA | NA |
| OTU4917 | + | NA | NA | NA | NA | NA |
| OTU1806 | NA | NA | + | NA | NA | NA |
| OTU1466 | NA | NA | NA | NA | NA | NA |
| OTU7926 | + | NA | NA | NA | NA | NA |
| OTU8066 | NA | NA | NA | NA | NA | NA |
| OTU6524 | NA | NA | NA | NA | NA | NA |
| OTU3277 | NA | NA | + | NA | NA | NA |
| OTU4301 | NA | NA | NA | + | NA | NA |
| OTU676 | NA | NA | + | NA | NA | NA |
| OTU1203 | NA | NA | NA | NA | NA | + |
| OTU4611 | NA | NA | NA | NA | NA | NA |
| OTU6164 | NA | NA | + | NA | NA | NA |
| OTU553 | NA | NA | NA | NA | NA | NA |
| OTU5106 | NA | NA | NA | NA | + | NA |
| OTU557 | NA | NA | + | NA | NA | NA |
| OTU559 | + | NA | + | NA | NA | NA |
| OTU4098 | NA | NA | NA | NA | + | NA |
| OTU2996 | NA | NA | + | NA | NA | NA |
| OTU687 | NA | NA | + | NA | NA | NA |
| OTU5411 | NA | NA | NA | NA | + | NA |
| OTU758 | NA | NA | NA | NA | NA | NA |
| OTU4532 | + | NA | NA | NA | NA | NA |
| OTU246 | NA | + | NA | NA | NA | NA |
| OTU1190 | + | NA | + | NA | NA | NA |
| OTU358 | NA | NA | NA | + | NA | NA |
| OTU3433 | NA | NA | NA | + | NA | NA |
| OTU8180 | NA | NA | + | NA | NA | NA |
| OTU8186 | NA | NA | + | NA | NA | + |
| OTU2149 | NA | NA | NA | NA | NA | + |
| OTU3536 | NA | NA | NA | + | NA | NA |
| OTU3538 | NA | NA | NA | + | NA | NA |
| OTU5694 | + | NA | NA | NA | NA | NA |
| OTU1753 | NA | NA | + | NA | NA | NA |
| OTU5488 | NA | NA | NA | NA | + | NA |
| OTU1750 | NA | NA | NA | + | NA | NA |
| OTU3453 | NA | NA | NA | + | NA | NA |
| OTU5491 | + | NA | NA | NA | NA | NA |
| OTU1673 | NA | NA | + | NA | NA | NA |
| OTU1499 | + | NA | NA | NA | NA | NA |
| OTU3131 | + | NA | NA | NA | NA | NA |
| OTU7965 | NA | NA | NA | + | NA | NA |
| OTU6317 | NA | + | NA | NA | NA | NA |
| OTU1979 | NA | NA | NA | NA | NA | NA |
| OTU8024 | NA | NA | NA | NA | NA | + |
| OTU7105 | NA | NA | + | NA | NA | NA |
| OTU6507 | NA | NA | NA | NA | NA | + |
| OTU2295 | NA | NA | NA | NA | NA | NA |
| OTU130 | NA | NA | NA | + | NA | NA |
| OTU2968 | NA | NA | + | NA | NA | NA |
| OTU5962 | NA | + | NA | NA | NA | NA |
| OTU3547 | NA | NA | NA | + | + | NA |
| OTU645 | NA | NA | + | NA | NA | NA |
| OTU2051 | NA | NA | NA | NA | NA | NA |
| OTU2252 | + | NA | NA | NA | NA | NA |
| OTU6337 | NA | NA | NA | NA | + | NA |
| OTU7437 | NA | NA | NA | NA | NA | + |
| OTU2957 | NA | NA | + | NA | NA | NA |
| OTU7113 | NA | NA | + | NA | NA | NA |
| OTU7110 | NA | NA | + | NA | NA | NA |
| OTU1609 | + | NA | NA | NA | NA | NA |
| OTU2978 | NA | NA | + | NA | NA | NA |
| OTU2946 | + | NA | NA | NA | NA | NA |
| OTU4316 | NA | NA | NA | + | NA | NA |
| OTU4313 | NA | NA | NA | + | NA | NA |
| OTU4530 | + | NA | NA | NA | NA | NA |
| OTU7030 | NA | NA | + | NA | NA | NA |
| OTU1940 | NA | NA | + | NA | NA | NA |
| OTU4314 | NA | NA | NA | + | NA | NA |
| OTU6984 | NA | NA | NA | NA | NA | + |
| OTU4311 | NA | NA | NA | + | NA | NA |
| OTU4312 | NA | NA | NA | + | NA | NA |
| OTU7474 | + | NA | NA | NA | NA | NA |
| OTU7475 | + | NA | NA | NA | NA | NA |
| OTU5192 | NA | NA | NA | NA | + | NA |
| OTU2856 | + | NA | NA | NA | NA | NA |
| OTU3146 | NA | NA | NA | NA | + | NA |
| OTU685 | NA | NA | + | NA | NA | NA |
| OTU517 | NA | NA | + | NA | NA | NA |
| OTU5557 | + | NA | NA | NA | NA | NA |
| OTU5885 | NA | NA | NA | NA | NA | + |
| OTU1565 | NA | NA | NA | NA | NA | NA |
| OTU5047 | NA | NA | + | NA | NA | NA |
| OTU3727 | NA | + | NA | NA | NA | + |
| OTU3054 | NA | NA | NA | + | NA | NA |
| OTU6612 | + | NA | NA | NA | NA | NA |
| OTU1892 | NA | NA | + | NA | NA | NA |
| OTU7944 | NA | NA | NA | + | NA | NA |
| OTU4857 | + | NA | NA | NA | NA | NA |
| OTU5386 | NA | NA | NA | + | NA | NA |
| OTU5730 | NA | + | NA | NA | NA | NA |
| OTU3783 | NA | NA | NA | + | NA | NA |
| OTU751 | + | NA | NA | NA | NA | NA |
| OTU3156 | + | NA | NA | NA | NA | NA |
| OTU7191 | NA | NA | NA | NA | NA | + |
| OTU7197 | NA | NA | NA | NA | NA | NA |
| OTU4736 | NA | NA | NA | NA | NA | NA |
| OTU7506 | NA | + | + | NA | NA | NA |
| OTU5130 | NA | + | NA | NA | NA | NA |
| OTU4586 | NA | NA | NA | NA | + | NA |
| OTU4495 | + | NA | NA | NA | NA | NA |
| OTU4588 | + | NA | NA | NA | NA | NA |
| OTU8036 | + | NA | NA | NA | NA | NA |
| OTU3736 | NA | NA | NA | NA | NA | NA |
| OTU5518 | NA | NA | NA | + | NA | NA |
| OTU5873 | NA | NA | NA | NA | NA | + |
| OTU4794 | NA | NA | + | NA | NA | NA |
| OTU4421 | + | NA | NA | NA | NA | NA |
| OTU2065 | NA | NA | NA | NA | NA | NA |
| OTU7746 | NA | NA | NA | NA | NA | + |
| OTU7632 | + | + | NA | NA | NA | NA |
| OTU6376 | NA | + | NA | NA | NA | NA |
| OTU5511 | NA | NA | NA | NA | NA | NA |
| OTU4806 | NA | NA | NA | + | + | NA |
| OTU5700 | + | NA | NA | NA | NA | NA |
| OTU4059 | NA | NA | NA | NA | NA | NA |
| OTU8261 | NA | NA | NA | + | NA | NA |
| OTU905 | NA | NA | NA | NA | NA | NA |
| OTU3429 | NA | NA | NA | + | NA | NA |
| OTU5459 | + | NA | NA | NA | NA | NA |
| OTU5530 | NA | NA | NA | NA | NA | NA |
| OTU4440 | + | NA | NA | NA | NA | NA |
| OTU5269 | NA | NA | NA | NA | + | NA |
| OTU5265 | NA | NA | NA | NA | + | NA |
| OTU5590 | NA | NA | NA | + | NA | NA |
| OTU4168 | NA | + | NA | NA | NA | NA |
| OTU6435 | NA | NA | NA | NA | NA | NA |
| OTU5844 | NA | NA | NA | NA | NA | + |
| OTU6002 | NA | NA | + | NA | NA | NA |
| OTU7119 | NA | NA | + | NA | NA | NA |
| OTU7112 | NA | NA | + | NA | NA | NA |
| OTU1138 | NA | NA | NA | + | NA | NA |
| OTU6319 | NA | NA | NA | + | NA | NA |
| OTU1135 | NA | NA | + | NA | NA | NA |
| OTU7343 | NA | + | NA | NA | NA | NA |
| OTU5373 | NA | NA | NA | NA | NA | NA |
| OTU7157 | NA | NA | NA | NA | NA | NA |
| OTU4479 | + | NA | NA | NA | NA | NA |
| OTU5387 | NA | NA | NA | + | NA | NA |
| OTU3164 | NA | + | NA | NA | + | NA |
| OTU3555 | NA | + | NA | NA | NA | NA |
| OTU3880 | NA | NA | NA | NA | NA | NA |
| OTU5955 | NA | NA | NA | + | NA | NA |
| OTU882 | NA | NA | NA | NA | + | NA |
| OTU4644 | NA | NA | NA | NA | NA | + |
| OTU3896 | NA | NA | + | NA | NA | NA |
| OTU5438 | + | NA | NA | NA | NA | NA |
| OTU2366 | NA | NA | + | NA | NA | NA |
| OTU7153 | NA | NA | + | NA | NA | NA |
| OTU6100 | NA | NA | NA | + | NA | NA |
| OTU4484 | + | NA | NA | NA | NA | NA |
| OTU3107 | NA | NA | NA | NA | NA | NA |
| OTU53 | NA | NA | + | NA | NA | NA |
| OTU6024 | NA | NA | + | NA | NA | NA |
| OTU3086 | NA | NA | NA | + | NA | NA |
| OTU7443 | + | NA | NA | NA | NA | NA |
| OTU1921 | NA | NA | NA | + | NA | NA |
| OTU3088 | NA | NA | NA | + | NA | NA |
| OTU2902 | NA | NA | + | NA | NA | NA |
| OTU6649 | NA | NA | + | NA | NA | NA |
| OTU2272 | NA | NA | NA | NA | + | NA |
| OTU5383 | NA | NA | NA | NA | + | NA |
| OTU2971 | NA | NA | + | NA | NA | NA |
| OTU5221 | NA | NA | NA | + | NA | NA |
| OTU2334 | NA | NA | NA | NA | NA | + |
| OTU4236 | NA | + | NA | NA | NA | NA |
| OTU2773 | NA | NA | NA | NA | NA | NA |
| OTU2303 | NA | NA | NA | NA | NA | + |
| OTU4871 | + | NA | NA | NA | NA | NA |
| OTU3113 | + | NA | NA | NA | NA | NA |
| OTU4295 | NA | NA | NA | + | NA | NA |
| OTU2107 | NA | NA | + | NA | NA | NA |
| OTU5901 | NA | NA | NA | NA | NA | + |
| OTU2109 | NA | NA | NA | NA | + | NA |
| OTU5908 | NA | NA | NA | NA | NA | + |
| OTU570 | NA | NA | + | NA | NA | NA |
| OTU5342 | NA | + | NA | NA | NA | NA |
| OTU6349 | NA | NA | + | NA | NA | NA |
| OTU5918 | NA | NA | NA | NA | NA | + |
| OTU4502 | + | NA | NA | NA | NA | NA |
| OTU4509 | + | NA | NA | NA | NA | NA |
| OTU750 | NA | NA | NA | NA | NA | NA |
| OTU8152 | NA | NA | + | NA | NA | NA |
| OTU4370 | NA | NA | NA | NA | NA | NA |
| OTU579 | NA | NA | + | NA | NA | NA |
| OTU576 | NA | NA | + | NA | NA | NA |
| OTU6889 | NA | NA | + | NA | + | NA |
| OTU3459 | NA | NA | NA | + | NA | NA |
| OTU2158 | NA | NA | NA | NA | NA | + |
| OTU7257 | NA | NA | + | NA | NA | NA |
| OTU7350 | NA | NA | NA | + | NA | NA |
| OTU2369 | NA | NA | + | NA | NA | NA |
| OTU5935 | NA | NA | NA | NA | + | NA |
| OTU6029 | + | NA | NA | NA | NA | NA |
| OTU4513 | NA | NA | NA | NA | NA | NA |
| OTU6597 | NA | NA | NA | NA | NA | + |
| OTU5802 | NA | NA | NA | NA | NA | + |
| OTU5702 | NA | NA | NA | NA | + | NA |
| OTU5705 | + | NA | NA | NA | NA | NA |
| OTU3548 | + | NA | NA | NA | NA | NA |
| OTU2344 | NA | NA | + | NA | NA | NA |
| OTU4287 | NA | NA | NA | + | NA | NA |
| OTU4607 | NA | NA | NA | NA | NA | NA |
| OTU4748 | NA | NA | NA | NA | + | NA |
| OTU6371 | NA | NA | NA | NA | NA | + |
| OTU6372 | NA | NA | NA | + | NA | NA |
| OTU5003 | + | NA | NA | NA | + | NA |
| OTU4072 | NA | + | + | NA | NA | NA |
| OTU3315 | NA | NA | NA | NA | + | NA |
| OTU1143 | NA | NA | + | NA | NA | NA |
| OTU3909 | + | NA | NA | NA | + | NA |
| OTU1867 | NA | NA | + | NA | NA | NA |
| OTU456 | + | NA | NA | NA | NA | NA |
| OTU4206 | + | NA | NA | NA | NA | NA |
| OTU2013 | NA | NA | NA | + | NA | NA |
| OTU6725 | NA | NA | + | NA | NA | NA |
| OTU2089 | NA | NA | NA | + | NA | NA |
| OTU4661 | + | NA | NA | NA | NA | NA |
| OTU1713 | NA | NA | + | NA | NA | NA |
| OTU6080 | NA | NA | NA | NA | NA | NA |
| OTU1908 | NA | NA | NA | + | NA | NA |
| OTU1443 | NA | NA | NA | + | NA | + |
| OTU7162 | NA | NA | + | NA | NA | NA |
| OTU5249 | NA | NA | NA | NA | + | NA |
| OTU109 | NA | NA | + | NA | NA | NA |
| OTU6417 | NA | NA | NA | NA | NA | + |
| OTU280 | NA | NA | + | NA | NA | NA |
| OTU4533 | + | NA | NA | NA | NA | NA |
| OTU4560 | NA | NA | NA | NA | + | NA |
| OTU4338 | NA | NA | NA | + | NA | NA |
| OTU4335 | NA | NA | NA | + | NA | NA |
| OTU4332 | NA | NA | NA | + | NA | NA |
| OTU4333 | NA | NA | NA | + | NA | NA |
| OTU556 | NA | NA | + | NA | NA | NA |
| OTU7750 | NA | NA | NA | NA | NA | NA |
| OTU3941 | NA | + | NA | NA | NA | NA |
| OTU3942 | NA | + | NA | NA | NA | NA |
| OTU3943 | NA | NA | NA | + | NA | NA |
| OTU3945 | NA | NA | NA | NA | NA | + |
| OTU766 | + | NA | NA | NA | NA | NA |
| OTU2731 | NA | NA | + | NA | NA | NA |
| OTU761 | NA | NA | NA | + | NA | NA |
| OTU6215 | NA | NA | NA | + | NA | NA |
| OTU4194 | NA | + | NA | NA | NA | NA |
| OTU4199 | NA | NA | NA | NA | NA | NA |
| OTU2491 | NA | NA | NA | NA | NA | + |
| OTU6726 | + | NA | NA | NA | NA | NA |
| OTU6564 | NA | NA | NA | NA | NA | + |
| OTU5068 | NA | + | NA | NA | NA | NA |
| OTU1010 | NA | NA | NA | + | NA | NA |
| OTU642 | NA | NA | + | NA | NA | NA |
| OTU5063 | NA | NA | + | NA | NA | NA |
| OTU6748 | NA | NA | NA | + | NA | NA |
| OTU6747 | NA | + | NA | NA | NA | NA |
| OTU2025 | NA | NA | NA | NA | NA | + |
| OTU3430 | NA | NA | NA | + | NA | NA |
| OTU4547 | NA | NA | NA | NA | + | NA |
| OTU598 | NA | NA | + | NA | NA | NA |
| OTU4402 | + | NA | NA | NA | NA | NA |
| OTU2340 | NA | NA | + | NA | NA | NA |
| OTU2733 | NA | + | NA | NA | NA | NA |
| OTU3437 | NA | NA | NA | + | NA | NA |
| OTU4624 | NA | + | NA | NA | NA | NA |
| OTU5191 | + | NA | NA | NA | NA | NA |
| OTU2167 | + | NA | NA | NA | NA | NA |
| OTU6914 | NA | NA | + | NA | NA | NA |
| OTU6874 | NA | NA | NA | NA | + | NA |
| OTU3719 | NA | + | + | NA | NA | NA |
| OTU516 | NA | NA | + | NA | NA | NA |
| OTU3711 | NA | NA | + | NA | NA | NA |
| OTU7098 | NA | NA | + | NA | NA | NA |
| OTU345 | NA | NA | + | NA | NA | NA |
| OTU5064 | + | NA | NA | NA | NA | NA |
| OTU240 | NA | NA | + | NA | NA | NA |
| OTU4792 | NA | NA | NA | NA | NA | NA |
| OTU8287 | NA | NA | + | NA | NA | NA |
| OTU2936 | + | NA | NA | NA | NA | NA |
| OTU2867 | NA | NA | + | NA | NA | NA |
| OTU3342 | NA | NA | NA | + | NA | NA |
| OTU3375 | NA | NA | NA | + | NA | NA |
| OTU1594 | NA | NA | NA | + | NA | NA |
| OTU8275 | NA | NA | + | NA | NA | NA |
| OTU4272 | NA | NA | NA | + | NA | NA |
| OTU4190 | NA | + | NA | NA | NA | NA |
| OTU441 | NA | + | NA | NA | NA | NA |
| OTU446 | NA | NA | + | NA | NA | NA |
| OTU945 | NA | NA | + | NA | NA | NA |
| OTU2564 | + | + | NA | NA | NA | NA |
| OTU2565 | NA | NA | NA | NA | NA | NA |
| OTU2214 | + | NA | NA | NA | NA | NA |
| OTU5724 | NA | + | NA | NA | NA | NA |
| OTU8127 | NA | NA | NA | + | NA | NA |
| OTU1761 | NA | NA | NA | NA | NA | NA |
| OTU552 | NA | NA | + | NA | NA | NA |
| OTU8232 | NA | NA | + | NA | NA | NA |
| OTU2790 | NA | NA | NA | NA | + | NA |
| OTU518 | NA | NA | + | NA | NA | NA |
| OTU1015 | NA | NA | NA | NA | NA | NA |
| OTU514 | NA | NA | NA | NA | NA | NA |
| OTU2470 | NA | NA | NA | + | NA | NA |
| OTU512 | NA | NA | + | NA | NA | NA |
| OTU5266 | NA | NA | NA | NA | + | NA |
| OTU5449 | + | NA | NA | NA | NA | NA |
| OTU3376 | NA | NA | NA | NA | NA | NA |
| OTU4452 | + | NA | NA | NA | NA | NA |
| OTU4910 | + | NA | NA | NA | NA | NA |
| OTU6110 | NA | NA | + | NA | NA | NA |
| OTU5015 | NA | + | NA | NA | NA | NA |
| OTU6103 | NA | NA | NA | NA | NA | NA |
| OTU4890 | NA | NA | NA | NA | NA | NA |
| OTU2023 | NA | NA | NA | NA | NA | + |
| OTU1458 | NA | NA | NA | + | NA | NA |
| OTU538 | NA | NA | NA | NA | NA | NA |
| OTU1186 | NA | NA | + | NA | NA | NA |
| OTU4664 | NA | NA | + | NA | NA | NA |
| OTU4434 | + | NA | NA | NA | NA | NA |
| OTU3574 | NA | NA | + | NA | NA | NA |
| OTU1994 | NA | NA | NA | NA | NA | + |
| OTU7326 | NA | + | + | NA | NA | NA |
| OTU4967 | + | NA | NA | NA | NA | NA |
| OTU1714 | NA | NA | + | NA | NA | NA |
| OTU1136 | NA | NA | + | NA | NA | + |
| OTU6358 | NA | NA | NA | NA | + | NA |
| OTU878 | NA | NA | NA | NA | NA | NA |
| OTU6603 | NA | NA | NA | NA | NA | NA |
| OTU4012 | + | NA | NA | NA | NA | NA |
| OTU3049 | NA | NA | NA | NA | NA | NA |
| OTU5287 | NA | NA | NA | NA | NA | NA |
| OTU5536 | + | NA | NA | NA | NA | NA |
| OTU3324 | NA | NA | NA | + | NA | NA |
| OTU2066 | NA | NA | + | NA | NA | NA |
| OTU3174 | + | NA | NA | NA | NA | NA |
| OTU2774 | NA | NA | NA | NA | NA | NA |
| OTU2148 | NA | NA | NA | NA | + | NA |
| OTU2298 | NA | NA | NA | NA | + | NA |
| OTU2650 | + | NA | NA | NA | + | NA |
| OTU3126 | NA | NA | NA | + | NA | + |
| OTU1937 | NA | + | NA | NA | NA | NA |
| OTU4264 | NA | NA | NA | + | NA | NA |
| OTU6350 | NA | NA | NA | NA | NA | + |
| OTU4015 | + | NA | NA | NA | NA | NA |
| OTU4620 | NA | + | NA | NA | NA | NA |
| OTU2904 | NA | NA | NA | + | NA | NA |
| OTU885 | NA | NA | NA | + | NA | NA |
| OTU5126 | + | NA | NA | NA | NA | NA |
| OTU2706 | NA | NA | + | NA | NA | NA |
| OTU3015 | NA | NA | + | NA | NA | NA |
| OTU318 | NA | NA | NA | NA | NA | + |
| OTU2925 | NA | NA | NA | NA | NA | + |
| OTU2922 | NA | NA | NA | NA | NA | + |
| OTU4310 | NA | NA | NA | + | NA | NA |
| OTU1430 | NA | + | NA | NA | NA | NA |
| OTU5206 | NA | NA | NA | NA | + | NA |
| OTU2879 | NA | + | NA | NA | NA | NA |
| OTU3104 | NA | NA | NA | + | NA | NA |
| OTU1744 | NA | NA | NA | NA | NA | NA |
| OTU831 | NA | NA | NA | NA | NA | NA |
| OTU571 | NA | NA | + | NA | NA | NA |
| OTU7398 | NA | NA | NA | + | NA | NA |
| OTU6401 | + | NA | NA | NA | NA | NA |
| OTU6605 | NA | NA | NA | + | NA | NA |
| OTU3966 | NA | NA | + | NA | NA | NA |
| OTU3965 | NA | NA | + | NA | NA | NA |
| OTU748 | NA | NA | NA | + | NA | NA |
| OTU749 | NA | NA | NA | + | NA | NA |
| OTU741 | + | NA | + | NA | NA | NA |
| OTU5125 | + | NA | NA | NA | NA | NA |
| OTU425 | NA | NA | + | NA | NA | NA |
| OTU5732 | NA | + | NA | NA | NA | NA |
| OTU7240 | NA | NA | + | NA | NA | NA |
| OTU4498 | + | NA | NA | NA | NA | NA |
| OTU5900 | NA | NA | NA | NA | NA | + |
| OTU1974 | NA | NA | + | NA | NA | NA |
| OTU4056 | NA | NA | NA | NA | + | NA |
| OTU6769 | NA | NA | + | NA | NA | NA |
| OTU4169 | NA | + | NA | NA | NA | NA |
| OTU6790 | NA | NA | NA | NA | NA | NA |
| OTU3388 | NA | NA | NA | + | NA | NA |
| OTU573 | NA | NA | NA | NA | NA | NA |
| OTU2402 | NA | NA | + | NA | NA | NA |
| OTU3385 | NA | NA | NA | + | NA | NA |
| OTU7400 | + | NA | NA | NA | NA | NA |
| OTU835 | NA | NA | NA | + | NA | NA |
| OTU1586 | NA | NA | NA | NA | + | NA |
| OTU8014 | NA | NA | + | NA | NA | NA |
| OTU1865 | NA | NA | NA | NA | + | NA |
| OTU1011 | NA | NA | + | NA | NA | NA |
| OTU4192 | NA | + | NA | NA | NA | NA |
| OTU4722 | NA | NA | NA | NA | NA | NA |
| OTU5299 | NA | NA | NA | + | NA | NA |
| OTU6585 | NA | NA | + | NA | NA | NA |
| OTU5498 | + | NA | NA | NA | NA | NA |
| OTU7282 | NA | NA | NA | + | NA | NA |
| OTU5887 | NA | NA | NA | NA | NA | + |
| OTU1391 | NA | NA | NA | + | NA | NA |
| OTU3397 | NA | NA | NA | + | NA | NA |
| OTU3533 | NA | NA | NA | NA | + | NA |
| OTU4344 | NA | NA | NA | NA | NA | NA |
| OTU1234 | NA | + | NA | NA | NA | NA |
| OTU1803 | NA | NA | + | NA | NA | NA |
| OTU4601 | NA | + | NA | NA | NA | NA |
| OTU1026 | NA | NA | NA | NA | NA | NA |
| OTU1572 | NA | NA | NA | NA | + | NA |
| OTU2588 | NA | NA | NA | + | NA | NA |
| OTU4836 | NA | NA | + | NA | NA | NA |
| OTU4837 | NA | NA | NA | NA | + | NA |
| OTU5804 | NA | NA | NA | NA | NA | NA |
| OTU6185 | NA | NA | + | NA | NA | NA |
| OTU6602 | NA | NA | + | NA | NA | NA |
| OTU8146 | NA | NA | + | NA | NA | NA |
| OTU3178 | + | NA | NA | NA | NA | NA |
| OTU5000 | + | + | NA | NA | + | NA |
| OTU5010 | NA | NA | NA | NA | + | NA |
| OTU1120 | NA | NA | NA | NA | NA | NA |
| OTU6063 | NA | NA | + | NA | NA | NA |
| OTU5496 | + | NA | NA | NA | NA | NA |
| OTU2803 | NA | NA | + | NA | NA | NA |
| OTU4712 | NA | + | NA | NA | NA | NA |
| OTU4713 | NA | NA | NA | NA | NA | NA |
| OTU4716 | NA | NA | NA | NA | NA | NA |
| OTU2956 | NA | NA | + | NA | NA | NA |
| OTU2882 | + | NA | NA | NA | NA | NA |
| OTU7130 | NA | NA | + | NA | NA | NA |
| OTU603 | NA | NA | + | NA | NA | NA |
| OTU3776 | NA | NA | + | NA | NA | NA |
| OTU7092 | NA | NA | + | NA | NA | NA |
| OTU4334 | NA | NA | NA | + | NA | NA |
| OTU3102 | NA | NA | NA | NA | NA | NA |
| OTU3779 | NA | NA | NA | + | NA | NA |
| OTU3373 | NA | NA | + | NA | NA | NA |
| OTU4196 | NA | + | NA | NA | NA | NA |
| OTU4146 | NA | + | NA | NA | NA | NA |
| OTU2424 | NA | NA | NA | + | NA | NA |
| OTU7575 | NA | NA | NA | NA | NA | NA |
| OTU670 | NA | NA | + | NA | NA | NA |
| OTU3534 | NA | NA | NA | NA | + | NA |
| OTU1672 | NA | NA | NA | + | NA | NA |
| OTU3597 | + | NA | NA | NA | NA | NA |
| OTU3595 | + | NA | NA | NA | NA | NA |
| OTU6165 | NA | NA | NA | NA | NA | NA |
| OTU1736 | NA | NA | + | NA | NA | NA |
| OTU8004 | NA | NA | NA | + | NA | NA |
| OTU3367 | NA | NA | NA | + | NA | NA |
| OTU4271 | NA | NA | NA | + | NA | NA |
| OTU4275 | NA | NA | NA | + | NA | NA |
| OTU4359 | NA | NA | NA | NA | NA | NA |
| OTU6723 | NA | NA | NA | NA | + | NA |
| OTU3673 | + | NA | NA | NA | NA | NA |
| OTU3296 | NA | NA | NA | NA | NA | NA |
| OTU1996 | + | NA | NA | NA | NA | NA |
| OTU7915 | NA | NA | NA | NA | NA | + |
| OTU2506 | NA | NA | NA | + | NA | NA |
| OTU2638 | NA | NA | + | NA | NA | NA |
| OTU5721 | NA | NA | NA | NA | + | NA |
| OTU2999 | NA | + | NA | NA | NA | NA |
| OTU2393 | NA | NA | + | NA | NA | NA |
| OTU432 | NA | NA | + | NA | NA | NA |
| OTU2937 | NA | NA | NA | NA | NA | NA |
| OTU2943 | NA | NA | NA | + | NA | NA |
| OTU7321 | NA | NA | NA | + | NA | NA |
| OTU3071 | NA | NA | NA | + | NA | NA |
| OTU1388 | NA | NA | + | NA | NA | NA |
| OTU5563 | NA | NA | NA | NA | + | NA |
| OTU5638 | + | NA | NA | NA | NA | NA |
| OTU5637 | NA | NA | NA | NA | NA | NA |
| OTU5632 | + | NA | NA | NA | NA | NA |
| OTU1992 | NA | NA | NA | + | NA | NA |
| OTU6069 | NA | NA | + | NA | NA | NA |
| OTU5055 | NA | NA | + | NA | NA | NA |
| OTU4942 | NA | NA | NA | NA | + | NA |
| OTU6335 | NA | NA | NA | NA | NA | + |
| OTU515 | NA | NA | + | NA | NA | NA |
| OTU2296 | + | NA | NA | NA | NA | NA |
| OTU5711 | NA | + | NA | NA | NA | NA |
| OTU7076 | NA | NA | NA | NA | NA | NA |
| OTU8267 | NA | NA | NA | + | NA | NA |
| OTU7822 | NA | + | NA | NA | NA | NA |
| OTU900 | NA | NA | + | NA | NA | NA |
| OTU689 | NA | NA | + | NA | NA | NA |
| OTU684 | NA | NA | + | NA | NA | NA |
| OTU686 | NA | NA | + | NA | NA | NA |
| OTU7947 | NA | NA | + | NA | NA | NA |
| OTU4018 | NA | NA | NA | NA | + | NA |
| OTU6931 | NA | NA | NA | + | NA | NA |
| OTU2708 | NA | NA | NA | NA | + | NA |
| OTU8215 | + | NA | NA | NA | NA | NA |
| OTU2920 | NA | NA | NA | + | NA | NA |
| OTU6047 | NA | NA | + | NA | NA | NA |
| OTU7126 | NA | NA | + | NA | NA | NA |
| OTU3097 | NA | NA | NA | + | NA | NA |
| OTU536 | NA | NA | + | NA | NA | NA |
| OTU535 | + | NA | NA | NA | NA | NA |
| OTU532 | NA | NA | + | NA | NA | NA |
| OTU530 | NA | NA | + | NA | NA | NA |
| OTU3143 | NA | NA | NA | NA | NA | NA |
| OTU3276 | NA | NA | NA | NA | NA | + |
| OTU1468 | NA | + | + | NA | NA | NA |
| OTU7023 | NA | NA | + | NA | NA | NA |
| OTU2484 | NA | NA | NA | NA | NA | NA |
| OTU4885 | + | NA | NA | NA | NA | NA |
| OTU5658 | + | NA | NA | NA | NA | NA |
| OTU6056 | NA | + | NA | NA | NA | NA |
| OTU5592 | NA | NA | + | NA | NA | NA |
| OTU5856 | NA | NA | NA | NA | NA | + |
| OTU6510 | NA | NA | NA | + | NA | NA |
| OTU4285 | NA | NA | NA | + | NA | NA |
| OTU2081 | NA | NA | NA | + | NA | NA |
| OTU1780 | NA | NA | + | NA | NA | NA |
| OTU4150 | NA | + | NA | NA | NA | NA |
| OTU3725 | + | NA | NA | NA | NA | NA |
| OTU1980 | NA | + | NA | NA | NA | NA |
| OTU3569 | NA | NA | + | NA | NA | NA |
| OTU108 | NA | NA | NA | NA | NA | NA |
| OTU4379 | NA | NA | NA | NA | + | NA |
| OTU5741 | + | NA | NA | NA | NA | NA |
| OTU4140 | NA | NA | NA | NA | NA | NA |
| OTU7019 | NA | NA | + | NA | NA | NA |
| OTU6551 | NA | NA | NA | NA | NA | + |
| OTU136 | NA | NA | + | NA | NA | NA |
| OTU4732 | NA | + | NA | NA | NA | NA |
| OTU6992 | NA | NA | NA | + | NA | NA |
| OTU1359 | NA | NA | NA | + | NA | NA |
| OTU7884 | NA | NA | + | NA | NA | NA |
| OTU501 | NA | NA | + | NA | NA | NA |
| OTU6920 | NA | NA | + | NA | NA | NA |
| OTU4034 | NA | + | NA | NA | NA | NA |
| OTU3434 | NA | NA | NA | + | NA | NA |
| OTU5022 | + | NA | NA | NA | NA | NA |
| OTU3345 | NA | NA | NA | + | NA | NA |
| OTU6436 | NA | NA | NA | NA | NA | NA |
| OTU7081 | NA | NA | + | NA | NA | NA |
| OTU3129 | NA | + | NA | NA | NA | NA |
| OTU2969 | NA | NA | + | NA | NA | NA |
| OTU5862 | NA | NA | NA | + | NA | NA |
| OTU1054 | NA | NA | NA | + | NA | NA |
| OTU1053 | NA | + | NA | + | NA | NA |
| OTU6775 | + | NA | NA | NA | NA | NA |
| OTU4487 | + | NA | NA | NA | NA | NA |
| OTU455 | + | NA | NA | NA | NA | NA |
| OTU451 | NA | NA | NA | NA | NA | NA |
| OTU5625 | NA | NA | NA | NA | NA | + |
| OTU2038 | NA | NA | NA | NA | NA | NA |
| OTU4261 | NA | NA | NA | + | NA | NA |
| OTU4263 | NA | NA | NA | NA | NA | NA |
| OTU6172 | + | NA | NA | NA | NA | NA |
| OTU5148 | NA | NA | NA | NA | + | NA |
| OTU3466 | NA | NA | NA | + | NA | NA |
| OTU3461 | NA | NA | NA | NA | NA | NA |
| OTU2559 | NA | NA | NA | NA | NA | NA |
| OTU4621 | NA | + | NA | NA | NA | NA |
| OTU590 | NA | NA | + | NA | NA | NA |
| OTU637 | NA | NA | + | NA | NA | NA |
| OTU3173 | NA | NA | NA | NA | NA | + |
| OTU7689 | NA | NA | NA | NA | NA | + |
| OTU8020 | NA | + | NA | NA | NA | NA |
| OTU3904 | NA | NA | NA | NA | + | NA |
| OTU3905 | NA | NA | + | NA | NA | NA |
| OTU3902 | NA | NA | NA | NA | NA | NA |
| OTU4949 | + | NA | NA | NA | NA | NA |
| OTU7287 | NA | NA | NA | + | NA | NA |
| OTU4152 | NA | NA | NA | + | NA | NA |
| OTU7284 | + | NA | NA | NA | NA | NA |
| OTU4887 | NA | NA | NA | NA | NA | NA |
| OTU5238 | + | NA | NA | NA | NA | NA |
| OTU3103 | NA | NA | NA | + | NA | NA |
| OTU1869 | NA | NA | NA | NA | NA | NA |
| OTU5471 | + | NA | NA | NA | NA | NA |
| OTU5964 | NA | NA | NA | NA | NA | + |
| OTU1 | NA | NA | + | NA | NA | NA |
| OTU5252 | NA | NA | NA | NA | + | NA |
| OTU3741 | NA | NA | NA | NA | NA | + |
| OTU7526 | NA | NA | + | NA | NA | NA |
| OTU4375 | NA | NA | NA | NA | + | NA |
| OTU3775 | NA | NA | NA | + | NA | NA |
| OTU6259 | NA | NA | NA | NA | NA | + |
| OTU3580 | NA | NA | NA | + | NA | NA |
| OTU967 | NA | NA | + | NA | NA | NA |
| OTU4129 | NA | NA | NA | + | + | NA |
| OTU7085 | NA | NA | + | NA | NA | NA |
| OTU3975 | NA | + | NA | NA | NA | NA |
| OTU4589 | NA | NA | NA | NA | + | NA |
| OTU4504 | + | NA | NA | NA | NA | NA |
| OTU4191 | NA | NA | NA | NA | NA | NA |
| OTU4843 | + | NA | NA | NA | NA | NA |
| OTU4198 | NA | + | NA | NA | NA | NA |
| OTU1317 | NA | NA | NA | NA | NA | + |
| OTU3563 | NA | NA | NA | NA | + | NA |
| OTU2124 | NA | NA | NA | + | NA | NA |
| OTU5896 | NA | NA | NA | NA | NA | + |
| OTU5378 | + | NA | NA | NA | NA | NA |
| OTU2195 | NA | NA | NA | NA | NA | NA |
| OTU4223 | NA | + | NA | NA | NA | NA |
| OTU462 | NA | + | NA | NA | NA | NA |
| OTU5035 | + | NA | NA | NA | + | NA |
| OTU2621 | + | NA | NA | NA | NA | NA |
| OTU6940 | NA | NA | NA | NA | + | NA |
| OTU6267 | NA | NA | NA | + | NA | NA |
| OTU3080 | NA | + | NA | NA | NA | NA |
| OTU3280 | NA | NA | + | NA | NA | NA |
| OTU1522 | NA | NA | + | NA | NA | NA |
| OTU2775 | NA | NA | NA | NA | NA | NA |
| OTU8140 | + | NA | NA | NA | NA | NA |
| OTU438 | + | NA | NA | NA | NA | NA |
| OTU1049 | NA | NA | NA | NA | NA | NA |
| OTU7893 | NA | NA | + | NA | NA | NA |
| OTU7963 | NA | NA | + | NA | NA | + |
| OTU641 | NA | NA | + | NA | NA | NA |
| OTU7968 | NA | NA | + | NA | NA | NA |
| OTU1000 | NA | NA | + | NA | NA | NA |
| OTU3019 | NA | NA | + | NA | NA | NA |
| OTU6422 | NA | NA | + | NA | NA | NA |
| OTU4850 | + | NA | NA | NA | NA | NA |
| OTU5163 | + | NA | NA | NA | NA | NA |
| OTU3348 | NA | NA | NA | + | NA | NA |
| OTU677 | NA | NA | + | NA | NA | NA |
| OTU6427 | NA | NA | NA | NA | NA | NA |
| OTU5734 | NA | + | NA | NA | NA | NA |
| OTU5410 | + | NA | NA | NA | NA | NA |
| OTU6331 | NA | NA | NA | NA | NA | + |
| OTU983 | NA | NA | NA | + | NA | NA |
| OTU4030 | NA | + | NA | NA | NA | NA |
| OTU4033 | NA | + | NA | NA | + | NA |
| OTU8272 | NA | NA | NA | + | NA | NA |
| OTU4233 | NA | + | NA | NA | NA | NA |
| OTU5785 | NA | NA | NA | + | NA | NA |
| OTU7961 | NA | NA | NA | NA | NA | + |
| OTU564 | NA | NA | + | NA | NA | NA |
| OTU3886 | NA | NA | NA | NA | NA | NA |
| OTU4416 | + | NA | NA | NA | NA | NA |
| OTU1989 | NA | NA | NA | NA | NA | NA |
| OTU7070 | NA | NA | + | NA | NA | NA |
| OTU7071 | NA | NA | + | NA | NA | NA |
| OTU7075 | NA | NA | + | NA | NA | NA |
| OTU3474 | NA | NA | NA | + | NA | NA |
| OTU683 | NA | NA | + | NA | NA | NA |
| OTU3354 | NA | NA | NA | + | NA | NA |
| OTU7097 | NA | NA | + | NA | NA | NA |
| OTU6099 | NA | NA | NA | + | NA | NA |
| OTU2448 | NA | NA | NA | + | NA | NA |
| OTU5255 | NA | NA | NA | NA | + | NA |
| OTU4058 | NA | NA | NA | + | NA | NA |
| OTU1302 | NA | NA | + | NA | NA | NA |
| OTU4132 | NA | NA | NA | NA | + | NA |
| OTU8141 | NA | NA | NA | NA | + | NA |
| OTU5656 | + | NA | NA | NA | NA | NA |
| OTU4674 | NA | + | + | NA | NA | NA |
| OTU5847 | NA | NA | NA | NA | NA | NA |
| OTU2111 | NA | NA | + | NA | NA | NA |
| OTU4259 | NA | NA | NA | + | NA | NA |
| OTU8139 | NA | NA | NA | + | NA | NA |
| OTU4250 | NA | NA | NA | + | NA | NA |
| OTU4252 | NA | NA | NA | + | NA | NA |
| OTU4299 | NA | + | NA | NA | NA | NA |
| OTU3398 | NA | NA | NA | + | NA | NA |
| OTU2640 | NA | NA | NA | NA | + | NA |
| OTU584 | NA | NA | + | NA | NA | NA |
| OTU5910 | NA | NA | NA | NA | + | NA |
| OTU3312 | NA | NA | NA | + | NA | NA |
| OTU2899 | NA | NA | NA | NA | NA | NA |
| OTU6560 | NA | NA | NA | NA | NA | + |
| OTU5708 | NA | + | NA | NA | NA | NA |
| OTU325 | NA | NA | + | NA | NA | NA |
| OTU4858 | + | NA | NA | NA | NA | NA |
| OTU5696 | NA | NA | NA | NA | + | NA |
| OTU3499 | NA | NA | + | NA | NA | NA |
| OTU177 | NA | NA | NA | NA | NA | NA |
| OTU3061 | NA | NA | NA | + | NA | NA |
| OTU4525 | + | NA | NA | NA | NA | NA |
| OTU2805 | NA | NA | NA | NA | NA | NA |
| OTU8123 | NA | NA | NA | + | NA | NA |
| OTU7553 | NA | NA | NA | NA | NA | + |
| OTU7550 | NA | NA | NA | + | NA | NA |
| OTU3723 | NA | NA | + | NA | NA | NA |
| OTU1695 | NA | NA | NA | NA | NA | NA |
| OTU6900 | NA | NA | + | NA | NA | NA |
| OTU1025 | NA | NA | + | NA | NA | NA |
| OTU2963 | NA | NA | + | NA | NA | NA |
| OTU2964 | NA | NA | + | NA | NA | NA |
| OTU4463 | + | NA | NA | NA | NA | NA |
| OTU5907 | NA | NA | NA | NA | NA | + |
| OTU4968 | NA | NA | NA | + | NA | NA |
| OTU1318 | NA | NA | + | NA | NA | NA |
| OTU7716 | NA | NA | NA | NA | NA | + |
| OTU1760 | NA | NA | + | NA | NA | NA |
| OTU5604 | NA | NA | NA | NA | NA | NA |
| OTU3044 | NA | NA | NA | NA | NA | NA |
| OTU385 | NA | NA | NA | NA | NA | NA |
| OTU7670 | NA | + | NA | NA | NA | NA |
| OTU6137 | NA | NA | + | NA | NA | NA |
| OTU1068 | NA | NA | + | NA | NA | NA |
| OTU7502 | NA | NA | NA | NA | + | NA |
| OTU4551 | NA | NA | NA | NA | + | NA |
| OTU3008 | NA | NA | NA | NA | NA | NA |
| OTU6418 | NA | NA | NA | + | NA | NA |
| OTU6648 | NA | + | NA | NA | NA | NA |
| OTU3807 | NA | + | NA | NA | NA | NA |
| OTU6680 | NA | NA | NA | NA | + | NA |
| OTU7436 | NA | NA | + | NA | NA | NA |
| OTU1900 | NA | NA | NA | NA | NA | NA |
| OTU3922 | + | NA | + | NA | NA | NA |
| OTU3256 | NA | + | NA | NA | NA | NA |
| OTU4413 | + | NA | NA | NA | NA | NA |
| OTU4410 | + | NA | NA | NA | NA | NA |
| OTU5879 | NA | NA | NA | NA | NA | + |
| OTU2251 | NA | NA | NA | + | NA | NA |
| OTU4119 | + | NA | NA | NA | NA | NA |
| OTU5561 | + | NA | NA | NA | NA | NA |
| OTU5415 | NA | NA | NA | NA | + | NA |
| OTU2847 | NA | NA | NA | NA | + | NA |
| OTU4083 | + | NA | NA | NA | NA | NA |
| OTU1967 | NA | NA | NA | + | NA | NA |
| OTU1775 | NA | NA | NA | NA | NA | NA |
| OTU7312 | NA | NA | + | NA | NA | NA |
| OTU1085 | + | NA | NA | NA | + | NA |
| OTU3507 | NA | NA | NA | + | NA | NA |
| OTU3479 | NA | NA | NA | NA | NA | + |
| OTU2678 | + | NA | NA | NA | NA | NA |
| OTU7604 | NA | NA | NA | + | NA | NA |
| OTU3917 | NA | NA | NA | + | NA | NA |
| OTU5505 | NA | NA | NA | NA | + | NA |
| OTU764 | + | NA | + | NA | NA | NA |
| OTU3046 | NA | NA | NA | + | NA | NA |
| OTU744 | NA | NA | NA | NA | NA | NA |
| OTU5756 | NA | NA | NA | + | NA | NA |
| OTU2958 | NA | NA | + | NA | NA | NA |
| OTU406 | NA | NA | + | NA | NA | NA |
| OTU634 | NA | NA | + | NA | NA | NA |
| OTU2556 | NA | NA | NA | NA | + | NA |
| OTU4616 | NA | + | NA | NA | NA | NA |
| OTU7267 | NA | NA | NA | NA | NA | + |
| OTU5226 | NA | NA | NA | NA | + | NA |
| OTU2750 | NA | NA | NA | NA | NA | NA |
| OTU2758 | NA | NA | NA | + | NA | NA |
| OTU5477 | + | NA | NA | NA | NA | NA |
| OTU2730 | NA | NA | NA | NA | + | NA |
| OTU4352 | NA | NA | NA | + | NA | NA |
| OTU5408 | + | NA | NA | NA | NA | NA |
| OTU7943 | NA | NA | + | NA | NA | NA |
| OTU8051 | NA | NA | NA | NA | NA | NA |
| OTU7995 | NA | NA | NA | NA | NA | + |
| OTU7992 | NA | + | NA | NA | NA | NA |
| OTU1524 | NA | NA | NA | + | NA | NA |
| OTU7403 | NA | NA | NA | NA | NA | + |
| OTU5771 | + | NA | NA | NA | NA | NA |
| OTU5432 | NA | NA | NA | NA | + | NA |
| OTU4854 | + | NA | NA | NA | NA | NA |
| OTU5439 | NA | NA | NA | NA | + | NA |
| OTU476 | NA | NA | NA | NA | NA | NA |
| OTU475 | NA | NA | + | NA | NA | NA |
| OTU2029 | NA | NA | + | NA | NA | NA |
| OTU1332 | NA | NA | + | NA | NA | NA |
| OTU3462 | NA | NA | NA | + | NA | NA |
| OTU3004 | NA | NA | + | NA | NA | NA |
| OTU3987 | NA | NA | NA | NA | + | NA |
| OTU3001 | NA | NA | + | NA | NA | NA |
| OTU7050 | NA | NA | + | NA | NA | NA |
| OTU6027 | NA | + | NA | NA | NA | + |
| OTU2892 | NA | + | NA | NA | NA | NA |
| OTU7118 | NA | NA | NA | NA | NA | NA |
| OTU5486 | + | NA | NA | NA | NA | NA |
| OTU4347 | NA | NA | NA | NA | NA | NA |
| OTU3413 | NA | NA | NA | + | NA | NA |
| OTU7040 | NA | NA | + | NA | NA | NA |
| OTU3468 | NA | NA | NA | NA | NA | NA |
| OTU7782 | NA | NA | NA | + | NA | NA |
| OTU1207 | NA | NA | NA | NA | NA | NA |
| OTU3188 | NA | NA | + | NA | NA | NA |
| OTU4958 | NA | NA | NA | NA | + | NA |
| OTU7639 | NA | NA | + | NA | NA | NA |
| OTU19 | NA | NA | + | NA | NA | NA |
| OTU3382 | NA | NA | NA | + | NA | NA |
| OTU13 | NA | NA | NA | NA | + | NA |
| OTU1008 | NA | NA | + | NA | NA | NA |
| OTU5726 | NA | NA | NA | NA | NA | + |
| OTU5082 | NA | NA | NA | NA | NA | NA |
| OTU8005 | NA | NA | NA | NA | NA | + |
| OTU6279 | NA | + | NA | + | NA | NA |
| OTU6273 | NA | NA | NA | NA | NA | + |
| OTU6771 | NA | NA | NA | + | NA | NA |
| OTU7676 | NA | NA | NA | NA | NA | + |
| OTU4178 | NA | + | NA | NA | NA | NA |
| OTU3841 | NA | NA | NA | + | NA | NA |
| OTU4177 | NA | + | NA | NA | NA | NA |
| OTU7564 | NA | NA | NA | NA | NA | + |
| OTU2670 | NA | + | NA | NA | NA | NA |
| OTU7172 | NA | NA | + | NA | NA | NA |
| OTU2864 | NA | NA | NA | + | NA | NA |
| OTU420 | NA | NA | NA | NA | NA | NA |
| OTU3564 | NA | NA | + | NA | NA | NA |
| OTU1111 | NA | NA | + | NA | NA | NA |
| OTU1094 | NA | NA | + | NA | NA | NA |
| OTU7579 | NA | NA | NA | NA | NA | NA |
| OTU7046 | NA | NA | NA | NA | NA | NA |
| OTU7571 | NA | NA | NA | NA | NA | NA |
| OTU2202 | NA | NA | NA | NA | NA | NA |
| OTU5960 | NA | NA | NA | + | NA | NA |
| OTU1399 | NA | NA | + | NA | NA | NA |
| OTU6044 | NA | NA | NA | NA | NA | + |
| OTU5982 | NA | NA | + | NA | NA | NA |
| OTU1426 | + | NA | NA | NA | NA | NA |
| OTU1335 | NA | NA | + | NA | NA | NA |
| OTU5362 | NA | + | NA | NA | NA | NA |
| OTU5868 | NA | NA | NA | NA | NA | + |
| OTU5521 | NA | NA | NA | NA | + | NA |
| OTU4904 | + | NA | NA | NA | NA | NA |
| OTU3075 | NA | NA | NA | + | NA | NA |
| OTU4901 | NA | NA | NA | + | NA | NA |
| OTU3436 | NA | NA | NA | + | NA | NA |
| OTU3091 | NA | NA | NA | + | NA | NA |
| OTU6406 | NA | NA | NA | NA | NA | NA |
| OTU4668 | NA | NA | NA | NA | NA | + |
| OTU7037 | NA | NA | + | NA | NA | NA |
| OTU1398 | NA | + | NA | NA | NA | NA |
| OTU4234 | NA | + | NA | NA | NA | NA |
| OTU4232 | NA | NA | NA | NA | NA | NA |
| OTU4225 | NA | NA | NA | + | NA | NA |
| OTU650 | NA | NA | NA | + | NA | NA |
| OTU653 | NA | NA | + | NA | NA | NA |
| OTU4238 | NA | + | NA | NA | NA | NA |
| OTU3169 | NA | NA | NA | NA | NA | + |
| OTU3249 | NA | NA | NA | + | NA | NA |
| OTU1300 | + | NA | NA | NA | NA | NA |
| OTU7304 | NA | NA | + | NA | NA | NA |
| OTU649 | NA | NA | + | NA | NA | NA |
| OTU648 | NA | NA | + | NA | NA | NA |
| OTU4992 | NA | NA | NA | NA | + | NA |
| OTU5989 | NA | NA | + | NA | NA | NA |
| OTU644 | NA | NA | + | NA | NA | NA |
| OTU5980 | NA | NA | NA | + | NA | NA |
| OTU646 | NA | NA | + | NA | NA | NA |
| OTU7811 | NA | + | NA | NA | NA | NA |
| OTU8250 | NA | NA | + | NA | NA | NA |
| OTU4179 | NA | + | NA | NA | NA | NA |
| OTU6124 | NA | NA | + | NA | NA | NA |
| OTU5539 | + | NA | NA | NA | + | NA |
| OTU1627 | NA | NA | + | NA | NA | NA |
| OTU4912 | + | NA | NA | NA | NA | NA |
| OTU5186 | NA | NA | + | NA | NA | NA |
| OTU7106 | NA | + | NA | NA | NA | NA |
| OTU5529 | NA | NA | NA | + | NA | NA |
| OTU4788 | NA | NA | NA | NA | + | NA |
| OTU2104 | NA | NA | NA | NA | NA | NA |
| OTU7792 | NA | + | NA | NA | NA | + |
| OTU3417 | NA | NA | NA | + | NA | NA |
| OTU7094 | NA | NA | + | NA | NA | NA |
| OTU5780 | NA | NA | NA | NA | NA | NA |
| OTU1981 | NA | NA | + | NA | NA | NA |
| OTU6515 | NA | NA | NA | NA | NA | + |
| OTU6516 | NA | NA | NA | NA | NA | + |
| OTU5544 | NA | NA | NA | + | NA | NA |
| OTU5469 | + | NA | NA | NA | NA | NA |
| OTU3496 | NA | NA | NA | + | NA | NA |
| OTU3497 | NA | NA | NA | NA | NA | NA |
| OTU3494 | NA | NA | NA | + | NA | NA |
| OTU3492 | NA | NA | NA | NA | NA | NA |
| OTU3491 | NA | NA | NA | + | NA | NA |
| OTU5317 | NA | + | NA | NA | NA | NA |
| OTU510 | NA | NA | + | NA | NA | NA |
| OTU6926 | NA | NA | NA | NA | NA | NA |
| OTU2329 | NA | NA | NA | NA | + | NA |
| OTU7132 | NA | NA | + | NA | NA | NA |
| OTU851 | NA | NA | + | NA | NA | NA |
| OTU1433 | NA | NA | + | NA | NA | NA |
| OTU3470 | NA | NA | NA | + | NA | NA |
| OTU5128 | NA | NA | NA | NA | + | NA |
| OTU2808 | NA | NA | NA | + | NA | NA |
| OTU8034 | NA | NA | NA | + | NA | NA |
| OTU5243 | NA | NA | NA | NA | + | NA |
| OTU7101 | NA | NA | + | NA | NA | NA |
| OTU4002 | NA | NA | NA | NA | + | NA |
| OTU2093 | NA | NA | NA | NA | NA | NA |
| OTU2863 | NA | NA | NA | + | NA | NA |
| OTU2890 | NA | NA | NA | + | NA | NA |
| OTU5072 | NA | NA | NA | NA | NA | NA |
| OTU4419 | + | NA | NA | NA | NA | NA |
| OTU842 | NA | + | NA | NA | NA | NA |
| OTU6586 | NA | NA | NA | NA | + | NA |
| OTU4158 | NA | + | NA | NA | NA | NA |
| OTU3899 | NA | NA | NA | NA | NA | NA |
| OTU458 | NA | + | NA | NA | NA | NA |
| OTU5867 | NA | NA | NA | NA | NA | + |
| OTU4738 | NA | NA | NA | NA | + | NA |
| OTU4438 | + | NA | NA | NA | NA | NA |
| OTU4913 | + | NA | NA | NA | NA | NA |
| OTU4916 | NA | NA | + | NA | NA | NA |
| OTU4915 | NA | NA | NA | NA | + | NA |
| OTU3024 | NA | NA | + | NA | NA | NA |
| OTU7937 | NA | NA | + | NA | NA | NA |
| OTU6455 | + | NA | NA | NA | NA | NA |
| OTU4439 | NA | NA | NA | NA | + | NA |
| OTU770 | NA | NA | + | NA | NA | NA |
| OTU1624 | NA | NA | + | NA | NA | NA |
| OTU3414 | NA | NA | NA | + | NA | NA |
| OTU5801 | NA | NA | NA | NA | NA | + |
| OTU2191 | NA | NA | NA | NA | NA | + |
| OTU404 | + | NA | NA | NA | NA | NA |
| OTU4156 | NA | + | NA | NA | NA | NA |
| OTU4155 | NA | + | + | NA | NA | NA |
| OTU148 | NA | NA | + | NA | NA | NA |
| OTU4686 | NA | NA | NA | NA | NA | NA |
| OTU7038 | NA | NA | + | NA | NA | NA |
| OTU4549 | NA | NA | NA | NA | + | NA |
| OTU5141 | NA | NA | NA | NA | + | NA |
| OTU4609 | NA | NA | NA | NA | NA | NA |
| OTU3517 | NA | NA | NA | NA | NA | + |
| OTU2515 | NA | NA | NA | NA | + | NA |
| OTU2517 | + | NA | NA | NA | NA | NA |
| OTU2510 | NA | NA | + | NA | NA | NA |
| OTU2519 | NA | + | + | NA | + | NA |
| OTU2692 | NA | NA | NA | + | NA | NA |
| OTU273 | NA | NA | NA | + | NA | NA |
| OTU4927 | NA | + | NA | + | NA | NA |
| OTU6671 | + | NA | NA | NA | NA | NA |
| OTU2196 | NA | NA | NA | + | NA | NA |
| OTU7897 | NA | NA | NA | + | NA | NA |
| OTU6012 | + | NA | NA | NA | NA | NA |
| OTU7087 | NA | NA | + | NA | NA | NA |
| OTU5722 | NA | NA | NA | NA | + | NA |
| OTU7337 | NA | NA | NA | + | NA | NA |
| OTU5427 | NA | NA | NA | NA | + | NA |
| OTU6673 | NA | NA | + | NA | NA | + |
| OTU6793 | NA | NA | NA | + | NA | NA |
| OTU4977 | NA | NA | NA | NA | + | NA |
| OTU697 | NA | NA | NA | NA | NA | NA |
| OTU5275 | NA | NA | NA | NA | + | NA |
| OTU3546 | NA | NA | NA | + | NA | NA |
| OTU4212 | NA | + | NA | + | NA | NA |
| OTU4216 | NA | NA | NA | NA | NA | NA |
| OTU4322 | NA | + | NA | NA | NA | NA |
| OTU3442 | NA | NA | NA | + | NA | NA |
| OTU663 | NA | NA | + | NA | NA | NA |
| OTU667 | NA | NA | + | NA | NA | NA |
| OTU1693 | NA | NA | NA | + | NA | NA |
| OTU664 | NA | NA | + | NA | NA | NA |
| OTU668 | NA | NA | NA | NA | NA | NA |
| OTU640 | NA | NA | + | NA | NA | NA |
| OTU866 | NA | + | NA | NA | + | NA |
| OTU5615 | NA | + | NA | NA | NA | NA |
| OTU593 | + | NA | + | NA | NA | NA |
| OTU4555 | NA | + | + | NA | + | NA |
| OTU1130 | NA | NA | + | NA | NA | NA |
| OTU3646 | NA | NA | NA | NA | + | NA |
| OTU7316 | NA | NA | NA | + | NA | NA |
| OTU7035 | NA | NA | + | NA | NA | NA |
| OTU7032 | NA | NA | NA | NA | NA | NA |
| OTU6437 | NA | NA | + | NA | NA | NA |
| OTU3069 | NA | NA | NA | + | NA | NA |
| OTU3068 | NA | NA | NA | + | NA | NA |
| OTU2053 | NA | NA | + | NA | NA | NA |
| OTU2590 | NA | + | NA | NA | NA | NA |
| OTU5926 | NA | NA | NA | NA | NA | + |
| OTU2988 | NA | NA | NA | NA | NA | + |
| OTU399 | NA | NA | + | NA | NA | NA |
| OTU7620 | NA | NA | NA | NA | NA | + |
| OTU1618 | NA | NA | NA | + | NA | NA |
| OTU1233 | + | NA | NA | NA | NA | + |
| OTU4217 | NA | + | NA | NA | NA | NA |
| OTU5736 | NA | NA | NA | NA | NA | NA |
| OTU4331 | NA | NA | NA | + | NA | NA |
| OTU37 | NA | NA | NA | + | NA | NA |
| OTU4026 | NA | NA | + | NA | NA | NA |
| OTU128 | NA | NA | NA | NA | + | NA |
| OTU3087 | NA | NA | NA | + | NA | NA |
| OTU4350 | NA | NA | NA | NA | NA | NA |
| OTU2242 | NA | NA | NA | NA | + | NA |
| OTU2300 | + | NA | NA | NA | NA | NA |
| OTU4889 | + | NA | NA | NA | NA | NA |
| OTU2655 | NA | NA | NA | NA | NA | + |
| OTU6493 | + | NA | NA | NA | NA | NA |
| OTU3101 | NA | NA | NA | + | NA | NA |
| OTU7041 | NA | NA | + | NA | NA | NA |
| OTU2787 | NA | NA | NA | NA | NA | NA |
| OTU1730 | NA | NA | NA | + | NA | NA |
| OTU3440 | NA | + | NA | NA | NA | NA |
| OTU2993 | NA | NA | + | NA | NA | NA |
| OTU2992 | NA | NA | + | NA | NA | NA |
| OTU6768 | NA | NA | NA | NA | + | NA |
| OTU4867 | + | NA | NA | NA | NA | NA |
| OTU1854 | NA | NA | NA | NA | NA | NA |
| OTU6066 | NA | NA | NA | + | NA | NA |
| OTU8047 | NA | NA | NA | + | NA | NA |
| OTU5309 | NA | NA | NA | NA | NA | + |
| OTU1952 | NA | NA | NA | + | NA | NA |
| OTU2851 | NA | NA | NA | NA | + | NA |
| OTU6842 | NA | + | NA | NA | NA | NA |
| OTU785 | NA | NA | NA | NA | NA | NA |
| OTU50 | NA | NA | NA | NA | NA | NA |
| OTU4468 | + | NA | NA | NA | NA | NA |
| OTU8246 | NA | NA | + | NA | NA | NA |
| OTU3420 | NA | NA | NA | + | NA | NA |
| OTU7297 | NA | NA | + | NA | NA | NA |
| OTU829 | NA | NA | NA | NA | NA | + |
| OTU1725 | NA | + | NA | NA | NA | NA |
| OTU4143 | NA | + | NA | NA | NA | NA |
| OTU5541 | + | NA | NA | NA | NA | NA |
| OTU6891 | + | NA | NA | NA | NA | NA |
| OTU4735 | + | NA | NA | NA | NA | NA |
| OTU4973 | + | NA | NA | NA | NA | NA |
| OTU558 | NA | NA | + | NA | NA | NA |
| OTU4050 | NA | NA | NA | NA | + | NA |
| OTU7017 | NA | NA | + | NA | NA | NA |
| OTU6844 | NA | NA | NA | NA | NA | + |
| OTU861 | NA | NA | NA | + | NA | NA |
| OTU3212 | NA | NA | NA | + | NA | NA |
| OTU7772 | NA | NA | NA | + | NA | NA |
| OTU7955 | NA | NA | NA | NA | NA | + |
| OTU1790 | NA | NA | + | NA | NA | NA |
| OTU4459 | + | NA | NA | NA | NA | NA |
| OTU4456 | + | NA | NA | NA | NA | NA |
| OTU4457 | + | NA | NA | NA | NA | NA |
| OTU4450 | + | NA | NA | NA | NA | NA |
| OTU7047 | NA | NA | + | NA | NA | NA |
| OTU3774 | NA | + | NA | NA | NA | NA |
| OTU2888 | NA | NA | NA | + | NA | NA |
| OTU5428 | NA | NA | NA | + | NA | NA |
| OTU3045 | NA | NA | NA | + | NA | NA |
| OTU3919 | NA | NA | NA | NA | NA | + |
| OTU4882 | + | NA | NA | NA | NA | NA |
| OTU1306 | NA | NA | NA | NA | NA | NA |
| OTU4426 | NA | NA | NA | NA | + | NA |
| OTU7205 | NA | NA | + | NA | NA | NA |
| OTU4211 | NA | + | NA | NA | NA | NA |
| OTU4506 | NA | NA | NA | + | NA | NA |
| OTU3040 | NA | NA | + | NA | NA | NA |
| OTU2554 | NA | NA | NA | NA | NA | NA |
| OTU6530 | NA | NA | NA | + | NA | NA |
| OTU7801 | NA | NA | + | NA | NA | NA |
| OTU3136 | NA | NA | NA | NA | NA | + |
| OTU3498 | NA | NA | NA | + | NA | NA |
| OTU7164 | NA | NA | + | NA | NA | NA |
| OTU3472 | NA | NA | + | NA | NA | NA |
| OTU2820 | NA | + | NA | NA | + | NA |
| OTU2826 | NA | NA | NA | NA | + | NA |
| OTU4130 | + | NA | NA | NA | NA | NA |
| OTU575 | NA | NA | NA | NA | NA | NA |
| OTU578 | NA | NA | + | NA | NA | NA |
| OTU6115 | NA | NA | NA | NA | NA | + |
| OTU4185 | NA | + | NA | NA | NA | NA |
| OTU5889 | NA | NA | NA | NA | NA | + |
| OTU956 | NA | NA | + | NA | NA | NA |
| OTU4591 | NA | NA | NA | NA | NA | + |
| OTU2359 | NA | NA | + | NA | NA | NA |
| OTU6683 | NA | NA | NA | NA | NA | + |
| OTU2844 | NA | NA | NA | NA | NA | + |
| OTU4182 | NA | + | NA | NA | NA | NA |
| OTU5447 | + | NA | NA | NA | NA | NA |
| OTU1419 | NA | NA | NA | + | NA | NA |
| OTU5090 | NA | NA | NA | NA | NA | NA |
| OTU2353 | NA | NA | NA | + | NA | NA |
| OTU4718 | NA | NA | NA | NA | NA | NA |
| OTU5755 | NA | NA | NA | NA | NA | NA |
| OTU4462 | + | NA | NA | NA | NA | NA |
| OTU3781 | NA | NA | NA | NA | NA | NA |
| OTU4953 | NA | NA | NA | NA | + | NA |
| OTU7480 | NA | NA | NA | NA | NA | + |
| OTU4758 | NA | NA | NA | NA | + | NA |
| OTU4208 | NA | + | NA | NA | NA | NA |
| OTU7117 | NA | NA | + | NA | NA | NA |
| OTU6925 | NA | NA | NA | + | NA | NA |
| OTU7599 | + | NA | NA | NA | NA | NA |
| OTU1765 | NA | + | NA | NA | NA | NA |
| OTU2663 | NA | NA | NA | NA | NA | NA |
| OTU5895 | NA | NA | NA | NA | NA | + |
| OTU7346 | NA | NA | NA | NA | + | NA |
| OTU4399 | + | NA | NA | NA | NA | NA |
| OTU4962 | NA | NA | NA | NA | NA | NA |
| OTU935 | NA | NA | + | NA | NA | NA |
| OTU2429 | NA | NA | NA | + | NA | NA |
| OTU845 | NA | NA | NA | NA | NA | NA |
| OTU704 | NA | NA | NA | NA | NA | NA |
| OTU4798 | NA | + | NA | NA | NA | + |
| OTU3668 | NA | NA | NA | NA | NA | + |
| OTU4218 | NA | + | NA | NA | NA | NA |
| OTU596 | NA | NA | + | NA | NA | NA |
| OTU7018 | NA | NA | + | NA | NA | NA |
| OTU3041 | NA | NA | NA | + | NA | NA |
| OTU3609 | NA | NA | NA | + | NA | NA |
| OTU7014 | NA | NA | + | NA | NA | NA |
| OTU2637 | + | NA | NA | NA | NA | NA |
| OTU3707 | NA | NA | + | NA | NA | NA |
| OTU1801 | + | NA | NA | NA | NA | NA |
| OTU2945 | NA | NA | NA | NA | + | NA |
| OTU7243 | NA | + | NA | NA | NA | NA |
| OTU6491 | NA | NA | + | NA | NA | NA |
| OTU6490 | NA | NA | NA | NA | NA | + |
| OTU4327 | NA | NA | NA | + | NA | NA |
| OTU950 | NA | NA | NA | + | NA | NA |
| OTU6945 | NA | NA | NA | NA | NA | + |
| OTU3484 | NA | NA | NA | + | NA | NA |
| OTU3630 | NA | NA | NA | + | NA | NA |
| OTU2526 | NA | NA | NA | NA | NA | + |
| OTU8095 | + | NA | NA | NA | NA | NA |
| OTU5484 | + | NA | NA | NA | + | NA |
| OTU1006 | NA | NA | NA | + | NA | NA |
| OTU4864 | + | NA | NA | NA | NA | NA |
| OTU7114 | NA | NA | + | NA | NA | NA |
| OTU774 | NA | NA | NA | NA | NA | NA |
| OTU5254 | NA | NA | NA | NA | + | NA |
| OTU5704 | + | NA | NA | NA | NA | NA |
| OTU4742 | NA | NA | NA | NA | + | NA |
| OTU6234 | NA | NA | NA | NA | NA | + |
| OTU5631 | NA | NA | + | NA | NA | NA |
| OTU8166 | NA | NA | NA | NA | NA | NA |
| OTU4210 | NA | + | NA | NA | NA | NA |
| OTU4171 | NA | + | NA | NA | NA | NA |
| OTU5083 | NA | NA | NA | NA | NA | NA |
| OTU5080 | NA | + | NA | NA | NA | NA |
| OTU2316 | NA | NA | NA | NA | NA | NA |
| OTU5146 | NA | NA | NA | NA | + | NA |
| OTU5149 | NA | NA | NA | NA | + | NA |
| OTU8142 | + | NA | NA | NA | NA | NA |
| OTU1104 | NA | NA | NA | NA | NA | NA |
| OTU8206 | NA | NA | NA | NA | NA | + |
| OTU1671 | NA | NA | NA | NA | NA | NA |
| OTU7200 | + | NA | NA | NA | NA | NA |
| OTU3989 | + | NA | NA | NA | NA | NA |
| OTU2422 | NA | NA | NA | + | NA | NA |
| OTU171 | NA | NA | + | NA | NA | NA |
| OTU5851 | NA | NA | NA | NA | NA | + |
| OTU5877 | NA | NA | NA | NA | NA | + |
| OTU5322 | NA | NA | NA | + | NA | NA |
| OTU5320 | NA | + | NA | NA | NA | NA |
| OTU8039 | + | NA | NA | NA | NA | NA |
| OTU4273 | NA | NA | NA | + | NA | NA |
| OTU5094 | NA | NA | + | NA | NA | NA |
| OTU5091 | NA | NA | NA | + | NA | NA |
| OTU4636 | NA | NA | NA | NA | NA | NA |
| OTU4634 | NA | + | NA | NA | NA | NA |
| OTU7560 | NA | NA | NA | NA | NA | + |
| OTU3185 | NA | NA | + | NA | NA | NA |
| OTU3252 | NA | NA | NA | + | NA | NA |
| OTU51 | NA | + | NA | NA | NA | NA |
| OTU3353 | NA | NA | NA | NA | NA | + |
| OTU4894 | + | NA | NA | NA | NA | NA |
| OTU805 | NA | NA | + | NA | NA | NA |
| OTU3404 | NA | NA | + | NA | NA | NA |
| OTU1050 | NA | NA | + | NA | NA | NA |
| OTU1309 | NA | NA | NA | NA | NA | NA |
| OTU604 | NA | NA | + | NA | NA | NA |
| OTU8045 | NA | NA | NA | + | NA | NA |
| OTU3012 | NA | NA | + | NA | NA | NA |
| OTU4093 | NA | NA | NA | NA | + | NA |
| OTU4070 | + | NA | NA | NA | NA | NA |
| OTU5460 | + | NA | NA | NA | NA | NA |
| OTU7607 | NA | NA | + | NA | NA | NA |
| OTU6118 | NA | NA | + | NA | NA | NA |
| OTU4892 | + | NA | NA | NA | NA | NA |
| OTU4914 | + | NA | NA | NA | NA | NA |
| OTU720 | NA | + | NA | NA | NA | NA |
| OTU6555 | NA | NA | + | NA | NA | + |
| OTU7211 | NA | NA | + | NA | NA | NA |
| OTU6000 | NA | + | NA | NA | NA | NA |
| OTU601 | NA | NA | + | NA | NA | NA |
| OTU6559 | NA | NA | NA | NA | NA | + |
| OTU1915 | NA | NA | NA | NA | NA | NA |
| OTU5560 | NA | NA | NA | NA | NA | NA |
| OTU83 | NA | NA | + | NA | NA | NA |
| OTU2579 | + | NA | NA | NA | NA | NA |
| OTU3187 | NA | NA | + | NA | NA | NA |
| OTU43 | NA | NA | NA | NA | NA | + |
| OTU1777 | NA | NA | NA | + | NA | NA |
| OTU3432 | NA | NA | NA | NA | NA | NA |
| OTU2823 | NA | NA | NA | + | NA | + |
| OTU4008 | NA | NA | NA | NA | NA | + |
| OTU3921 | NA | NA | NA | + | NA | NA |
| OTU6102 | NA | NA | NA | NA | NA | NA |
| OTU5629 | NA | + | NA | NA | NA | NA |
| OTU382 | NA | NA | NA | + | NA | NA |
| OTU383 | NA | NA | NA | NA | NA | NA |
| OTU4782 | NA | NA | NA | NA | + | NA |
| OTU6788 | NA | + | NA | NA | NA | NA |
| OTU4519 | + | NA | NA | NA | NA | NA |
| OTU6353 | NA | NA | NA | NA | NA | + |
| OTU1588 | NA | + | NA | NA | NA | + |
| OTU5154 | + | NA | NA | NA | NA | NA |
| OTU1583 | NA | NA | + | NA | NA | NA |
| OTU3451 | NA | NA | NA | + | NA | NA |
| OTU5409 | + | NA | NA | NA | NA | NA |
| OTU555 | NA | NA | + | NA | NA | NA |
| OTU4409 | + | NA | NA | NA | NA | NA |
| OTU293 | + | + | NA | NA | NA | NA |
| OTU3704 | + | NA | NA | NA | + | NA |
| OTU7725 | NA | NA | NA | NA | NA | NA |
| OTU7063 | NA | NA | + | NA | NA | NA |
| OTU4348 | NA | NA | NA | NA | NA | NA |
| OTU7578 | NA | + | NA | NA | NA | NA |
| OTU4819 | NA | NA | NA | NA | + | NA |
| OTU569 | NA | NA | + | NA | NA | NA |
| OTU5622 | + | NA | NA | NA | NA | NA |
| OTU1666 | NA | NA | + | NA | NA | NA |
| OTU336 | NA | NA | NA | NA | NA | NA |
| OTU3452 | NA | NA | NA | + | NA | NA |
| OTU568 | NA | NA | + | NA | NA | NA |
| OTU27 | NA | NA | + | NA | NA | NA |
| OTU504 | NA | NA | NA | NA | + | NA |
| OTU5799 | NA | NA | NA | NA | NA | + |
| OTU3291 | NA | NA | NA | + | NA | NA |
| OTU5712 | + | NA | NA | NA | NA | NA |
| OTU508 | NA | NA | + | NA | NA | NA |
| OTU4950 | NA | NA | NA | NA | NA | + |
| OTU665 | NA | NA | + | NA | NA | NA |
| OTU4791 | NA | NA | NA | NA | + | NA |
| OTU4623 | NA | NA | NA | NA | NA | NA |
| OTU7755 | NA | NA | + | NA | NA | NA |
| OTU3985 | NA | NA | NA | + | NA | NA |
| OTU5634 | NA | NA | NA | + | NA | NA |
| OTU4841 | + | NA | NA | NA | NA | NA |
| OTU3370 | NA | NA | NA | + | NA | NA |
| OTU7206 | + | NA | NA | NA | NA | NA |
| OTU7151 | NA | NA | + | NA | NA | NA |
| OTU2434 | NA | + | NA | NA | NA | NA |
| OTU118 | NA | NA | NA | + | NA | NA |
| OTU2954 | NA | NA | NA | + | NA | NA |
| OTU1385 | NA | NA | + | NA | NA | NA |
| OTU7996 | NA | NA | NA | NA | NA | NA |
| OTU3000 | NA | NA | + | NA | NA | NA |
| OTU4789 | NA | NA | NA | NA | + | NA |
| OTU1360 | NA | NA | + | NA | NA | NA |
| OTU4786 | + | NA | NA | NA | NA | NA |
| OTU2352 | + | NA | NA | NA | NA | NA |
| OTU4112 | NA | NA | + | NA | NA | NA |
| OTU229 | NA | NA | NA | NA | + | NA |
| OTU6076 | NA | NA | NA | NA | NA | NA |
| OTU4833 | + | NA | NA | NA | NA | NA |
| OTU189 | NA | NA | + | NA | NA | NA |
| OTU7858 | NA | NA | NA | NA | NA | + |
| OTU7147 | NA | NA | + | NA | NA | NA |
| OTU3122 | NA | NA | + | NA | NA | + |
| OTU2461 | NA | NA | + | NA | NA | NA |
| OTU6972 | NA | NA | NA | NA | NA | + |
| OTU960 | NA | NA | NA | NA | + | NA |
| OTU2210 | NA | NA | + | NA | NA | NA |
| OTU597 | NA | NA | + | NA | NA | NA |
| OTU72 | NA | NA | NA | NA | NA | NA |
| OTU1471 | NA | NA | + | NA | NA | NA |
| OTU4090 | NA | NA | NA | NA | + | NA |
| OTU5461 | + | NA | NA | NA | NA | NA |
| OTU5465 | + | NA | NA | NA | NA | NA |
| OTU7975 | NA | NA | NA | NA | NA | NA |
| OTU6856 | NA | NA | NA | NA | NA | + |
| OTU6614 | NA | NA | NA | NA | NA | + |
| OTU73 | NA | NA | NA | NA | NA | NA |
| OTU76 | NA | NA | NA | + | NA | NA |
| OTU4881 | + | NA | NA | NA | NA | NA |
| OTU5713 | NA | NA | NA | NA | + | NA |
| OTU3814 | NA | NA | NA | NA | NA | NA |
| OTU4542 | NA | NA | NA | NA | + | NA |
| OTU4226 | NA | NA | NA | NA | NA | NA |
| OTU628 | NA | NA | + | NA | NA | NA |
| OTU624 | NA | NA | + | NA | NA | NA |
| OTU4797 | + | NA | NA | NA | + | NA |
| OTU3933 | NA | + | NA | NA | NA | NA |
| OTU2327 | NA | NA | NA | NA | NA | NA |
| OTU3365 | NA | NA | NA | + | NA | NA |
| OTU7771 | NA | NA | + | NA | NA | NA |
| OTU8041 | NA | NA | NA | NA | NA | NA |
| OTU5188 | NA | NA | NA | NA | NA | NA |
| OTU581 | NA | NA | + | NA | NA | NA |
| OTU4546 | NA | NA | NA | NA | + | NA |
| OTU4576 | NA | NA | NA | NA | NA | NA |
| OTU7568 | NA | NA | + | NA | NA | NA |
| OTU5216 | NA | NA | NA | + | NA | NA |
| OTU6960 | NA | NA | NA | NA | NA | NA |
| OTU971 | NA | NA | + | NA | NA | NA |
| OTU4821 | NA | NA | NA | NA | + | NA |
| OTU7216 | NA | NA | NA | + | NA | NA |
| OTU5401 | NA | NA | NA | NA | + | NA |
| OTU190 | NA | NA | NA | + | NA | NA |
| OTU68 | NA | NA | + | NA | NA | NA |
| OTU401 | NA | NA | + | NA | NA | NA |
| OTU3099 | NA | NA | NA | + | NA | NA |
| OTU4725 | NA | NA | NA | + | NA | NA |
| OTU4064 | NA | + | + | NA | NA | NA |
| OTU206 | NA | NA | + | NA | NA | NA |
| OTU1417 | NA | NA | NA | NA | NA | NA |
| OTU7079 | NA | NA | NA | NA | NA | NA |
| OTU3192 | NA | NA | NA | + | NA | NA |
| OTU8156 | NA | NA | NA | NA | NA | NA |
| OTU5451 | NA | NA | NA | NA | + | NA |
| OTU5641 | NA | NA | NA | NA | + | NA |
| OTU369 | NA | NA | NA | + | NA | NA |
| OTU2383 | NA | + | NA | NA | NA | NA |
| OTU366 | NA | NA | + | NA | NA | NA |
| OTU4870 | + | NA | NA | NA | NA | NA |
| OTU6239 | NA | NA | NA | NA | NA | NA |
| OTU3371 | NA | NA | NA | + | NA | NA |
| OTU6778 | NA | NA | NA | + | NA | NA |
| OTU3055 | NA | NA | NA | + | NA | NA |
| OTU1975 | NA | NA | + | NA | NA | NA |
| OTU6023 | NA | NA | + | NA | NA | NA |
| OTU1460 | NA | NA | NA | NA | NA | NA |
| OTU2314 | NA | NA | NA | + | NA | NA |
| OTU1808 | NA | NA | + | NA | NA | NA |
| OTU2405 | + | NA | NA | NA | NA | NA |
| OTU5698 | NA | NA | NA | NA | + | NA |
| OTU5872 | NA | NA | NA | NA | NA | + |
| OTU5876 | NA | NA | NA | NA | NA | + |
| OTU1396 | NA | NA | + | NA | NA | NA |
| OTU7222 | NA | NA | NA | NA | NA | + |
| OTU4614 | + | NA | NA | NA | NA | NA |
| OTU4612 | NA | + | NA | NA | NA | NA |
| OTU1066 | NA | NA | NA | NA | NA | NA |
| OTU4237 | NA | + | NA | NA | NA | NA |
| OTU3213 | NA | NA | NA | + | NA | NA |
| OTU3435 | NA | NA | + | NA | NA | NA |
| OTU3431 | + | NA | NA | NA | NA | NA |
| OTU840 | NA | NA | + | NA | NA | NA |
| OTU2133 | NA | + | NA | NA | NA | NA |
| OTU5435 | NA | NA | NA | NA | + | NA |
| OTU186 | NA | NA | NA | NA | NA | NA |
| OTU3763 | NA | NA | NA | NA | + | NA |
| OTU3033 | NA | NA | + | NA | NA | NA |
| OTU534 | NA | NA | + | NA | NA | NA |
| OTU4930 | NA | + | NA | NA | NA | NA |
| OTU4142 | NA | NA | NA | NA | NA | NA |
| OTU7422 | NA | NA | NA | NA | + | NA |
| OTU4494 | + | NA | NA | NA | NA | NA |
| OTU6084 | + | NA | NA | NA | NA | NA |
| OTU1369 | NA | NA | + | NA | NA | NA |
| OTU4492 | + | NA | NA | NA | NA | NA |
| OTU7224 | NA | NA | NA | + | NA | NA |
| OTU7220 | NA | NA | NA | NA | NA | NA |
| OTU4240 | NA | + | NA | NA | NA | NA |
| OTU2361 | NA | NA | NA | + | NA | NA |
| OTU4243 | NA | NA | NA | NA | NA | NA |
| OTU1208 | NA | NA | NA | NA | NA | + |
| OTU4087 | NA | NA | NA | NA | NA | NA |
| OTU563 | NA | NA | + | NA | NA | NA |
| OTU2614 | NA | NA | + | NA | NA | NA |
| OTU3284 | NA | + | NA | NA | NA | NA |
| OTU4393 | NA | NA | + | NA | NA | NA |
| OTU7682 | NA | NA | NA | NA | + | NA |
| OTU1493 | NA | NA | NA | NA | NA | NA |
| OTU6094 | NA | NA | NA | NA | NA | NA |
| OTU4853 | + | NA | NA | NA | NA | NA |
| OTU5719 | NA | NA | NA | NA | NA | NA |
| OTU6575 | NA | NA | NA | NA | NA | + |
| OTU8257 | NA | NA | + | NA | NA | NA |
| OTU5310 | NA | NA | + | NA | NA | NA |
| OTU3357 | NA | NA | NA | + | NA | NA |
| OTU3697 | NA | NA | NA | NA | + | NA |
| OTU2676 | NA | NA | + | NA | NA | NA |
| OTU1547 | NA | NA | + | NA | NA | NA |
| OTU1813 | NA | NA | + | NA | NA | NA |
| OTU2629 | + | NA | NA | NA | NA | NA |
| OTU6615 | NA | NA | NA | + | NA | NA |
| OTU4025 | NA | + | NA | NA | NA | NA |
| OTU7910 | NA | NA | NA | NA | NA | + |
| OTU4883 | + | NA | NA | NA | NA | NA |
| OTU5482 | + | NA | NA | NA | NA | NA |
| OTU5487 | + | NA | NA | NA | + | NA |
| OTU4558 | NA | NA | NA | NA | + | NA |
| OTU7059 | NA | NA | + | NA | NA | NA |
| OTU7668 | NA | NA | NA | NA | NA | + |
| OTU3978 | NA | + | NA | NA | NA | NA |
| OTU4432 | + | NA | NA | NA | NA | NA |
| OTU4420 | + | NA | NA | NA | NA | NA |
| OTU5717 | NA | NA | NA | NA | + | NA |
| OTU4877 | NA | NA | NA | NA | NA | NA |
| OTU3140 | + | NA | NA | NA | NA | NA |
| OTU1372 | NA | NA | NA | NA | NA | NA |
| OTU4251 | NA | NA | NA | + | NA | NA |
| OTU4845 | + | NA | NA | NA | NA | NA |
| OTU5260 | NA | + | NA | NA | NA | NA |
| OTU4084 | NA | NA | + | NA | NA | NA |
| OTU6967 | NA | NA | NA | NA | NA | + |
| OTU195 | NA | NA | + | NA | NA | NA |
| OTU732 | NA | + | NA | NA | NA | NA |
| OTU3485 | NA | NA | NA | + | NA | NA |
| OTU1904 | NA | NA | NA | NA | NA | NA |
| OTU4619 | NA | + | NA | NA | NA | NA |
| OTU365 | NA | NA | NA | + | NA | NA |
| OTU6903 | NA | NA | NA | NA | NA | + |
| OTU992 | NA | + | NA | NA | NA | NA |
| OTU995 | NA | + | NA | NA | NA | NA |
| OTU3938 | NA | NA | NA | NA | + | NA |
| OTU1848 | NA | NA | + | NA | NA | NA |
| OTU5660 | + | NA | NA | NA | NA | NA |
| OTU8102 | NA | NA | NA | NA | + | NA |
| OTU6011 | NA | NA | NA | NA | NA | NA |
| OTU5182 | + | NA | NA | NA | NA | NA |
| OTU2843 | NA | NA | NA | NA | + | NA |
| OTU4330 | NA | NA | NA | + | NA | NA |
| OTU1815 | NA | NA | + | NA | NA | NA |
| OTU6907 | NA | + | NA | NA | NA | NA |
| OTU1345 | NA | NA | NA | NA | NA | NA |
| OTU116 | + | NA | NA | + | NA | NA |
| OTU1440 | NA | NA | + | NA | NA | NA |
| OTU608 | NA | NA | + | NA | NA | NA |
| OTU6268 | NA | NA | + | NA | NA | NA |
| OTU3640 | NA | NA | + | NA | NA | NA |
| OTU4328 | NA | NA | NA | + | NA | NA |
| OTU1628 | NA | NA | + | NA | NA | NA |
| OTU489 | NA | + | NA | NA | NA | NA |
| OTU1828 | NA | + | NA | NA | NA | NA |
| OTU1620 | NA | NA | NA | NA | NA | NA |
| OTU4118 | NA | NA | NA | NA | + | NA |
| OTU2927 | NA | NA | + | NA | NA | NA |
| OTU3056 | + | NA | NA | NA | NA | NA |
| OTU5328 | NA | + | NA | NA | NA | NA |
| OTU4971 | NA | NA | NA | NA | NA | NA |
| OTU5819 | NA | NA | NA | NA | NA | NA |
| OTU1069 | NA | NA | NA | NA | + | NA |
| OTU4249 | NA | NA | NA | + | NA | NA |
| OTU5813 | NA | NA | NA | NA | NA | + |
| OTU411 | NA | NA | NA | + | NA | NA |
| OTU6488 | NA | + | NA | NA | NA | NA |
| OTU4679 | NA | + | NA | NA | NA | NA |
| OTU6483 | NA | NA | NA | NA | + | NA |
| OTU3415 | NA | NA | NA | + | NA | NA |
| OTU3207 | NA | + | NA | NA | NA | NA |
| OTU4437 | NA | NA | NA | NA | + | NA |
| OTU7176 | NA | NA | + | NA | NA | NA |
| OTU7541 | NA | + | NA | NA | NA | NA |
| OTU6639 | NA | NA | NA | NA | NA | NA |
| OTU5773 | + | NA | NA | NA | NA | NA |
| OTU5389 | NA | NA | + | NA | NA | NA |
| OTU7462 | NA | NA | NA | NA | NA | + |
| OTU6978 | NA | NA | NA | NA | NA | NA |
| OTU3377 | NA | NA | + | NA | NA | NA |
| OTU3378 | NA | NA | NA | + | NA | NA |
| OTU7171 | NA | NA | NA | NA | NA | NA |
| OTU3624 | NA | NA | NA | NA | + | NA |
| OTU4244 | NA | NA | NA | NA | NA | NA |
| OTU3165 | NA | NA | + | NA | NA | NA |
| OTU7179 | NA | NA | + | NA | NA | NA |
| OTU4351 | NA | NA | NA | + | NA | NA |
| OTU6017 | NA | NA | + | NA | NA | NA |
| OTU4793 | NA | NA | NA | NA | + | NA |
| OTU4997 | + | NA | NA | NA | NA | NA |
| OTU2754 | + | NA | NA | NA | NA | NA |
| OTU5697 | + | NA | NA | NA | NA | NA |
| OTU2008 | NA | NA | NA | NA | NA | + |
| OTU6997 | NA | NA | NA | NA | NA | + |
| OTU5499 | + | NA | NA | NA | NA | NA |
| OTU1734 | + | + | NA | NA | NA | NA |
| OTU201 | NA | NA | NA | NA | NA | NA |
| OTU203 | + | NA | + | NA | NA | NA |
| OTU2980 | NA | NA | + | NA | NA | NA |
| OTU2982 | NA | NA | + | NA | NA | NA |
| OTU2930 | NA | NA | + | NA | NA | NA |
| OTU1126 | NA | NA | NA | NA | NA | NA |
| OTU3180 | NA | NA | + | NA | NA | NA |
| OTU3911 | NA | NA | NA | NA | NA | NA |
| OTU7600 | NA | NA | NA | NA | NA | NA |
| OTU4490 | NA | NA | NA | NA | NA | NA |
| OTU3127 | NA | NA | NA | + | NA | NA |
| OTU344 | NA | NA | NA | + | NA | NA |
| OTU1846 | NA | + | NA | NA | NA | NA |
| OTU347 | NA | NA | NA | + | NA | NA |
| OTU6257 | NA | NA | NA | NA | NA | + |
| OTU1402 | NA | NA | + | NA | NA | NA |
| OTU3352 | NA | NA | NA | + | NA | NA |
| OTU5340 | NA | NA | NA | NA | NA | NA |
| OTU4153 | NA | + | NA | NA | NA | NA |
| OTU2084 | NA | NA | NA | NA | + | NA |
| OTU5426 | NA | NA | NA | NA | + | NA |
| OTU8137 | NA | NA | + | NA | NA | NA |
| OTU4444 | + | NA | NA | NA | NA | + |
| OTU1901 | NA | NA | + | NA | NA | NA |
| OTU4441 | NA | NA | NA | NA | NA | NA |
| OTU6445 | NA | NA | + | NA | NA | NA |
| OTU4245 | NA | NA | NA | + | NA | NA |
| OTU7538 | NA | NA | NA | NA | + | NA |
| OTU7863 | + | NA | NA | NA | NA | NA |
| OTU2870 | NA | NA | NA | + | NA | NA |
| OTU5624 | + | NA | NA | NA | NA | NA |
| OTU4966 | NA | NA | NA | NA | + | NA |
| OTU5365 | NA | NA | NA | NA | + | NA |
| OTU205 | NA | NA | + | NA | NA | NA |
| OTU869 | NA | NA | + | NA | NA | NA |
| OTU3418 | NA | NA | NA | + | NA | NA |
| OTU3416 | NA | NA | NA | NA | NA | NA |
| OTU3141 | NA | NA | NA | NA | NA | NA |
| OTU3412 | NA | NA | NA | + | NA | NA |
| OTU3410 | NA | NA | + | NA | NA | NA |
| OTU3421 | NA | NA | NA | + | NA | NA |
| OTU6794 | + | NA | NA | NA | NA | NA |
| OTU3094 | NA | NA | NA | NA | NA | NA |
| OTU7247 | NA | NA | NA | NA | NA | NA |
| OTU7713 | NA | NA | NA | NA | NA | NA |
| OTU5703 | + | NA | NA | NA | NA | NA |
| OTU5905 | NA | NA | NA | NA | NA | NA |
| OTU1549 | NA | NA | NA | NA | NA | NA |
| OTU8278 | NA | NA | + | NA | NA | NA |
| OTU961 | NA | NA | + | NA | NA | NA |
| OTU3026 | NA | NA | + | NA | NA | NA |
| OTU7131 | NA | NA | + | NA | NA | NA |
| OTU3604 | NA | NA | NA | + | NA | NA |
| OTU7139 | NA | NA | + | NA | NA | NA |
| OTU4121 | NA | + | NA | NA | NA | NA |
| OTU938 | NA | + | + | NA | NA | NA |
| OTU5398 | + | NA | NA | NA | NA | NA |
| OTU7192 | NA | NA | NA | + | NA | NA |
| OTU8230 | NA | NA | NA | NA | NA | NA |
| OTU2821 | NA | NA | NA | + | NA | NA |
| OTU3310 | NA | NA | NA | NA | NA | + |
| OTU5207 | NA | NA | NA | NA | + | NA |
| OTU2878 | NA | NA | NA | + | NA | NA |
| OTU3955 | NA | NA | + | NA | NA | NA |
| OTU5203 | NA | NA | NA | NA | + | NA |
| OTU523 | NA | NA | NA | NA | NA | NA |
| OTU4154 | NA | NA | + | NA | NA | NA |
| OTU7285 | NA | NA | NA | NA | NA | + |
| OTU2815 | NA | NA | + | NA | NA | NA |
| OTU4550 | NA | NA | NA | NA | + | NA |
| OTU3372 | NA | NA | NA | + | NA | NA |
| OTU6679 | NA | NA | NA | NA | NA | + |
| OTU941 | NA | NA | + | NA | NA | NA |
| OTU5462 | NA | NA | + | NA | NA | NA |
| OTU4626 | NA | + | NA | NA | NA | NA |
| OTU2740 | NA | NA | NA | NA | NA | NA |
| OTU2898 | + | NA | NA | NA | NA | NA |
| OTU5714 | NA | NA | NA | + | NA | NA |
| OTU2566 | NA | + | NA | NA | NA | NA |
| OTU6155 | NA | NA | + | NA | NA | NA |
| OTU4175 | NA | + | + | NA | NA | NA |
| OTU4204 | NA | + | NA | + | NA | NA |
| OTU2577 | NA | + | NA | NA | NA | NA |
| OTU6653 | + | NA | NA | NA | NA | NA |
| OTU457 | NA | NA | NA | NA | NA | NA |
| OTU4879 | NA | NA | NA | NA | + | NA |
| OTU4878 | NA | NA | + | NA | NA | NA |
| OTU4874 | NA | NA | NA | NA | NA | NA |
| OTU4384 | NA | NA | NA | NA | NA | NA |
| OTU4873 | + | NA | NA | NA | NA | NA |
| OTU3092 | NA | NA | NA | + | NA | NA |
| OTU3614 | NA | NA | + | NA | NA | NA |
| OTU360 | + | NA | NA | NA | NA | NA |
| OTU4630 | NA | NA | NA | NA | NA | NA |
| OTU5568 | NA | + | NA | NA | NA | NA |
| OTU3374 | NA | NA | + | NA | NA | NA |
| OTU3358 | NA | NA | NA | + | NA | NA |
| OTU7384 | NA | NA | NA | + | NA | NA |
| OTU6937 | NA | NA | NA | NA | NA | + |
| OTU2686 | NA | NA | NA | + | NA | NA |
| OTU398 | NA | NA | NA | NA | NA | NA |
| OTU4517 | NA | NA | NA | NA | + | NA |
| OTU4640 | + | NA | NA | NA | + | NA |
| OTU4512 | + | NA | NA | NA | NA | NA |
| OTU5385 | NA | NA | NA | NA | NA | NA |
| OTU2718 | NA | NA | NA | NA | NA | NA |
| OTU4124 | + | NA | NA | NA | NA | NA |
| OTU8179 | NA | + | NA | NA | NA | NA |
| OTU2017 | NA | + | NA | NA | NA | NA |
| OTU5894 | + | NA | NA | NA | NA | NA |
| OTU3391 | NA | NA | NA | NA | + | NA |
| OTU693 | NA | NA | NA | NA | NA | NA |
| OTU6709 | NA | NA | NA | NA | + | NA |
| OTU6745 | + | NA | NA | NA | NA | NA |
| OTU5277 | NA | NA | NA | NA | + | NA |
| OTU3447 | + | NA | NA | NA | NA | NA |
| OTU5849 | NA | NA | NA | NA | NA | + |
| OTU615 | NA | NA | + | NA | NA | NA |
| OTU4665 | NA | NA | + | NA | NA | NA |
| OTU7830 | NA | NA | NA | NA | NA | + |
| OTU2083 | + | NA | NA | NA | NA | NA |
| OTU6432 | NA | NA | NA | NA | NA | NA |
| OTU4667 | NA | NA | NA | + | NA | NA |
| OTU6388 | NA | NA | + | NA | NA | NA |
| OTU6557 | NA | NA | NA | NA | NA | NA |
| OTU4291 | NA | NA | NA | + | NA | NA |
| OTU7149 | NA | NA | + | NA | NA | NA |
| OTU5995 | NA | NA | NA | + | NA | NA |
| OTU2436 | NA | NA | + | NA | NA | NA |
| OTU4499 | + | NA | NA | NA | NA | NA |
| OTU386 | NA | NA | + | NA | NA | NA |
| OTU2782 | NA | NA | NA | NA | + | NA |
| OTU227 | NA | NA | NA | + | NA | NA |
| OTU7504 | NA | NA | NA | NA | NA | NA |
| OTU1134 | NA | NA | + | NA | NA | NA |
| OTU2388 | NA | NA | + | NA | NA | NA |
| OTU2449 | NA | NA | NA | NA | NA | NA |
| OTU2386 | NA | + | NA | + | NA | NA |
| OTU505 | NA | NA | + | NA | NA | NA |
| OTU4861 | + | NA | NA | NA | NA | NA |
| OTU5523 | NA | + | NA | NA | NA | NA |
| OTU322 | NA | NA | + | NA | NA | NA |
| OTU5526 | NA | NA | NA | NA | NA | + |
| OTU321 | NA | NA | NA | NA | NA | + |
| OTU7510 | NA | + | NA | NA | NA | NA |
| OTU8109 | NA | NA | NA | NA | NA | NA |
| OTU4855 | + | NA | NA | NA | NA | NA |
| OTU3339 | NA | NA | NA | + | NA | NA |
| OTU4324 | NA | NA | NA | + | NA | NA |
| OTU1841 | + | NA | NA | NA | NA | NA |
| OTU4294 | NA | NA | NA | + | NA | NA |
| OTU7120 | NA | NA | + | NA | NA | NA |
| OTU2116 | NA | NA | + | NA | NA | NA |
| OTU4389 | NA | NA | NA | NA | + | NA |
| OTU1091 | + | NA | NA | NA | NA | NA |
| OTU4872 | NA | NA | NA | NA | NA | NA |
| OTU898 | NA | NA | NA | + | NA | NA |
| OTU525 | NA | NA | + | NA | NA | NA |
| OTU527 | NA | NA | + | NA | NA | NA |
| OTU509 | NA | NA | + | NA | NA | NA |
| OTU4618 | NA | + | NA | NA | NA | NA |
| OTU5391 | + | NA | NA | NA | NA | NA |
| OTU2188 | + | NA | NA | NA | NA | NA |
| OTU8271 | NA | + | NA | NA | NA | NA |
| OTU5830 | NA | NA | NA | + | NA | NA |
| OTU4435 | + | NA | NA | NA | NA | NA |
| OTU4656 | + | NA | NA | NA | + | NA |
| OTU4128 | NA | NA | NA | NA | NA | NA |
| OTU586 | NA | NA | NA | NA | NA | NA |
| OTU4694 | NA | NA | + | NA | NA | NA |
| OTU2966 | NA | NA | + | NA | NA | NA |
| OTU5493 | + | NA | NA | NA | NA | NA |
| OTU4581 | NA | NA | NA | + | NA | NA |
| OTU202 | NA | NA | NA | + | NA | NA |
| OTU4552 | NA | NA | NA | NA | + | NA |
| OTU5692 | + | NA | NA | NA | NA | NA |
| OTU194 | NA | NA | + | NA | NA | NA |
| OTU1850 | NA | NA | NA | + | NA | NA |
| OTU2716 | NA | NA | NA | NA | NA | NA |
| OTU7262 | NA | NA | NA | + | NA | NA |
| OTU5450 | + | NA | NA | NA | NA | NA |
| OTU1286 | NA | NA | NA | NA | NA | NA |
| OTU4491 | NA | NA | NA | NA | NA | NA |
| OTU891 | NA | NA | NA | NA | NA | NA |
| OTU4400 | + | NA | NA | NA | NA | NA |
| OTU2347 | NA | NA | NA | NA | NA | NA |
| OTU4801 | NA | NA | NA | NA | + | NA |
| OTU1652 | NA | + | NA | NA | NA | NA |
| OTU5445 | NA | + | NA | NA | NA | NA |
| OTU4077 | NA | + | NA | NA | NA | NA |
| OTU5914 | NA | NA | NA | NA | NA | + |
| OTU144 | NA | NA | NA | + | NA | NA |
| OTU1654 | NA | NA | NA | + | NA | NA |
| OTU2832 | NA | NA | NA | NA | NA | NA |
| OTU7408 | NA | NA | NA | NA | NA | + |
| OTU5346 | NA | NA | NA | NA | NA | NA |
| OTU3796 | NA | NA | NA | + | NA | NA |
| OTU6440 | NA | NA | NA | NA | NA | NA |
| OTU2570 | NA | NA | NA | NA | + | NA |
| OTU4979 | NA | NA | NA | NA | NA | + |
| OTU6744 | NA | NA | NA | NA | NA | + |
| OTU6572 | + | NA | NA | NA | NA | NA |
| OTU6916 | NA | NA | NA | NA | NA | + |
| OTU5739 | NA | NA | NA | NA | + | NA |
| OTU5737 | NA | + | NA | NA | NA | NA |
| OTU5731 | NA | NA | NA | NA | NA | NA |
| OTU6796 | NA | NA | NA | + | NA | NA |
| OTU4812 | NA | NA | NA | NA | + | NA |
| OTU6039 | NA | NA | + | NA | NA | NA |
| OTU6336 | NA | NA | + | NA | NA | NA |
| OTU7020 | NA | NA | + | NA | NA | NA |
| OTU7311 | NA | NA | NA | NA | NA | + |
| OTU1619 | NA | NA | NA | NA | NA | + |
| OTU2781 | NA | NA | NA | + | NA | NA |
| OTU4847 | NA | NA | NA | NA | NA | NA |
| OTU6048 | NA | + | NA | NA | NA | NA |
| OTU1708 | + | NA | NA | NA | NA | NA |
| OTU2809 | NA | NA | NA | NA | NA | + |
| OTU730 | NA | NA | + | NA | NA | NA |
| OTU4566 | + | NA | NA | NA | NA | NA |
| OTU4522 | NA | NA | NA | + | NA | NA |
| OTU4653 | + | NA | NA | NA | NA | NA |
| OTU3835 | + | NA | NA | NA | NA | NA |
| OTU7048 | NA | NA | + | NA | NA | NA |
| OTU5628 | NA | NA | NA | NA | + | NA |
| OTU5843 | NA | NA | NA | NA | NA | + |
| OTU862 | NA | NA | NA | + | NA | NA |
| OTU4852 | + | NA | NA | NA | NA | NA |
| OTU1947 | NA | + | NA | NA | NA | NA |
| OTU4582 | NA | NA | NA | NA | + | NA |
| OTU7667 | NA | NA | NA | NA | + | NA |
| OTU4497 | + | NA | NA | NA | NA | NA |
| OTU6411 | NA | NA | NA | NA | NA | + |
| OTU4617 | NA | + | NA | NA | NA | NA |
| OTU627 | NA | NA | + | NA | NA | NA |
| OTU975 | NA | NA | + | NA | NA | NA |
| OTU5084 | NA | NA | + | NA | NA | NA |
| OTU6761 | + | NA | NA | NA | NA | NA |
| OTU7976 | + | NA | NA | NA | NA | NA |
| OTU5257 | NA | NA | NA | NA | + | NA |
| OTU422 | NA | NA | NA | + | NA | NA |
| OTU2990 | NA | NA | + | NA | NA | NA |
| OTU716 | NA | NA | NA | + | NA | NA |
| OTU3063 | NA | NA | NA | + | NA | NA |
| OTU7115 | NA | NA | + | NA | NA | NA |
| OTU933 | NA | NA | + | NA | NA | NA |
| OTU5267 | NA | NA | NA | NA | + | NA |
| OTU2005 | NA | NA | + | NA | NA | NA |
| OTU932 | + | NA | NA | NA | NA | NA |
| OTU4926 | + | NA | NA | NA | NA | NA |
| OTU4762 | NA | NA | NA | NA | + | NA |
| OTU2631 | + | NA | NA | NA | NA | NA |
| OTU2632 | + | NA | NA | NA | NA | NA |
| OTU776 | NA | NA | + | NA | NA | NA |
| OTU2227 | NA | NA | NA | NA | + | NA |
| OTU1776 | NA | NA | NA | NA | + | NA |
| OTU7102 | NA | NA | + | NA | NA | NA |
| OTU7108 | NA | NA | + | NA | NA | NA |
| OTU7875 | NA | NA | NA | NA | NA | NA |
| OTU1661 | NA | NA | NA | NA | NA | + |
| OTU5727 | + | + | NA | NA | NA | NA |
| OTU1180 | NA | NA | NA | + | NA | NA |
| OTU7127 | NA | NA | + | NA | NA | NA |
| OTU8130 | NA | NA | NA | NA | NA | NA |
| OTU7029 | NA | NA | + | NA | NA | NA |
| OTU2523 | NA | NA | NA | NA | NA | NA |
| OTU3110 | NA | NA | NA | + | NA | NA |
| OTU1357 | NA | NA | NA | + | NA | NA |
| OTU795 | NA | + | NA | NA | NA | NA |
| OTU1140 | NA | NA | NA | + | NA | NA |
| OTU4106 | NA | + | NA | NA | NA | NA |
| OTU4108 | NA | NA | NA | NA | + | NA |
| OTU6674 | NA | NA | NA | NA | NA | + |
| OTU3286 | NA | NA | NA | + | NA | NA |
| OTU4816 | + | NA | NA | NA | NA | NA |
| OTU4814 | NA | NA | NA | NA | + | NA |
| OTU4811 | NA | NA | NA | NA | + | NA |
| OTU7028 | NA | NA | + | NA | NA | NA |
| OTU2389 | NA | NA | NA | NA | NA | + |
| OTU2064 | NA | NA | + | NA | NA | NA |
| OTU3622 | NA | NA | NA | + | NA | NA |
| OTU139 | NA | + | NA | NA | NA | NA |
| OTU4750 | NA | NA | NA | NA | + | NA |
| OTU6032 | NA | NA | + | NA | NA | NA |
| OTU4570 | + | NA | NA | NA | NA | NA |
| OTU5594 | + | NA | NA | NA | NA | NA |
| OTU6474 | NA | NA | NA | + | NA | NA |
| OTU4893 | + | NA | NA | NA | NA | NA |
| OTU4071 | NA | NA | NA | NA | NA | NA |
| OTU1089 | NA | NA | + | NA | NA | NA |
| OTU5273 | NA | NA | NA | NA | + | NA |
| OTU2337 | NA | NA | NA | NA | NA | + |
| OTU4315 | NA | NA | NA | + | NA | NA |
| OTU1984 | NA | NA | NA | + | NA | NA |
| OTU1478 | NA | NA | NA | NA | + | NA |
| OTU6789 | NA | NA | NA | NA | NA | + |
| OTU8199 | NA | NA | NA | + | NA | NA |
| OTU4176 | NA | + | NA | NA | NA | NA |
| OTU3383 | NA | NA | NA | + | NA | + |
| OTU5834 | NA | NA | NA | NA | NA | + |
| OTU1281 | NA | + | NA | NA | NA | NA |
| OTU2411 | NA | NA | + | NA | NA | NA |
| OTU918 | NA | NA | NA | + | NA | NA |
| OTU363 | NA | NA | + | NA | NA | NA |
| OTU2414 | NA | NA | + | NA | NA | NA |
| OTU2416 | NA | NA | NA | NA | NA | + |
| OTU2320 | NA | NA | NA | NA | NA | + |
| OTU243 | NA | + | + | NA | NA | NA |
| OTU4846 | + | NA | NA | NA | NA | NA |
| OTU6509 | NA | NA | + | NA | NA | NA |
| OTU2641 | NA | NA | + | NA | NA | NA |
| OTU6847 | NA | NA | NA | + | NA | NA |
| OTU4514 | + | NA | NA | NA | NA | NA |
| OTU3454 | NA | NA | NA | + | NA | NA |
| OTU974 | NA | NA | + | NA | NA | NA |
| OTU6862 | NA | NA | NA | NA | NA | NA |
| OTU1444 | NA | NA | NA | + | NA | NA |
| OTU3475 | NA | NA | NA | + | NA | NA |
| OTU308 | NA | NA | + | NA | NA | NA |
| OTU1100 | NA | NA | + | NA | NA | NA |
| OTU3944 | NA | NA | NA | NA | NA | NA |
| OTU1886 | NA | NA | + | NA | NA | NA |
| OTU1997 | NA | + | NA | NA | NA | NA |
| OTU7170 | NA | NA | + | NA | NA | NA |
| OTU7733 | NA | NA | NA | NA | NA | + |
| OTU326 | NA | NA | + | NA | NA | NA |
| OTU6355 | NA | NA | NA | NA | + | NA |
| OTU4488 | + | NA | NA | NA | NA | NA |
| OTU1726 | NA | NA | + | NA | NA | NA |
| OTU1648 | NA | NA | + | NA | NA | NA |
| OTU1640 | NA | NA | NA | NA | + | NA |
| OTU6924 | NA | NA | NA | + | NA | + |
| OTU354 | NA | NA | + | NA | NA | NA |
| OTU3813 | NA | NA | + | NA | NA | NA |
| OTU7507 | NA | NA | NA | NA | NA | + |
| OTU7090 | NA | NA | + | NA | NA | NA |
| OTU4869 | NA | NA | NA | NA | NA | NA |
| OTU490 | NA | NA | NA | + | NA | NA |
| OTU7168 | NA | NA | + | NA | NA | NA |
| OTU3219 | NA | NA | NA | + | NA | NA |
| OTU305 | NA | NA | NA | NA | NA | NA |
| OTU5578 | + | NA | NA | NA | NA | NA |
| OTU8277 | NA | NA | + | NA | NA | + |
| OTU3106 | NA | NA | NA | + | NA | NA |
| OTU3100 | NA | NA | NA | + | NA | NA |
| OTU7424 | NA | + | NA | NA | NA | NA |
| OTU7918 | NA | + | NA | NA | NA | NA |
| OTU376 | NA | NA | + | NA | NA | NA |
| OTU2305 | NA | + | NA | NA | NA | NA |
| OTU2499 | NA | NA | NA | + | NA | NA |
| OTU3712 | NA | NA | + | NA | NA | NA |
| OTU1260 | NA | + | NA | NA | + | NA |
| OTU2453 | NA | NA | + | NA | NA | NA |
| OTU4466 | NA | NA | NA | NA | NA | NA |
| OTU1308 | NA | NA | NA | + | NA | NA |
| OTU4167 | NA | + | NA | NA | NA | NA |
| OTU5274 | NA | NA | NA | NA | + | NA |
| OTU837 | NA | NA | + | NA | NA | NA |
| OTU786 | NA | + | NA | NA | NA | NA |
| OTU3439 | NA | NA | NA | + | NA | NA |
| OTU784 | + | NA | NA | NA | NA | NA |
| OTU2244 | NA | NA | NA | + | NA | NA |
| OTU2991 | NA | NA | + | NA | NA | NA |
| OTU2995 | NA | NA | + | NA | NA | NA |
| OTU2997 | NA | NA | + | NA | NA | NA |
| OTU4998 | NA | NA | NA | NA | + | NA |
| OTU594 | NA | NA | + | NA | NA | NA |
| OTU423 | NA | NA | NA | NA | + | NA |
| OTU113 | NA | NA | + | NA | NA | NA |
| OTU1631 | NA | NA | NA | + | NA | NA |
| OTU4627 | NA | NA | NA | NA | + | NA |
| OTU3495 | NA | NA | NA | NA | + | NA |
| OTU701 | NA | NA | + | NA | NA | NA |
| OTU1941 | NA | NA | NA | NA | NA | NA |
| OTU982 | NA | NA | + | NA | NA | NA |
| OTU811 | NA | + | NA | NA | NA | NA |
| OTU3732 | NA | NA | NA | + | + | NA |
| OTU2199 | NA | NA | NA | + | NA | NA |
| OTU3423 | NA | NA | NA | + | NA | NA |
| OTU6093 | NA | + | NA | NA | NA | NA |
| OTU5513 | NA | NA | NA | + | NA | NA |
| OTU6082 | NA | NA | NA | NA | NA | + |
| OTU585 | NA | NA | + | NA | NA | NA |
| OTU587 | NA | NA | + | NA | NA | NA |
| OTU8119 | NA | NA | NA | NA | NA | NA |
| OTU5855 | NA | NA | NA | NA | NA | + |
| OTU6757 | NA | NA | + | NA | NA | NA |
| OTU4464 | + | NA | NA | NA | NA | NA |
| OTU7684 | NA | NA | NA | NA | NA | NA |
| OTU1083 | NA | NA | + | NA | NA | NA |
| OTU5754 | NA | NA | NA | NA | NA | NA |
| OTU6941 | NA | NA | NA | NA | + | NA |
| OTU5975 | NA | + | NA | NA | NA | NA |
| OTU3366 | NA | NA | NA | + | NA | NA |
| OTU5758 | + | NA | NA | NA | NA | NA |
| OTU5745 | NA | + | NA | NA | NA | + |
| OTU7467 | NA | NA | NA | NA | NA | NA |
| OTU7463 | + | NA | NA | NA | NA | NA |
| OTU5480 | NA | NA | NA | NA | NA | + |
| OTU1667 | NA | NA | + | NA | NA | NA |
| OTU5381 | NA | NA | NA | NA | NA | NA |
| OTU7233 | NA | NA | NA | NA | NA | NA |
| OTU6692 | NA | NA | NA | + | NA | NA |
| OTU6691 | NA | NA | + | NA | NA | NA |
| OTU6694 | NA | NA | NA | NA | NA | + |
| OTU4831 | + | NA | NA | NA | NA | NA |
| OTU4830 | + | NA | NA | NA | NA | NA |
| OTU4832 | + | NA | NA | NA | NA | NA |
| OTU4834 | + | NA | NA | NA | NA | NA |
| OTU729 | NA | NA | NA | NA | NA | NA |
| OTU3649 | NA | NA | NA | + | NA | NA |
| OTU5610 | NA | NA | NA | NA | NA | NA |
| OTU6377 | NA | NA | NA | NA | NA | + |
| OTU4780 | NA | NA | NA | NA | + | NA |
| OTU3683 | NA | + | NA | NA | NA | NA |
| OTU7239 | NA | NA | NA | NA | NA | + |
| OTU125 | NA | NA | + | NA | NA | NA |
| OTU4536 | + | NA | NA | NA | NA | NA |
| OTU2739 | NA | NA | NA | + | NA | NA |
| OTU7570 | NA | NA | NA | NA | NA | + |
| OTU2254 | + | NA | NA | NA | NA | NA |
| OTU1214 | NA | NA | NA | NA | NA | + |
| OTU5178 | + | NA | NA | NA | NA | NA |
| OTU1843 | NA | NA | + | NA | NA | NA |
| OTU5172 | + | NA | NA | NA | NA | NA |
| OTU3550 | NA | NA | NA | + | NA | NA |
| OTU6473 | NA | NA | NA | NA | NA | + |
| OTU256 | NA | NA | + | NA | NA | NA |
| OTU4888 | + | NA | NA | NA | NA | NA |
| OTU2737 | NA | NA | NA | NA | NA | NA |
| OTU4227 | NA | + | NA | NA | NA | NA |
| OTU2255 | NA | + | NA | NA | NA | NA |
| OTU3458 | NA | NA | NA | + | NA | NA |
| OTU265 | NA | NA | + | NA | NA | NA |
| OTU5318 | NA | NA | + | NA | NA | NA |
| OTU5319 | + | NA | NA | NA | NA | NA |
| OTU5294 | NA | NA | + | NA | NA | NA |
| OTU2639 | NA | NA | NA | NA | + | NA |
| OTU3014 | NA | NA | + | NA | NA | NA |
| OTU6517 | NA | NA | NA | NA | NA | + |
| OTU182 | NA | NA | NA | NA | NA | NA |
| OTU1222 | NA | NA | + | NA | NA | NA |
| OTU5120 | NA | NA | NA | NA | NA | NA |
| OTU6543 | NA | NA | + | NA | NA | NA |
| OTU660 | NA | NA | + | NA | + | NA |
| OTU5198 | + | NA | NA | NA | NA | NA |
| OTU5748 | NA | NA | NA | NA | + | NA |
| OTU3482 | NA | NA | NA | + | NA | NA |
| OTU2112 | + | NA | NA | NA | NA | NA |
| OTU7121 | NA | NA | + | NA | NA | NA |
| OTU7124 | NA | NA | + | NA | NA | NA |
| OTU7125 | NA | NA | + | NA | NA | NA |
| OTU1649 | NA | NA | NA | NA | NA | + |
| OTU7016 | NA | NA | + | NA | NA | NA |
| OTU1643 | NA | NA | NA | NA | + | NA |
| OTU1229 | + | NA | NA | NA | NA | NA |
| OTU5169 | NA | NA | NA | NA | + | NA |
| OTU4349 | NA | NA | NA | + | NA | NA |
| OTU8189 | NA | NA | NA | + | NA | NA |
| OTU4343 | + | NA | NA | NA | NA | NA |
| OTU4342 | NA | NA | NA | NA | NA | NA |
| OTU4346 | NA | NA | NA | + | NA | NA |
| OTU4345 | NA | + | NA | NA | NA | NA |
| OTU2694 | NA | NA | NA | NA | + | NA |
| OTU3873 | NA | NA | NA | + | NA | NA |
| OTU3020 | NA | NA | + | NA | NA | NA |
| OTU6810 | NA | NA | + | NA | NA | NA |
| OTU3027 | NA | NA | + | NA | NA | NA |
| OTU1275 | NA | NA | NA | NA | NA | + |
| OTU5024 | + | NA | NA | NA | NA | NA |
| OTU1539 | NA | NA | NA | + | NA | NA |
| OTU2185 | NA | NA | NA | NA | + | NA |
| OTU2021 | + | NA | NA | NA | NA | NA |
| OTU6373 | NA | NA | NA | + | NA | NA |
| OTU3050 | NA | NA | NA | + | NA | NA |
| OTU4164 | NA | + | NA | NA | NA | NA |
| OTU4163 | NA | + | NA | NA | NA | NA |
| OTU4690 | NA | NA | NA | NA | + | NA |
| OTU3996 | NA | NA | NA | NA | NA | NA |
| OTU4000 | + | NA | NA | NA | NA | NA |
| OTU3356 | NA | NA | NA | + | NA | NA |
| OTU7052 | NA | NA | + | NA | NA | NA |
| OTU7809 | NA | + | NA | NA | NA | NA |
| OTU4548 | NA | NA | NA | NA | NA | NA |
| OTU8207 | NA | NA | NA | NA | NA | NA |
| OTU6553 | NA | NA | NA | NA | NA | + |
| OTU6868 | NA | NA | NA | + | NA | NA |
| OTU8129 | NA | + | NA | NA | NA | NA |
| OTU1116 | NA | NA | NA | NA | + | NA |
| OTU853 | NA | + | NA | NA | NA | NA |
| OTU854 | NA | NA | NA | + | NA | NA |
| OTU2615 | NA | NA | NA | NA | + | NA |
| OTU3196 | NA | NA | + | NA | NA | NA |
| OTU3109 | NA | NA | NA | + | NA | NA |
| OTU4764 | NA | NA | NA | NA | + | NA |
| OTU4767 | NA | NA | NA | NA | + | NA |
| OTU4760 | NA | NA | NA | NA | + | NA |
| OTU4339 | NA | NA | NA | NA | NA | NA |
| OTU6645 | NA | + | NA | NA | NA | NA |
| OTU1849 | NA | NA | + | NA | NA | NA |
| OTU4574 | NA | + | NA | NA | NA | NA |
| OTU4606 | NA | NA | NA | NA | + | NA |
| OTU7966 | NA | NA | + | NA | NA | NA |
| OTU2516 | NA | NA | NA | + | NA | NA |
| OTU4319 | NA | NA | NA | + | NA | NA |
| OTU2507 | NA | + | NA | NA | NA | NA |
| OTU3674 | NA | NA | + | NA | NA | NA |
| OTU5360 | NA | NA | + | NA | NA | NA |
| OTU2034 | NA | NA | NA | NA | NA | + |
| OTU1007 | NA | NA | + | NA | NA | NA |
| OTU4473 | + | NA | NA | NA | NA | NA |
| OTU1799 | NA | NA | + | NA | NA | NA |
| OTU1960 | NA | NA | NA | NA | NA | NA |
| OTU4195 | NA | + | NA | NA | NA | NA |
| OTU6123 | NA | NA | + | NA | NA | NA |
| OTU1250 | NA | NA | + | NA | NA | NA |
| OTU3802 | NA | NA | NA | NA | NA | NA |
| OTU8126 | NA | NA | + | NA | NA | NA |
| OTU4329 | NA | NA | NA | + | NA | NA |
| OTU4563 | NA | + | NA | NA | NA | NA |
| OTU5246 | NA | NA | NA | NA | NA | NA |
| OTU5778 | NA | NA | NA | NA | NA | + |
| OTU1132 | NA | NA | + | NA | NA | NA |
| OTU5958 | NA | NA | NA | NA | NA | NA |
| OTU5776 | NA | + | NA | NA | NA | NA |
| OTU1973 | NA | NA | NA | NA | NA | NA |
| OTU6611 | NA | NA | NA | NA | NA | + |
| OTU4572 | NA | NA | NA | NA | + | NA |
| OTU4573 | NA | + | NA | NA | + | NA |
| OTU7448 | NA | NA | + | NA | NA | NA |
| OTU4575 | NA | + | NA | NA | NA | NA |
| OTU4578 | NA | NA | NA | NA | NA | NA |
| OTU4579 | NA | NA | NA | NA | + | NA |
| OTU4021 | NA | NA | + | NA | NA | NA |
| OTU833 | NA | NA | NA | NA | NA | NA |
| OTU5423 | + | NA | NA | NA | NA | NA |
| OTU4593 | NA | NA | NA | NA | + | NA |
| OTU2625 | NA | + | NA | NA | NA | NA |
| OTU5858 | NA | NA | NA | NA | NA | + |
| OTU4583 | NA | NA | NA | NA | + | NA |
| OTU1172 | NA | NA | + | NA | NA | NA |
| OTU651 | NA | NA | NA | NA | NA | NA |
| OTU652 | NA | NA | + | NA | NA | NA |
| OTU7998 | + | NA | NA | NA | NA | NA |
| OTU7990 | + | NA | NA | NA | NA | NA |
| OTU3874 | NA | NA | NA | + | NA | NA |
| OTU7433 | NA | NA | NA | NA | + | NA |
| OTU4766 | NA | NA | NA | NA | + | NA |
| OTU1167 | + | NA | NA | NA | + | NA |
| OTU7138 | NA | NA | + | NA | NA | NA |
| OTU3870 | NA | NA | NA | NA | NA | + |
| OTU7800 | NA | NA | NA | + | NA | NA |
| OTU4730 | NA | + | NA | NA | + | NA |
| OTU4125 | NA | NA | NA | + | NA | NA |
| OTU2378 | NA | NA | NA | + | NA | NA |
| OTU7364 | NA | NA | NA | NA | NA | NA |
| OTU5984 | NA | + | NA | NA | NA | NA |
| OTU6554 | NA | NA | NA | NA | NA | + |
| OTU7625 | + | NA | NA | + | NA | NA |
| OTU5308 | NA | NA | + | NA | NA | NA |
| OTU5585 | + | NA | NA | NA | NA | NA |
| OTU2713 | NA | NA | NA | + | NA | NA |
| OTU2711 | + | NA | NA | NA | NA | NA |
| OTU7752 | NA | + | NA | NA | NA | NA |
| OTU899 | NA | NA | + | NA | NA | NA |
| OTU4585 | NA | + | NA | NA | NA | NA |
| OTU3011 | NA | NA | NA | + | NA | NA |
| OTU8003 | NA | NA | NA | NA | NA | NA |
| OTU5635 | + | NA | NA | NA | NA | NA |
| OTU6324 | NA | NA | NA | + | NA | NA |
| OTU5241 | NA | NA | NA | NA | + | NA |
| OTU7588 | NA | + | NA | NA | NA | NA |
| OTU5570 | NA | NA | NA | NA | + | NA |
| OTU5574 | NA | NA | NA | NA | + | NA |
| OTU4297 | NA | NA | NA | + | NA | NA |
| OTU5187 | NA | + | NA | NA | NA | NA |
| OTU4403 | + | NA | NA | NA | NA | NA |
| OTU896 | NA | NA | + | NA | NA | NA |
| OTU1032 | NA | NA | NA | NA | NA | NA |
| OTU2891 | NA | NA | NA | + | NA | NA |
| OTU6501 | NA | NA | + | NA | NA | NA |
| OTU2179 | NA | NA | NA | + | NA | NA |
| OTU2209 | + | NA | NA | NA | NA | NA |
| OTU3521 | NA | NA | NA | NA | NA | + |
| OTU661 | NA | NA | + | NA | NA | NA |
| OTU680 | NA | NA | + | NA | NA | NA |
| OTU8050 | NA | NA | NA | NA | NA | + |
| OTU7141 | NA | NA | + | NA | NA | NA |
| OTU2673 | NA | NA | NA | + | NA | NA |
| OTU7148 | NA | NA | + | NA | NA | NA |
| OTU7144 | NA | NA | NA | NA | NA | NA |
| OTU7145 | NA | NA | + | NA | NA | NA |
| OTU3031 | NA | NA | NA | NA | NA | + |
| OTU5464 | + | NA | NA | NA | NA | NA |
| OTU3163 | NA | + | NA | NA | NA | NA |
| OTU5153 | + | NA | NA | NA | NA | NA |
| OTU3782 | NA | NA | NA | + | NA | NA |
| OTU1063 | NA | + | NA | NA | NA | NA |
| OTU3003 | + | NA | NA | NA | NA | NA |
| OTU876 | NA | NA | + | NA | NA | NA |
| OTU5891 | NA | NA | NA | NA | NA | + |
| OTU5892 | NA | NA | NA | NA | NA | + |
| OTU8151 | NA | NA | NA | + | NA | NA |
| OTU5263 | NA | NA | NA | NA | + | NA |
| OTU3030 | NA | NA | + | NA | NA | NA |
| OTU101 | NA | NA | NA | + | NA | NA |
| OTU2550 | NA | NA | NA | + | NA | NA |
| OTU6570 | NA | NA | NA | NA | NA | + |
| OTU2045 | NA | NA | NA | NA | NA | NA |
| OTU4704 | NA | + | NA | + | NA | NA |
| OTU3666 | NA | NA | NA | + | NA | NA |
| OTU5174 | + | NA | NA | NA | NA | NA |
| OTU4800 | NA | NA | NA | NA | + | NA |
| OTU7133 | NA | NA | + | NA | NA | NA |
| OTU5232 | NA | NA | NA | NA | + | NA |
| OTU355 | NA | NA | + | NA | NA | NA |
| OTU3290 | NA | NA | NA | NA | NA | NA |
| OTU2282 | NA | NA | NA | NA | + | NA |
| OTU583 | NA | NA | + | NA | NA | NA |
| OTU5792 | NA | NA | NA | + | NA | NA |
| OTU4896 | + | NA | NA | NA | NA | NA |
| OTU3476 | NA | NA | NA | + | NA | NA |
| OTU2734 | NA | NA | NA | + | NA | NA |
| OTU6206 | NA | NA | + | NA | NA | NA |
| OTU1057 | + | NA | NA | NA | NA | NA |
| OTU7128 | NA | NA | + | NA | NA | NA |
| OTU2714 | NA | NA | NA | + | NA | NA |
| OTU7622 | NA | NA | NA | NA | NA | + |
| OTU7623 | NA | NA | NA | + | NA | NA |
| OTU2018 | NA | NA | + | NA | NA | NA |
| OTU3449 | NA | NA | NA | + | NA | NA |
| OTU4141 | NA | + | NA | NA | NA | NA |
| OTU4144 | NA | NA | NA | + | NA | NA |
| OTU4147 | NA | + | NA | NA | NA | NA |
| OTU4149 | NA | + | NA | NA | NA | NA |
| OTU4148 | NA | + | NA | NA | NA | NA |
| OTU4829 | + | NA | NA | NA | NA | NA |
| OTU2575 | NA | NA | NA | NA | NA | NA |
| OTU6712 | NA | NA | NA | NA | NA | + |
| OTU3450 | NA | NA | NA | + | NA | NA |
| OTU289 | NA | NA | NA | NA | NA | NA |
| OTU498 | NA | NA | NA | NA | NA | NA |
| OTU4673 | NA | NA | + | NA | NA | NA |
| OTU284 | NA | NA | + | NA | NA | NA |
| OTU5138 | NA | NA | NA | NA | + | NA |
| OTU7161 | NA | NA | + | NA | NA | NA |
| OTU5133 | NA | NA | NA | NA | + | NA |
| OTU3150 | NA | NA | NA | NA | NA | NA |
| OTU3077 | NA | NA | NA | + | NA | NA |
| OTU809 | NA | NA | NA | NA | NA | NA |
| OTU2418 | NA | + | NA | NA | NA | NA |
| OTU6783 | NA | NA | NA | + | NA | NA |
| OTU5250 | NA | NA | NA | NA | + | NA |
| OTU1473 | NA | NA | NA | NA | NA | NA |
| OTU5230 | NA | NA | NA | NA | + | NA |
| OTU7787 | NA | NA | NA | NA | NA | NA |
| OTU940 | + | NA | NA | NA | NA | NA |
| OTU8060 | NA | NA | NA | NA | NA | + |
| OTU6150 | NA | + | NA | NA | NA | NA |
| OTU4835 | + | NA | NA | NA | NA | NA |
| OTU3981 | NA | NA | NA | NA | NA | NA |
| OTU2466 | NA | NA | NA | NA | NA | NA |
| OTU4520 | + | NA | NA | NA | NA | NA |
| OTU682 | NA | NA | + | NA | NA | NA |
| OTU5240 | NA | NA | NA | NA | + | NA |
| OTU299 | NA | NA | NA | NA | + | NA |
| OTU8276 | NA | NA | + | NA | NA | NA |
| OTU3508 | + | + | NA | + | NA | NA |
| OTU6970 | NA | NA | NA | NA | NA | + |
| OTU5361 | NA | NA | NA | NA | NA | NA |
| OTU2855 | NA | NA | NA | NA | NA | NA |
| OTU1519 | NA | NA | + | NA | NA | NA |
| OTU7683 | NA | NA | + | NA | NA | NA |
| OTU3490 | NA | NA | NA | + | NA | NA |
| OTU3151 | NA | + | NA | NA | NA | NA |
| OTU211 | NA | NA | + | NA | NA | NA |
| OTU7648 | NA | + | NA | NA | NA | NA |
| OTU6841 | NA | + | + | NA | NA | NA |
| OTU6848 | NA | + | NA | NA | NA | NA |
| OTU3235 | + | NA | NA | NA | NA | + |
| OTU5888 | NA | NA | NA | NA | NA | + |
| OTU4952 | + | NA | NA | NA | NA | NA |
| OTU97 | NA | NA | + | NA | NA | NA |
| OTU6795 | NA | NA | NA | NA | NA | + |
| OTU582 | NA | NA | NA | NA | NA | NA |
| OTU699 | NA | NA | NA | NA | + | NA |
| OTU8019 | NA | NA | NA | NA | NA | + |
| OTU5688 | NA | NA | NA | + | NA | NA |
| OTU567 | NA | NA | NA | NA | NA | NA |
| OTU543 | NA | NA | + | NA | NA | NA |
| OTU540 | NA | NA | + | NA | NA | NA |
| OTU541 | NA | + | + | NA | NA | NA |
| OTU547 | NA | NA | + | NA | NA | NA |
| OTU548 | NA | NA | NA | NA | NA | NA |
| OTU225 | NA | NA | + | NA | NA | NA |
| OTU2763 | NA | + | NA | NA | NA | NA |
| OTU3893 | NA | NA | NA | NA | + | NA |
| OTU1636 | NA | + | NA | NA | NA | NA |
| OTU1048 | NA | NA | + | NA | NA | NA |
| OTU1909 | NA | NA | NA | NA | NA | + |
| OTU996 | NA | NA | + | NA | NA | NA |
| OTU6096 | NA | NA | + | NA | NA | NA |
| OTU5121 | + | NA | NA | NA | NA | NA |
| OTU8091 | + | NA | NA | NA | NA | NA |
| OTU4562 | NA | NA | NA | NA | + | NA |
| OTU5129 | + | NA | NA | NA | NA | NA |
| OTU1236 | NA | NA | NA | NA | + | NA |
| OTU6346 | NA | NA | NA | NA | NA | + |
| OTU1231 | NA | NA | NA | NA | NA | + |
| OTU3302 | NA | NA | NA | + | + | NA |
| OTU6932 | NA | NA | NA | NA | NA | + |
| OTU4592 | NA | NA | NA | NA | + | NA |
| OTU7509 | NA | NA | NA | + | NA | + |
| OTU4556 | + | NA | NA | NA | NA | NA |
| OTU4188 | NA | + | NA | NA | NA | NA |
| OTU4478 | + | NA | NA | NA | NA | NA |
| OTU3121 | NA | + | NA | NA | NA | NA |
| OTU4166 | NA | + | NA | NA | NA | NA |
| OTU5552 | NA | NA | + | NA | NA | NA |
| OTU4300 | NA | NA | NA | + | NA | NA |
| OTU7013 | NA | NA | + | NA | NA | NA |
| OTU8222 | NA | NA | + | NA | NA | NA |
| OTU7077 | NA | NA | + | NA | NA | NA |
| OTU5854 | NA | NA | NA | NA | NA | + |
| OTU4266 | NA | NA | NA | + | NA | NA |
| OTU4802 | + | NA | NA | NA | NA | NA |
| OTU7309 | NA | + | NA | NA | NA | NA |
| OTU4705 | NA | + | NA | NA | NA | NA |
| OTU1968 | NA | NA | NA | + | NA | NA |
| OTU4554 | NA | NA | NA | NA | + | NA |
| OTU860 | NA | NA | NA | NA | NA | NA |
| OTU3038 | NA | NA | + | NA | NA | NA |
| OTU5242 | NA | NA | NA | NA | + | NA |
| OTU1074 | NA | NA | + | NA | NA | NA |
| OTU1311 | NA | NA | NA | NA | NA | NA |
| OTU214 | NA | NA | NA | NA | NA | NA |
| OTU7248 | + | NA | NA | NA | NA | NA |
| OTU4193 | NA | NA | + | NA | NA | NA |
| OTU4465 | + | NA | NA | NA | NA | NA |
| OTU3670 | NA | NA | NA | NA | + | NA |
| OTU7837 | NA | NA | NA | + | NA | NA |
| OTU7739 | NA | NA | NA | NA | NA | + |
| OTU7213 | NA | NA | + | NA | NA | NA |
| OTU4898 | + | NA | NA | NA | NA | NA |
| OTU1415 | NA | NA | + | NA | + | NA |
| OTU1579 | NA | NA | NA | + | NA | NA |
| OTU4174 | NA | + | NA | NA | NA | NA |
| OTU7934 | NA | NA | NA | + | NA | NA |
| OTU4317 | NA | NA | NA | + | NA | NA |
| OTU5497 | NA | + | NA | NA | NA | NA |
| OTU524 | NA | NA | + | NA | NA | NA |
| OTU3910 | NA | NA | NA | NA | + | NA |
| OTU2007 | NA | NA | NA | NA | NA | NA |
| OTU4895 | + | NA | NA | NA | NA | NA |
| OTU4336 | NA | NA | NA | + | NA | NA |
| OTU7534 | NA | NA | NA | + | NA | NA |
| OTU3051 | NA | NA | NA | + | NA | NA |
| OTU4541 | NA | NA | NA | NA | + | NA |
| OTU7767 | NA | NA | NA | + | NA | NA |
| OTU7814 | NA | NA | + | NA | NA | NA |
| OTU242 | NA | NA | NA | + | NA | NA |
| OTU7908 | NA | NA | NA | + | NA | NA |
| OTU7166 | NA | NA | + | NA | NA | NA |
| OTU7165 | NA | NA | NA | NA | NA | NA |
| OTU7160 | NA | NA | + | NA | NA | NA |
| OTU622 | NA | NA | + | NA | NA | NA |
| OTU7516 | NA | NA | NA | NA | + | NA |
| OTU819 | NA | NA | NA | + | NA | NA |
| OTU4826 | NA | NA | NA | NA | + | NA |
| OTU5890 | NA | NA | NA | NA | NA | + |
| OTU4303 | NA | NA | NA | + | NA | NA |
| OTU4302 | NA | NA | NA | + | NA | NA |
| OTU6091 | NA | NA | + | NA | NA | NA |
| OTU3067 | NA | NA | NA | NA | NA | NA |
| OTU5374 | NA | NA | NA | + | NA | NA |
| OTU733 | NA | NA | NA | + | NA | NA |
| OTU737 | NA | NA | NA | NA | NA | NA |
| OTU793 | NA | + | NA | NA | NA | NA |
| OTU5248 | NA | NA | NA | NA | + | NA |
| OTU3960 | NA | NA | NA | + | NA | NA |
| OTU2139 | NA | + | NA | NA | NA | NA |
| OTU1659 | NA | NA | + | NA | NA | NA |
| OTU3255 | + | NA | NA | NA | NA | NA |
| OTU4427 | + | NA | NA | NA | NA | NA |
| OTU4752 | NA | NA | NA | NA | + | NA |
| OTU5050 | NA | NA | NA | NA | + | NA |
| OTU6827 | NA | NA | NA | NA | NA | NA |
| OTU6821 | NA | + | NA | NA | NA | NA |
| OTU6791 | NA | NA | NA | + | NA | NA |
| OTU3078 | NA | NA | NA | + | NA | NA |
| OTU2795 | NA | NA | + | NA | NA | NA |
| OTU5916 | NA | NA | + | NA | NA | NA |
| OTU1898 | NA | NA | NA | + | NA | NA |
| OTU6367 | NA | NA | NA | NA | NA | + |
| OTU1033 | NA | NA | + | NA | NA | NA |
| OTU5735 | NA | + | NA | NA | NA | NA |
| OTU4911 | NA | NA | NA | + | NA | NA |
| OTU7295 | NA | NA | + | NA | NA | NA |
| OTU2880 | NA | + | + | NA | NA | NA |
| OTU5886 | NA | NA | NA | + | NA | NA |
| OTU7269 | NA | NA | NA | NA | NA | NA |
| OTU437 | NA | NA | + | NA | NA | NA |
| OTU1297 | NA | NA | NA | + | NA | NA |
| OTU8116 | NA | NA | NA | NA | NA | + |
| OTU349 | NA | NA | NA | NA | + | NA |
| OTU5235 | NA | NA | NA | NA | + | NA |
| OTU2126 | NA | + | NA | NA | NA | NA |
| OTU1219 | NA | NA | NA | NA | NA | NA |
| OTU5394 | NA | NA | + | NA | NA | NA |
| OTU1210 | NA | NA | NA | + | NA | NA |
| OTU1740 | NA | NA | NA | NA | NA | NA |
| OTU4422 | + | NA | NA | NA | NA | NA |
| OTU4528 | + | NA | NA | NA | NA | NA |
| OTU1142 | NA | NA | + | NA | NA | NA |
| OTU5911 | NA | + | NA | NA | NA | NA |
| OTU1935 | NA | NA | + | NA | NA | NA |
| OTU4539 | NA | NA | NA | NA | + | NA |
| OTU161 | NA | + | NA | NA | NA | NA |
| OTU4535 | + | NA | NA | NA | NA | NA |
| OTU4537 | + | NA | NA | NA | NA | NA |
| OTU4531 | + | NA | NA | NA | NA | NA |
| OTU6178 | NA | NA | NA | + | NA | NA |
| OTU966 | NA | NA | + | NA | NA | NA |
| OTU8114 | NA | NA | NA | NA | NA | NA |
| OTU6579 | + | NA | NA | NA | NA | NA |
| OTU3082 | NA | NA | NA | + | NA | NA |
| OTU561 | NA | NA | + | NA | NA | NA |
| OTU6991 | NA | NA | NA | NA | NA | NA |
| OTU7959 | NA | NA | NA | NA | NA | NA |
| OTU2231 | NA | NA | NA | + | NA | NA |
| OTU5302 | NA | NA | NA | NA | NA | + |
| OTU605 | NA | NA | NA | NA | NA | NA |
| OTU4948 | NA | NA | NA | NA | + | NA |
| OTU6739 | NA | + | NA | NA | NA | NA |
| OTU1767 | NA | NA | + | NA | NA | NA |
| OTU5222 | NA | NA | NA | NA | NA | NA |
| OTU3908 | NA | NA | NA | NA | + | NA |
| OTU4503 | + | NA | NA | NA | NA | NA |
| OTU3644 | NA | NA | + | NA | NA | NA |
| OTU4443 | + | NA | NA | NA | NA | NA |
| OTU5276 | NA | NA | NA | NA | + | NA |
| OTU5271 | NA | NA | NA | NA | + | NA |
| OTU2593 | NA | NA | NA | NA | + | NA |
| OTU8158 | NA | NA | + | NA | NA | NA |
| OTU2456 | + | NA | NA | NA | NA | NA |
| OTU151 | + | + | NA | NA | NA | NA |
| OTU7522 | NA | NA | NA | NA | + | NA |
| OTU3680 | + | NA | NA | + | NA | NA |
| OTU3112 | NA | NA | NA | + | NA | NA |
| OTU433 | NA | NA | NA | + | NA | NA |
| OTU2949 | NA | NA | + | NA | NA | NA |
| OTU6365 | NA | NA | NA | NA | NA | NA |
| OTU3918 | NA | NA | NA | NA | NA | NA |
| OTU5990 | NA | NA | + | NA | NA | NA |
| OTU7785 | NA | NA | NA | + | NA | NA |
| OTU1367 | NA | NA | NA | + | NA | NA |
| OTU6290 | NA | NA | NA | + | NA | NA |
| OTU2796 | NA | NA | NA | NA | NA | NA |
| OTU507 | NA | NA | + | NA | NA | NA |
| OTU2444 | NA | NA | NA | NA | NA | NA |
| OTU565 | NA | NA | + | NA | NA | NA |
| OTU562 | NA | NA | + | NA | NA | NA |
| OTU52 | NA | NA | NA | NA | NA | + |
| OTU4777 | NA | NA | NA | NA | + | NA |
| OTU7129 | NA | NA | NA | NA | NA | NA |
| OTU4092 | NA | NA | NA | NA | + | NA |
| OTU4321 | NA | NA | NA | + | NA | NA |
| OTU292 | NA | NA | NA | + | NA | NA |
| OTU7596 | NA | NA | NA | NA | NA | + |
| OTU3083 | NA | NA | NA | + | + | NA |
| OTU7055 | NA | NA | + | NA | NA | NA |
| OTU4969 | NA | NA | NA | NA | NA | NA |
| OTU343 | NA | NA | + | NA | NA | NA |
| OTU5701 | + | NA | NA | NA | NA | NA |
| OTU3825 | NA | NA | + | NA | NA | NA |
| OTU6343 | NA | NA | NA | NA | NA | NA |
| OTU1946 | NA | NA | + | NA | NA | NA |
| OTU7189 | NA | + | NA | NA | NA | NA |
| OTU7180 | NA | NA | + | NA | NA | NA |
| OTU7183 | NA | NA | + | NA | NA | NA |
| OTU5775 | NA | NA | NA | NA | NA | NA |
| OTU4980 | + | NA | NA | NA | NA | NA |
| OTU4320 | NA | NA | NA | + | NA | NA |
| OTU4323 | NA | NA | NA | + | NA | NA |
| OTU4145 | NA | + | NA | NA | NA | NA |
| OTU7109 | NA | NA | + | NA | NA | NA |
| OTU7983 | NA | NA | NA | NA | NA | + |
| OTU7985 | NA | NA | NA | NA | NA | + |
| OTU705 | NA | NA | + | NA | NA | NA |
| OTU710 | NA | NA | + | NA | NA | NA |
| OTU6728 | NA | NA | + | NA | NA | NA |
| OTU2376 | NA | NA | NA | + | NA | NA |
| OTU3598 | NA | NA | + | NA | NA | NA |
| OTU4241 | NA | NA | NA | + | NA | NA |
| OTU4247 | NA | NA | NA | + | NA | NA |
| OTU8042 | + | NA | NA | NA | NA | NA |
| OTU3243 | NA | + | NA | NA | NA | NA |
| OTU7525 | NA | + | NA | NA | NA | NA |
| OTU3043 | NA | NA | NA | + | NA | NA |
| OTU6581 | NA | NA | NA | NA | NA | + |
| OTU5865 | NA | NA | NA | NA | NA | + |
| OTU1612 | NA | NA | NA | NA | + | NA |
| OTU4518 | NA | NA | NA | NA | NA | NA |
| OTU671 | + | NA | NA | NA | NA | NA |
| OTU4666 | NA | + | NA | NA | NA | NA |
| OTU410 | NA | NA | NA | + | NA | NA |
| OTU3380 | NA | NA | NA | NA | NA | + |
| OTU2585 | NA | NA | NA | + | NA | NA |
| OTU2581 | NA | + | NA | NA | NA | NA |
| OTU7091 | NA | NA | NA | + | NA | NA |
| OTU7804 | NA | NA | NA | NA | NA | + |
| OTU6378 | + | NA | NA | + | NA | NA |
| OTU6276 | NA | + | NA | NA | NA | NA |
| OTU2163 | NA | NA | NA | + | + | NA |
| OTU2726 | NA | NA | + | NA | NA | NA |
| OTU2724 | + | NA | NA | NA | NA | NA |
| OTU5157 | NA | NA | NA | NA | + | NA |
| OTU3540 | NA | NA | + | NA | NA | NA |
| OTU2717 | NA | NA | NA | + | NA | NA |
| OTU4183 | NA | + | NA | NA | NA | NA |
| OTU3060 | NA | NA | NA | + | NA | NA |
| OTU6953 | NA | NA | NA | + | NA | NA |
| OTU3209 | + | NA | NA | NA | NA | NA |
| OTU7468 | NA | NA | NA | + | NA | NA |
| OTU4246 | NA | NA | NA | + | NA | NA |
| OTU4783 | NA | NA | NA | NA | + | NA |
| OTU3438 | NA | NA | NA | + | NA | NA |
| OTU4663 | NA | NA | NA | NA | NA | NA |
| OTU3405 | NA | NA | NA | + | NA | NA |
| OTU7310 | NA | NA | + | NA | NA | NA |
| OTU577 | NA | NA | + | NA | NA | NA |
| OTU659 | NA | NA | + | NA | NA | NA |
| OTU1003 | NA | NA | NA | NA | NA | + |
| OTU1002 | NA | NA | + | NA | NA | NA |
| OTU5056 | NA | NA | NA | NA | + | NA |
| OTU719 | NA | NA | NA | + | NA | NA |
| OTU3399 | NA | NA | NA | + | NA | NA |
| OTU5179 | NA | NA | NA | + | NA | NA |
| OTU6735 | NA | NA | NA | + | NA | NA |
| OTU4629 | + | NA | NA | NA | NA | NA |
| OTU2159 | NA | NA | NA | NA | + | NA |
| OTU6222 | NA | NA | NA | NA | NA | + |
| OTU6333 | NA | NA | NA | NA | + | NA |
| OTU6114 | NA | NA | NA | + | NA | NA |
| OTU5278 | NA | NA | NA | NA | + | NA |
| OTU909 | NA | + | NA | NA | NA | NA |
| OTU1554 | NA | NA | NA | NA | NA | NA |
| OTU6913 | NA | NA | NA | + | NA | NA |
| OTU635 | NA | NA | + | NA | NA | NA |
| OTU1684 | NA | NA | NA | NA | NA | NA |
| OTU3687 | + | NA | NA | NA | NA | NA |
| OTU3098 | NA | NA | NA | NA | NA | NA |
| OTU3722 | NA | NA | NA | NA | NA | NA |
| OTU3096 | NA | NA | NA | NA | + | NA |
| OTU3095 | NA | NA | NA | + | NA | NA |
| OTU5200 | NA | NA | NA | NA | + | NA |
| OTU232 | NA | + | NA | + | NA | NA |
| OTU2974 | NA | NA | + | NA | NA | NA |
| OTU4139 | NA | NA | NA | NA | NA | NA |
| OTU2118 | NA | NA | NA | NA | NA | NA |
| OTU3549 | NA | + | + | NA | NA | NA |
| OTU3543 | NA | NA | + | NA | NA | NA |
| OTU359 | NA | NA | NA | NA | NA | NA |
| OTU4281 | NA | NA | NA | + | NA | NA |
| OTU1774 | NA | NA | NA | NA | NA | NA |
| OTU3829 | NA | NA | NA | NA | NA | NA |
| OTU1178 | + | NA | NA | NA | NA | NA |
| OTU3793 | NA | NA | NA | NA | NA | NA |
| OTU4408 | + | NA | NA | NA | NA | NA |
| OTU5842 | NA | NA | NA | NA | NA | + |
| OTU6809 | NA | NA | NA | + | NA | NA |
| OTU6806 | NA | NA | NA | NA | NA | + |
| OTU6807 | NA | NA | + | NA | NA | NA |
| OTU6803 | NA | NA | NA | NA | NA | + |
| OTU7235 | NA | NA | NA | NA | NA | + |
| OTU159 | NA | NA | NA | + | NA | NA |
| OTU8211 | NA | NA | + | NA | NA | NA |
| OTU2264 | NA | NA | NA | NA | + | NA |
| OTU526 | NA | NA | NA | NA | NA | NA |
| OTU1194 | NA | NA | + | NA | NA | NA |
| OTU3167 | NA | NA | NA | NA | NA | NA |
| OTU502 | NA | NA | + | NA | NA | NA |
| OTU3877 | NA | NA | NA | + | NA | NA |
| OTU4907 | + | NA | NA | NA | NA | NA |
| OTU3677 | NA | NA | NA | + | NA | NA |
| OTU4214 | NA | + | NA | NA | NA | NA |
| OTU7663 | NA | NA | + | NA | NA | NA |
| OTU7212 | + | NA | NA | NA | NA | NA |
| OTU783 | NA | NA | + | NA | NA | NA |
| OTU804 | NA | NA | NA | + | NA | NA |
| OTU2913 | + | NA | NA | NA | NA | NA |
| OTU1604 | NA | NA | NA | + | NA | NA |
| OTU6479 | NA | NA | + | NA | NA | NA |
| OTU3198 | NA | NA | NA | NA | + | NA |
| OTU6558 | NA | NA | NA | NA | NA | + |
| OTU6408 | NA | NA | NA | NA | NA | NA |
| OTU7520 | NA | NA | NA | NA | NA | NA |
| OTU6697 | NA | NA | NA | NA | NA | NA |
| OTU6495 | NA | NA | NA | + | NA | NA |
| OTU2865 | NA | NA | NA | + | NA | NA |
| OTU3025 | NA | NA | + | NA | NA | NA |
| OTU3021 | NA | NA | + | NA | NA | NA |
| OTU84 | NA | NA | NA | NA | NA | + |
| OTU4553 | NA | NA | NA | NA | + | NA |
| OTU1101 | NA | NA | NA | NA | NA | + |
| OTU7306 | NA | NA | NA | NA | NA | NA |
| OTU8101 | NA | NA | + | NA | NA | NA |
| OTU2151 | NA | NA | NA | NA | + | NA |
| OTU1492 | NA | NA | NA | NA | NA | NA |
| OTU5985 | NA | NA | + | NA | NA | NA |
| OTU4516 | + | NA | NA | NA | NA | NA |
| OTU4515 | + | NA | NA | NA | NA | NA |
| OTU4510 | + | NA | NA | NA | NA | NA |
| OTU260 | NA | NA | NA | + | NA | NA |
| OTU4595 | + | NA | NA | NA | + | NA |
| OTU4534 | NA | NA | NA | NA | NA | NA |
| OTU1413 | + | NA | NA | NA | NA | NA |
| OTU1021 | NA | NA | NA | NA | NA | NA |
| OTU3224 | + | NA | NA | NA | NA | NA |
| OTU5924 | NA | NA | NA | NA | + | NA |
| OTU6468 | NA | NA | NA | + | NA | NA |
| OTU4207 | NA | + | NA | NA | NA | NA |
| OTU7438 | NA | + | NA | NA | NA | NA |
| OTU7431 | NA | NA | NA | + | NA | NA |
| OTU4527 | + | NA | NA | NA | NA | NA |
| OTU3394 | NA | NA | NA | + | NA | NA |
| OTU2364 | NA | NA | + | NA | NA | NA |
| OTU5472 | + | NA | NA | NA | NA | NA |
| OTU7912 | + | NA | NA | NA | NA | NA |
| OTU2623 | NA | + | NA | NA | NA | NA |
| OTU5436 | + | NA | NA | NA | NA | + |
| OTU788 | + | NA | NA | NA | NA | NA |
| OTU4635 | NA | + | NA | NA | NA | NA |
| OTU7447 | NA | NA | NA | + | NA | NA |
| OTU178 | NA | NA | + | + | NA | NA |
| OTU8233 | NA | NA | + | NA | NA | NA |
| OTU170 | + | NA | NA | NA | + | NA |
| OTU8241 | NA | NA | + | NA | NA | NA |
| OTU3525 | NA | NA | NA | NA | NA | NA |
| OTU7430 | NA | + | NA | NA | NA | NA |
| OTU2961 | NA | NA | + | NA | NA | NA |
| OTU3875 | NA | + | NA | NA | NA | NA |
| OTU3048 | NA | NA | NA | + | NA | NA |
| OTU497 | NA | NA | NA | NA | NA | NA |
| OTU3360 | NA | NA | NA | NA | NA | NA |
| OTU8013 | NA | NA | NA | NA | NA | NA |
| OTU520 | NA | NA | + | NA | NA | NA |
| OTU1107 | NA | NA | NA | + | NA | NA |
| OTU2984 | NA | NA | + | NA | NA | NA |
| OTU1535 | NA | NA | NA | NA | NA | NA |
| OTU1020 | NA | NA | + | NA | + | NA |
| OTU1022 | NA | NA | NA | NA | NA | + |
| OTU5077 | NA | NA | + | NA | NA | NA |
| OTU7491 | NA | NA | NA | NA | NA | + |
| OTU5078 | NA | NA | NA | + | NA | NA |
| OTU2598 | NA | + | NA | NA | + | NA |
| OTU7724 | + | NA | NA | NA | NA | NA |
| OTU1438 | NA | NA | NA | NA | NA | NA |
| OTU5054 | NA | + | NA | NA | NA | NA |
| OTU6758 | NA | NA | NA | NA | NA | NA |
| OTU7686 | NA | NA | NA | NA | NA | + |
| OTU187 | NA | NA | NA | + | NA | NA |
| OTU6129 | NA | NA | + | NA | NA | NA |
| OTU2142 | NA | + | NA | NA | NA | NA |
| OTU513 | NA | NA | + | NA | NA | NA |
| OTU981 | NA | NA | NA | NA | NA | NA |
| OTU8011 | NA | NA | + | NA | NA | NA |
| OTU8010 | NA | NA | NA | NA | NA | NA |
| OTU1331 | NA | NA | NA | NA | NA | NA |
| OTU775 | NA | NA | NA | NA | NA | + |
| OTU2653 | NA | NA | NA | NA | + | NA |
| OTU551 | NA | NA | + | NA | NA | NA |
| OTU8237 | NA | NA | + | NA | NA | NA |
| OTU8235 | NA | NA | + | NA | NA | NA |
| OTU5893 | NA | NA | NA | NA | NA | + |
| OTU4429 | NA | NA | NA | NA | NA | NA |
| OTU654 | NA | NA | + | NA | NA | NA |
| OTU6751 | NA | NA | NA | NA | NA | + |
| OTU8163 | NA | NA | + | NA | NA | NA |
| OTU6825 | NA | NA | NA | NA | NA | NA |
| OTU1446 | NA | NA | + | NA | NA | NA |
| OTU2487 | NA | NA | NA | NA | NA | NA |
| OTU1857 | NA | NA | + | NA | NA | NA |
| OTU3693 | + | NA | NA | NA | + | NA |
| OTU5279 | NA | NA | + | NA | NA | NA |
| OTU1454 | NA | NA | + | NA | NA | NA |
| OTU630 | NA | NA | + | NA | NA | NA |
| OTU5253 | NA | NA | NA | NA | + | NA |
| OTU5151 | NA | NA | NA | + | NA | NA |
| OTU5258 | NA | NA | NA | NA | + | NA |
| OTU621 | NA | NA | + | NA | NA | NA |
| OTU1625 | NA | + | NA | NA | NA | NA |
| OTU6861 | + | NA | NA | NA | NA | NA |
| OTU2592 | NA | NA | + | NA | NA | NA |
| OTU5013 | + | NA | NA | NA | NA | NA |
| OTU1991 | NA | NA | NA | NA | + | NA |
| OTU3455 | NA | NA | NA | + | NA | NA |
| OTU7635 | NA | NA | NA | NA | NA | + |
| OTU599 | NA | NA | NA | NA | NA | NA |
| OTU5673 | NA | + | NA | NA | NA | NA |
| OTU1702 | NA | NA | NA | + | NA | NA |
| OTU4749 | NA | NA | NA | NA | + | NA |
| OTU2645 | NA | NA | + | NA | + | NA |
| OTU1709 | NA | NA | + | NA | NA | NA |
| OTU235 | + | NA | NA | NA | NA | NA |
| OTU4286 | NA | NA | NA | NA | NA | + |
| OTU4284 | NA | NA | NA | + | NA | NA |
| OTU4288 | NA | NA | NA | + | NA | NA |
| OTU4289 | NA | NA | NA | + | NA | NA |
| OTU6381 | NA | NA | NA | NA | NA | + |
| OTU755 | NA | NA | + | NA | NA | NA |
| OTU696 | NA | NA | + | NA | NA | NA |
| OTU694 | NA | NA | + | NA | NA | NA |
| OTU695 | NA | NA | + | NA | NA | NA |
| OTU690 | NA | NA | + | NA | NA | NA |
| OTU691 | NA | NA | + | NA | NA | NA |
| OTU1879 | NA | NA | + | NA | NA | NA |
| OTU6958 | NA | NA | NA | NA | NA | NA |
| OTU4062 | NA | NA | NA | NA | + | NA |
| OTU6139 | NA | NA | NA | NA | NA | + |
| OTU8155 | NA | NA | + | NA | NA | NA |
| OTU7083 | NA | NA | + | NA | NA | NA |
| OTU7082 | NA | NA | + | NA | NA | NA |
| OTU3706 | NA | NA | NA | + | NA | NA |
| OTU4428 | + | NA | NA | NA | NA | NA |
| OTU7086 | NA | NA | + | NA | NA | + |
| OTU8136 | NA | NA | NA | NA | + | NA |
| OTU5025 | NA | NA | NA | NA | NA | NA |
| OTU1491 | NA | NA | + | NA | NA | NA |
| OTU8187 | NA | NA | + | NA | NA | NA |
| OTU2012 | NA | NA | + | NA | NA | NA |
| OTU3949 | + | NA | NA | NA | NA | NA |
| OTU6705 | NA | NA | NA | NA | NA | + |
| OTU5001 | NA | NA | NA | NA | NA | NA |
| OTU5508 | NA | NA | NA | NA | + | NA |
| OTU3713 | NA | + | NA | + | NA | NA |
| OTU777 | NA | NA | + | NA | NA | NA |
| OTU3325 | NA | + | NA | NA | NA | NA |
| OTU3537 | NA | NA | NA | NA | NA | NA |
| OTU7626 | NA | NA | NA | NA | NA | NA |
| OTU5878 | NA | NA | NA | NA | NA | + |
| OTU3445 | NA | NA | NA | NA | NA | + |
| OTU5977 | NA | + | NA | NA | NA | NA |
| OTU1075 | NA | NA | NA | + | NA | NA |
| OTU7451 | NA | NA | NA | + | NA | NA |
| OTU7503 | + | NA | NA | NA | NA | NA |
| OTU610 | NA | NA | + | NA | NA | NA |
| OTU1518 | + | NA | NA | NA | NA | NA |
| OTU1041 | NA | + | NA | NA | NA | NA |
| OTU4411 | + | NA | NA | NA | NA | NA |
| OTU5476 | + | NA | NA | NA | NA | NA |
| OTU928 | NA | NA | NA | + | NA | NA |
| OTU2223 | NA | NA | NA | + | NA | NA |
| OTU625 | NA | NA | + | NA | NA | NA |
| OTU539 | NA | NA | + | NA | NA | NA |
| OTU7265 | NA | NA | + | NA | NA | NA |
| OTU522 | NA | NA | + | NA | NA | NA |
| OTU3797 | NA | NA | NA | NA | NA | NA |
| OTU529 | NA | NA | + | NA | NA | NA |
| OTU3471 | NA | NA | NA | + | NA | NA |
| OTU4974 | + | NA | NA | NA | NA | NA |
| OTU4699 | NA | NA | NA | + | NA | NA |
| OTU5921 | NA | NA | NA | NA | NA | + |
| OTU839 | NA | NA | NA | NA | + | NA |
| OTU6461 | NA | NA | NA | NA | NA | + |
| OTU620 | NA | NA | + | NA | NA | NA |
| OTU7574 | NA | NA | NA | NA | + | NA |
| OTU7078 | NA | NA | + | NA | NA | NA |
| OTU1694 | NA | NA | + | NA | NA | NA |
| OTU3486 | NA | NA | NA | + | NA | NA |
| OTU4001 | + | NA | NA | NA | NA | NA |
| OTU4610 | NA | + | NA | NA | NA | NA |
| OTU721 | NA | NA | NA | NA | NA | NA |
| OTU2998 | NA | NA | NA | NA | NA | NA |
| OTU8205 | NA | NA | NA | NA | NA | NA |
| OTU2463 | + | NA | NA | NA | NA | NA |
| OTU2464 | NA | NA | + | NA | NA | NA |
| OTU7199 | NA | NA | NA | NA | NA | + |
| OTU6737 | NA | NA | NA | + | NA | NA |
| OTU4397 | NA | + | NA | NA | NA | NA |
| OTU3733 | NA | + | NA | NA | NA | NA |
| OTU6414 | NA | NA | NA | NA | NA | + |
| OTU2874 | NA | NA | NA | NA | NA | + |
| OTU2707 | NA | NA | NA | NA | NA | NA |
| OTU3594 | NA | NA | + | NA | NA | NA |
| OTU7653 | NA | NA | + | NA | NA | NA |
| OTU4224 | NA | + | NA | NA | NA | NA |
| OTU3912 | + | NA | NA | NA | NA | NA |
| OTU5494 | + | NA | NA | NA | NA | NA |
| OTU4897 | + | NA | NA | NA | NA | NA |
| OTU3556 | NA | NA | + | NA | NA | NA |
| OTU4477 | + | NA | NA | NA | NA | NA |
| OTU82 | NA | NA | NA | NA | NA | NA |
| OTU1051 | NA | NA | NA | NA | NA | NA |
| OTU6985 | NA | NA | + | NA | NA | NA |
| OTU4283 | NA | NA | NA | + | NA | NA |
| OTU993 | NA | NA | + | NA | NA | NA |
| OTU4884 | + | NA | NA | NA | NA | NA |
| OTU773 | NA | NA | NA | NA | NA | NA |
| OTU4840 | + | NA | NA | NA | NA | NA |
| OTU8037 | NA | NA | NA | NA | NA | NA |
| OTU7418 | NA | NA | NA | NA | + | NA |
| OTU647 | NA | NA | NA | NA | + | NA |
| OTU4027 | NA | NA | NA | NA | NA | NA |
| OTU2838 | NA | NA | NA | NA | + | NA |
| OTU3053 | NA | NA | NA | NA | NA | NA |
| OTU2542 | NA | NA | NA | + | NA | NA |
| OTU5740 | NA | + | NA | NA | NA | NA |
| OTU4687 | NA | NA | NA | NA | NA | NA |
| OTU470 | NA | NA | + | NA | NA | NA |
| OTU5034 | + | NA | NA | NA | NA | NA |
| OTU474 | NA | NA | + | NA | NA | NA |
| OTU2895 | NA | NA | NA | + | NA | NA |
| OTU183 | NA | NA | NA | + | NA | NA |
| OTU4483 | + | NA | NA | NA | NA | NA |
| OTU6285 | NA | NA | NA | NA | + | NA |
| OTU1206 | NA | NA | + | NA | NA | NA |
| OTU5313 | NA | NA | NA | NA | NA | + |
| OTU2769 | + | NA | NA | NA | NA | NA |
| OTU2767 | NA | NA | NA | + | NA | NA |
| OTU2020 | NA | NA | NA | + | NA | NA |
| OTU6070 | NA | NA | NA | NA | NA | + |
| OTU5769 | NA | NA | + | NA | NA | NA |
| OTU2755 | NA | NA | NA | NA | + | NA |
| OTU4751 | NA | NA | NA | NA | + | NA |
| OTU7886 | NA | NA | NA | NA | NA | + |
| OTU8040 | + | NA | NA | NA | NA | NA |
| OTU8068 | NA | NA | NA | NA | NA | + |
| OTU1514 | NA | + | NA | NA | NA | NA |
| OTU5012 | + | NA | NA | NA | NA | NA |
| OTU2 | NA | NA | NA | NA | NA | NA |
| OTU5270 | NA | NA | NA | NA | + | NA |
| OTU937 | NA | NA | + | NA | NA | NA |
| OTU2505 | NA | + | NA | NA | NA | NA |
| OTU5475 | NA | NA | NA | NA | NA | NA |
| OTU4306 | NA | NA | NA | + | NA | NA |
| OTU4603 | NA | NA | NA | NA | NA | NA |
| OTU4600 | NA | + | NA | NA | NA | NA |
| OTU2538 | + | NA | NA | NA | NA | NA |
| OTU3411 | NA | NA | NA | NA | NA | NA |
| OTU5448 | + | NA | NA | NA | NA | NA |
| OTU6217 | NA | NA | NA | NA | NA | NA |
| OTU7344 | + | NA | NA | NA | NA | + |
| OTU64 | NA | NA | NA | NA | NA | + |
| OTU7069 | NA | NA | + | NA | NA | NA |
| OTU7068 | NA | NA | + | NA | NA | NA |
| OTU4613 | NA | + | NA | NA | NA | NA |
| OTU7062 | NA | NA | + | NA | NA | NA |
| OTU7061 | NA | NA | + | NA | NA | NA |
| OTU7060 | NA | NA | + | NA | NA | NA |
| OTU7067 | NA | NA | + | NA | NA | NA |
| OTU7064 | NA | NA | + | NA | NA | NA |
| OTU1920 | NA | NA | + | NA | NA | NA |
| OTU7143 | NA | + | NA | NA | NA | NA |
| OTU5683 | NA | NA | NA | NA | + | NA |
| OTU4757 | NA | NA | NA | NA | + | NA |
| OTU7007 | NA | NA | NA | NA | + | NA |
| OTU396 | NA | NA | + | NA | NA | NA |
| OTU5351 | + | NA | NA | NA | NA | NA |
| OTU4094 | NA | NA | NA | NA | NA | NA |
| OTU453 | NA | NA | NA | NA | NA | + |
| OTU1525 | NA | NA | + | NA | NA | NA |
| OTU1035 | NA | NA | + | NA | NA | NA |
| OTU6700 | NA | NA | + | NA | NA | NA |
| OTU7714 | NA | + | NA | + | NA | NA |
| OTU6 | NA | NA | + | NA | NA | NA |
| OTU1044 | NA | + | NA | NA | NA | NA |
| OTU7096 | NA | NA | + | NA | NA | NA |
| OTU1040 | NA | + | NA | NA | NA | NA |
| OTU2335 | NA | NA | + | NA | NA | NA |
| OTU3073 | NA | NA | NA | + | NA | NA |
| OTU6731 | + | NA | NA | NA | NA | NA |
| OTU3489 | NA | NA | NA | + | NA | NA |
| OTU335 | NA | NA | + | NA | NA | NA |
| OTU4775 | NA | NA | NA | NA | + | NA |
| OTU542 | NA | NA | NA | NA | NA | NA |
| OTU4480 | + | NA | NA | NA | NA | NA |
| OTU2175 | NA | NA | + | NA | NA | NA |
| OTU5479 | + | NA | NA | NA | NA | NA |
| OTU678 | NA | NA | + | NA | NA | NA |
| OTU5457 | + | NA | NA | NA | NA | NA |
| OTU2423 | NA | NA | + | NA | NA | NA |
| OTU5663 | + | NA | NA | NA | NA | NA |
| OTU930 | NA | + | NA | NA | + | NA |
| OTU7277 | NA | NA | NA | + | NA | NA |
| OTU5489 | + | NA | NA | NA | NA | NA |
| OTU1183 | NA | NA | + | NA | NA | NA |
| OTU1772 | NA | NA | + | NA | NA | NA |
| OTU3233 | + | NA | NA | NA | NA | NA |
| OTU632 | NA | + | NA | NA | NA | NA |
| OTU2955 | NA | NA | + | NA | NA | NA |
| OTU2950 | NA | NA | + | NA | NA | NA |
| OTU2953 | NA | NA | + | NA | NA | NA |
| OTU4695 | NA | NA | NA | + | NA | NA |
| OTU5600 | NA | NA | NA | NA | + | NA |
| OTU4876 | NA | NA | NA | NA | NA | NA |
| OTU5602 | NA | NA | NA | NA | + | NA |
| OTU1986 | + | NA | NA | NA | NA | NA |
| OTU1985 | NA | NA | NA | + | NA | NA |
| OTU2306 | NA | NA | NA | NA | NA | + |
| OTU2931 | NA | NA | NA | NA | NA | NA |
| OTU3948 | NA | NA | NA | NA | NA | NA |
| OTU3013 | + | NA | NA | NA | NA | NA |
| OTU1723 | NA | NA | NA | + | NA | NA |
| OTU1720 | NA | NA | NA | NA | + | NA |
| OTU2983 | NA | NA | + | NA | NA | NA |
| OTU4269 | NA | NA | NA | + | NA | NA |
| OTU4262 | NA | NA | NA | + | NA | NA |
| OTU1162 | + | NA | NA | NA | NA | NA |
| OTU4265 | NA | NA | NA | + | NA | NA |
| OTU4714 | NA | NA | NA | NA | NA | NA |
| OTU4023 | NA | NA | NA | NA | NA | NA |
| OTU4908 | + | NA | NA | NA | NA | NA |
| OTU4298 | NA | NA | NA | NA | NA | NA |
| OTU7307 | NA | + | NA | NA | NA | NA |
| OTU4006 | NA | NA | NA | + | NA | NA |
| OTU7449 | NA | NA | NA | + | NA | NA |
| OTU4003 | NA | NA | + | NA | NA | NA |
| OTU4899 | NA | NA | NA | NA | NA | NA |
| OTU5759 | NA | NA | NA | NA | NA | + |
| OTU5751 | + | NA | NA | NA | NA | NA |
| OTU2668 | NA | NA | NA | NA | + | NA |
| OTU6949 | NA | NA | NA | NA | + | NA |
| OTU3037 | NA | NA | + | NA | NA | NA |
| OTU2212 | NA | NA | + | NA | NA | NA |
| OTU4197 | NA | + | NA | NA | NA | NA |
| OTU7586 | NA | NA | NA | NA | NA | NA |
| OTU1476 | NA | NA | NA | NA | + | NA |
| OTU4013 | NA | NA | NA | NA | NA | NA |
| OTU7472 | NA | NA | NA | NA | NA | + |
| OTU7473 | NA | NA | NA | NA | NA | NA |
| OTU6935 | NA | NA | NA | NA | NA | + |
| OTU7487 | NA | NA | NA | NA | + | NA |
| OTU7003 | NA | NA | NA | NA | NA | + |
| OTU2780 | + | NA | NA | NA | NA | NA |
| OTU7839 | + | NA | NA | NA | NA | NA |
| OTU1512 | NA | NA | NA | NA | NA | NA |
| OTU4828 | + | NA | NA | NA | NA | NA |
| OTU1209 | NA | NA | + | NA | NA | NA |
| OTU6638 | NA | NA | NA | NA | NA | + |
| OTU852 | NA | NA | NA | + | NA | NA |
| OTU4580 | NA | NA | NA | NA | + | NA |
| OTU1071 | NA | NA | NA | NA | NA | + |
| OTU8120 | NA | NA | NA | NA | NA | NA |
| OTU4928 | NA | NA | NA | NA | NA | NA |
| OTU1698 | NA | NA | NA | + | NA | NA |
| OTU2379 | NA | NA | NA | NA | + | NA |
| OTU595 | NA | NA | + | NA | NA | NA |
| OTU5850 | NA | NA | NA | NA | NA | + |
| OTU7033 | NA | NA | + | NA | NA | NA |
| OTU1431 | NA | NA | NA | NA | NA | NA |
| OTU3362 | NA | NA | + | + | NA | NA |
| OTU4453 | NA | NA | NA | NA | NA | NA |
| OTU5081 | NA | NA | NA | NA | NA | + |
| OTU6508 | NA | NA | NA | + | NA | NA |
| OTU4274 | NA | NA | NA | + | NA | NA |
| OTU287 | + | NA | NA | + | NA | NA |
| OTU7042 | NA | NA | + | NA | NA | NA |
| OTU6957 | NA | NA | NA | NA | NA | + |
| OTU1062 | NA | NA | + | NA | NA | NA |
| OTU4201 | NA | + | NA | NA | NA | NA |
| OTU2903 | NA | + | NA | NA | NA | NA |
| OTU4085 | + | NA | NA | NA | NA | NA |
| OTU6711 | NA | NA | NA | NA | NA | + |
| OTU6077 | NA | NA | + | NA | NA | NA |
| OTU7289 | + | NA | NA | NA | NA | NA |
| OTU7107 | NA | NA | + | NA | NA | NA |
| OTU4401 | + | NA | NA | NA | NA | NA |
| OTU5653 | NA | NA | + | NA | NA | NA |
| OTU2833 | NA | + | NA | NA | NA | NA |
| OTU5039 | NA | NA | NA | NA | + | NA |
| OTU6840 | NA | NA | + | NA | NA | NA |
| OTU5682 | NA | NA | NA | + | NA | NA |
| OTU5210 | + | NA | NA | NA | NA | NA |
| OTU4371 | NA | + | NA | NA | NA | NA |
| OTU7099 | NA | NA | + | NA | NA | NA |
| OTU3317 | NA | NA | NA | NA | + | NA |
| OTU4920 | NA | NA | NA | NA | + | NA |
| OTU4925 | + | NA | NA | NA | NA | NA |
| OTU4924 | + | NA | NA | NA | NA | NA |
| OTU3842 | NA | NA | + | NA | NA | NA |
| OTU1782 | NA | NA | + | NA | NA | NA |
| OTU8058 | NA | NA | NA | + | NA | NA |
| OTU3861 | NA | NA | + | NA | NA | NA |
| OTU3930 | + | NA | NA | NA | NA | NA |
| OTU7294 | NA | NA | NA | + | NA | NA |
| OTU4184 | NA | + | NA | NA | NA | NA |
| OTU2743 | NA | NA | NA | NA | NA | + |
| OTU2746 | NA | NA | NA | + | NA | NA |
| OTU4189 | NA | + | NA | NA | NA | NA |
| OTU4839 | + | NA | NA | NA | NA | NA |
| OTU5586 | + | NA | NA | NA | NA | NA |
| OTU8213 | NA | NA | NA | + | NA | NA |
| OTU4988 | NA | NA | NA | NA | + | NA |
| OTU6719 | NA | NA | NA | + | NA | NA |
| OTU1820 | + | NA | NA | NA | NA | NA |
| OTU1812 | + | NA | NA | NA | NA | NA |
| OTU2256 | + | NA | NA | NA | NA | NA |
| OTU28 | NA | + | NA | NA | NA | NA |
| OTU2777 | NA | NA | NA | NA | + | NA |
| OTU790 | + | NA | + | NA | NA | NA |
| OTU4496 | NA | NA | NA | NA | + | NA |
| OTU5998 | NA | NA | NA | NA | NA | + |
| OTU1541 | NA | NA | NA | + | NA | NA |
| OTU5059 | + | NA | NA | NA | NA | NA |
| OTU5127 | NA | NA | NA | NA | NA | NA |
| OTU4747 | NA | NA | NA | NA | + | NA |
| OTU4746 | NA | NA | NA | NA | + | NA |
| OTU4740 | NA | + | NA | NA | NA | NA |
| OTU2569 | + | NA | NA | NA | NA | NA |
| OTU2568 | + | NA | NA | NA | NA | NA |
| OTU7093 | NA | NA | + | NA | NA | NA |
| OTU2229 | NA | NA | + | NA | NA | NA |
| OTU4918 | NA | NA | NA | NA | NA | NA |
| OTU4737 | + | NA | NA | NA | NA | NA |
| OTU5841 | NA | NA | NA | NA | NA | + |
| OTU6051 | NA | NA | NA | + | NA | NA |
| OTU4827 | NA | NA | NA | + | NA | NA |
| OTU5983 | NA | NA | NA | NA | NA | + |
| OTU7957 | NA | NA | NA | NA | NA | + |
| OTU2818 | + | NA | NA | NA | NA | NA |
| OTU2234 | + | NA | NA | NA | NA | NA |
| OTU5442 | NA | NA | NA | + | NA | NA |
| OTU7624 | NA | NA | NA | NA | NA | + |
| OTU5706 | + | NA | NA | NA | NA | NA |
| OTU327 | NA | NA | NA | + | NA | NA |
| OTU1711 | NA | NA | NA | NA | NA | + |
| OTU4242 | NA | + | NA | NA | NA | NA |
| OTU4248 | NA | NA | NA | + | NA | NA |
| OTU8178 | NA | NA | NA | NA | NA | NA |
| OTU4052 | NA | NA | NA | + | NA | NA |
| OTU5999 | NA | NA | + | NA | NA | NA |
| OTU5993 | NA | NA | NA | NA | NA | + |
| OTU6756 | NA | NA | NA | NA | NA | NA |
| OTU655 | NA | NA | NA | NA | NA | NA |
| OTU4701 | + | NA | NA | NA | NA | NA |
| OTU39 | NA | NA | NA | NA | NA | + |
| OTU4803 | NA | + | NA | NA | + | NA |
| OTU4307 | NA | NA | NA | + | NA | NA |
| OTU4009 | NA | NA | NA | NA | NA | + |
| OTU2215 | NA | NA | NA | + | NA | NA |
| OTU3369 | NA | NA | NA | + | NA | NA |
| OTU3661 | + | NA | NA | NA | NA | NA |
| OTU2266 | NA | NA | NA | + | NA | NA |
| OTU3039 | NA | NA | + | NA | NA | + |
| OTU7049 | NA | NA | + | NA | NA | NA |
| OTU3032 | NA | NA | + | NA | NA | NA |
| OTU6064 | NA | NA | + | NA | NA | NA |
| OTU5244 | NA | NA | NA | NA | + | NA |
| OTU6965 | + | NA | NA | NA | NA | NA |
| OTU4807 | NA | NA | NA | NA | + | NA |
| OTU3390 | NA | NA | NA | + | NA | NA |
| OTU172 | NA | + | NA | NA | NA | NA |
| OTU7952 | NA | NA | NA | NA | NA | + |
| OTU5204 | NA | NA | NA | NA | + | NA |
| OTU2912 | NA | NA | NA | NA | NA | NA |
| OTU4545 | NA | NA | NA | NA | + | NA |
| OTU2279 | NA | NA | NA | NA | + | NA |
| OTU2273 | NA | NA | NA | NA | + | NA |
| OTU5913 | NA | NA | NA | NA | NA | + |
| OTU5915 | NA | NA | NA | NA | NA | + |
| OTU2222 | + | NA | NA | NA | NA | NA |
| OTU5919 | NA | NA | NA | NA | NA | + |
| OTU6567 | + | NA | NA | NA | NA | NA |
| OTU6812 | NA | NA | NA | NA | NA | NA |
| OTU2881 | NA | NA | NA | + | NA | NA |
| OTU3465 | NA | NA | NA | + | NA | NA |
| OTU4022 | NA | NA | NA | + | NA | NA |
| OTU5337 | NA | NA | + | NA | NA | NA |
| OTU7877 | NA | NA | + | NA | NA | NA |
| OTU5827 | NA | NA | NA | NA | NA | NA |
| OTU5231 | NA | NA | NA | + | NA | NA |
| OTU5195 | + | NA | NA | NA | NA | NA |
| OTU5199 | NA | NA | NA | NA | NA | NA |
| OTU7044 | NA | NA | + | NA | NA | NA |
| OTU7515 | + | NA | NA | NA | NA | NA |
| OTU7882 | NA | NA | NA | NA | NA | NA |
| OTU1124 | NA | NA | + | NA | NA | NA |
| OTU3768 | + | NA | NA | NA | NA | NA |
| OTU1303 | NA | + | NA | NA | NA | NA |
| OTU5371 | + | NA | NA | + | NA | NA |
| OTU5899 | NA | NA | NA | + | NA | NA |
| OTU133 | NA | NA | NA | + | NA | NA |
| OTU6504 | NA | NA | NA | + | NA | NA |
| OTU4424 | + | NA | NA | NA | NA | NA |
| OTU1368 | NA | NA | NA | NA | + | NA |
| OTU3483 | NA | NA | NA | + | NA | NA |
| OTU2959 | NA | NA | + | NA | NA | NA |
| OTU3488 | NA | NA | NA | + | NA | NA |
| OTU4260 | NA | NA | NA | + | NA | NA |
| OTU4799 | NA | NA | NA | NA | + | NA |
| OTU4461 | + | NA | NA | NA | NA | NA |
| OTU5525 | NA | NA | NA | NA | + | NA |
| OTU4903 | NA | NA | NA | + | NA | NA |
| OTU4902 | + | NA | NA | NA | NA | NA |
| OTU5757 | NA | NA | NA | NA | + | NA |
| OTU3261 | NA | NA | + | NA | NA | NA |
| OTU3260 | NA | NA | NA | NA | NA | NA |
| OTU5777 | NA | NA | + | NA | NA | NA |
| OTU2748 | NA | NA | NA | + | NA | NA |
| OTU4642 | NA | NA | NA | + | NA | NA |
| OTU3307 | NA | NA | NA | + | NA | NA |
| OTU8113 | NA | + | NA | NA | NA | NA |
| OTU2186 | NA | NA | NA | NA | + | NA |
| OTU8100 | + | NA | NA | NA | NA | NA |
| OTU2054 | NA | NA | NA | + | NA | NA |
| OTU3634 | NA | NA | NA | NA | + | NA |
| OTU5909 | NA | NA | NA | NA | NA | + |
| OTU1839 | NA | NA | NA | + | NA | NA |
| OTU1432 | NA | NA | NA | NA | NA | NA |
| OTU4761 | NA | NA | NA | NA | + | NA |
| OTU6478 | NA | + | NA | NA | NA | NA |
| OTU5193 | NA | NA | NA | NA | + | NA |
| OTU5419 | NA | NA | + | NA | NA | NA |
| OTU7053 | NA | NA | + | NA | NA | NA |
| OTU3441 | NA | NA | NA | + | NA | NA |
| OTU1257 | NA | NA | + | NA | NA | NA |
| OTU1389 | NA | NA | NA | + | NA | NA |
| OTU3308 | NA | NA | NA | + | NA | NA |
| OTU4844 | + | NA | NA | NA | NA | NA |
| OTU4770 | NA | NA | NA | NA | + | NA |
| OTU4754 | NA | NA | NA | NA | NA | NA |
| OTU2301 | NA | NA | NA | NA | NA | NA |
| OTU460 | NA | NA | + | NA | NA | NA |
| OTU430 | NA | NA | + | NA | NA | NA |
| OTU467 | NA | + | NA | NA | NA | NA |
| OTU468 | NA | NA | + | NA | NA | NA |
| OTU6610 | NA | NA | NA | + | NA | NA |
| OTU4763 | NA | NA | NA | NA | + | NA |
| OTU3059 | NA | NA | NA | NA | NA | NA |
| OTU4765 | NA | NA | NA | NA | + | NA |
| OTU3065 | NA | NA | NA | + | NA | NA |
| OTU4769 | NA | NA | NA | NA | NA | + |
| OTU1241 | NA | NA | NA | NA | NA | + |
| OTU629 | NA | NA | + | NA | NA | NA |
| OTU7057 | NA | NA | + | NA | NA | NA |
| OTU5516 | NA | NA | NA | + | NA | NA |
| OTU2942 | NA | NA | NA | NA | NA | NA |
| OTU673 | NA | NA | + | NA | NA | NA |
| OTU662 | NA | NA | + | NA | NA | NA |
| OTU197 | NA | NA | NA | NA | NA | + |
| OTU7174 | NA | NA | + | NA | NA | NA |
| OTU1408 | NA | NA | + | NA | NA | NA |
| OTU3320 | NA | NA | NA | + | NA | NA |
| OTU1878 | NA | + | NA | NA | NA | NA |
| OTU5312 | NA | NA | NA | NA | NA | NA |
| OTU1962 | NA | NA | + | NA | NA | NA |
| OTU1348 | NA | NA | NA | NA | NA | NA |
| OTU6686 | NA | NA | NA | NA | NA | NA |
| OTU1827 | NA | NA | NA | NA | NA | NA |
| OTU4781 | NA | NA | NA | NA | + | NA |
| OTU2908 | NA | NA | NA | + | NA | NA |
| OTU1268 | NA | NA | NA | NA | NA | NA |
| OTU6348 | + | NA | NA | NA | NA | NA |
| OTU8181 | NA | NA | + | NA | NA | NA |
| OTU1337 | NA | + | NA | + | NA | NA |
| OTU3601 | NA | NA | NA | NA | NA | + |
| OTU6194 | NA | NA | NA | NA | NA | + |
| OTU6197 | NA | NA | NA | NA | NA | + |
| OTU6190 | NA | NA | NA | NA | NA | + |
| OTU574 | NA | NA | + | NA | NA | NA |
| OTU8169 | + | NA | NA | NA | NA | NA |
| OTU5738 | NA | + | NA | NA | NA | NA |
| OTU7802 | NA | NA | + | NA | NA | NA |
| OTU7027 | NA | NA | + | NA | NA | NA |
| OTU429 | NA | NA | + | NA | NA | NA |
| OTU3010 | NA | NA | + | NA | NA | NA |
| OTU7024 | NA | NA | + | NA | NA | NA |
| OTU3016 | NA | NA | + | NA | NA | NA |
| OTU537 | NA | NA | + | NA | NA | NA |
| OTU4005 | NA | NA | NA | NA | NA | NA |
| OTU4526 | + | NA | NA | NA | NA | NA |
| OTU7960 | NA | NA | NA | NA | + | NA |
| OTU5103 | NA | NA | NA | NA | NA | + |
| OTU7021 | NA | NA | + | NA | NA | NA |
| OTU5412 | + | NA | NA | NA | NA | NA |
| OTU5256 | NA | + | NA | NA | + | NA |
| OTU7950 | + | NA | NA | NA | NA | NA |
| OTU5832 | NA | NA | NA | + | NA | NA |
| OTU1272 | NA | + | NA | NA | NA | NA |
| OTU4325 | NA | NA | NA | + | NA | NA |
| OTU4947 | NA | NA | NA | + | NA | NA |
| OTU4753 | NA | NA | NA | NA | + | NA |
| OTU5176 | NA | NA | NA | NA | + | NA |
| OTU3047 | NA | NA | NA | + | NA | NA |
| OTU2635 | NA | NA | NA | NA | NA | NA |
| OTU2876 | + | NA | NA | NA | NA | NA |
| OTU6262 | NA | NA | + | NA | NA | NA |
| OTU5095 | NA | + | NA | NA | NA | NA |
| OTU3703 | NA | NA | NA | NA | NA | NA |
| OTU4961 | NA | NA | + | NA | NA | NA |
| OTU2627 | NA | NA | NA | NA | + | NA |
| OTU5002 | NA | NA | + | NA | NA | NA |
| OTU4631 | NA | + | NA | NA | NA | NA |
| OTU6886 | NA | NA | NA | NA | NA | + |
| OTU4604 | NA | NA | + | NA | NA | NA |
| OTU4109 | NA | NA | + | NA | NA | NA |
| OTU6592 | NA | NA | + | NA | NA | NA |
| OTU8174 | NA | NA | + | NA | NA | NA |
| OTU1610 | NA | NA | NA | NA | NA | NA |
| OTU7022 | NA | NA | + | NA | NA | NA |
| OTU7505 | NA | NA | NA | NA | NA | NA |
| OTU688 | NA | NA | + | NA | NA | NA |
| OTU612 | NA | NA | NA | NA | NA | NA |
| OTU2077 | NA | NA | NA | + | NA | NA |
| OTU3005 | NA | NA | + | NA | NA | NA |
| OTU1411 | NA | NA | NA | + | NA | NA |
| OTU880 | NA | NA | + | NA | NA | NA |
| OTU1321 | + | NA | NA | NA | NA | NA |
| OTU4019 | NA | NA | NA | NA | NA | NA |
| OTU1987 | NA | NA | + | NA | NA | NA |
| OTU2807 | NA | NA | + | NA | NA | NA |
| OTU8254 | NA | NA | NA | NA | NA | + |
| OTU2831 | NA | NA | NA | NA | + | NA |
| OTU4564 | NA | NA | NA | NA | + | NA |
| OTU7031 | NA | NA | + | NA | NA | NA |
| OTU4220 | NA | + | NA | NA | NA | NA |
| OTU4221 | NA | + | NA | NA | NA | NA |
| OTU4222 | NA | + | NA | NA | NA | NA |
| OTU3656 | + | NA | NA | NA | NA | NA |
| OTU4228 | NA | + | NA | NA | NA | NA |
| OTU417 | NA | + | + | NA | NA | NA |
| OTU675 | NA | NA | + | NA | NA | NA |
| OTU1509 | + | NA | NA | NA | NA | NA |
| OTU60 | NA | NA | + | NA | NA | NA |
| OTU5797 | NA | NA | NA | NA | NA | + |
| OTU5214 | NA | NA | NA | NA | + | NA |
| OTU5159 | NA | NA | NA | NA | + | NA |
| OTU123 | NA | NA | NA | NA | NA | NA |
| OTU5236 | NA | NA | NA | NA | + | NA |
| OTU910 | NA | NA | + | NA | NA | NA |
| OTU2319 | + | NA | NA | NA | NA | NA |
| OTU2030 | NA | NA | + | NA | NA | NA |
| OTU797 | NA | NA | NA | NA | + | NA |
| OTU4036 | NA | NA | NA | NA | + | NA |
| OTU8270 | NA | NA | NA | + | NA | NA |
| OTU2582 | NA | NA | NA | NA | + | NA |
| OTU6779 | NA | NA | NA | NA | NA | NA |
| OTU2972 | NA | + | NA | NA | NA | NA |
| OTU4181 | NA | NA | NA | NA | NA | NA |
| OTU6394 | NA | NA | NA | + | NA | NA |
| OTU3878 | + | NA | NA | NA | NA | NA |
| OTU890 | NA | + | NA | NA | NA | NA |
| OTU7815 | NA | NA | NA | NA | NA | + |
| OTU2332 | NA | NA | + | NA | NA | NA |
| OTU7923 | NA | + | NA | NA | NA | NA |
| OTU5897 | NA | NA | NA | NA | NA | + |
| OTU5335 | NA | NA | NA | NA | NA | + |
| OTU7045 | NA | + | NA | NA | NA | NA |
| OTU3619 | NA | NA | NA | NA | NA | NA |
| OTU7163 | NA | NA | NA | NA | NA | NA |
| OTU4987 | NA | + | NA | NA | NA | NA |
| OTU1258 | NA | NA | NA | NA | NA | NA |
| OTU7181 | NA | NA | + | NA | NA | NA |
| OTU5608 | NA | NA | NA | NA | + | NA |
| OTU1511 | NA | NA | NA | NA | NA | NA |
| OTU4875 | NA | NA | NA | NA | NA | NA |
| OTU3323 | NA | + | NA | NA | NA | NA |
| OTU271 | NA | NA | NA | + | NA | NA |
| OTU4745 | NA | NA | NA | NA | + | NA |
| OTU5197 | + | NA | NA | NA | NA | NA |
| OTU879 | NA | NA | NA | NA | NA | + |
| OTU4493 | + | NA | NA | NA | NA | NA |
| OTU3002 | NA | NA | + | NA | NA | NA |
| OTU6628 | NA | NA | NA | NA | NA | NA |
| OTU6525 | NA | NA | + | NA | NA | NA |
| OTU5406 | NA | + | NA | NA | NA | NA |
| OTU5073 | + | + | NA | + | NA | NA |
| OTU4449 | NA | NA | NA | NA | NA | NA |
| OTU4448 | + | NA | NA | NA | NA | NA |
| OTU4442 | + | NA | NA | NA | NA | NA |
| OTU3792 | NA | NA | NA | NA | NA | + |
| OTU5456 | + | NA | NA | NA | NA | NA |
| OTU3159 | NA | NA | NA | NA | NA | + |
| OTU3720 | NA | NA | NA | NA | + | NA |
| OTU268 | NA | + | NA | NA | NA | NA |
| OTU3023 | NA | NA | + | NA | NA | NA |
| OTU4565 | NA | NA | NA | NA | + | NA |
| OTU4111 | + | NA | NA | NA | NA | NA |
| OTU4529 | + | NA | NA | NA | NA | NA |
| OTU7853 | NA | NA | NA | + | NA | NA |
| OTU4647 | NA | NA | + | NA | NA | NA |
| OTU4960 | NA | NA | NA | NA | NA | NA |
| OTU709 | NA | NA | NA | + | NA | NA |
| OTU5538 | NA | + | NA | NA | NA | NA |
| OTU1787 | NA | NA | + | NA | NA | NA |
| OTU3200 | NA | NA | + | NA | NA | NA |
| OTU5512 | NA | NA | NA | NA | NA | + |
| OTU3105 | NA | NA | + | + | NA | NA |
| OTU5097 | NA | + | NA | NA | NA | NA |
| OTU4134 | + | NA | NA | NA | NA | NA |
| OTU3293 | + | NA | NA | NA | NA | NA |
| OTU5156 | NA | NA | NA | NA | + | NA |
| OTU1699 | NA | NA | NA | NA | NA | NA |
| OTU5234 | NA | NA | NA | NA | + | NA |
| OTU2371 | NA | NA | + | NA | NA | NA |
| OTU6201 | NA | NA | NA | NA | NA | + |
| OTU3384 | NA | NA | NA | + | NA | NA |
| OTU3153 | NA | NA | NA | NA | + | NA |
| OTU1240 | NA | NA | NA | + | NA | NA |
| OTU5922 | + | NA | NA | NA | NA | NA |
| OTU5468 | + | NA | NA | NA | NA | NA |
| OTU7043 | NA | NA | NA | NA | NA | NA |
| OTU666 | NA | NA | NA | NA | NA | + |
| OTU5979 | NA | NA | NA | + | NA | NA |
| OTU7561 | NA | NA | NA | NA | NA | + |
| OTU1243 | + | NA | NA | NA | NA | NA |
| OTU4849 | + | NA | NA | NA | NA | NA |
| OTU6511 | NA | NA | NA | NA | NA | NA |
| OTU3576 | NA | NA | NA | NA | NA | NA |
| OTU1199 | + | NA | NA | NA | NA | NA |
| OTU4784 | NA | NA | NA | NA | + | NA |
| OTU2529 | NA | NA | + | NA | NA | NA |
| OTU5668 | + | NA | NA | NA | NA | NA |
| OTU3142 | + | NA | NA | NA | NA | NA |
| OTU5009 | NA | NA | NA | NA | NA | NA |
| OTU5881 | NA | NA | NA | NA | NA | + |
| OTU8263 | NA | + | NA | NA | NA | NA |
| OTU8138 | NA | NA | + | NA | NA | NA |
| OTU4219 | NA | + | NA | NA | NA | NA |
| OTU5029 | NA | NA | NA | NA | NA | NA |
| OTU657 | NA | NA | + | NA | NA | NA |
| OTU6390 | NA | NA | NA | NA | + | NA |
| OTU25 | NA | + | NA | NA | NA | NA |
| OTU117 | NA | NA | + | NA | NA | NA |
| OTU4205 | NA | + | NA | NA | NA | NA |
| OTU4202 | NA | + | NA | NA | NA | NA |
| OTU4200 | NA | + | NA | NA | NA | NA |
| OTU2370 | NA | NA | NA | NA | NA | NA |
| OTU1615 | NA | NA | + | NA | NA | NA |
| OTU7674 | NA | NA | NA | NA | NA | NA |
| OTU4776 | NA | NA | NA | NA | + | NA |
| OTU4270 | NA | NA | NA | NA | NA | NA |
| OTU617 | NA | NA | + | NA | NA | NA |
| OTU614 | NA | NA | + | NA | NA | NA |
| OTU1555 | NA | NA | NA | NA | + | NA |
| OTU613 | NA | NA | + | NA | NA | NA |
| OTU2725 | NA | NA | NA | NA | + | NA |
| OTU5237 | NA | NA | NA | NA | + | NA |
| OTU619 | NA | NA | + | NA | NA | NA |
| OTU3493 | NA | NA | NA | + | NA | NA |
| OTU519 | NA | NA | + | NA | NA | NA |
| OTU5392 | NA | + | NA | NA | NA | NA |
| OTU5633 | NA | NA | NA | NA | NA | NA |
| OTU4412 | + | NA | NA | NA | NA | NA |
| OTU3427 | NA | NA | NA | NA | NA | NA |
| OTU618 | NA | NA | + | NA | NA | NA |
| OTU4209 | NA | + | NA | NA | NA | NA |
| OTU3215 | NA | NA | + | NA | NA | NA |
| OTU4856 | + | NA | NA | NA | NA | NA |
| OTU1982 | NA | NA | NA | NA | NA | NA |
| OTU4859 | + | NA | NA | NA | NA | NA |
| OTU7527 | + | NA | NA | NA | + | NA |
| OTU2712 | + | NA | NA | NA | NA | NA |
| OTU6902 | + | NA | NA | NA | NA | NA |
| OTU3074 | NA | NA | NA | + | NA | NA |
| OTU7559 | NA | NA | NA | NA | NA | + |
| OTU7558 | NA | NA | + | NA | NA | NA |
| OTU7005 | NA | NA | NA | NA | NA | + |
| OTU436 | NA | NA | + | NA | NA | NA |
| OTU4103 | + | NA | NA | NA | NA | NA |
| OTU4368 | + | NA | NA | NA | NA | NA |
| OTU5332 | NA | NA | NA | NA | NA | NA |
| OTU6255 | NA | NA | + | NA | NA | NA |
| OTU5846 | + | NA | NA | NA | NA | NA |
| OTU5845 | NA | NA | NA | NA | NA | + |
| OTU4972 | NA | NA | NA | NA | + | NA |
| OTU4772 | + | NA | NA | NA | + | NA |
| OTU7530 | NA | NA | NA | NA | NA | + |
| OTU2951 | NA | NA | + | NA | NA | NA |
| OTU4133 | NA | NA | + | NA | NA | + |
| OTU5840 | NA | NA | NA | NA | NA | NA |
| OTU2235 | + | NA | NA | NA | NA | NA |
| OTU6753 | + | NA | NA | NA | NA | NA |
| OTU2981 | NA | NA | + | NA | NA | NA |
| OTU3351 | NA | NA | NA | NA | + | NA |
| OTU6873 | NA | NA | NA | NA | NA | NA |
| OTU6364 | NA | NA | NA | NA | NA | NA |
| OTU2317 | + | NA | NA | + | NA | NA |
| OTU2457 | NA | + | NA | NA | NA | NA |
| OTU2994 | NA | NA | NA | + | NA | NA |
| OTU5859 | NA | NA | NA | NA | NA | + |
| OTU2182 | NA | NA | NA | NA | + | NA |
| OTU1696 | NA | NA | + | NA | NA | NA |
| OTU7009 | NA | NA | NA | + | NA | NA |
| OTU5158 | + | NA | NA | NA | NA | NA |
| OTU5155 | NA | NA | NA | NA | NA | NA |
| OTU7156 | NA | NA | + | NA | NA | NA |
| OTU769 | NA | NA | NA | + | NA | NA |
| OTU4277 | NA | NA | NA | + | NA | NA |
| OTU4215 | NA | + | NA | NA | NA | NA |
| OTU4253 | NA | NA | NA | + | NA | NA |
| OTU3428 | NA | NA | NA | + | NA | NA |
| OTU389 | NA | NA | + | NA | NA | NA |
| OTU1349 | NA | NA | NA | + | NA | NA |
| OTU1344 | + | NA | + | NA | NA | NA |
| OTU1347 | NA | NA | NA | NA | NA | + |
| OTU4467 | + | NA | NA | NA | NA | NA |
| OTU4469 | + | NA | NA | NA | NA | NA |
| OTU2385 | NA | NA | + | NA | NA | NA |
| OTU6015 | NA | NA | + | NA | NA | NA |
| OTU6542 | NA | NA | NA | NA | NA | + |
| OTU2390 | NA | NA | NA | NA | NA | NA |
| OTU7351 | NA | + | NA | NA | NA | NA |
| OTU3203 | NA | NA | + | NA | NA | NA |
| OTU3444 | NA | NA | NA | + | NA | NA |
| OTU3446 | NA | NA | NA | + | NA | NA |
| OTU3443 | NA | NA | NA | + | NA | NA |
| OTU2586 | NA | NA | NA | + | NA | NA |
| OTU6763 | NA | NA | NA | NA | + | NA |
| OTU6872 | NA | NA | + | NA | NA | NA |
| OTU5789 | NA | NA | + | NA | NA | NA |
| OTU6384 | NA | + | NA | NA | NA | NA |
| OTU3828 | NA | NA | NA | + | NA | NA |
| OTU611 | NA | NA | + | NA | NA | NA |
| OTU5259 | NA | NA | NA | NA | + | NA |
| OTU1224 | NA | NA | NA | NA | NA | NA |
| OTU3148 | + | NA | NA | NA | NA | NA |
| OTU4161 | NA | + | NA | NA | NA | NA |
| OTU4404 | + | NA | NA | NA | NA | NA |
| OTU3892 | NA | NA | + | NA | NA | NA |
| OTU3795 | + | NA | NA | NA | NA | NA |
| OTU4584 | NA | NA | NA | NA | + | NA |
| OTU5949 | NA | NA | NA | + | NA | NA |
| OTU4886 | + | NA | NA | NA | NA | NA |
| OTU4986 | NA | NA | NA | NA | NA | NA |
| OTU4906 | + | NA | NA | NA | NA | NA |
| OTU2268 | NA | NA | NA | NA | NA | + |
| OTU2819 | NA | + | NA | NA | NA | NA |
| OTU7860 | NA | NA | NA | + | NA | NA |
| OTU5008 | + | NA | NA | NA | NA | NA |
| OTU7012 | + | NA | NA | NA | NA | NA |
| OTU745 | NA | NA | NA | NA | NA | + |
| OTU2979 | NA | NA | + | NA | NA | NA |
| OTU7100 | NA | NA | + | NA | NA | NA |
| OTU1930 | NA | NA | NA | NA | NA | NA |
| OTU1796 | NA | NA | NA | NA | NA | NA |
| OTU2417 | NA | NA | NA | NA | NA | NA |
| OTU7150 | NA | NA | + | NA | NA | NA |
| OTU3994 | NA | NA | NA | + | NA | NA |
| OTU3226 | NA | NA | NA | NA | NA | NA |
| OTU3229 | NA | + | NA | NA | NA | NA |
| OTU7948 | NA | NA | + | NA | NA | NA |
| OTU780 | NA | NA | + | NA | NA | NA |
| OTU3111 | + | NA | NA | NA | NA | NA |
| OTU5871 | NA | NA | NA | NA | NA | + |
| OTU3419 | NA | NA | NA | + | NA | NA |
| OTU4655 | + | NA | NA | NA | + | NA |
| OTU4046 | NA | NA | NA | NA | NA | NA |
| OTU7074 | NA | NA | + | NA | NA | NA |
| OTU2355 | NA | NA | + | NA | NA | NA |
| OTU1215 | + | NA | NA | NA | NA | NA |
| OTU226 | NA | NA | + | NA | NA | NA |
| OTU4744 | NA | NA | NA | NA | NA | NA |
| OTU812 | NA | NA | + | NA | NA | NA |
| OTU7461 | NA | NA | NA | NA | + | NA |
| OTU2127 | NA | NA | NA | + | NA | NA |
| OTU8274 | NA | NA | + | NA | NA | NA |
| OTU4231 | NA | + | NA | NA | NA | NA |
| OTU4417 | + | NA | NA | NA | NA | NA |
| OTU4415 | + | NA | NA | NA | NA | NA |
| OTU6574 | NA | NA | NA | NA | NA | NA |
| OTU3473 | NA | NA | NA | + | NA | NA |
| OTU5616 | NA | NA | + | NA | NA | NA |
| OTU3824 | NA | NA | + | NA | NA | NA |
| OTU5614 | NA | + | NA | NA | NA | NA |
| OTU566 | NA | NA | + | NA | NA | NA |
| OTU2933 | NA | NA | NA | + | NA | NA |
| OTU3788 | NA | NA | NA | + | NA | NA |
| OTU7805 | NA | NA | NA | NA | + | NA |
| OTU3057 | NA | NA | NA | + | NA | NA |
| OTU3052 | NA | NA | NA | + | NA | NA |
| OTU7140 | NA | NA | + | NA | NA | NA |
| OTU5950 | NA | NA | NA | NA | NA | NA |
| OTU533 | NA | + | NA | NA | NA | NA |
| OTU3977 | NA | NA | + | NA | NA | NA |
| OTU6959 | NA | + | NA | NA | NA | NA |
| OTU6489 | NA | NA | NA | + | NA | NA |
| OTU4267 | NA | NA | NA | + | NA | NA |
| OTU6956 | NA | NA | NA | NA | NA | + |
| OTU4081 | NA | NA | + | NA | NA | NA |
| OTU3900 | NA | NA | NA | NA | NA | NA |
| OTU7065 | NA | NA | NA | NA | NA | NA |
| OTU5453 | + | NA | NA | NA | NA | NA |
| OTU5137 | NA | NA | NA | NA | NA | + |
| OTU8153 | NA | NA | NA | NA | NA | + |
| OTU7116 | NA | NA | + | NA | NA | NA |
| OTU6131 | NA | NA | NA | NA | NA | NA |
| OTU3085 | NA | NA | NA | NA | + | NA |
| OTU7962 | NA | NA | NA | NA | NA | NA |
| OTU6541 | NA | NA | + | NA | NA | NA |
| OTU8285 | + | NA | NA | + | NA | NA |
| OTU2839 | NA | NA | NA | NA | NA | NA |
| OTU6105 | NA | NA | NA | + | NA | NA |
| OTU6229 | NA | NA | NA | + | NA | NA |
| OTU681 | NA | NA | NA | NA | NA | NA |
| OTU4919 | NA | NA | NA | NA | NA | NA |
| OTU7615 | NA | + | NA | NA | NA | NA |
| OTU3201 | NA | + | + | NA | NA | NA |
| OTU5883 | NA | NA | NA | NA | NA | + |
| OTU3283 | NA | + | NA | NA | NA | NA |
| OTU8284 | NA | NA | NA | NA | NA | NA |
| OTU8289 | NA | NA | + | NA | NA | NA |
| OTU96 | NA | NA | NA | NA | NA | NA |
| OTU91 | NA | NA | NA | NA | NA | NA |
| OTU6563 | NA | NA | NA | NA | NA | + |
| OTU7452 | NA | NA | NA | NA | NA | + |
| OTU2302 | + | NA | NA | NA | NA | NA |
| OTU7498 | NA | NA | NA | NA | NA | NA |
| OTU8103 | NA | NA | + | NA | NA | NA |
| OTU4813 | NA | NA | NA | NA | + | NA |
| OTU5280 | NA | NA | NA | NA | NA | NA |
| OTU962 | NA | NA | NA | + | NA | NA |
| OTU6774 | NA | NA | NA | NA | NA | + |
| OTU2027 | + | NA | NA | NA | NA | NA |
| OTU554 | NA | NA | + | NA | NA | NA |
| OTU7073 | NA | NA | + | NA | NA | NA |
| OTU6547 | NA | NA | NA | NA | NA | NA |
| OTU5345 | NA | NA | NA | NA | + | NA |
| OTU4778 | NA | NA | NA | NA | + | NA |
| OTU2555 | NA | NA | NA | NA | NA | NA |
| OTU2520 | NA | NA | + | NA | NA | NA |
| OTU45 | NA | NA | + | NA | NA | NA |
| OTU40 | NA | NA | NA | NA | NA | + |
| OTU4880 | + | NA | NA | NA | NA | NA |
| OTU5864 | NA | NA | NA | NA | NA | NA |
| OTU4276 | NA | NA | NA | + | NA | NA |
| OTU638 | NA | NA | + | NA | NA | NA |
| OTU631 | NA | NA | + | NA | NA | NA |
| OTU2285 | NA | NA | NA | + | NA | NA |
| OTU600 | NA | NA | + | NA | NA | NA |
| OTU636 | NA | NA | + | NA | NA | NA |
| OTU8209 | + | NA | NA | NA | NA | NA |
| OTU1769 | NA | NA | + | NA | NA | NA |
| OTU1092 | + | NA | NA | NA | NA | NA |
| OTU528 | NA | NA | + | NA | NA | NA |
| OTU6081 | NA | NA | NA | + | NA | NA |
| OTU2918 | NA | NA | NA | + | NA | NA |
| OTU7871 | + | NA | NA | NA | NA | NA |
| OTU5036 | NA | NA | + | NA | NA | NA |
| OTU3665 | NA | NA | NA | + | NA | NA |
| OTU820 | NA | NA | NA | NA | NA | NA |
| OTU4292 | NA | NA | NA | + | NA | NA |
| OTU2085 | NA | NA | NA | NA | NA | + |
| OTU1036 | NA | NA | + | NA | NA | NA |
| OTU4073 | NA | NA | + | NA | NA | NA |
| OTU4787 | NA | NA | NA | NA | + | NA |
| OTU5060 | NA | + | NA | NA | NA | NA |
| OTU6258 | NA | + | NA | NA | NA | NA |
| OTU3950 | NA | NA | NA | NA | NA | NA |
| OTU4304 | NA | NA | NA | + | NA | NA |
| OTU5860 | NA | NA | NA | NA | NA | + |
| OTU5869 | NA | NA | NA | NA | NA | + |
| OTU5687 | + | + | NA | NA | NA | NA |
| OTU806 | NA | NA | + | NA | NA | NA |
| OTU4162 | NA | + | NA | NA | NA | NA |
| OTU88 | NA | NA | NA | + | NA | NA |
| OTU2166 | NA | NA | NA | NA | + | NA |
| OTU4628 | NA | NA | NA | NA | NA | NA |
| OTU4905 | NA | NA | NA | NA | + | NA |
| OTU4406 | + | NA | NA | NA | NA | NA |
| OTU5359 | NA | NA | + | NA | NA | NA |
| OTU3667 | + | NA | NA | NA | NA | NA |
| OTU3361 | NA | NA | NA | NA | NA | NA |
| OTU2265 | NA | NA | NA | NA | + | NA |
| OTU5944 | NA | + | + | NA | + | NA |
| OTU1912 | + | NA | NA | NA | NA | NA |
| OTU6676 | NA | NA | + | NA | NA | NA |
| OTU2665 | NA | NA | NA | NA | NA | NA |
| OTU7691 | NA | NA | NA | + | + | NA |
| OTU2181 | NA | NA | NA | + | NA | NA |
| OTU8194 | NA | NA | NA | NA | NA | NA |
| OTU3134 | + | + | NA | NA | NA | NA |
| OTU7072 | NA | NA | + | NA | NA | NA |
| OTU874 | NA | NA | NA | + | NA | NA |
| OTU1490 | NA | NA | NA | NA | NA | NA |
| OTU607 | NA | NA | + | NA | NA | NA |
| OTU832 | NA | NA | + | NA | NA | NA |
| OTU1043 | NA | NA | NA | + | NA | NA |
| OTU2219 | NA | NA | NA | NA | + | NA |
| OTU3288 | NA | NA | NA | NA | NA | + |
| OTU2709 | NA | NA | NA | NA | + | NA |
| OTU4436 | + | NA | NA | NA | NA | NA |
| OTU4489 | NA | NA | NA | NA | NA | NA |
| OTU5474 | + | NA | NA | NA | NA | NA |
| OTU4818 | NA | NA | NA | NA | + | NA |
| OTU7134 | NA | NA | + | NA | NA | NA |
| OTU4482 | NA | NA | NA | NA | NA | NA |
| OTU4485 | + | NA | NA | NA | NA | NA |
| OTU6569 | NA | NA | NA | NA | NA | + |
| OTU6568 | NA | NA | NA | NA | NA | + |
| OTU6561 | NA | NA | NA | NA | NA | + |
| OTU6566 | NA | NA | NA | NA | NA | + |
| OTU2187 | NA | NA | NA | NA | NA | NA |
| OTU4923 | + | NA | NA | NA | NA | NA |
| OTU3426 | NA | NA | NA | + | NA | NA |
| OTU3424 | NA | NA | NA | + | NA | NA |
| OTU5478 | + | NA | NA | NA | NA | NA |
| OTU5870 | NA | NA | NA | NA | NA | + |
| OTU5473 | + | NA | NA | NA | NA | NA |
| OTU2224 | NA | NA | NA | NA | NA | NA |
| OTU5647 | NA | NA | NA | + | + | NA |
| OTU1009 | NA | + | NA | NA | NA | NA |
| OTU3392 | NA | NA | NA | + | NA | NA |
| OTU3976 | NA | NA | NA | NA | NA | NA |
| OTU4011 | NA | + | NA | NA | NA | NA |
| OTU884 | NA | NA | NA | + | NA | NA |
| OTU3093 | NA | NA | NA | + | NA | NA |
| OTU3464 | NA | NA | NA | + | NA | NA |
| OTU6658 | NA | NA | NA | NA | NA | + |
| OTU889 | NA | NA | + | NA | NA | NA |
| OTU5837 | NA | NA | NA | NA | + | NA |
| OTU4817 | NA | NA | NA | NA | + | NA |
| OTU4257 | NA | NA | NA | + | NA | NA |
| OTU5853 | NA | NA | NA | NA | NA | + |
| OTU5852 | NA | NA | NA | NA | NA | + |
| OTU1883 | NA | NA | + | NA | NA | NA |
| OTU872 | NA | NA | + | NA | NA | + |
| OTU4596 | NA | NA | NA | NA | + | NA |
| OTU4779 | NA | NA | NA | NA | + | NA |
| OTU499 | NA | NA | + | NA | NA | NA |
| OTU3848 | NA | NA | NA | + | NA | NA |
| OTU408 | NA | NA | NA | + | NA | NA |
| OTU2138 | NA | NA | NA | NA | + | NA |
| OTU4165 | NA | + | NA | NA | NA | NA |
| OTU5108 | NA | NA | NA | NA | + | NA |
| OTU4305 | NA | NA | NA | + | NA | NA |
| OTU7612 | NA | + | NA | NA | NA | NA |
| OTU4160 | NA | NA | NA | NA | NA | NA |
| OTU2877 | NA | NA | NA | + | NA | NA |
| OTU2872 | NA | NA | NA | NA | NA | NA |
| OTU2871 | NA | NA | + | NA | NA | NA |
| OTU2841 | NA | NA | NA | NA | NA | NA |
| OTU6993 | NA | NA | NA | + | NA | NA |
| OTU5971 | NA | NA | NA | NA | NA | + |
| OTU4587 | NA | + | NA | NA | NA | NA |
| OTU8184 | NA | NA | NA | NA | NA | NA |
| OTU988 | NA | NA | + | NA | NA | NA |
| OTU6971 | NA | NA | NA | NA | NA | NA |
| OTU1307 | NA | NA | + | NA | NA | NA |
| OTU980 | NA | NA | NA | NA | NA | NA |
| OTU3018 | NA | NA | + | NA | NA | NA |
| OTU7026 | NA | NA | + | NA | NA | NA |
| OTU2503 | NA | NA | NA | NA | + | NA |
| OTU3642 | NA | NA | NA | NA | + | NA |
| OTU2785 | NA | NA | NA | NA | NA | + |
| OTU7058 | NA | NA | + | NA | NA | NA |
| OTU7056 | NA | NA | + | NA | NA | NA |
| OTU6759 | NA | NA | + | NA | NA | NA |
| OTU2742 | NA | NA | NA | NA | NA | NA |
| OTU3089 | NA | NA | NA | NA | NA | NA |
| OTU5303 | NA | NA | NA | + | NA | NA |
| OTU6608 | NA | NA | + | NA | NA | NA |
| OTU61 | + | NA | NA | NA | NA | NA |
| OTU7088 | NA | NA | + | NA | NA | NA |
| OTU6387 | NA | NA | NA | NA | NA | + |
| OTU1964 | NA | NA | + | NA | NA | NA |
| OTU1781 | NA | NA | NA | NA | NA | NA |
| OTU4815 | NA | NA | NA | NA | + | NA |
| OTU5026 | + | NA | NA | NA | NA | NA |
| OTU4900 | + | NA | NA | NA | NA | NA |
| OTU5247 | NA | NA | NA | NA | + | NA |
| OTU1153 | NA | NA | + | NA | NA | NA |
| OTU30 | NA | NA | + | NA | NA | NA |
| OTU8143 | NA | NA | NA | + | NA | NA |
| OTU5917 | NA | NA | NA | NA | NA | + |
| OTU572 | NA | NA | NA | + | NA | NA |
| OTU7455 | + | NA | NA | NA | NA | NA |
| OTU5675 | NA | NA | NA | NA | NA | NA |
| OTU4538 | + | NA | NA | NA | NA | NA |
| OTU2121 | NA | NA | NA | + | NA | NA |
| OTU1670 | NA | NA | NA | NA | NA | NA |
| OTU1235 | NA | NA | + | NA | NA | NA |
| OTU2129 | NA | NA | NA | + | NA | NA |
| OTU7301 | NA | NA | + | NA | NA | NA |
| OTU244 | NA | NA | NA | NA | NA | NA |
| OTU757 | NA | NA | NA | NA | NA | NA |
| OTU1392 | NA | NA | NA | NA | NA | NA |
| OTU1395 | NA | NA | + | NA | NA | NA |
| OTU3740 | NA | NA | NA | NA | NA | NA |
| OTU7517 | NA | NA | NA | NA | NA | + |
| OTU7514 | NA | NA | NA | NA | NA | + |
| OTU6344 | NA | NA | + | NA | NA | NA |
| OTU5874 | NA | NA | NA | NA | NA | NA |
| OTU7519 | NA | NA | NA | NA | NA | NA |
| OTU7518 | NA | NA | NA | NA | NA | NA |
|  |  |  |  |  |  |  |

**Table S4.** Abbreviations for the top 30 species of soil bacteria and fungi at family and genus levels.

| microorganism | Abbreviation | Genus | Abbreviation | Species |
| --- | --- | --- | --- | --- |
| Bacteria | Gp1 | Udaeobacter | Sp1 | Udaeobacter |
|  | Gp2 | Subgroup_2 | Sp2 | unclassified_g__Bradyrhizobium |
|  | Gp3 | Bradyrhizobium | Sp3 | Rokubacteriales |
|  | Gp4 | Rokubacteriales | Sp4 | Vicinamibacterales |
|  | Gp5 | Acidobacteriales | Sp5 | unclassified_g__norank_f__norank_o__Subgroup_2 |
|  | Gp6 | Gemmataceae | Sp6 | Subgroup_2 |
|  | Gp7 | Vicinamibacterales | Sp7 | unclassified_g__norank_f__Gemmataceae |
|  | Gp8 | Xanthobacteraceae | Sp8 | AD3 |
|  | Gp9 | Gaiellales | Sp9 | unclassified_k__norank_d__Bacteria |
|  | Gp10 | AD3 | Sp10 | Udaeobacter |
|  | Gp11 | Elsterales | Sp11 | unclassified_g__norank_f__norank_o__Gaiellales |
|  | Gp12 | unclassified_k__norank_d__Bacteria | Sp12 | unclassified_g__Burkholderia-Caballeronia-Paraburkholderia |
|  | Gp13 | Xiphinematobacter | Sp13 | Mycobacterium |
|  | Gp14 | Mycobacterium | Sp14 | unclassified_g__Candidatus_Udaeobacter |
|  | Gp15 | Burkholderia-Caballeronia-Paraburkholderia | Sp15 | unclassified_g__norank_f__norank_o__Acidobacteriales |
|  | Gp16 | Gemmatimonadaceae | Sp16 | Xanthobacteraceae |
|  | Gp17 | IMCC26256 | Sp17 | Elsterales |
|  | Gp18 | RCP2-54 | Sp18 | Xiphinematobacter |
|  | Gp19 | Bryobacter | Sp19 | norank |
|  | Gp20 | Pseudolabrys | Sp20 | unclassified_g__norank_f__Xanthobacteraceae |
|  | Gp21 | Methyloligellaceae | Sp21 | _Methyloligellaceae |
|  | Gp22 | Solibacter | Sp22 | RCP2-54 |
|  | Gp23 | RB41 | Sp23 | unclassified_g__Pseudolabrys |
|  | Gp24 | Gaiella | Sp24 | unclassified_g__mle1-7 |
|  | Gp25 | Subgroup_7 | Sp25 | unclassified_f__Xanthobacteraceae |
|  | Gp26 | Alphaproteobacteria | Sp26 | unclassified_c__Alphaproteobacteria |
|  | Gp27 | Acidibacter | Sp27 | Gemmataceae |
|  | Gp28 | mle1-7 | Sp28 | Xanthobacteraceae |
|  | Gp29 | KD4-96 | Sp29 | WD260 |
|  | Gp30 | Acidothermus | Sp30 | KD4-96 |
| Fungi | Gp1 | Russula | Sp1 | Sebacina_sp |
|  | Gp2 | Mortierella | Sp2 | unclassified_g__Russula |
|  | Gp3 | Sebacina | Sp3 | vesca |
|  | Gp4 | Solicoccozyma | Sp4 | elongata |
|  | Gp5 | unclassified_f__Hyaloscyphaceae | Sp5 | terricola |
|  | Gp6 | Saitozyma | Sp6 | variata |
|  | Gp7 | Clavulina | Sp7 | Saitozyma_sp |
|  | Gp8 | Piloderma | Sp8 | Hyaloscyphaceae_sp |
|  | Gp9 | unclassified_k__Fungi | Sp9 | humilis |
|  | Gp10 | Delastria | Sp10 | unclassified_g__Mortierella |
|  | Gp11 | Amanita | Sp11 | Clavulina_sp |
|  | Gp12 | unclassified_p__Ascomycota | Sp12 | Piloderma_sp |
|  | Gp13 | unclassified_p__Rozellomycota | Sp13 | Russula_cyanoxantha |
|  | Gp14 | Leotia | Sp14 | unclassified_k__Fungi |
|  | Gp15 | unclassified_o__Agaricales | Sp15 | Delastria_sp |
|  | Gp16 | unclassified_o__Sordariales | Sp16 | unclassified_p__Ascomycota |
|  | Gp17 | unclassified_o__GS11 | Sp17 | rosea |
|  | Gp18 | Trichoderma | Sp18 | lubrica |
|  | Gp19 | Lactarius | Sp19 | longistriata |
|  | Gp20 | Entoloma | Sp20 | unclassified_o__Agaricales |
|  | Gp21 | Descolea | Sp21 | unclassified_o__Sordariales |
|  | Gp22 | unclassified_f__Thelephoraceae | Sp22 | GS11_sp |
|  | Gp23 | Tremellodendron | Sp23 | terrea |
|  | Gp24 | Penicillium | Sp24 | pseudozygospora |
|  | Gp25 | Trechispora | Sp25 | unclassified_g__Entoloma |
|  | Gp26 | unclassified_o__Helotiales | Sp26 | Descolea_sp |
|  | Gp27 | unclassified_c__Sordariomycetes | Sp27 | virescens |
|  | Gp28 | Cladophialophora | Sp28 | acrifolia |
|  | Gp29 | Cantharellus | Sp29 | unclassified_p__Rozellomycota |
|  | Gp30 | Archaeorhizomyces | Sp30 | schweinitzii |
